# Diversity in the Toll-Like Receptor Genes of the African Penguin (*Spheniscus demersus*)

**DOI:** 10.1371/journal.pone.0163331

**Published:** 2016-10-19

**Authors:** Desiré Lee Dalton, Elaine Vermaak, Marli Roelofse, Antoinette Kotze

**Affiliations:** 1 Centre for Conservation Science, National Zoological Gardens of South Africa, Pretoria, Gauteng, South Africa; 2 Genetics Department, University of the Free State, Bloemfontein, Free State, South Africa; University of Illinois at Urbana-Champaign, UNITED STATES

## Abstract

The African penguin, *Spheniscus demersus*, is listed as Endangered by the IUCN Red List of Threatened Species due to the drastic reduction in population numbers over the last 20 years. To date, the only studies on immunogenetic variation in penguins have been conducted on the major histocompatibility complex (MHC) genes. It was shown in humans that up to half of the genetic variability in immune responses to pathogens are located in non-MHC genes. Toll-like receptors (TLRs) are now increasingly being studied in a variety of taxa as a broader approach to determine functional genetic diversity. In this study, we confirm low genetic diversity in the innate immune region of African penguins similar to that observed in New Zealand robin that has undergone several severe population bottlenecks. Single nucleotide polymorphism (SNP) diversity across TLRs varied between *ex situ* and *in situ* penguins with the number of non-synonymous alterations in *ex situ* populations (n = 14) being reduced in comparison to *in situ* populations (n = 16). Maintaining adaptive diversity is of vital importance in the assurance populations as these animals may potentially be used in the future for re-introductions. Therefore, this study provides essential data on immune gene diversity in penguins and will assist in providing an additional monitoring tool for African penguin in the wild, as well as to monitor diversity in *ex situ* populations and to ensure that diversity found in the *in situ* populations are captured in the assurance populations.

## Introduction

The African or “jackass” penguin, *Spheniscus demersus*, is endemic to southern Africa with breeding sites distributed at 28 locations in South Africa and Namibia [1, 2, 3]. The population has experienced a long-term decline since the late 1800s, with numbers continually decreasing steeply in recent years [4]. In 2010, the species was changed from vulnerable to endangered by the IUCN Red List of Threatened Species [5] due to the reduction in population numbers. The population size was estimated at less than 26 000 breeding pairs in 2009 from an estimated 141 000 breeding pairs in 1956–1957, equating to a decline of 60.5% over 28 years [6, 7]. Population declines have been attributed to competition for food with commercial fisheries, whereby adult penguins have to travel longer distances in order to find sufficient food which can result in starvation and increases the likelihood of chick predation [8]. Predators of African penguin include seals, sharks and terrestrial predators [9]. Additional threats include habitat and nest destruction due to historical guano harvesting, and interspecific competition for nesting sites [10]. Oiling has had a devastating effect on the African penguin populations with 13 major oil spills being reported in South Africa since 1948 that affected the species [11]. The increased accessibility of tourists to penguin habitat may intensify the likelihood of pathogen introductions to susceptible penguin species which in turn may be another reason for the population decline [1, 2, 12, 13].

It has been hypothesised that in order to maintain population fitness, a population requires high genetic diversity. Low genetic diversity has long been associated with inbreeding depression and a reduction in the survival of species [14, 15]. Conservation genetic studies to determine genetic diversity of individuals and populations using neutral markers such as microsatellites are well known. In African penguin, genetic diversity has been reported to be similar to levels determined for other species of penguin [16]. In addition, genetic diversity in the *ex situ* African penguin population was found to be comparable to the *in situ* populations in terms of H_o_, H_e_ and H_Z_ [16]. However, although these markers can be used effectively to determine population structure and gene flow, they may not be relevant to determine the degree of functional diversity. Studies have previously focused on the analysis of the major histocompatibility complex (MHC) which provides information for individual and population viability due to their direct association with immune function. The analysis of MHC loci is challenging, however, in non-model organisms due to the high number of pseudogenes and duplications which interfere with genetic diversity estimates [17]. Taking a broader approach to the analysis of wildlife functional genetic diversity beyond MHC is therefore an option. Toll-like receptor (TLR) genes are highly conserved, can be amplified from diverse avian species, and are responsible for initiating innate and acquired immune responses due to recognition of a wide variety of pathogens. TLRs are therefore an attractive tool to investigate specific loci relevant for immune system function [18, 19, 20]. There is currently limited knowledge of disease outbreaks in *in situ* populations of penguins and in several cases the identification of the causative agent has been unsuccessful [21]. The African penguin has, however, been reported to be susceptible to avian malaria, a serious infectious disease and the major cause of mortality in *ex situ* penguins for example [22]. In addition, a variety of viruses have been detected in penguin, including avian pox virus [23], Newcastle disease virus [24] and papillomavirus [25]. Sequence variations in the TLRs have been associated with variation in resilience to disease and infection and can influence the survival of species [26]. Expression of TLRs is variable among host tissues [27]. In mammals, 13 TLRs have been identified (*TLR1-13*) and have been organised into six major groups based on phylogenetic analysis namely; *TLR2* group (*TLRs 1*, *2*, *6*, *10*), *TLR3* group, *TLR4* group, *TLR5* group, *TLR 7/8/9* group and *TLR11* group (*TLRs 11*, *12*, *13*) [28]. Thus far, ten avian TLRs (*TLR1LA*, *TLR1LB*, *TLR2A*, *TLR2B*, *TLR3*, *TLR4*, *TLR5*, *TLR7*, *TLR15* and *TLR21*) have been reported of which four genes (*TLR3*, *4*, *5* and 7) have orthologs in other vertebrate groups [19]. *TLR15* appears to be unique to avian and reptile species [29, 30], however, it is phylogenetically related to *TLR2*.

In this study, we determined the levels of TLR diversity in both *in situ* and *ex situ* African penguins to gain a more comprehensive understanding of innate immunity, as well as to develop new measures of functional diversity to assist in the management of the species. There are several genetic concerns that should be taken into account for the management of *ex situ* penguin populations. Since *ex situ* populations are derived from a small number of individuals, these populations face the similar threats to small and isolated natural populations which may jeopardise the ability of *ex situ* populations to reproduce and survive when returned to the wild. In addition, research has demonstrated that inbred individuals have lower resistance to disease [31]. Thus functional diversity needs to be considered in management plans for small and isolated *ex situ* populations. *TLR1LA*, *TLR1LB*, *TRL2*, *TLR5* and *TLR7* were targeted in order to encompass an array of ligand/pathogen-associated molecular patterns (PAMPs) specificities, namely di-and triacylated lipoproteins found in the cell wall of bacteria, fungi and parasites recognised by *TLR2* and members of the *TLR1/6/10* family, flagellins of flagellated bacteria recognised by *TLR5* and single-stranded viral RNA detected by *TLR7* [19, 32, 33, 34, 35, 36]. We hypothesise that both the *ex situ* and *in situ* African penguin populations will show a reduced variation at the majority of the TLR loci due to the significant population declines and bottlenecks. To our knowledge this is the first study to analyse TLR diversity in African penguin populations.

## Materials and Methods

### Sample collection

**Fig 1:**
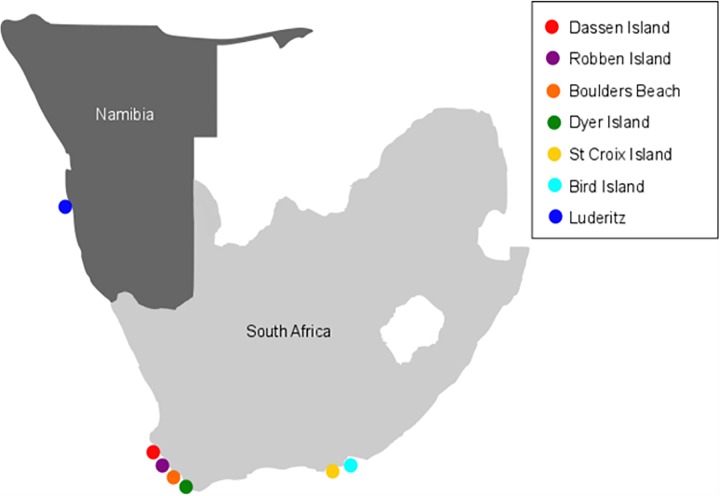
Map indicating the respective sampling localities of this study of *in situ* African penguins in Southern Africa.

Blood samples were collected from 20 African penguins in South Africa from three breeding facilities namely: Two Oceans Aquarium (n = 7), uShaka Marine World (n = 6) and National Zoological Gardens of South Africa (NZG; n = 7). Currently, penguin populations are being kept in zoo and aquarium facilities throughout South Africa. As part of the management plan for this species, a Pan-African Association of Zoos and Aquaria (PAAZA) regional studbook is maintained by the NZG. The African regional studbook for the African penguin uses the Single Population Analysis and Record Keeping System (SPARKS) developed by the International Species Information System (ISIS) and the PM2000 database programme. Based on studbook information, only unrelated adult birds were selected and were included in this study. In addition, 21 samples were collected by SANCCOB from *in situ* colonies (Fig 1) at the following locations: Namibia (Lüderitz; n = 6; 26.6420° S, 15.1639° E), Dassen Island (n = 3; 33.4236° S, 18.0865° E), Bird Island (n = 5; 32.0901° S, 18.3026° E), Robben Island (n = 4; 33.8076° S, 18.3712° E), Dyer Island (n = 5; 34.5805° S, 19.3518° E), Boulders Beach (n = 4; 34.1972° S, 18.4513° E) and St Croix (n = 4; 33.5013° S, 26.1648° E). All necessary research and ethics permits were approved for the collection of samples (South African Department of Environmental Affairs permit number: RES2010/66). The NZG Research and Ethics Scientific Committee approved this study.

### Genomic DNA Isolation, Amplification and Sequencing

DNA extraction was conducted using the ZR Genomic DNA^™^ -Tissue Miniprep kit (Zymo Research), according to the manufacturer’s protocol. Primers developed for members of Apterygiformes, Gruiformes, Psittaciformes and Passeriformes [19] (S1 Table), were used to target portions of five TLR gene regions, namely *TLR1LA*, *TLR1LB*, *TLR2*, *TLR5* and *TLR7*. Amplification was carried out in separate PCR reactions consisting of 1 × DreamTaq Green PCR Master Mix, 0.4 μM of each primer, and approximately 20 ng template DNA in a total volume of 20 μl. The temperature profile was as follows: an initial denaturation at 95°C for 3 min, 35 cycles of 95°C for 30 s, 53–58°C for 30 s, and 72°C for 1 min, followed by a final extension at 72°C for 10 min. Successful PCR products were purified with Exonuclease I and FastAP (Thermo Fisher Scientific Inc.). Gene fragments were sequenced in both directions using the BigDye Terminator v3.1 Cycle Sequencing Kit and visualised on a 3500 Genetic Analyzer (Applied Biosystems). Sequence chromatograms were edited and assembled using Geneious v.8.0.3 (created by Biomatters).

### Diversity Estimates

Differences in mean observed heterozygosity (H_o_), mean expected heterozygosity (H_e_), and unbiased expected heterozygosity (H_z_) were determined using GenAlEx [37] and included synonymous and non-synonymous mutations. The number of SNPs, the number of haplotypes, haplotype diversity and nucleotide diversity were determined in DnaSP v.5.10.01. The number of alleles was determined in Arlequin v.3.5.2.2.

### Phylogenetic Analyses

Sequence alignments were generated using Clustal W [38] in BioEdit v.7.0.9.0 [39] and were visually inspected. The sample dataset was supplemented with sequences from *Atlapetes pallidiceps* (*TLR1LA*: KM095968.1; *TLR1LB*: KM096012.1), *Carpodacus mexicanus* (*TLR1LA*: GU904991.1; *TLR1LB*: GU904945.1; *TLR7*: GU904978.1), *Falco naumanni* (*TLR1LA*: GU904990.1; *TLR1LB*: GU904944.1; *TLR5*: GU904973.1), *Petroica austrakis rakiura* (*TLR1LA*: JX502625.1; *TLR1LB*: JX502628.1; *TLR2*: JX502631.1; *TLR5*: JX502645.1; *TLR7*: JX502660.1), *Aptenodytes forsteri* (*TLR1LB*: XM_009280152.1; *TLR2*: XM_009288440.1), *Pygoscelis adeliae* (*TLR2*: XM_009319611.1; *TLR5*: XM_009333665.1) and *Fulmarus glacialis* (*TLR5*: XM_009572390.1; *TLR7*: XM_009572361.1), obtained from NCBI Genbank to facilitate a more robust phylogenetic analyses. Distance-based analyses (Neighbor-joining, NJ) of the final dataset was conducted in MEGA v.6.06 using p-distance estimates with nodal support being assessed through 10 000 non-parametric bootstrap replications.

### Identification of SNPs

Synonymous and non-synonymous SNP variations were determined by translating the TLR gene nucleotide sequences to the longest open reading frames. The identity and integrity of the respective amino acid sequences were confirmed by standard protein BLAST. Amino acid variations were visually inspected using BioEdit v.7.0.9.0 [39].

## Results and Discussion

### Amplification of TLR genes in African penguin

**Fig 2:**
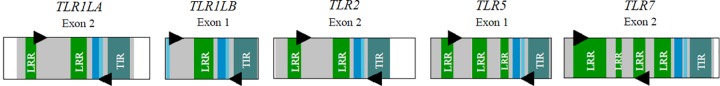
Schematic representation of the structure of the targeted TLR genes (adapted from Temperley *et al*., 2008; Alcaide and Edwards, 2011). Exons are represented by boxes. Arrow heads denote the position of the primers used in this study. Coloured areas designate coding regions, whereas white areas are non-coding regions. The gene regions that code for conserved domains of the protein are represented by different colours [Green, leucine-rich repeat (LRR) domains; dark blue, C-terminal LRR domains; light blue, transmembrane region; teal, cytoplasmic Toll/interleukin I resistance (TIR) domain].

Genes *TLR1LA*, *TLR1LB*, *TLR2*, *TLR5* and *TLR7* amplified in all penguin samples (S2 Table). Based on the well characterized chicken (*Gallus gallus*) TLR gene sequences [29], the successfully amplified penguin TLR gene regions were found to encode key functional conserved residues in exons (Fig 2), where variability is associated with pathogen binding [40, 41, 42]. Coding sequences ranged from 564 to 1082 bp. As reported for the New Zealand robin (*Petrocia australis rakiura*) [43], co-amplification of duplicate loci for *TLR7* was observed in this study. Due to the amplification of a *TLR7* pseudogene, evidenced by the presence of premature stop codons, subsequent analyses of *TLR7* was consequently omitted. An excess of heterozygosity at SNPs within the *TLR1LA*, *TLR1LB*, *TLR2* and *TLR5* gene regions was not observed providing evidence that duplicate copies are either not present or were not amplified in the case of these genes for the African penguin. In addition, it is unlikely that pseudogenes were co-amplified as there was an absence of stop codons and disrupted reading frames [19]. All SNPs observed were diallelic.

**Fig 3:**
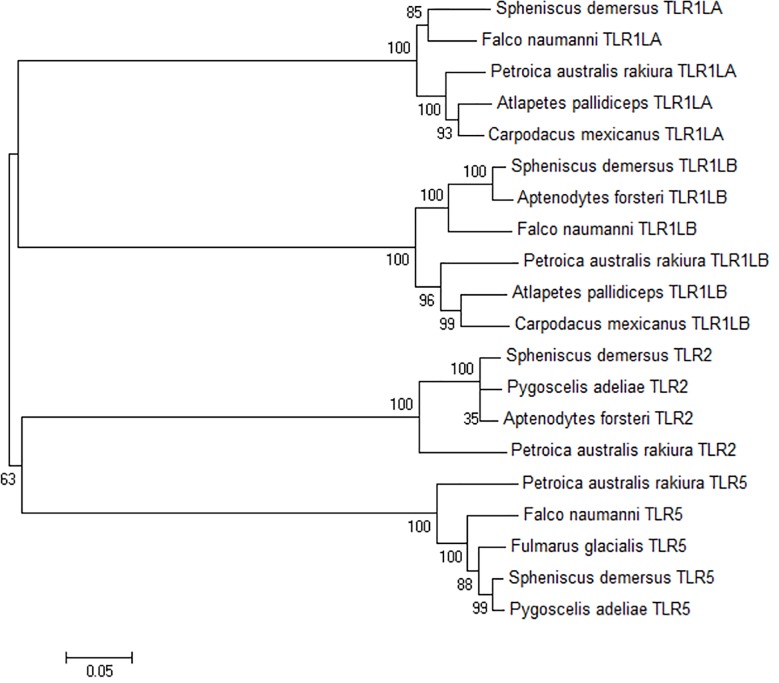
Combined neighbour-joining phylogenetic analysis of the *TLR1LA*, *TLR1LB*, *TLR2* and *TLR5* genes of the African penguin (*Spheniscus demersus*). Supplementary sequences from other bird species are specified. Bootstrap values are indicated at each branch point.

The phylogenetic relationships of African penguin TLR genes are depicted in Fig 3, and resemble those previously described [19]. *TLR1LA* shares a 92% sequence identity to lesser kestrel (*Falco naumanni*); *TLR1LB* shares a 97% sequence identity to Emperor penguin (*Aptenodytes forsteri*) and a 91% sequence identity to lesser kestrel (*Falco naumanni*). *TLR2* shares a 97% sequence identity to both emperor penguin (*Aptenodytes forsteri*) and Adelie penguin (*Pygoscelis adeliae*) and a 94% sequence identify to northern fulmar (*Fulmarus galcialis*). *TLR5* shared a 99% and 98% sequence identity to two penguin species, namely emperor penguin (*Aptenodytes forsteri*) and Adelie penguin (*Pygoscelis adeliae*), respectively, and a 96% sequence identity to northern fulmar (*Fulmarus galcialis*). Conservation of these regions among a wide range of bird species (Fig 3) provides support that each TLR gene is functionally conserved.

### Level of polymorphism

Polymorphisms were detected in all African penguin TLR genes. A subset of ten *in situ* African penguin samples was selected at random in order to compare polymorphism statistics between this species and three other avian species (Table 1). Analyses included a threatened species that has undergone several population bottlenecks (New Zealand robin [*Petroica australis rakiura*]) as well as more common species; house finch (*Carpodacus mexicanus*) and lesser kestrel (*Falco naumanni*) (Table 1). House finch (*Carpodacus mexicanus*) has undergone a brief but severe demographic bottleneck that was followed by population growth and range expansion [44]. The lesser kestrel (*Falco naumanni*) has experienced population declines and has been reported to be extinct in several locations throughout its breeding range resulting in fragmented populations with restricted gene flow due to isolation [19]. Levels of TLR polymorphism in these two species has been reported as low to moderate [19]. New Zealand robin (*Petroica australis rakiura*) has experienced several severe bottlenecks. The New Zealand robin found on Stewart Island has experienced two bottlenecks over the last four centuries [43]. Diversity estimates for African penguin (*h* = 2–4, π = 0.0002–0.0020) and New Zealand robin (*h* = 2–3, π = 0.0005–0.0021) were comparable and were low compared to more common species; house finch (*h* = 3–62, π = 0.0001–0.0078) and lesser kestrel (*h* = 3–16, π = 0.0024–0.0043; Table 1).

**Table 1:** Comparison of four TLR polymorphisms between 10 *in situ* African penguins and other birds species.

Species	Genes	N^1^	SNPs	*h*^2^	π^3^	Reference
African penguin (*Spheniscus demersus*)	*TLR1LA*	10	4	4	0.0014	This study
	*TLR1LB*	10	2	3	0.0005	
	*TLR2*	10	4	3	0.0020	
	*TLR5*	10	1	2	0.0002	
New Zealand robin (*Petroica australis rakiura*)	*TLR1LA*	10	2	2	0.0009	[41]
	*TLR1LB*	10	3	2	0.0016	
	*TLR2*	10	5	3	0.0021	
	*TLR5*	10	2	3	0.0005	
Lesser kestrel (*Falco naumanni*)	*TLR1LA*	8	19	11	0.0039	[23]
	*TLR1LB*	8	16	15	0.0039	
	*TLR2*	8	2	3	0.0024	
	*TLR5*	8	20	16	0.0043	
House finch (*Carpodacus mexicanus*)	*TLR1LA*	51	44	62	0.0058	[23]
	*TLR1LB*	8	25	20	0.0067	
	*TLR2*	8	11	11	0.0078	
	*TLR5*	8	2	3	0.0001	

^1^ N: number of samples

^2^ *h*: the number of inferred haplotypes

^3^ π: mean nucleotide diversity

In this study we were able to determine the number per site of non-synonymous (d_N_) and synonymous (d_S_) alterations. Non-synonymous alterations (encoding different amino acid residues) were observed in all loci analysed for the *in situ* African penguin populations (Table 2, d_N_/d_S_ = 6.33) and in the *ex situ* populations (d_N_/d_S_ = 3.86). Non-synonymous alterations have been reported in African penguin (d_N_/d_S_ = 16) for the MHC class II DRB-like gene [45]. In general, TLR loci are reported to be not as polymorphic as MHC genes [46, 47]. An excess of synonymous over non-synonymous alterations has been identified in several species due to functional constraints in each TLR gene due to purifying selection [48]. However, balancing selection and positive selection have been reported in a smaller number of studies [19, 49]. In the case of the African penguin, an excess of non-synonymous over synonymous SNPs was the general pattern found at each TLR loci studied, indicating positive selection. This observation may be due to only non-viral TLR loci being included in this study. Non-viral TLRs more easily tolerate non-synonymous mutations which can be subject to positive selection [50]. The higher tolerance of mutations is reported to be due to the redundant function of non-viral TLRs (several surface TLRs are able to recognize the same bacteria and fungi components, thus one microorganism can be recognised by several TLRs), thus a non-synonymous mutation in one TLR does not necessarily mean the loss of the function and does not compromise immunity [50].

**Table 2:** Polymorphisms in African penguin TLRs. Synonymous SNPs indicated outside of parentheses and non-synonymous SNPs in the coding regions indicated in parentheses.

Population	Description	*TLR1LA*	*TLR1LB*	*TLR2*	*TLR5*	Total
Two Oceans Aquarium	*ex situ*	0 (1)	0 (0)	0 (2)	0 (0)	0 (3)
uShaka Marine World	*ex situ*	2 (3)	0 (2)	0 (2)	0 (0)	2 (7)
NZG^1^	*ex situ*	0 (2)	1 (5)	0 (1)	0 (1)	1 (9)
**Total**		**2 (4)**	**1 (5)**	**0 (4)**	**0 (1)**	**3 (14)**
Namibia (Lüderitz)	*in situ*	0 (0)	0 (1)	0 (2)	0 (0)	0 (3)
Dassen Island	*in situ*	1 (0)	0 (0)	0 (0)	0 (0)	1 (0)
Bird Island	*in situ*	1 (2)	0 (0)	0 (0)	0 (0)	1 (2)
Robben Island	*in situ*	1 (5)	1 (2)	0 (1)	0 (1)	2 (9)
Dyer Island	*in situ*	0 (2)	0 (2)	0 (0)	0 (1)	0 (5)
Boulders Beach	*in situ*	0 (2)	1 (0)	1 (1)	0 (0)	2 (3)
St Croix	*in situ*	1 (5)	0 (0)	0 (0)	0 (0)	1 (5)
**Total**		**3 (8)**	**1 (3)**	**1 (4)**	**0 (1)**	**5 (16)**

^1^ NZG: National Zoological Gardens of South Africa

### SNP diversity across *ex situ* and *in situ* African penguins

TLR polymorphisms varied between *ex situ* and *in situ* penguins (Table 2). *TLR2* and *TLR5* had the lowest diversity in all penguins. In humans, alterations at these loci have been associated with sepsis [51] and susceptibility to Legionnaires’ disease [52], respectively. The highest number of SNPs was observed in *TLRLA* followed by *TLR1LB*. *TLR1LA* has been reported to cover functions of both *TLR1* and *TLR6* in mammals and is found to localize on the cell surface [53]. Heterozygosity estimates between *ex situ* (H_o_ = 0.260, H_e_ = 0.249 and H_z_ = 0.269) and *in situ* (H_o_ = 0.226, H_e_ = 0.198 and H_z_ = 0.238) populations were similar (Table 3), however the number of non-synonymous alterations in *ex situ* populations (n = 14) is slightly reduced in comparison to *in situ* populations (n = 16). It has been postulated that diversity in TLRs is required for genetic fitness and long term survival, thus it is of critical importance that diversity of TLRs are captured in the assurance populations. The loss of functional genetic diversity at TLR genes in the African penguin *ex situ* population may indicate a loss of adaptive potential; however the importance of polymorphism of TLRs in comparison to other protein coding immune genes has not yet been determined. Further studies on the level of expression as well as the functional relevance of these loci in African penguin would have to be conducted. In order to ensure that the *ex situ* population does not experience an additional bottleneck it is of critical importance that alterations in adaptive genes such as TLRs are thus captured to inform optimal management.

**Table 3:** Observed (H_o_), expected heterozygosity (H_e_) and unbiased heterozygosity (H_z_) estimates and polymorphism statistics at four TLR genotyped in African penguins.

Population	Description	N^1^	H_o_	H_e_	H_Z_
Two Oceans Aquarium	*ex situ*	7	0.071	0.066	0.071
uShaka Marine World	*ex situ*	6	0.208	0.264	0.288
NZG	*ex situ*	7	0.500	0.416	0.448
**Total**		**20**	**0.260**	**0.249**	**0.269**
Namibia (Lüderitz)	*in situ*	3	0.167	0.139	0.167
Dassen Island	*in situ*	3	0.083	0.069	0.083
Bird Island	*in situ*	3	0.167	0.125	0.150
Robben Island	*in situ*	3	0.333	0.375	0.450
Dyer Island	*in situ*	3	0.167	0.250	0.300
Boulders Beach	*in situ*	3	0.417	0.264	0.317
St Croix	*in situ*	3	0.250	0.167	0.200
**Total**		**21**	**0.226**	**0.198**	**0.238**

^1^ N: Number of samples

## Supporting Information

S1 Table PCR primers for five TLR genes in African penguin. (DOCX)

S2 Table Nucleotide sequence alignments of TLR genes of African penguin. (DOCX)
