# Supplementary material for: Diversity in the Toll-Like Receptor Genes of the African Penguin (Spheniscus demersus)
Source: PLoS One. 2016 Oct 19;11(10):e0163331. doi: 10.1371/journal.pone.0163331 (PMC5070850; doi:10.1371/journal.pone.0163331)
Supplement: S1 Table — (DOCX) [file pone.0163331.s001.docx]

**S 1 Table :** PCR primers for five TLR genes in African penguin

| **Genes** | **Fragment Length (bp)** | **F/R** | **Primer Sequence 5ʹ-3ʹ** | **T_a_** |
| --- | --- | --- | --- | --- |
| TLR1LA | 934 | F | GATGGAATGAGCACTTCAGA | 58°C |
|  |  | R | CTTCGTCTGCGTCACTG |  |
| TLR1LB | 891 | F | TCCAGGYTWCAAAATCTGACAC | 55°C |
|  |  | R | CGGCACRTCCARGTAGATG |  |
| TLR2 | 565 | F | AGGGACCTTCTGCACTCTG | 55°C |
|  |  | R | AGGAGACAAAAGCGTCGTAG |  |
| TLR5 | 1082 | F | GTAATCTTACCAGCTTCCAAGG | 55°C |
|  |  | R | GCTGGAGTTCATCTTCATC |  |
| TLR7 | 1042 | F | GTATCTKGGACARAACTGYTA | 55°C |
|  |  | R | TYGAAGAGATTGGCTTTCC |  |
