# Supplementary material for: Diversity in the Toll-Like Receptor Genes of the African Penguin (Spheniscus demersus)
Source: PLoS One. 2016 Oct 19;11(10):e0163331. doi: 10.1371/journal.pone.0163331 (PMC5070850; doi:10.1371/journal.pone.0163331)
Supplement: S2 Table — (DOCX) [file pone.0163331.s002.docx]

**TLR1LA**

>4156 TLR1A

TTACAATGGGCTATCATTTTGCAAATTTTCCTGTTTATTTGGGGTTCACCTGTGGAGCATTTGACTGTGAGAAATTTGACTTTTCGGGGACCACTGGGGGAGCTGACTGAGTATGAATTTCTAACCTTATTAAGCTCTCTGGAACAATTAATATCTTTGGGTGGCTCCATGAAAGTGCTAACTTTGGAGCACGTTCGTAATAAAGTTTTTTATTTCAACCAGGAGATTCTATACCGACAGTTTTCAGAGATGAATATTGCCAGTTTGACAATATATGATGCATATATGCCACACATGGTTTGCCCCAATAGAACAAGCTCATTTCAGTATATAAATTTTTCTCACAATGCCCTGACGGATGAATTGTTCCAGAATTGTGGTACTCTGACAGATCTGAAATTACTTATTTTGGAGAAGAATAAATTTGAGAGCCTTTCCAAGGTAAGCTTCATGACCAGCCGTATGAAATTGCTGCAATATTTGGACATGAGCAACAACTTGCTGAGTCACGATGCAGCTGATGTGCAATGCCGGTGGGCTGAGTCTCTGACAGAGTTGGACCTGTCCTCAAATCAGTTGACGGATTCCGTGTTTGAGTGCTTGCCAGTCAACATCAAAAAACTCAACCTACAAAACAATCAGATCACCAGTGTCGCCAAAGGGATGGCTGAGCTGAAATCCTTGGAAGAGCTGAACCTGGCATCGAACAGGCTGGCTGACCTGCCGGGGTGCAGTGGCTTTATGTCCCTGGAGTTCCTGAACGTAGAGATGAATTTGATCCTCACCCCATCTGCCGACTTCTTCCAGAGCTGCCTAAGGGTCAAGGAGCTACAAGCCGGGCACAACCCGTTCAAGTGTTCCTGTGAACTGCAAGACTTTATCCGTCTGGAGAGGCAGTCTGGGGTGAAGCTGTCTGACTGGCCGGAGGCATA

>4161 TLR1A

TTACAATGGGCTATCATTTTGCAAATTTTCCTGTTTATTTGGGGTTCACCTGTGGAGCATTTGACTGTGAGAAATTTGACTTTTCGGGGACCACTGGGGGAGCTGACTGAGTATGAATTTCTAACCTTATTAAGCTCTCTGGAACAATTAATATCTTTGGGTGGCTCCATGAAAGTGCTAACTTTGGAGCACGTTCGTAATAAAGTTTTTTATTTCAACCAGGAGATTCTATACCGACAGTTTTCAGAGATGAATATTGCCAGTTTGACAATATATGATGCATATATGCCACACATGGTTTGCCCCAATAGAACAAGCTCATTTCAGTATATAAATTTTTCTCACAATGCCCTGACGGATGAATTGTTCCAGAATTGTGGTACTCTGACAGATCTGAAATTACTTATTTTGGAGAAGAATAAATTTGAGAGCCTTTCCAAGGTAAGCTTCATGACCAGCCGTATGAAATTGCTGCAATATTTGGACATGAGCAACAACTTGCTGAGTCACGATGCAGCTGATGTGCAATGCCGGTGGGCTGAGTCTCTGACAGAGTTGGACCTGTCCTCAAATCAGTTGACGGATTCCGTGTTTGAGTGCTTGCCAGTCAACATCAAAAAACTCAACCTACAAAACAATCAGATCACCAGTGTCGCCAAAGGGATGGCTGAGCTGAAATCCTTGGAAGAGCTGAACCTGGCATCGAACAGGCTGGCTGACCTGCCGGGGTGCAGTGGCTTTATGTCCCTGGAGTTCCTGAACGTAGAGATGAATTTGATCCTCACCCCATCTGCCGACTTCTTCCAGAGCTGCCTAAGGGTCAAGGAGCTACAAGCCGGGCACAACCCGTTCAAGTGTTCCTGTGAACTGCAAGACTTTATCCGTCTGGAGAGGCAGTCTGGGGTGAAGCTGTCTGACTGGCCGGAGGCATA

>4177 TLR1A

TTACAATGGGCTATCATTTTGCAAATTTTCCTGTTTATTTGGGGTTCACCTGTGGAGCATTTGACTGTGAGAAATTTGACTTTTCGGGGACCACTGGGGGAGCTGACTGAGTATGAATTTCTAACCTTATTAAGCTCTCTGGAACAATTAATATCTTTGGGTGGCTCCATGAAAGTGCTAACTTTGGAGCACGTTCGTAATAAAGTTTTTTATTTCAACCAGGAGATTCTATACCGACAGTTTTCAGAGATGAATATTGCCAGTTTGACAATATATGATGCATATATGCCACACATGGTTTGCCCCAATAGAACAAGCTCATTTCAGTATATAAATTTTTCTCACAATGCCCTGACGGATGAATTGTTCCAGAATTGTGGTACTCTGACAGATCTGAAATTACTTATTTTGGAGAAGAATAAATTTGAGAGCCTTTCCAAGGTAAGCTTCATGACCAGCCGTATGAAATTGCTGCAATATTTGGACATGAGCAACAACTTGCTGAGTCACGATGCAGCTGATGTGCAATGCCGGTGGGCTGAGTCTCTGACAGAGTTGGACCTGTCCTCAAATCAGTTGACGGATTCCGTGTTTGAGTGCTTGCCAGTCAACATCAAAAAACTCAACCTACAAAACAATCAGATCACCAGTGTCGCCAAAGGGATGGCTGAGCTGAAATCCTTGGAAGAGCTGAACCTGGCATCGAACAGGCTGGCTGACCTGCCGGGGTGCAGTGGCTTTATGTCCCTGGAGTTCCTGAACGTAGAGATGAATTTGATCCTCACCCCATCTGCCGACTTCTTCCAGAGCTGCCTAAGGGTCAAGGAGCTACAAGCCGGGCACAACCCGTTCAAGTGTTCCTGTGAACTGCAAGACTTTATCCGTCTGGAGAGGCAGTCTGGGGTGAAGCTGTCTGACTGGCCGGAGGCATA

>711 TLR1A

TTACAATGGGCTATCATTTTGCAAATTTTCCTGTTTATTTGGGGTTCACCTGTGGAGCATTTGACTGTGAGAAATTTGACTTTTCGGGGACCACTGGGGGAGCTGACTGAGTATGAATTTCTAACCTTATTAAGCTCTCTGGAACAATTAATATCTTTGGGTGGCTCCATGAAAGTGCTAACTTTGGAGCACGTTCGTAATAAAGTTTTTTATTTCAACCAGGAGATTCTATACCGACAGTTTTCAGAGATGAATATTGCCAGTTTGACAATATATGATGCATATATGCCACACATGGTTTGCCCCAATAGAACAAGCTCATTTCAGTATATAAATTTTTCTCACAATGCCCTGACGGATGAATTGTTCCAGAATTGTGGTACTCTGACAGATCTGAAATTACTTATTTTGGAGAAGAATAAATTTGAGAGCCTTTCCAAGGTAAGCTTCATGACCAGCCGTATGAAATTGCTGCAATATTTGGACATGAGCAACAACTTGTTGAGTCACGATGCAGCTGATGTGCAATGCCGGTGGGCTGAGTCTCTGACAGAGTTGGACCTGTCCTCAAATCAGTTGACGGATTCCGTGTTTGAGTGCTTGCCAGTCAACATCAAAAAACTCAACCTACAAAACAATCAGATCACCAGTGTCGCCAAAGGGATGGCTGAGCTGAAATCCTTGGAAGAGCTGAACCTGGCATCGAACAGGCTGGCTGACCTGCCGGGGTGCAGTGGCTTTATGTCCCTGGAGTTCCTGAACGTAGAGATGAATTTGATCCTCACCCCATCTGCCGACTTCTTCCAGAGCTGCCTAAGGGTCAAGGAGCTACAAGCCGGGCACAACCCGTTCAAGTGTTCCTGTGAACTGCAAGACTTTATCCGTCTGGAGAGGCAGTCTGGGGTGAAGCTGTCTGACTGGCCGGAGGCATA

>713 TLR1A

TTACAATGGGCTATCATTTTGCAAATTTTCCTGTTTATTTGGGGTTCACCTGTGGAGCATTTGACTGTGAGAAATTTGACTTTTCGGGGACCACTGGGGGAGCTGACTGAGTATGAATTTCTAACCTTATTAAGCTCTCTGGAACAATTAATATCTTTGGGTGGCTCCATGAAAGTGCTAACTTTGGAGCACGTTCGTAATAAAGTTTTTTATTTCAACCAGGAGATTCTATACCGACAGTTTTCAGAGATGAATATTGCCAGTTTGACAATATATGATGCATATATGCCACACATGGTTTGCCCCAATAGAACAAGCTCATTTCAGTATATAAATTTTTCTCACAATGCCCTGACGGATGAATTGTTCCAGAATTGTGGTACTCTGACAGATCTGAAATTACTTATTTTGGAGAAGAATAAATTTGAGAGCCTTTCCAAGGTAAGCTTCATGACCAGCCGTATGAAATTGCTGCAATATTTGGACATGAGCAACAACTTGCTGAGTCACGATGCAGCTGATGTGCAATGCCGGTGGGCTGAGTCTCTGACAGAGTTGGACCTGTCCTCAAATCAGTTGACGGATTCCGTGTTTGAGTGCTTGCCAGTCAACATCAAAAAACTCAACCTACAAAACAATCAGATCACCAGTGTCGCCAAAGGGATGGCTGAGCTGAAATCCTTGGAAGAGCTGAACCTGGCATCGAACAGGCTGGCTGACCTGCCGGGGTGCAGTGGCTTTATGTCCCTGGAGTTCCTGAACGTAGAGATGAATTTGATCCTCACCCCATCTGCCGACTTCTTCCAGAGCTGCCTAAGGGTCAAGGAGCTACAAGCCGGGCACAACCCGTTCAAGTGTTCCTGTGAACTGCAAGACTTTATCCGTCTGGAGAGGCAGTCTGGGGTGAAGCTGTCTGACTGGCCGGAGGCATA

>715 TLR1A

TTACAATGGGCTATCATTTTGCAAATTTTCCTGTTTATTTGGGGTTCACCTGTGGAGCATTTGACTGTGAGAAATTTGACTTTTCGGGGACCACTGGGGGAGCTGACTGAGTATGAATTTCTAACCTTATTAAGCTCTCTGGAACAATTAATATCTTTGGGTGGCTCCATGAAAGTGCTAACTTTGGAGCACGTTCGTAATAAAGTTTTTTATTTCAACCAGGAGATTCTATACCGACAGTTTTCAGAGATGAATATTGCCAGTTTGACAATATATGATGCATATATGCCACACATGGTTTGCCCCAATAGAACAAGCTCATTTCAGTATATAAATTTTTCTCACAATGCCCTGACGGATGAATTGTTCCAGAATTGTGGTACTCTGACAGATCTGAAATTACTTATTTTGGAGAAGAATAAATTTGAGAGCCTTTCCAAGGTAAGCTTCATGACCAGCCGTATGAAATTGCTGCAATATTTGGACATGAGCAACAACTTGCTGAGTCACGATGCAGCTGATGTGCAATGCCGGTGGGCTGAGTCTCTGACAGAGTTGGACCTGTCCTCAAATCAGTTGACGGATTCCGTGTTTGAGTGCTTGCCAGTCAACATCAAAAAACTCAACCTACAAAACAATCAGATCACCAGTGTCGCCAAAGGGATGGCTGAGCTGAAATCCTTGGAAGAGCTGAACCTGGCATCGAACAGGCTGGCTGACCTGCCGGGGTGCAGTGGCTTTATGTCCCTGGAGTTCCTGAACGTAGAGATGAATTTGATCCTCACCCCATCTGCCGACTTCTTCCAGAGCTGCCTAAGGGTCAAGGAGCTACAAGCCGGGCACAACCCGTTCAAGTGTTCCTGTGAACTGCAAGACTTTATCCGTCTGGAGAGGCAGTCTGGGGTGAAGCTGTCTGACTGGCCGGAGGCATA

>822 TLR1A

TTACAATGGGCTATCATTTTGCAAATTTTCCTGTTTATTTGGGGTTCACCTGTGGAGCATTTGACTGTGAGAAATTTGACTTTTCGGGGACCACTGGGGGAGCTGACTGAGTATGAATTTCTAACCTTATTAAGCTCTCTGGAACAATTAATATCTTTGGGTGGCTCCATGAAAGTGCTAACTTTGGAGCACGTTCGTAATAAAGTTTTTTATTTCAACCAGGAGATTCTATACCGACAGTTTTCAGAGATGAATATTGCCAGTTTGACAATATATGATGCATATATGCCACACATGGTTTGCCCCAATAGAACAAGCTCATTTCAGTATATAAATTTTTCTCACAATGCCCTGACGGATGAATTGTTCCAGAATTGTGGTACTCTGACAGATCTGAAATTACTTATTTTGGAGAAGAATAAATTTGAGAGCCTTTCCAAGGTAAGCTTCATGACCAGCCGTATGAAATTGCTGCAATATTTGGACATGAGCAACAACTTGCTGAGTCACGATGCAGCTGATGTGCAATGCCGGTGGGCTGAGTCTCTGACAGAGTTGGACCTGTCCTCAAATCAGTTGACGGATTCCGTGTTTGAGTGCTTGCCAGTCAACATCAAAAAACTCAACCTACAAAACAATCAGATCACCAGTGTCGCCAAAGGGATGGCTGAGCTGAAATCCTTGGAAGAGCTGAACTGGGCATCGAACAGGCTGGCTGACCTGCCGGGGTGCAGTGGCTTTATGTCCCTGGAGTTCCTGAACGTAGAGATGAATTTGATCCTCACCCCATCTGCCGACTTCTTCCAGAGCTGCCTAAGGGTCAAGGAGCTACAAGCCGGGCACAACCCGTTCAAGTGTTCCTGTGAACTGCAAGACTTTATCCGTCTGGAGAGGCAGTCTGGGGTGAAGCTGTCTGACTGGCCGGAGGCATA

>823 TLR1A

TTACAATGGGCTATCATTTTGCAAATTTTCCTGTTTATTTGGGGTTCACCTGTGGAGCATTTGACTGTGAGAAATTTGACTTTTCGGGGACCACTGGGGGAGCTGACTGAGTATGAATTTCTAACCTTATTAAGCTCTCTGGAACAATTAATATCTTTGGGTGGCTCCATGAAAGTGCTAACTTTGGAGCACGTTCGTAATAAAGTTTTTTATTTCAACCAGGAGATTCTATACCGACAGTTTTCAGAGATGAATATTGCCAGTTTGACAATATATGATGCATATATGCCACACATGGTTTGCCCCAATAGAACAAGCTCATTTCAGTATATAAATTTTTCTCACAATGCCCTGACGGATGAATTGTTCCAGAATTGTGGTACTCTGACAGATCTGAAATTACTTATTTTGGAGAAGAATAAATTTGAGAGCCTTTCCAAGGTAAGCTTCATGACCAGCCGTATGAAATTGCTGCAATATTTGGACATGAGCAACAACTTGCTGAGTCACGATGCAGCTGATGTGCAATGCCGGTGGGCTGAGTCTCTGACAGAGTTGGACCTGTCCTCAAATCAGTTGACGGATTCCGTGTTTGAGTGCTTGCCAGTCAACATCAAAAAACTCAACCTACAAAACAATCAGATCACCAGTGTCGCCAAAGGGATGGCTGAGCTGAAATCCTTGGAAGAGCTGAACCTGGCATCGAACAGGCTGGCTGACCTGCCGGGGTGCAGTGGCTTTATGTCCCTGGAGTTCCTGAACGTAGAGATGAATTTGATCCTCACCCCATCTGCCGACTTCTTCCAGAGCTGCCTAAGGGTCAAGGAGCTACAAGCCGGGCACAACCCGTTCAAGTGTTCCTGTGAACTGCAAGACTTTATCCGTCTGGAGAGGCAGTCTGGGGTGAAGCTGTCTGACTGGCCGGAGGCATA

>826 TLR1A

TTACAATGGGCTATCATTTTGCAAATTTTCCTGTTTATTTGGGGTTCACCTGTGGAGCATTTGACTGTGAGAAATTTGACTTTTCGGGGACCACTGGGGGAGCTGACTGAGTATGAATTTCTAACCTTATTAAGCTCTCTGGAACAATTAATATCTTTGGGTGGCTCCATGAAAGTGCTAACTTTGGAGCACGTTCGTAATAAAGTTTTTTATTTCAACCAGGAGATTCTATACCGACAGTTTTCAGAGATGAATATTGCCAGTTTGACAATATATGATGCATATATGCCACACATGGTTTGCCCCAATAGAACAAGCTCATTTCAGTATATAAATTTTTCTCACAATGCCCTGACGGATGAATTGTTCCAGAATTGTGGTACTCTGACAGATCTGAAATTACTTATTTTGGAGAAGAATAAATTTGAGAGCCTTTCCAAGGTAAGCTTCATGACCAGCCGTATGAAATTGCTGCAGTATTTGGACATGAGCAACAACTTGTTGAGTCACGATGCAGCTGATGTGCAATGCCGGTGGGCTGAGTCTCTGACAGAGTTGGACCTGTCCTCAAATCAGTTGACGGATTCCGTGTTTGAGTGCTTGCCAGTCAACATCAAAAAACTCAACCTACAAAACAATCAGATCACCAGTGTCGCCAAAGGGATGGCTGAGCTGAAATCCTTGGAAGAGCTGAACCTGGCATCGAACAGGCTGGCTGACCTGCCGGGGTGCAGTGGCTTTATGTCCCTGGAGTTCCTGAACGTAGAGATGAATTTGATCCTCACCCCATCTGCCGACTTCTTCCAGAGCTGCCTAAGGGTCAAGGAGCTACAAGCCGGGCACAACCCGTTCAAGTGTTCCTGTGAACTGCAAGACTTTATCCGTCTGGAGAGGCAGTCTGGGGTGAAGCTGTCTGACTGGCCGGAGGCATA

>103 TLR1A

TTACAAAGGGCTATCATTTTGCAAATTTTCCTGTTTATTTGGGGTTCACCTGTGGAGCATTTGACTGTGAGAAATTTGACTTTTCGGGGACCACTGGGGGAGCTGACTGAGTATGAATTTCTAACCTTATTAAGCTCTCTGGAACAATTAATATCTTTGGGTGGCTCCATGAAAGTGCTAACTTTGGAGCACGTTCGTAATAAAGTTTTTTATTTCAACCAGGAGATTCTATACCGACAGTTTTCAGAGATGAATATTGCCAGTTTGACAATATATGATGCATATATGCCACACATGGTTTGCCCCAATAGAACAAGCTCATTTCAGTATATAAATTTTTCTCACAATGCCCTGACGGATGAATTGTTCCAGAATTGTGGTACTCTGACAGATCTGAAATTACTTATTTTGGAGAAGAATAAATTTGAGAGCCTTTCCAAGGTAAGCTTCATGACCAGCCGTATGAAATTGCTGCAATATTTGGACATGAGCAACAACTTGCTGAGTCACGATGCAGCTGATGTGCAATGCCGGTGGGCTGAGTCTCTGACAGAGTTGGACCTGTCCTCAAATCAGTTGACGGATTCCGTGTTTGAGTGCTTGCCAGTCAACATCAAAAAACTCAACCTACAAAACAATCAGATCACCAGTGTCGCCAAAGGGATGGCTGAGCTGAAATCCTTGGAAGAGCTGAACCTGGCATCGAACAGGCTGGCTGACCTGCCGGGGTGCAGTGGCTTTATGTCCCTGGAGTTCCTGAACGTAGAGATGAATTTGATCCTCACCCCATCTGCCGACTTCTTCCAGAGCTGCCTAAGGGTCAAGGAGCTACAAGCCGGGCACAACCCGTTCAAGTGTTCCTGTGAACTGCAAGACTTTATCCGTCTGGAGAGGCAGTCTGGGGTGAAGCTGTCTGACTGGCCGGAGGCATA

>1011 TLR1A

TTACAATGGGCTATCATTTTGCAAATTTTCCTGTTTATTTGGGGTTCACCTGTGGAGCATTTGACTGTGAGAAATTTGACTTTTCGGGGACCACTGGGGGAGCTGACTGAGTATGAATTTCTAACCTTATTAAGCTCTCTGGAACAATTAATATCTTTGGGTGGCTCCGTGAAAGTGCTAACTTTGGAGCACGTTCGTAATAAAGTTTTTTATTTCAACCAGGAGATTCTATACCGACAGTTTTCAGAGATGAATATTGCCAGTTTGACAATATATGATGCATATATGCCACACATGGTTTGCCCCAATAGAACAAGCTCATTTCAGTATATAAaTTTTTCTCACAATGCCCTGACGGATGAATTGTTCCAGAATTGTGGTACTCTGACAGATCTGAAATTACTTATTTTGGAGAAGAATAAATTTGAGAGCCTTTCCAAGGTAAGCTTCATGACCAGCCGTATGAAATTGCTGCAATATTTGGACATGAGCAACAACTTGCTGAGTCACGATGCAGCTGATGTGCAATGCCGGTGGGCTGAGTCTCTGACAGAGTTGGACCTGTCCTCAAATCAGTTGACGGATTCCGTGTTTGAGTGCTTGCCAGTCAACATCAAAAAACTCAACCTACAAAACAATCAGATCACCAGTGTCGCCAAAGGGATGGCTGAGCTGAAATCCTTGGAAGAGCTGAACCTGGCATCGAACAGGCTGGCTGACCTGCCGGGGTGCAGTGGCTTTATGTCCCTGGAGTTCCTGAACGTAGAGATGAATTTGATCCTCACCCCATCTGCCGACTTCTTCGAGAGCTGCCTAAGGGTCAAGGAGCTACAAGCCGGGCACAACCCGTTCAAGTGTTCCTGTGAACTGCAAGACTTTATCCGTCTGGAGAGGCAGTCTGGGGTGAAGCTGTCTGACTGGCCGGAGGCATA

>1017 TLR1A

TTACAATGGGCTATCATTTTGCAAATTTTCCTGTTTATTTGGGGTTCACCTGTGGAGCATTTGACTGTGAGAAATTTGACTTTTCGGGGACCACTGGGGGAGCTGACTGAGTATGAATTTCTAACCTTATTAAGCTCTCTGGAACAATTAATATCTTTGGGTGGCTCCATGAAAGTGCTAACTTTGGAGCACGTTCGTAATAAAGTTTTTTATTTCAACCAGGAGATTCTATACCGACAGTTTTCAGAGATGAATATTGCCAGTTTGACAATATATGATGCATATATGCCACACATGGTTTGCCCCAATAGAACAAGCTCATTTCAGTATATAAATTTTTCTCACAATGCCCTGACGGATGAATTGTTCCAGAATTGTGGTACTCTGACAGATCTGAAATTACTTATTTTGGAGAAGAATAAATTTGAGAGCCTTTCCAAGGTAAGCTTCATGACCAGCCGTATGAAATTGCTGCAATATTTGGACATGAGCAACAACTTGTTGAGTCACGATGCAGCTGATGTGCAATGCCGGTGGGCTGAGTCTCTGACAGAGTTGGACCTGTCCTCAAATCAGTTGACGGATTCCGTGTTTGAGTGCTTGCCAGTCAACATCAAAAAACTCAACCTACAAAACAATCAGATCACCAGTGTCGCCAAAGGGATGGCTGAGCTGAAATCCTTGGAAGAGCTGAACTGGGCATCGAACAGGCTGGCTGACCTGCCGGGGTGCAGTGGCTTTATGTCCCTGGAGTTCCTGAACGTAGAGATGAATTTGATCCTCACCCCATCTGCCGACTTCTTCCAGAGCTGCCTAAGGGTCAAGGAGCTACAAGCCGGGCACAACCCGTTCAAGTGTTCCTGTGAACTGCAAGACTTTATCCGTCTGGAGAGGCAGTCTGGGGTGAAGCTGTCTGACTGGCCGGAGGCATA

>1210 TLR1A

TTACAATGGGCTATCATTTTGCAAATTTTCCTGTTTATTTGGGGTTCACCTGTGGAGCATTTGACTGTGAGAAATTTGACTTTTCGGGGACCACTGGGGGAGCTGACTGAGTATGAATTTCTAACCTTATTAAGCTCTCTGGAACAATTAATATCTTTGGGTGGCTCCATGAAAGTGCTAACTTTGGAGCACGTTCGTAATAAAGTTTTTTATTTCAACCAGGAGATTCTATACCGACAGTTTTCAGAGATGAATATTGCCAGTTTGACAATATATGATGCATATATGCCACACATGGTTTGCCCCAATAGAACAAGCTCATTTCAGTATATAAATTTTTCTCACAATGCCCTGACGGATGAATTGTTCCAGAATTGTGGTACTCTGACAGATCTGAAATTACTTATTTTGGAGAAGAATAAATTTGAGAGCCTTTCCAAGGTAAGCTTCATGACCAGCCGTATGAAATTGCTGCAATATTTGGACATGAGCAACAACTTGCTGAGTCACGATGCAGCTGATGTGCAATGCCGGTGGGCTGAGTCTCTGACAGAGTTGGACCTGTCCTCAAATCAGTTGACGGATTCCGTGTTTGAGTGCTTGCCAGTCAACATCAAAAAACTCAACCTACAAAACAATCAGATCACCAGTGTCGCCAAAGGGATGGCTGAGCTGAAATCCTTGGAAGAGCTGAACCTGGCATCGAACAGGCTGGCTGACCTGCCGGGGTGCAGTGGCTTTATGTCCCTGGAGTTCCTGAACGTAGAGATGAATTTGATCCTCACCCCATCTGCCGACTTCTTCCAGAGCTGCCTAAGGGTCAAGGAGCTACAAGCCGGGCACAACCCGTTCAAGTGTTCCTGTGAACTGCAAGACTTTATCCGTCTGGAGAGGCAGTCTGGGGTGAAGCTGTCTGACTGGCCGGAGGCATA

>1211 TLR1A

TTACAATGGGCTATCATTTTGCAAATTTTCCTGTTTATTTGGGGTTCACCTGTGGAGCATTTGACTGTGAGAAATTTGACTTTTCGGGGACCACTGGGGGAGCTGACTGAGTATGAATTTCTAACCTTATTAAGCTCTCTGGAACAATTAATATCTTTGGGTGGCTCCATGAAAGTGCTAACTTTGGAGCACGTTCGTAATAAAGTTTTTTATTTCAACCAGGAGATTCTATACCGACAGTTTTCAGAGATGAATATTGCCAGTTTGACAATATATGATGCATATATGCCACACATGGTTTGCCCCAATAGAACAAGCTCATTTCAGTATATAAATTTTTCTCACAATGCCCTGACGGATGAATTGTTCCAGAATTGTGGTACTCTGACAGATCTGAAATTACTTATTTTGGAGAAGAATAAATTTGAGAGCCTTTCCAAGGTAAGCTTCATGACCAGCCGTATGAAATTGCTGCAATATTTGGACATGAGCAACAACTTGCTGAGTCACGATGCAGCTGATGTGCAATGCCGGTGGGCTGAGTCTCTGACAGAGTTGGACCTGTCCTCAAATCAGTTGACGGATTCCGTGTTTGAGTGCTTGCCAGTCAACATCAAAAAACTCAACCTACAAAACAATCAGATCACCAGTGTCGCCAAAGGGATGGCTGAGCTGAAATCCTTGGAAGAGCTGAACCTGGCATCGAACAGGCTGGCTGACCTGCCGGGGTGCAGTGGCTTTATGTCCCTGGAGTTCCTGAACGTAGAGATGAATTTGATCCTCACCCCATCTGCCGACTTCTTCCAGAGCTGCCTAAGGGTCAAGGAGCTACAAGCCGGGCACAACCCGTTCAAGTGTTCCTGTGAACTGCAAGACTTTATCCGTCTGGAGAGGCAGTCTGGGGTGAAGCTGTCTGACTGGCCGGAGGCATA

>1214 TLR1A

TTACAATGGGCTATCATTTTGCAAATTTTCCTGTTTATTTGGGGTTCACCTGTGGAGCATTTGACTGTGAGAAATTTGACTTTTCGGGGACCACTGGGGGAGCTGACTGAGTATGAATTTCTAACCTTATTAAGCTCTCTGGAACAATTAATATCTTTGGGTGGCTCCATGAAAGTGCTAACTTTGGAGCACGTTCGTAATAAAGTTTTTTATTTCAACCAGGAGATTCTATACCGACAGTTTTCAGAGATGAATATTGCCAGTTTGACAATATATGATGCATATATGCCACACATGGTTTGCCCCAATAGAACAAGCTCATTTCAGTATATAAATTTTTCTCACAATGCCCTGACGGATGAATTGTTCCAGAATTGTGGTACTCTGACAGATCTGAAATTACTTATTTTGGAGAAGAATAAATTTGAGAGCCTTTCCAAGGTAAGCTTCATGACCAGCCGTATGAAATTGCTGCAATATTTGGACATGAGCAACAACTTGCTGAGTCACGATGCAGCTGATGTGCAATGCCGGTGGGCTGAGTCTCTGACAGAGTTGGACCTGTCCTCAAATCAGTTGACGGATTCCGTGTTTGAGTGCTTGCCAGTCAACATCAAAAAACTCAACCTACAAAACAATCAGATCACCAGTGTCGCCAAAGGGATGGCTGAGCTGAAATCCTTGGAAGAGCTGAACTGGGCATCGAACAGGCTGGCTGACCTGCCGGGGTGCAGTGGCTTTATGTCCCTGGAGTTCCTGAACGTAGAGATGAATTTGATCCTCACCCCATCTGCCGACTTCTTCCAGAGCTGCCTAAGGGTCAAGGAGCTACAAGCCGGGCACAACCCGTTCAAGTGTTCCTGTGAACTGCAAGACTTTATCCGTCTGGAGAGGCAGTCTGGGGTGAAGCTGTCTGACTGGCCGGAGGCATA

>169 TLR1A

TTACAATGGGCTATCATTTTGCAAATTTTCCTGTTTATTTGGGGTTCACCTGTGGAGCATTTGACTGTGAGAAATTTGACTTTTCGGGGACCACTGGGGGAGCTGACTGAGTATGAATTTCTAACCTTATTAAGCTCTCTGGAACAATTAATATCTTTGGGTGGCTCCATGAAAGTGCTAACTTTGGAGCACGTTCGTAATAAAGTTTTTTATTTCAACCAGGAGATTCTATACCGACAGTTTTCAGAGATGAATATTGCCAGTTTGACAATATATGATGCATATATGCCACACATGGTTTGCCCCAATAGAACAAGCTCATTTCAGTATATAAATTTTTCTCACAATGCCCTGACGGATGAATTGTTCCAGAATTGTGGTACTCTGACAGATCTGAAATTACTTATTTTGGAGAAGAATAAATTTGAGAGCCTTTCCAAGGTAAGCTTCATGACCAGCCGTATGAAATTGCTGCAATATTTGGACATGAGCAACAACTTGCTGAGTCACGATGCAGCTGATGTGCAATGCCGGTGGGCTGAGTCTCTGACAGAGTTGGACCTGTCCTCAAATCAGTTGACGGATTCCGTGTTTGAGTGCTTGCCAGTCAACATCAAAAAACTCAACCTACAAAACAATCAGATCACCAGTGTCGCCAAAGGGATGGCTGAGCTGAAATCCTTGGAAGAGCTGAACCTGGCATCGAACAGGCTGGCTGACCTGCCGGGGTGCAGTGGCTTTATGTCCCTGGAGTTCCTGAACGTAGAGATGAATTTGATCCTCACCCCATCTGCCGACTTCTTCCAGAGCTGCCTAAGGGTCAAGGAGCTACAAGCCGGGCACAACCCGTTCAAGTGTTCCTGTGAACTGCAAGACTTTATCCGTCTGGAGAGGCAGTCTGGGGTGAAGCTGTCTGACTGGCCGGAGGCATA

>1610 TLR1A

TTACAATGGGCTATCATTTTGCAAATTTTCCTGTTTATTTGGGGTTCACCTGTGGAGCATTTGACTGTGAGAAATTTGACTTTTCGGGGACCACTGGGGGAGCTGACTGAGTATGAATTTCTAACCTTATTAAGCTCTCTGGAACAATTAATATCTTTGGGTGGCTCCATGAAAGTGCTAACTTTGGAGCACGTTCGTAATAAAGTTTTTTATTTCAACCAGGAGATTCTATACCGACAGTTTTCAGAGATGAATATTGCCAGTTTGACAATATATGATGCATATATGCCACACATGGTTTGCCCCAATAGAACAAGCTCATTTCAGTATATAAATTTTTCTCACAATGCCCTGACGGATGAATTGTTCCAGAATTGTGGTACTCTGACAGATCTGAAATTACTTATTTTGGAGAAGAATAAATTTGAGAGCCTTTCCAAGGTAAGCTTCATGACCAGCCGTATGAAATTGCTGCAATATTTGGACATGAGCAACAACTTGCTGAGTCACGATGCAGCTGATGTGCAATGCCGGTGGGCTGAGTCTCTGACAGAGTTGGACCTGTCCTCAAATCAGTTGACGGATTCCGTGTTTGAGTGCTTGCCAGTCAACATCAAAAAACTCAACCTACAAAACAATCAGATCACCAGTGTCGCCAAAGGGATGGCTGAGCTGAAATCCTTGGAAGAGCTGAACTGGGCATCGAACAGGCTGGCTGACCTGCCGGGGTGCAGTGGCTTTATGTCCCTGGAGTTCCTGAACGTAGAGATGAATTTGATCCTCACCCCATCTGCCGACTTCTTCCAGAGCTGCCTAAGGGTCAAGGAGCTACAAGCCGGGCACAACCCGTTCAAGTGTTCCTGTGAACTGCAAGACTTTATCCGTCTGGAGAGGCAGTCTGGGGTGAAGCTGTCTGACTGGCCGGAGGCATA

>1616 TLR1A

TTACAATGGGCTATCATTTTGCAAATTTTCCTGTTTATTTGGGGTTCACCTGTGGAGCATTTGACTGTGAGAAATTTGACTTTTCGGGGACCACTGGGGGAGCTGACTGAGTATGAATTTCTAACCTTATTAAGCTCTCTGGAACAATTAATATCTTTGGGTGGCTCCATGAAAGTGCTAACTTTGGAGCACGTTCGTAATAAAGTTTTTTATTTCAACCAGGAGATTCTATACCGACAGTTTTCAGAGATGAATATTGCCAGTTTGACAATATATGATGCATATATGCCACACATGGTTTGCCCCAATAGAACAAGCTCATTTCAGTATATAAATTTTTCTCACAATGCCCTGACGGATGAATTGTTCCAGAATTGTGGTACTCTGACAGATCTGAAATTACTTATTTTGGAGAAGAATAAATTTGAGAGCCTTTCCAAGGTAAGCTTCATGACCAGCCGTATGAAATTGCTGCAATATTTGGACATGAGCAACAACTTGCTGAGTCACGATGCAGCTGATGTGCAATGCCGGTGGGCTGAGTCTCTGACAGAGTTGGACCTGTCCTCAAATCAGTTGACGGATTCCGTGTTTGAGTGCTTGCCAGTCAACATCAAAAAACTCAACCTACAAAACAATCAGATCACCAGTGTCGCCAAAGGGATGGCTGAGCTGAAATCCTTGGAAGAGCTGAACCTGGCATCGAACAGGCTGGCTGACCTGCCGGGGTGCAGTGGCTTTATGTCCCTGGAGTTCCTGAACGTAGAGATGAATTTGATCCTCACCCCATCTGCCGACTTCTTCCAGAGCTGCCTAAGGGTCAAGGAGCTACAAGCCGGGCACAACCCGTTCAAGTGTTCCTGTGAACTGCAAGACTTTATCCGTCTGGAGAGGCAGTCTGGGGTGAAGCTGTCTGACTGGCCGGAGGCATA

>175 TLR1A

TTACAATGGGCTATCATTTTGCAAATTTTCCTGTTTATTTGGGGTTCACCTGTGGAGCATTTGACTGTGAGAAATTTGACTTTTCGGGGACCACTGGGGGAGCTGACTGAGTATGAATTTCTAACCTTATTAAGCTCTCTGGAACAATTAATATCTTTGGGTGGCTCCATGAAAGTGCTAACTTTGGAGCACGTTCGTAATAAAGTTTTTTATTTCAACCAGGAGATTCTATACCGACAGTTTTCAGAGATGAATATTGCCAGTTTGACAATATATGATGCATATATGCCACACATGGTTTGCCCCAATAGAACAAGCTCATTTCAGTATATAAATTTTTCTCACAATGCCCTGACGGATGAATTGTTCCAGAATTGTGGTACTCTGACAGATCTGAAATTACTTATTTTGGAGAAGAATAAATTTGAGAGCCTTTCCAAGGTAAGCTTCATGACCAGCCGTATGAAATTGCTGCAATATTTGGACATGAGCAACAACTTGCTGAGTCACGATGCAGCTGATGTGCAATGCCGGTGGGCTGAGTCTCTGACAGAGTTGGACCTGTCCTCAAATCAGTTGACGGATTCCGTGTTTGAGTGCTTGCCAGTCAACATCAAAAAACTCAACCTACAAAACAATCAGATCACCAGTGTCGCCAAAGGGATGGCTGAGCTGAAATCCTTGGAAGAGCTGAACTGGGCATCGAACAGGCTGGCTGACCTGCCGGGGTGCAGTGGCTTTATGTCCCTGGAGTTCCTGAACGTAGAGATGAATTTGATCCTCACCCCATCTGCCGACTTCTTCCAGAGCTGCCTAAGGGTCAAGGAGCTACAAGCCGGGCACAACCCGTTCAAGTGTTCCTGTGAACTGCAAGACTTTATCCGTCTGGAGAGGCAGTCTGGGGTGAAGCTGTCTGACTGGCCGGAGGCATA

>176 TLR1A

TTACAATGGGCTATCATTTTGCAAATTTTCCTGTTTATTTGGGGTTCACCTGTGGAGCATTTGACTGTGAGAAATTTGACTTTTCGGGGACCACTGGGGGAGCTGACTGAGTATGAATTTCTAACCTTATTAAGCTCTCTGGAACAATTAATATCTTTGGGTGGCTCCATGAAAGTGCTAACTTTGGAGCACGTTCGTAATAAAGTTTTTTATTTCAACCAGGAGATTCTATACCGACAGTTTTCAGAGATGAATATTGCCAGTTTGACAATATATGATGCATATATGCCACACATGGTTTGCCCCAATAGAACAAGCTCATTTCAGTATATAAATTTTTCTCACAATGCCCTGACGGATGAATTGTTCCAGAATTGTGGTACTCTGACAGATCTGAAATTACTTATTTTGGAGAAGAATAAATTTGAGAGCCTTTCCAAGGTAAGCTTCATGACCAGCCGTATGAAATTGCTGCAATATTTGGACATGAGCAACAACTTGCTGAGTCACGATGCAGCTGATGTGCAATGCCGGTGGGCTGAGTCTCTGACAGAGTTGGACCTGTCCTCAAATCAGTTGACGGATTCCGTGTTTGAGTGCTTGCCAGTCAACATCAAAAAACTCAACCTACAAAACAATCAGATCACCAGTGTCGCCAAAGGGATGGCTGAGCTGAAATCCTTGGAAGAGCTGAACTGGGCATCGAACAGGCTGGCTGACCGGCCGGGGTGCAGTGGCTTTATGTCCCTGGAGTTCCTGAACGTAGAGAGGAATTTGATCCTCACCCCATCTGCCGACTTCTTCCAGAACTGCCTAAGGGTCAAGGAGCTACAAGCCGGGCACAACCCGTTCAAGTGTTCCTGTGAACTGCAAGACTTTATCCGTCTGGAGAGGCAGTCCGGGGTGAAGCTGTCTGACTGGCCGGAGGCATA

>1714 TLR1A

TTACAATGGGCTATCATTTTGCAAATTTTCCTGTTTATTTGGGGTTCACCTGTGGAGCATTTGACTGTGAGAAATTTGACTTTTCGGGGACCACTGGGGGAGCTGACTGAGTATGAATTTCTAACCTTATTAAGCTCTCTGGAACAATTAATATCTTTGGGTGGCTCCATGAAAGTGCTAACTTTGGAGCACGTTCGTAATAAAGTTTTTTATTTCAACCAGGAGATTCTATACCGACAGTTTTCAGAGATGAATATTGCCAGTTTGACAATATATGATGCATATATGCCACACATGGTTTGCCCCAATAGAACAAGCTCATTTCAGTATATAAATTTTTCTCACAATGCCCTGACGGATGAATTGTTCCAGAATTGTGGTACTCTGACAGATCTGAAATTACTTATTTTGGAGAAGAATAAATTTGAGAGCCTTTCCAAGGTAAGCTTCATGACCAGCCGTATGAAATTGCTGCAATATTTGGACATGAGCAACAACTTGCTGAGTCACGATGCAGCTGATGTGCAATGCCGGTGGGCTGAGTCTCTGACAGAGTTGGACCTGTCCTCAAATCAGTTGACGGATTCCGTGTTTGAGTGCTTGCCAGTCAACATCAAAAAACTCAACCTACAAAACAATCAGATCACCAGTGTCGCCAAAGGGATGGCTGAGCTGAAATCCTTGGAAGAGCTGAACCGGGCATCGAACAGGCTGGCTGACCGGCCGGGGTGCAGTGGCTTTATGTCCCTGGAGTTCCTGAACGTAGAGATGAATTTGATCCTCACCCCATCTGCCGACTTCTTCCAGAGCTGCCTAAGGGTCAAGGAGCTACAAGCCGGGCACAACCCGTTCAAGTGTTCCTGTGAACTGCAAGACTTTATCCGTCTGGAGAGGCAGTCTGGGGTGAAGCTGTCTGACTGGCCGGAGGCATA

>30583 TLR1A

TTACAATGGGCTATCATTTTGCAAATTTTCCTGTTTATTTGGGGTTCACCTGTGGAGCATTTGACTGTGAGAAATTTGACTTTTCGGGGACCACTGGGGGAGCTGACTGAGTATGAATTTCTAACCTTATTAAGCTCTCTGGAACAATTAATATCTTTGGGTGGCTCCATGAAAGTGCTAACTTTGGAGCACGTTCGTAATAAAGTTTTTTATTTCAACCAGGAGATTCTATACCGACAGTTTTCAGAGATGAATATTGCCAGTTTGACAATATATGATGCATATATGCCACACATGGTTTGCCCCAATAGAACAAGCTCATTTCAGTATATAAATTTTTCTCACAATGCCCTGACGGATGAATTGTTCCAGAATTGTGGTACTCTGACAGATCTGAAATTACTTATTTTGGAGAAGAATAAATTTGAGAGCCTTTCCAAGGTAAGCTTCATGACCAGCCGTATGAAATTGCTGCAATATTTGGACATGAGCAACAACTTGCTGAGTCACGATGCAGCTGATGTGCAATGCCGGTGGGCTGAGTCTCTGACAGAGTTGGACCTGTCCTCAAATCAGTTGACGGATTCCGTGTTTGAGTGCTTGCCAGTCAACATCAAAAAACTCAACCTACAAAACAATCAGATCACCAGTGTCGCCAAAGGGATGGCTGAGCTGAAATCCTTGGAAGAGCTGAACCTGGCATCGAACAGGCTGGCTGACCTGCCGGGGTGCAGTGGCTTTATGTCCCTGGAGTTCCTGAACGTAGAGATGAATTTGATCCTCACCCCATCTGCCGACTTCTTCCAGAGCTGCCTAAGGGTCAAGGAGCTACAAGCCGGGCACAACCCGTTCAAGTGTTCCTGTGAACTGCAAGACTTTATCCGTCTGGAGAGGCAGTCTGGGGTGAAGCTGTCTGACTGGCCGGAGGCATA

>30585 TLR1A

TTACAATGGGCTATCATTTTGCAAATTTTCCTGTTTATTTGGGGTTCACCTGTGGAGCATTTGACTGTGAGAAATTTGACTTTTCGGGGACCACTGGGGGAGCTGACTGAGTATGAATTTCTAACCTTATTAAGCTCTCTGGAACAATTAATATCTTTGGGTGGCTCCATGAAAGTGCTAACTTTGGAGCACGTTCGTAATAAAGTTTTTTATTTCAACCAGGAGATTCTATACCGACAGTTTTCAGAGATGAATATTGCCAGTTTGACAATATATGATGCATATATGCCACACATGGTTTGCCCCAATAGAACAAGCTCATTTCAGTATATAAATTTTTCTCACAATGCCCTGACGGATGAATTGTTCCAGAATTGTGGTACTCTGACAGATCTGAAATTACTTATTTTGGAGAAGAATAAATTTGAGAGCCTTTCCAAGGTAAGCTTCATGACCAGCCGTATGAAATTGCTGCAATATTTGGACATGAGCAACAACTTGCTGAGTCACGATGCAGCTGATGTGCAATGCCGGTGGGCTGAGTCTCTGACAGAGTTGGACCTGTCCTCAAATCAGTTGACGGATTCCGTGTTTGAGTGCTTGCCAGTCAACATCAAAAAACTCAACCTACAAAACAATCAGATCACCAGTGTCGCCAAAGGGATGGCTGAGCTGAAATCCTTGGAAGAGCTGAACCTGGCATCGAACAGGCTGGCTGACCTGCCGGGGTGCAGTGGCTTTATGTCCCTGGAGTTCCTGAACGTAGAGATGAATTTGATCCTCACCCCATCTGCCGACTTCTTCCAGAGCTGCCTAAGGGTCAAGGAGCTACAAGCCGGGCACAACCCGTTCAAGTGTTCCTGTGAACTGCAAGACTTTATCCGTCTGGAGAGGCAGTCTGGGGTGATGCTGTCTGACTGGCCGGAGGCATA

>30586 TLR1A

TTACAATGGGCTATCATTTTGCAAATTTTCCTGTTTATTTGGGGTTCACCTGTGGAGCATTTGACTGTGAGAAATTTGACTTTTCGGGGACCACTGGGGGAGCTGACTGAGTATGAATTTCTAACCTTATTAAGCTCTCTGGAACAATTAATATCTTTGGGTGGCTCCATGAAAGTGCTAACTTTGGAGCACGTTCGTAATAAAGTTTTTTATTTCAACCAGGAGATTCTATACCGACAGTTTTCAGAGATGAATATTGCCAGTTTGACAATATATGATGCATATATGCCACACATGGTTTGCCCCAATAGAACAAGCTCATTTCAGTATATAAATTTTTCTCACAATGCCCTGACGGATGAATTGTTCCAGAATTGTGGTACTCTGACAGATCTGAAATTACTTATTTTGGAGAAGAATAAATTTGAGAGCCTTTCCAAGGTAAGCTTCATGACCAGCCGTATGAAATTGCTGCAATATTTGGACATGAGCAACAACTTGCTGAGTCACGATGCAGCTGATGTGCAATGCCGGTGGGCTGAGTCTCTGACAGAGTTGGACCTGTCCTCAAATCAGTTGACGGATTCCGTGTTTGAGTGCTTGCCAGTCAACATCAAAAAACTCAACCTACAAAACAATCAGATCACCAGTGTCGCCAAAGGGATGGCTGAGCTGAAATCCTTGGAAGAGCTGAACCTGGCATCGAACAGGCTGGCTGACCTGCCGGGGTGCAGTGGCTTTATGTCCCTGGAGTTCCTGAACGTAGAGATGAATTTGATCCTCACCCCATCTGCCGACTTCTTCCAGAGCTGCCTAAGGGTCAAGGAGCTACAAGCCGGGCACAACCCGTTCAAGTGTTCCTGTGAACTGCAAGACTTTATCCGTCTGGAGAGGCAGTCTGGGGTGAAGCTGTCTGACTGGCCGGAGGCATA

>30588 TLR1A

TTACAATGGGCTATCATTTTGCAAATTTTCCTGTTTATTTGGGGTTCACCTGTGGAGCATTTGACTGTGAGAAATTTGACTTTTCGGGGACCACTGGGGGAGCTGACTGAGTATGAATTTCTAACCTTATTAAGCTCTCTGGAACAATTAATATCTTTGGGTGGCTCCATGAAAGTGCTAACTTTGGAGCACGTTCGTAATAAAGTTTTTTATTTCAACCAGGAGATTCTATACCGACAGTTTTCAGAGATGAATATTGCCAGTTTGACAATATATGATGCATATATGCCACACATGGTTTGCCCCAATAGAACAAGCTCATTTCAGTATATAAATTTTTCTCACAATGCCCTGACGGATGAATTGTTCCAGAATTGTGGTACTCTGACAGATCTGAAATTACTTATTTTGGAGAAGAATAAATTTGAGAGCCTTTCCAAGGTAAGCTTCATGACCAGCCGTATGAAATTGCTGCAATATTTGGACATGAGCAACAACTTGCTGAGTCACGATGCAGCTGATGTGCAATGCCGGTGGGCTGAGTCTCTGACAGAGTTGGACCTGTCCTCAAATCAGTTGACGGATTCCGTGTTTGAGTGCTTGCCAGTCAACATCAAAAAACTCAACCTACAAAACAATCAGATCACCAGTGTCGCCAAAGGGATGGCTGAGCTGAAATCCTTGGAAGAGCTGAACCTGGCATCGAACAGGCTGGCTGACCTGCCGGGGTGCAGTGGCTTTATGTCCCTGGAGTTCCTGAACGTAGAGATGAATTTGATCCTCACCCCATCTGCCGACTTCTTCCAGAGCTGCCTAAGGGTCAAGGAGCTACAAGCCGGGCACAACCCGTTCAAGTGTTCCTGTGAACTGCAAGACTTTATCCGTCTGGAGAGGCAGTCTGGGGTGAAGCTGTCTGACTGGCCGGAGGCATA

>30589 TLR1A

TTACAATGGGCTATCATTTTGCAAATTTTCCTGTTTATTTGGGGTTCACCTGTGGAGCATTTGACTGTGAGAAATTTGACTTTTCGGGGACCACTGGGGGAGCTGACTGAGTATGAATTTCTAACCTTATTAAGCTCTCTGGAACAATTAATATCTTTGGGTGGCTCCATGAAAGTGCTAACTTTGGAGCACGTTCGTAATAAAGTTTTTTATTTCAACCAGGAGATTCTATACCGACAGTTTTCAGAGATGAATATTGCCAGTTTGACAATATATGATGCATATATGCCACACATGGTTTGCCCCAATAGAACAAGCTCATTTCAGTATATAAATTTTTCTCACAATGCCCTGACGGATGAATTGTTCCAGAATTGTGGTACTCTGACAGATCTGAAATTACTTATTTTGGAGAAGAATAAATTTGAGAGCCTTTCCAAGGTAAGCTTCATGACCAGCCGTATGAAATTGCTGCAATATTTGGACATGAGCAACAACTTGCTGAGTCACGATGCAGCTGATGTGCAATGCCGGTGGGCTGAGTCTCTGACAGAGTTGGACCTGTCCTCAAATCAGTTGACGGATTCCGTGTTTGAGTGCTTGCCAGTCAACATCAAAAAACTCAACCTACAAAACAATCAGATCACCAGTGTCGCCAAAGGGATGGCTGAGCTGAAATCCTTGGAAGAGCTGAACCTGGCATCGAACAGGCTGGCTGACCTGCCGGGGTGCAGTGGCTTTATGTCCCTGGAGTTCCTGAACGTAGAGATGAATTTGATCCTCACCCCATCTGCCGACTTCTTCCAGAGCTGCCTAAGGGTCAAGGAGCTACAAGCCGGGCACAACCCGTTCAAGTGTTCCTGTGAACTGCAAGACTTTATCCGTCTGGAGAGGCAGTCTGGGGTGAAGCTGTCTGACTGGCCGGAGGCATA

>30590 TLR1A

TTACAATGGGCTATCATTTTGCAAATTTTCCTGTTTATTTGGGGTTCACCTGTGGAGCATTTGACTGTGAGAAATTTGACTTTTCGGGGACCACTGGGGGAGCTGACTGAGTATGAATTTCTAACCTTATTAAGCTCTCTGGAACAATTAATATCTTTGGGTGGCTCCATGAAAGTGCTAACTTTGGAGCACGTTCGTAATAAAGTTTTTTATTTCAACCAGGAGATTCTATACCGACAGTTTTCAGAGATGAATATTGCCAGTTTGACAATATATGATGCATATATGCCACACATGGTTTGCCCCAATAGAACAAGCTCATTTCAGTATATAAATTTTTCTCACAATGCCCTGACGGATGAATTGTTCCAGAATTGTGGTACTCTGACAGATCTGAAATTACTTATTTTGGAGAAGAATAAATTTGAGAGCCTTTCCAAGGTAAGCTTCATGACCAGCCGTATGAAATTGCTGCAATATTTGGACATGAGCAACAACTTGCTGAGTCACGATGCAGCTGATGTGCAATGCCGGTGGGCTGAGTCTCTGACAGAGTTGGACCTGTCCTCAAATCAGTTGACGGATTCCGTGTTTGAGTGCTTGCCAGTCAACATCAAAAAACTCAACCTACAAAACAATCAGATCACCAGTGTCGCCAAAGGGATGGCTGAGCTGAAATCCTTGGAAGAGCTGAACCTGGCATCGAACAGGCTGGCTGACCTGCCGGGGTGCAGTGGCTTTATGTCCCTGGAGTTCCTGAACGTAGAGATGAATTTGATCCTCACCCCATCTGCCGACTTCTTCCAGAGCTGCCTAAGGGTCAAGGAGCTACAAGCCGGGCACAACCCGTTCAAGTGTTCCTGTGAACTGCAAGACTTTATCCGTCTGGAGAGGCAGTCTGGGGTGAAGCTGTCTGACTGGCCGGAGGCATA

>30591 TLR1A

TTACAATGGGCTATCATTTTGCAAATTTTCCTGTTTATTTGGGGTTCACCTGTGGAGCATTTGACTGTGAGAAATTTGACTTTTCGGGGACCACTGGGGGAGCTGACTGAGTATGAATTTCTAACCTTATTAAGCTCTCTGGAACAATTAATATCTTTGGGTGGCTCCATGAAAGTGCTAACTTTGGAGCACGTTCGTAATAAAGTTTTTTATTTCAACCAGGAGATTCTATACCGACAGTTTTCAGAGATGAATATTGCCAGTTTGACAATATATGATGCATATATGCCACACATGGTTTGCCCCAATAGAACAAGCTCATTTCAGTATATAAATTTTTCTCACAATGCCCTGACGGATGAATTGTTCCAGAATTGTGGTACTCTGACAGATCTGAAATTACTTATTTTGGAGAAGAATAAATTTGAGAGCCTTTCCAAGGTAAGCTTCATGACCAGCCGTATGAAATTGCTGCAATATTTGGACATGAGCAACAACTTGCTGAGTCACGATGCAGCTGATGTGCAATGCCGGTGGGCTGAGTCTCTGACAGAGTTGGACCTGTCCTCAAATCAGTTGACGGATTCCGTGTTTGAGTGCTTGCCAGTCAACATCAAAAAACTCAACCTACAAAACAATCAGATCACCAGTGTCGCCAAAGGGATGGCTGAGCTGAAATCCTTGGAAGAGCTGAACCTGGCATCGAACAGGCTGGCTGACCTGCCGGGGTGCAGTGGCTTTATGTCCCTGGAGTTCCTGAACGTAGAGATGAATTTGATCCTCACCCCATCTGCCGACTTCTTCCAGAGCTGCCTAAGGGTCAAGGAGCTACAAGCCGGGCACAACCCGTTCAAGTGTTCCTGTGAACTGCAAGACTTTATCCGTCTGGAGAGGCAGTCTGGGGTGAAGCTGTCTGACTGGCCGGAGGCATA

>30703 TLR1A

TTACAATGGGCTATCATTTTGCAAATTTTCCTGTTTATTTGGGGTTCACCTGTGGAGCATTTGACTGTGAGAAATTTGACTTTTCGGGGACCACTGGGGGAGCTGACTGAGTATGAATTTCTAACCTTATTAAGCTCTCTGGAACAATTAATATCTTTGGGTGGCTCCATGAAAGTGCTAACTTTGGAGCACGTTCGTAATAAAGTTTTTTATTTCAACCAGGAGATTCTATACCGACAGTTTTCAGAGATGAATATTGCCAGTTTGACAATATATGATGCATATATGCCACACATGGTTTGCCCCAATAGAACAAGCTCATTTCAGTATATAAATTTTTCTCACAATGCCCTGACGGATGAATTGTTCCAGAATTGTGGTACTCTGACAGATCTGAAATTACTTATTTTGGAGAAGAATAAATTTGAGAGCCTTTCCAAGGTAAGCTTCATGACCAGCCGTATGAAATTGCTGCAATATTTGGACATGAGCAACAACTTGCTGAGTCACGATGCAGCTGATGTGCAATGCCGGTGGGCTGAGTCTCTGACAGAGTTGGACCTGTCCTCAAATCAGTTGACGGATTCCGTGTTTGAGTGCTTGCCAGTCAACATCAAAAAACTCAACCTACAAAACAATCAGATCACCAGTGTCGCCAAAGGGATGGCTGAGCTGAAATCCTTGGAAGAGCTGAACTTGGCATCGAACAGGCTGGCTGACCTGCCGGGGTGCAGTGGCTTTATGTCCCTGGAGTTCCTGAACGTAGAGATGAATTTGATCCTCACCCCATCTGCTGACTTCTTCCAGAGCTGCCTAAGGGTCAAGGAGCTACAAGCCGGGCACAACCCGTTCAAGTGTTCCTGTGAACTGCAAGACTTTATCCGTCTGGAGAGGCAGTCTGGGGTGAAGCTGTCTGACTGGCCGGAGGCATA

>30704 TLR1A

TTACAATGGGCTATCATTTTGCAAATTTTCCTGTTTATTTGGGGTTCACCTGTGGAGCATTTGACTGTGAGAAATTTGACTTTTCGGGGACCACTGGGGGAGCTGACTGAGTATGAATTTCTAACCTTATTAAGCTCTCTGGAACAATTAATATCTTTGGGTGGCTCCATGAAAGTGCTAACTTTGGAGCACGTTCGTAATAAAGTTTTTTATTTCAACCAGGAGATTCTATACCGACAGTTTTCAGAGATGAATATTGCCAGTTTGACAATATATGATGCATATATGCCACACATGGTTTGCCCCAATAGAACAAGCTCATTTCAGTATATAAATTTTTCTCACAATGCCCTGACGGATGAATTGTTCCAGAATTGTGGTACTCTGACAGATCTGAAATTACTTATTTTGGAGAAGAATAAATTTGAGAGCCTTTCCAAGGTAAGCTTCATGACCAGCCGTATGAAATTGCTGCAATATTTGGACATGAGCAACAACTTGCTGAGTCACGATGCAGCTGATGTGCAATGCCGGTGGGCTGAGTCTCTGACAGAGTTGGACCTGTCCTCAAATCAGTTGACGGATTCCGTGTTTGAGTGCTTGCCAGTCAACATCAAAAAACTCAACCTACAAAACAATCAGATCACCAGTGTCGCCAAAGGGATGGCTGAGCTGAAATCCTTGGAAGAGCTGAACCTGGCATCGAACAGGCTGGCTGACCTGCCGGGGTGCAGTGGCTTTATGTCCCTGGAGTTCCTGAACGTAGAGATGAATTTGATCCTCACCCCATCTGCCGACTTCTTCCAGAGCTGCCTAAGGGTCAAGGAGCTACAAGCCGGGCACAACCCGTTCAAGTGTTCCTGTGAACTGCAAGACTTTATCCGTCTGGAGAGGCAGTCTGGGGTGAAGCTGTCTGACTGGCCGGAGGCATA

>30706 TLR1A

TTACAATGGGCTATCATTTTGCAAATTTTCCTGTTTATTTGGGGTTCACCTGTGGAGCATTTGACTGTGAGAAATTTGACTTTTCGGGGACCACTGGGGGAGCTGACTGAGTATGAATTTCTAACCTTATTAAGCTCTCTGGAACAATTAATATCTTTGGGTGGCTCCATGAAAGTGCTAACTTTGGAGCACGTTCGTAATAAAGTTTTTTATTTCAACCAGGAGATTCTATACCGACAGTTTTCAGAGATGAATATTGCCAGTTTGACAATATATGATGCATATATGCCACACATGGTTTGCCCCAATAGAACAAGCTCATTTCAGTATATAAATTTTTCTCACAATGCCCTGACGGATGAATTGTTCCAGAATTGTGGTACTCTGACAGATCTGAAATTACTTATTTTGGAGAAGAATAAATTTGAGAGCCTTTCCAAGGTAAGCTTCATGACCAGCCGTATGAAATTGCTGCAATATTTGGACATGAGCAACAACTTGCTGAGTCACGATGCAGCTGATGTGCAATGCCGGTGGGCTGAGTCTCTGACAGAGTTGGACCTGTCCTCAAATCAGTTGACGGATTCCGTGTTTGAGTGCTTGCCAGTCAACATCAAAAAACTCAACCTACAAAACAATCAGATCACCAGTGTCGCCAAAGGGATGGCTGAGCTGAAATCCTTGGAAGAGCTGAACCTGGCATCGAACAGGCTGGCTGACCTGCCGGGGTGCAGTGGCTTTATGTCCCTGGAGTTCCTGAACGTAGAGATGAATTTGATCCTCACCCCATCTGCCGACTTCTTCCAGAGCTGCCTAAGGGTCAAGGAGCTACAAGCCGGGCACAACCCGTTCAAGTGTTCCTGTGAACTGCAAGACTTTATCCGTCTGGAGAGGCAGTCTGGGGTGAAGCTGTCTGACTGGCCGGAGGCATA

>30708 TLR1A

TTACAATGGGCTATCATTTTGCAAATTTTCCTGTTTATTTGGGGTTCACCTGTGGAGCATTTGACTGTGAGAAATTTGACTTTTCGGGGACCACTGGGGGAGCTGACTGAGTATGAATTTCTAACCTTATTAAGCTCTCTGGAACAATTAATATCTTTGGGTGGCTCCATGAAAGTGCTAACTTTGGAGCACGTTCGTAATAAAGTTTTTTATTTCAACCAGGAGATTCTATACCGACAGTTTTCAGAGATGAATATTGCCAGTTTGACAATATATGATGCATATATGCCACACATGGTTTGCCCCAATAGAACAAGCTCATTTCAGTATATAAATTTTTCTCACAATGCCCTGACGGATGAATTGTTCCAGAATTGTGGTACTCTGACAGATCTGAAATTACTTATTTTGGAGAAGAATAAATTTGAGAGCCTTTCCAAGGTAAGCTTCATGACCAGCCGTATGAAATTGCTGCAATATTTGGACATGAGCAACAACTTGCTGAGTCACGATGCAGCTGATGTGCAATGCCGGTGGGCTGAGTCTCTGACAGAGTTGGACCTGTCCTCAAATCAGTTGACGGATTCCGTGTTTGAGTGCTTGCCAGTCAACATCAAAAAACTCAACCTACAAAACAATCAGATCACCAGTGTCGCCAAAGGGATGGCTGAGCTGAAATCCTTGGAAGAGCTGAACCTGGCATCGAACAGGCTGGCTGACCTGCCGGAGTGCAGTGGCTTTATGTCCCTGGAGTTCCTGAACGTAGAGATGAATTTGATCCTCACCCCATCTGCCGACTTCTTCCAGAGCTGCCTAAGGGTCAAGGAGCTACAAGCCGGGCACAACCCGTTCAAGTGTTCCTGTGAACTGCAAGACTTTATCCGTCTGGAGAGGCAGTCTGGGGTGAAGCTGTCTGACTGGCCGGAGGCATA

>30710 TLR1A

TTACAATGGGCTATCATTTTGCAAATTTTCCTGTTTATTTGGGGTTCACCTGTGGAGCATTTGACTGTGAGAAATTTGACTTTTCGGGGACCACTGGGGGAGCTGACTGAGTATGAATTTCTAACCTTATTAAGCTCTCTGGAACAATTAATATCTTTGGGTGGCTCCATGAAAGTGCTAACTTTGGAGCACGTTCGTAATAAAGTTTTTTATTTCAACCAGGAGATTCTATACCGACAGTTTTCAGAGATGAATATTGCCAGTTTGACAATATATGATGCATATATGCCACACATGGTTTGCCCCAATAGAACAAGCTCATTTCAGTATATAAATTTTTCTCACAATGCCCTGACGGATGAATTGTTCCAGAATTGTGGTACTCTGACAGATCTGAAATTACTTATTTTGGAGAAGAATAAATTTGAGAGCCTTTCCAAGGTAAGCTTCATGACCAGCCGTATGAAATTGCTGCAATATTTGGACATGAGCAACAACTTGCTGAGTCACGATGCAGCTGATGTGCAATGCCGGTGGGCTGAGTCTCTGACAGAGTTGGACCTGTCCTCAAATCAGTTGACGGATTCCGTGTTTGAGTGCTTGCCAGTCAACATCAAAAAACTCAACCTACAAAACAATCAGATCACCAGTGTCGCCAAAGGGATGGCTGAGCTGAAATCCTTGGAAGAGCTGAACTTGGCATCGAACAGGCTGGCTGACCTGCCGGGGTGCAGTGGCTTTATGTCCCTGGAGTTCCTGAACGTAGAGATGAATTTGATCCTCACCCCATCTGCCGACTTCTTCCAGAGCTGCCTAAGGGTCAAGGAGCTACAAGCCGGGCACAACCCGTTCAAGTGTTCCTGTGAACTGCAAGACTTTATCCGTCTGGAGAGGCAGTCTGGGGTGATGCTGTATGACTGGCCGGAGGCATA

>30711 TLR1A

TTACAATGGGCTATCATTTTGCAAATTTTCCTGTTTATTTGGGGTTCACCTGTGGAGCATTTGACTGTGAGAAATTTGACTTTTCGGGGACCACTGGGGGAGCTGACTGAGTATGAATTTCTAACCTTATTAAGCTCTCTGGAACAATTAATATCTTTGGGTGGCTCCATGAAAGTGCTAACTTTGGAGCACGTTCGTAATAAAGTTTTTTATTTCAACCAGGAGATTCTATACCGACAGTTTTCAGAGATGAATATTGCCAGTTTGACAATATATGATGCATATATGCCACACATGGTTTGCCCCAATAGAACAAGCTCATTTCAGTATATAAATTTTTCTCACAATGCCCTGACGGATGAATTGTTCCAGAATTGTGGTACTCTGACAGATCTGAAATTACTTATTTTGGAGAAGAATAAATTTGAGAGCCTTTCCAAGGTAAGCTTCATGACCAGCCGTATGAAATTGCTGCAATATTTGGACATGAGCAACAACTTGCTGAGTCACGATGCAGCTGATGTGCAATGCCGGTGGGCTGAGTCTCTGACAGAGTTGGACCTGTCCTCAAATCAGTTGACGGATTCCGTGTTTGAGTGCTTGCCAGTCAACATCAAAAAACTCAACCTACAAAACAATCAGATCACCAGTGTCGCCAAAGGGATGGCTGAGCTGAAATCCTTGGAAGAGCTGAACCTGGCATCGAACAGGCTGGCTGACCTGCCGGGGTGCAGTGGCTTTATGTCCCTGGAGTTCCTGAACGTAGAGATGAATTTGATCCTCACCCCATCTGCCGACTTCTTCCAGAGCTGCCTAAGGGTCAAGGAGCTACAAGCCGGGCACAACCCGTTCAAGTGTTCCTGTGAACTGCAAGACTTTATCCGTCTGGAGAGGCAGTCTGGGGTGAAGCTGTCTGACTGGCCGGAGGCATA

>46151 TLR1A

TTACAATGGGCTATCATTTTGCAAATTTTCCTGTTTATTTGGGGTTCACCTGTGGAGCATTTGACTGTGAGAAATTTGACTTTTCGGGGACCACTGGGGGAGCTGACTGAGTATGAATTTCTAACCTTATTAAGCTCTCTGGAACAATTAATATCTTTGGGTGGCTCCATGAAAGTGCTAACTTTGGAGCACGTTCGTAATAAAGTTTTTTATTTCAACCAGGAGATTCTATACCGACAGTTTTCAGAGATGAATATTGCCAGTTTGACAATATATGATGCATATATGCCACACATGGTTTGCCCCAATAGAACAAGCTCATTTCAGTATATAAATTTTTCTCACAATGCCCTGACGGATGAATTGTTCCAGAATTGTGGTACTCTGACAGATCTGAAATTACTTATTTTGGAGAAGAATAAATTTGAGAGCCTTTCCAAGGTAAGCTTCATGACCAGCCGTATGAAATTGCTGCAATATTTGGACATGAGCAACAACTTGCTGAGTCACGATGCAGCTGATGTGCAATGCCGGTGGGCTGAGTCTCTGACAGAGTTGGACCTGTCCTCAAATCAGTTGACGGATTCCGTGTTTGAGTGCTTGCCAGTCAACATCAAAAAACTCAACCTACAAAACAATCAGATCACCAGTGTCGCCAAAGGGATGGCTGAGCTGAAATCCTTGGAAGAGCTGAACCTGGCATCGAACAGGCTGGCTGACCTGCCGGGGTGCAGTGGCTTTATGTCCCTGGAGTTCCTGAACGTAGAGATGAATTTGATCCTCACCCCATCTGCCGACTTCTTCCAGAGCTGCCTAAGGGTCAAGGAGCTACAAGCCGGGCACAACCCGTTCAAGTGTTCCTGTGAACTGCAAGACTTTATCCGTCTGGAGAGGCAGTCTGGGGTGAAGCTGTCTGACTGGCCGGAGGCATA

>46152 TLR1A

TTACAATGGGCTATCATTTTGCAAATTTTCCTGTTTATTTGGGGTTCACCTGTGGAGCATTTGACTGTGAGAAATTTGACTTTTCGGGGACCACTGGGGGAGCTGACTGAGTATGAATTTCTAACCTTATTAAGCTCTCTGGAACAATTAATATCTTTGGGTGGCTCCATGAAAGTGCTAACTTTGGAGCACGTTCGTAATAAAGTTTTTTATTTCAACCAGGAGATTCTATACCGACAGTTTTCAGAGATGAATATTGCCAGTTTGACAATATATGATGCATATATGCCACACATGGTTTGCCCCAATAGAACAAGCTCATTTCAGTATATAAATTTTTCTCACAATGCCCTGACGGATGAATTGTTCCAGAATTGTGGTACTCTGACAGATCTGAAATTACTTATTTTGGAGAAGAATAAATTTGAGAGCCTTTCCAAGGTAAGCTTCATGACCAGCCGTATGAAATTGCTGCAATATTTGGACATGAGCAACAACTTGCTGAGTCACGATGCAGCTGATGTGCAATGCCGGTGGGCTGAGTCTCTGACAGAGTTGGACCTGTCCTCAAATCAGTTGACGGATTCCGTGTTTGAGTGCTTGCCAGTCAACATCAAAAAACTCAACCTACAAAACAATCAGATCACCAGTGTCGCCAAAGGGATGGCTGAGCTGAAATCCTTGGAAGAGCTGAACCTGGCATCGAACAGGCTGGCTGACCTGCCGGGGTGCAGTGGCTTTATGTCCCTGGAGTTCCTGAACGTAGAGATGAATTTGATCCTCACCCCATCTGCCGACTTCTTCCAGAGCTGCCTAAGGGTCAAGGAGCTACAAGCCGGGCACAACCCGTTCAAGTGTTCCTGTGAACTGCAAGACTTTATCCGTCTGGAGAGGCAGTCTGGGGTGAAGCTGTCTGACTGGCCGGAGGCATA

>46153 TLR1A

TTACAATGGGCTATCATTTTGCAAATTTTCCTGTTTATTTGGGGTTCACCTGTGGAGCATTTGACTGTGAGAAATTTGACTTTTCGGGGACCACTGGGGGAGCTGACTGAGTATGAATTTCTAACCTTATTAAGCTCTCTGGAACAATTAATATCTTTGGGTGGCTCCATGAAAGTGCTAACTTTGGAGCACGTTCGTAATAAAGTTTTTTATTTCAACCAGGAGATTCTATACCGACAGTTTTCAGAGATGAATATTGCCAGTTTGACAATATATGATGCATATATGCCACACATGGTTTGCCCCAATAGAACAAGCTCATTTCAGTATATAAATTTTTCTCACAATGCCCTGACGGATGAATTGTTCCAGAATTGTGGTACTCTGACAGATCTGAAATTACTTATTTTGGAGAAGAATAAATTTGAGAGCCTTTCCAAGGTAAGCTTCATGACCAGCCGTATGAAATTGCTGCAATATTGGGACATGAGCAACAACTTGCTGAGTCACGATGCAGCTGATGTGCAATGCCGGTGGGCTGAGTCTCTGACAGAGTTGGACCTGTCCTCAAATCAGTTGACGGATTCCGTGTTTGAGTGCTTGCCAGTCAACATCAAAAAACTCAACCTACAAAACAATCAGATCACCAGTGTCGCCAAAGGGATGGCTGAGCTGAAATCCTTGGAAGAGCTGAACCTGGCATCGAACAGGCTGGCTGACCTGCCGGGGTGCAGTGGCTTTATGTCCCTGGAGTTCCTGAACGTAGAGATGAATTTGATCCTCACCCCATCTGCCGACTTCTTCCAGAGCTGCCTAAGGGTCAAGGAGCTACAAGCCGGGCACAACCCGTTCAAGTGTTCCTGTGAACTGCAAGACTTTATCCGTCTGGAGAGGCAGTCTGGGGTGAAGCTGTCTGACTGGCCGGAGGCATA

>46154 TLR1A

TTACAATGGGCTATCATTTTGCAAATTTTCCTGTTTATTTGGGGTTCACCTGTGGAGCATTTGACTGTGAGAAATTTGACTTTTCGGGGACCACTGGGGGAGCTGACTGAGTATGAATTTCTAACCTTATTAAGCTCTCTGGAACAATTAATATCTTTGGGTGGCTCCATGAAAGTGCTAACTTTGGAGCACGTTCGTAATAAAGTTTTTTATTTCAACCAGGAGATTCTATACCGACAGTTTTCAGAGATGAATATTGCCAGTTTGACAATATATGATGCATATATGCCACACATGGTTTGCCCCAATAGAACAAGCTCATTTCAGTATATAAATTTTTCTCACAATGCCCTGACGGATGAATTGTTCCAGAATTGTGGTACTCTGACAGATCTGAAATTACTTATTTTGGAGAAGAATAAATTTGAGAGCCTTTCCAAGGTAAGCTTCATGACCAGCCGTATGAAATTGCTGCAATATTTGGACATGAGCAACAACTTGCTGAGTCACGATGCAGCTGATGTGCAATGCCGGTGGGCTGAGTCTCTGACAGAGTTGGACCTGTCCTCAAATCAGTTGACGGATTCCGTGTTTGAGTGCTTGCCAGTCAACATCAAAAAACTCAACCTACAAAACAATCAGATCACCAGTGTCGCCAAAGGGATGGCTGAGCTGAAATCCTTGGAAGAGCTGAACCTGGCATCGAACAGGCTGGCTGACCTGCCGGGGTGCAGTGGCTTTATGTCCCTGGAGTTCCTGAACGTAGAGATGAATTTGATCCTCACCCCATCTGCCGACTTCTTCCAGAGCTGCCTAAGGGTCAAGGAGCTACAAGCCGGGCACAACCCGTTCAAGTGTTCCTGTGAACTGCAAGACTTTATCCGTCTGGAGAGGCAGTCTGGGGTGAAGCTGTCTGACTGGCCGGAGGCATA

>46155 TLR1A

TTACAATGGGCTATCATTTTGCAAATTTTCCTGTTTATTTGGGGTTCACCTGTGGAGCATTTGACTGTGAGAAATTTGACTTTTCGGGGACCACTGGGGGAGCTGACTGAGTATGAATTTCTAACCTTATTAAGCTCTCTGGAACAATTAATATCTTTGGGTGGCTCCATGAAAGTGCTAACTTTGGAGCACGTTCGTAATAAAGTTTTTTATTTCAACCAGGAGATTCTATACCGACAGTTTTCAGAGATGAATATTGCCAGTTTGACAATATATGATGCATATATGCCACACATGGTTTGCCCCAATAGAACAAGCTCATTTCAGTATATAAATTTTTCTCACAATGCCCTGACGGATGAATTGTTCCAGAATTGTGGTACTCTGACAGATCTGAAATTACTTATTTTGGAGAAGAATAAATTTGAGAGCCTTTCCAAGGTAAGCTTCATGACCAGCCGTATGAAATTGCTGCAATATTTGGACATGAGCAACAACTTGCTGAGTCACGATGCAGCTGATGTGCAATGCCGGTGGGCTGAGTCTCTGACAGAGTTGGACCTGTCCTCAAATCAGTTGACGGATTCCGTGTTTGAGTGCTTGCCAGTCAACATCAAAAAACTCAACCTACAAAACAATCAGATCACCAGTGTCGCCAAAGGGATGGCTGAGCTGAAATCCTTGGAAGAGCTGAACCTGGCATCGAACAGGCTGGCTGACCTGCCGGGGTGCAGTGGCTTTATGTCCCTGGAGTTCCTGAACGTAGAGATGAATTTGATCCTCACCCCATCTGCCGACTTCTTCCAGAGCTGCCTAAGGGTCAAGGAGCTACAAGCCGGGCACAACCCGTTCAAGTGTTCCTGTGAACTGCAAGACTTTATCCGTCTGGAGAGGCAGTCTGGGGTGAAGCTGTCTGACTGGCCGGAGGCATA

>46157 TLR1A

TTACAATGGGCTATCATTTTGCAAATTTTCCTGTTTATTTGGGGTTCACCTGTGGAGCATTTGACTGTGAGAAATTTGACTTTTCGGGGACCACTGGGGGAGCTGACTGAGTATGAATTTCTAACCTTATTAAGCTCTCTGGAACAATTAATATCTTTGGGTGGCTCCATGAAAGTGCTAACTTTGGAGCACGTTCGTAATAAAGTTTTTTATTTCAACCAGGAGATTCTATACCGACAGTTTTCAGAGATGAATATTGCCAGTTTGACAATATATGATGCATATATGCCACACATGGTTTGCCCCAATAGAACAAGCTCATTTCAGTATATAAATTTTTCTCACAATGCCCTGACGGATGAATTGTTCCAGAATTGTGGTACTCTGACAGATCTGAAATTACTTATTTTGGAGAAGAATAAATTTGAGAGCCTTTCCAAGGTAAGCTTCATGACCAGCCGTATGAAATTGCTGCAATATTTGGACATGAGCAACAACTTGCTGAGTCACGATGCAGCTGATGTGCAATGCCGGTGGGCTGAGTCTCTGACAGAGTTGGACCTGTCCTCAAATCAGTTGACGGATTCCGTGTTTGAGTGCTTGCCAGTCAACATCAAAAAACTCAACCTACAAAACAATCAGATCACCAGTGTCGCCAAAGGGATGGCTGAGCTGAAATCCTTGGAAGAGCTGAACCTGGCATCGAACAGGCTGGCTGACCTGCCGGGGTGCAGTGGCTTTATGTCCCTGGAGTTCCTGAACGTAGAGATGAATTTGATCCTCACCCCATCTGCCGACTTCTTCCAGAGCTGCCTAAGGGTCAAGGAGCTACAAGCCGGGCACAACCCGTTCAAGTGTTCCTGTGAACTGCAAGACTTTATCCGTCTGGAGAGGCAGTCTGGGGTGAAGCTGTCTGACTGGCCGGAGGCATA

**TLR1LB**

>4156 TLR1B

TGGAATGCTCTTACTGAAGTTTTTCAGACTGTATGGCACTCATCCATTGAATACTTCAATATTAACAGTCTAACACAATTGTCGGACATCAAAAGATATGACTTTGACTATTCAGGTACCTCTATGAAAGCATTGACAATAAAGAAAGTTTTAATCACAGATCTGTACTTCTCACAGGATGACCTATACAAAATGTTTGCAGACATGAATATTGCAGCCTTGACAATAGCTGAATCAGAGATGATTCATATGCTGTGTCCTTTGTCTAACAGTCCCTTTAAATACTTAAATTTTTTAAAGAATGATTTAACAGATCTTCTTTTTCAAAATTGTGACAAATTAGTTCAACTGGATACATTAATCTTGCAGAAGAATAAATTTGAGAGCCTTTCCAAGGTAAGCTTCATGACCAGCCGTATGAAATTGCTGCAGTATTTGGACATGAGCAACAACTTGCTGAGTCACGATGCAGCTGATGTGCAATGCCGGTGGGCTGAGTCTCTGACAGAGTTGGACCTGTCCTCAAATCAGTTGACGGATTCCGTGTTTGAGTGCTTGCCAGTCAACATCAAAAAACTCAACCTACAAAACAATCAGATCACCAGTGTCACCAAAGGGATGGCTGAGCTGAAATCCTTGGAAGAGCTGAACCTGGCATCGAACAGGCTGGCTGACCTGCCGGAGTGCAGTGGCTTTATGTCCCTGGAGTTCCTGAACGTAGAGATGAATTTGATCCTCACCCCATCTGCCGACTTCTTCCAGAGCTGCCTAAGGGTCAAGGAGCTACAAGCCGGGCACAACCCGTTCAAGTGTTCCTGTGAACTGCAAGACTTTATCCGTCTGGAGAGGCAGTCTGGGGTGAAGCTGTCTGACTGGCCGGAGGCATACGTG

>4161 TLR1B

TGGAATGCTCTTACTGAAGTTTTTCAGACTGTATGGCACTCATCCATTGAATACTTCAATATTAACAGTCTAACACAATTGTCGGACATCAAAAGATATGACTTTGACTATTCAGGTACCTCTATGAAAGCATTGACAATAAAGAAAGTTTTAATCACAGATCTGTACTTCTCACAGGATGACCTATACAAAATGTTTGCAGACATGAATATTGCAGCCTTGACAATAGCTGAATCAGAGATGATTCATATGCTGTGTCCTTTGTCTAACAGTCCCTTTAAATACTTAAATTTTTTAAAGAATGATTTAACAGATCTTCTTTTTCAAAATTGTGACAAATTAGTTCAACTGGATACATTAATCTTGCAGAAGAATAAATTTGAGAGCCTTTCCAAGGTAAGCTTCATGACCAGCCGTATGAAATTGCTGCAGTATTTGGACATGAGCAACAACTTGCTGAGTCACGATGCAGCTGATGTGCAATGCCGGTGGGCTGAGTCTCTGACAGAGTTGGACCTGTCCTCAAATCAGTTGACGGATTCCGTGTTTGAGTGCTTGCCAGTCAACATCAAAAAACTCAACCTACAAAACAATCAGATCACCAGTGTCACCAAAGGGATGGCTGAGCTGAAATCCTTGGAAGAGCTGAACCTGGCATCGAACAGGCTGGCTGACCTGCCGGAGTGCAGTGGCTTTATGTCCCTGGAGTTCCTGAACGTAGAGATGAATTTGATCCTCACCCCATCTGCCGACTTCTTCCAGAGCTGCCTAAGGGTCAAGGAGCTACAAGCCAGGCACAACCCGTTCAAGTGTTCCTGTGAACTGCAAGACTTTATCCGTCTGGAGAGGCAGTCTGGGGTGAAGCTGTCTGACTGGCCGGAGGCATACGTG

>4177 TLR1B

TGGAATGCTCTTACTGAAGTTTTTCAGACTGTATGGCACTCATCCATTGAATACTTCAATATTAACAGTCTAACACAATTGTCGGACATCAAAAGATATGACTTTGACTATTCAGGTACCTCTATGAAAGCATTGACAATAAAGAAAGTTTTAATCACAGATCTGTACTTCTCACAGGATGACCTATACAAAATGTTTGCAGACATGAATATTGCAGCCTTGACAATAGCTGAATCAGAGATGATTCATATGCTGTGTCCTTTGTCTAACAGTCCCTTTAAATACTTAAATTTTTTAAAGAATGATTTAACAGATCTTCTTTTTCAAAATTGTGACAAATTAGTTCAACTGGATACATTAATCTTGCAGAAGAATAAATTTGAGAGCCTTTCCAAGGTAAGCTTCATGACCAGCCGTATGAAATTGCTGCAGTATTTGGACATGAGCAACAACTTGCTGAGTCACGATGCAGCTGATGTGCAATGCCGGTGGGCTGAGTCTCTGACAGAGTTGGACCTGTCCTCAAATCAGTTGACGGATTCCGTGTTTGAGTGCTTGCCAGTCAACATCAAAAAACTCAACCTACAAAACAATCAGATCACCAGTGTCACCAAAGGGATGGCTGAGCTGAAATCCTTGGAAGAGCTGAACCTGGCATCGAACAGGCTGGCTGACCTGCCGGAGTGCAGTGGCTTTATGTCCCTGGAGTTCCTGAACGTAGAGATGAATTTGATCCTCACCCCATCTGCCGACTTCTTCCAGAGCTGCCTAAGGGTCAAGGAGCTACAAGCCGGGCACAACCCGTTCAAGTGTTCCTGTGAACTGCAAGACTTTATCCGTCTGGAGAGGCAGTCTGGGGTGAAGCTGTCTGACTGGCCGGAGGCATACGTG

>711 TLR1B

TGGAATGCTCTTACTGAAGTTTTTCAGACTGTATGGCACTCATCCATTGAATACTTCAATATTAACAGTCTAACACAATTGTCGGACATCAAAAGATATGACTTTGACTATTCAGGTACCTCTATGAAAGCATTGACAATAAAGAAAGTTTTAATCACAGATCTGTACTTCTCACAGGATGACCTATACAAAATGTTTGCAGACATGAATATTGCAGCCTTGACAATAGCTGAATCAGAGATGATTCATATGCTGTGTCCTTTGTCTAACAGTCCCTTTAAATACTTAAATTTTTTAAAGAATGATTTAACAGATCTTCTTTTTCAAAATTGTGACAAATTAGTTCAACTGGATACATTAATCTTGCAGAAGAATAAATTTGAGAGCCTTTCCAAGGTAAGCTTCATGACCAGCCGTATGAAATTGCTGCAGTATTTGGACATGAGCAACAACTTGCTGAGTCACGATGCAGCTGATGTGCAATGCCGGTGGGCTGAGTCTCTGACAGAGTTGGACCTGTCCTCAAATCAGTTGACGGATTCCGTGTTTGAGTGCTTGCCAGTCAACATCAAAAAACTCAACCTACAAAACAATCAGATCACCAGTGTCACCAAAGGGATGGCTGAGCTGAAATCCTTGGAAGAGCTGAACCTGGCATCGAACAGGCTGGCTGACCTGCCGGAGTGCAGTGGCTTTATGTCCCTGGAGTTCCTGAACGTAGAGATGAATTTGATCCTCACCCCATCTGCCGACTTCTTCCAGAGCTGCCTAAGGGTCAAGGAGCTACAAGCCGGGCACAACCCGTTCAAGTGTTCCTGTGAACTGCAAGACTTTATCCGTCTGGAGAGGCAGTCTGGGGTGAAGCTGTCTGACTGGCCGGAGGCATACGTG

>713 TLR1B

TGGAATGCTCTTACTGAAGTTTTTCAGACTGTATGGCACTCATCCATTGAATACTTCAATATTAACAGTCTAACACAATTGTCGGACATCAAAAGATATGACTTTGACTATTCAGGTACCTCTATGAAAGCATTGACAATAAAGAAAGTTTTAATCACAGATCTGTACTTCTCACAGGATGACCTATACAAAATGTTTGCAGACATGAATATTGCAGCCTTGACAATAGCTGAATCAGAGATGATTCATATGCTGTGTCCTTTGTCTAACAGTCCCTTTAAATACTTAAATTTTTTAAAGAATGATTTAACAGATCTTCTTTTTCAAAATTGTGACAAATTAGTTCAACTGGATACATTAATCTTGCAGAAGAATAAATTTGAGAGCCTTTCCAAGGTAAGCTTCATGACCAGCCGTATGAAATTGCTGCAGTATTTGGACATGAGCAACAACTTGCTGAGTCACGATGCAGCTGATGTGCAATGCCGGTGGGCTGAGTCTCTGACAGAGTTGGACCTGTCCTCAAATCAGTTGACGGATTCCGTGTTTGAGTGCTTGCCAGTCAACATCAAAAAACTCAACCTACAAAACAATCAGATCACCAGTGTCACCAAAGGGATGGCTGAGCTGAAATCCTTGGAAGAGCTGAACCTGGCATCGAACAGGCTGGCTGACCTGCCGGAGTGCAGTGGCTTTATGTCCCTGGAGTTCCTGAACGTAGAGATGAATTTGATCCTCACCCCATCTGCCGACTTCTTCCAGAGCTGCCTAAGGGTCAAGGAGCTACAAGCCGGGCACAACCCGTTCAAGTGTTCCTGTGAACTGCAAGACTTTATCCGTCTGGAGAGGCAGTCTGGGGTGAAGCTGTCTGACTGGCCGGAGGCATACGTG

>715 TLR1B

TGGAATGCTCTTACTGAAGTTTTTCAGACTGTATGGCACTCATCCATTGAATACTTCAATATTAACAGTCTAACACAATTGTCGGACATCAAAAGATATGACTTTGACTATTCAGGTACCTCTATGAAAGCATTGACAATAAAGAAAGTTTTAATCACAGATCTGTACTTCTCACAGGATGACCTATACAAAATGTTTGCAGACATGAATATTGCAGCCTTGACAATAGCTGAATCAGAGATGATTCATATGCTGTGTCCTTTGTCTAACAGTCCCTTTAAATACTTAAATTTTTTAAAGAATGATTTAACAGATCTTCTTTTTCAAAATTGTGACAAATTAGTTCAACTGGATACATTAATCTTGCAGAAGAATAAATTTGAGAGCCTTTCCAAGGTAAGCTTCATGACCAGCCGTATGAAATTGCTGCAGTATTTGGACATGAGCAACAACTTGCTGAGTCACGATGCAGCTGATGTGCAATGCCGGTGGGCTGAGTCTCTGACAGAGTTGGACCTGTCCTCAAATCAGTTGACGGATTCCGTGTTTGAGTGCTTGCCAGTCAACATCAAAAAACTCAACCTACAAAACAATCAGATCACCAGTGTCACCAAAGGGATGGCTGAGCTGAAATCCTTGGAAGAGCTGAACCTGGCATCGAACAGGCTGGCTGACCTGCCGGAGTGCAGTGGCTTTATGTCCCTGGAGTTCCTGAACGTAGAGATGAATTTGATCCTCACCCCATCTGCCGACTTCTTCCAGAGCTGCCTAAGGGTCAAGGAGCTACAAGCCGGGCACAACCCGTTCAAGTGTTCCTGTGAACTGCAAGACTTTATCCGTCTGGAGAGGCAGTCTGGGGTGAAGCTGTCTGACTGGCCGGAGGCATACGTG

>822 TLR1B

TGGAATGCTCTTACTGAAGTTTTTCAGACTGTATGGCACTCATCCATTGAATACTTCAATATTAACAGTCTAACACAATTGTCGGACATCAAAAGATATGACTTTGACTATTCAGGTACCTCTATGAAAGCATTGACAATAAAGAAAGTTTTAATCACAGATCTGTACTTCTCACAGGATGACCTATACAAAATGTTTGCAGACATGAATATTGCAGCCTTGACAATAGCTGAATCAGAGATGATTCATATGCTGTGTCCTTTGTCTAACAGTCCCTTTAAATACTTAAATTTTTTAAAGAATGATTTAACAGATCTTCTTTTTCAAAATTGTGACAAATTAGTTCAACTGGATACATTAATCTTGCAGAAGAATAAATTTGAGAGCCTTTCCAAGGTAAGCTTCATGACCAGCCGTATGAAATTGCTGCAGTATTTGGACATGAGCAACAACTTGCTGAGTCACGATGCAGCTGATGTGCAATGCCGGTGGGCTGAGTCTCTGACAGAGTTGGACCTGTCCTCAAATCAGTTGACGGATTCCGTGTTTGAGTGCTTGCCAGTCAACATCAAAAAACTCAACCTACAAAACAATCAGATCACCAGTGTCACCAAAGGGATGGCTGAGCTGAAATCCTTGGAAGAGCTGAACCTGGCATCGAACAGGCTGGCTGACCTGCCGGAGTGCAGTGGCTTTATGTCCCTGGAGTTCCTGAACGTAGAGATGAATTTGATCCTCACCCCATCTGCCGACTTCTTCCAGAGCTGCCTAAGGGTCAAGGAGCTACAAGCCGGGCACAACCCGTTCAAGTGTTCCTGTGAACTGCAAGACTTTATCCGTCTGGAGAGGCAGTCTGGGGTGAAGCTGTCTGACTGGCCGGAGGCATACGTG

>823 TLR1B

TGGAATGCTCTTACTGAAGTTTTTCAGACTGTATGGCACTCATCCATTGAATACTTCAATATTAACAGTCTAACACAATTGTCGGACATCAAAAGATATGACTTTGACTATTCAGGTACCTCTATGAAAGCATTGACAATAAAGAAAGTTTTAATCACAGATCTGTACTTCTCACAGGATGACCTATACAAAATGTTTGCAGACATGAATATTGCAGCCTTGACAATAGCTGAATCAGAGATGATTCATATGCTGTGTCCTTTGTCTAACAGTCCCTTTAAATACTTAAATTTTTTAAAGAATGATTTAACAGATCTTCTTTTTCAAAATTGTGACAAATTAGTTCAACTGGATACATTAATCTTGCAGAAGAATAAATTTGAGAGCCTTTCCAAGGTAAGCTTCATGACCAGCCGTATGAAATTGCTGCAGTATTTGGACATGAGCAACAACTTGCTGAGTCACGATGCAGCTGATGTGCAATGCCGGTGGGCTGAGTCTCTGACAGAGTTGGACCTGTCCTCAAATCAGTTGACGGATTCCGTGTTTGAGTGCTTGCCAGTCAACATCAAAAAACTCAACCTACAAAACAATCAGATCACCAGTGTCACCAAAGGGATGGCTGAGCTGAAATCCTTGGAAGAGCTGAACCTGGCATCGAACAGGCTGGCTGACCTGCCGGAGTGCAGTGGCTTTATGTCCCTGGAGTTCCTGAACGTAGAGATGAATTTGATCCTCACCCCATCTGCCGACTTCTTCCAGAGCTGCCTAAGGGTCAAGGAGCTACAAGCCGGGCACAACCCGTTCAAGTGTTCCTGTGAACTGCAAGACTTTATCCGTCTGGAGAGGCAGTCTGGGGTGAAGCTGTCTGACTGGCCGGAGGCATACGTG

>826 TLR1B

TGGAATGCTCTTACTGAAGTTTTTCAGACTGTATGGCACTCATCCATTGAATACTTCAATATTAACAGTCTAACACAATTGTCGGACATCAAAAGATATGACTTTGACTATTCAGGTACCTCTATGAAAGCATTGACAATAAAGAAAGTTTTAATCACAGATCTGTACTTCTCACAGGATGACCTATACAAAATGTTTGCAGACATGAATATTGCAGCCTTGACAATAGCTGAATCAGAGATGATTCATATGCTGTGTCCTTTGTCTAACAGTCCCTTTAAATACTTAAATTTTTTAAAGAATGATTTAACAGATCTTCTTTTTCAAAATTGTGACAAATTAGTTCAACTGGATACATTAATCTTGCAGAAGAATAAATTTGAGAGCCTTTCCAAGGTAAGCTTCATGACCAGCCGTATGAAATTGCTGCAGTATTTGGACATGAGCAACAACTTGCTGAGTCACGATGCAGCTGATGTGCAATGCCGGTGGGCTGAGTCTCTGACAGAGTTGGACCTGTCCTCAAATCAGTTGACGGATTCCGTGTTTGAGTGCTTGCCAGTCAACATCAAAAAACTCAACCTACAAAACAATCAGATCACCAGTGTCACCAAAGGGATGGCTGAGCTGAAATCCTTGGAAGAGCTGAACCTGGCATCGAACAGGCTGGCTGACCTGCCGGAGTGCAGTGGCTTTATGTCCCTGGAGTTCCTGAACGTAGAGATGAATTTGATCCTCACCCCATCTGCCGACTTCTTCCAGAGCTGCCTAAGGGTCAAGGAGCTACAAGCCGGGCACAACCCGTTCAAGTGTTCCTGTGAACTGCAAGACTTTATCCGTCTGGAGAGGCAGTCTGGGGTGAAGCTGTCTGACTGGCCGGAGGCATACGTG

>103 TLR1B

TGGAATGCTCTTACTGAAGTTTTTCAGACTGTATGGCACTCATCCATTGAATACTTCAATATTAACAGTCTAACACAATTGTCGGACATCAAAAGATATGACTTTGACTATTCAGGTACCTCTATGAAAGCATTGACAATAAAGAAAGTTTTAATCACAGATCTGTACTTCTCACAGGATGACCTATACAAAATGTTTGCAGACATGAATATTGCAGCCTTGACAATAGCTGAATCAGAGATGATTCATATGCTGTGTCCTTTGTCTAACAGTCCCTTTAAATACTTAAATTTTTTAAAGAATGATTTAACAGATCTTCTTTTTCAAAATTGTGACAAATTAGTTCAACTGGATACATTAATCTTGCAGAAGAATAAATTTGAGAGCCTTTCCAAGGTAAGCTTCATGACCAGCCGTATGAAATTGCTGCAGTATTTGGACATGAGCAACAACTTGCTGAGTCACGATGCAGCTGATGTGCAATGCCGGTGGGCTGAGTCTCTGACAGAGTTGGACCTGTCCTCAAATCAGTTGACGGATTCCGTGTTTGAGTGCTTGCCAGTCAACATCAAAAAACTCAACCTACAAAACAATCAGATCACCAGTGTCACCAAAGGGATGGCTGAGCTGAAATCCTTGGAAGAGCTGAACCTGGCATCGAACAGGCTGGCTGACCTGCCGGAGTGCAGTGGCTTTATGTCCCTGGAGTTCCTGAACGTAGAGATGAATTTGATCCTCACCCCATCTGCCGACTTCTTCCAGAGCTGCCTAAGGGTCAAGGAGCTACAAGCCGGGCACAACCCGTTCAAGTGTTCCTGTGAACTGCAAGACTTTATCCGTCTGGAGAGGCAGTCTGGGGTGAAGCTGTCTGACTGGCCGGAGGCATACGTG

>1011 TLR1B

TGGAATGCTCTTACTGAAGTTTTTCAGACTGTATGGCACTCATCCATTGAATACTTCAATATTAACAGTCTAACACAATTGTCGGACATCAAAAGATATGACTTTGACTATTCAGGTACCTCTATGAAAGCATTGACAATAAAGAAAGTTTTAATCACAGATCTGTACTTCTCACAGGATGACCTATACAAAATGTTTGCAGACATGAATATTGCAGCCTTGACAATAGCTGAATCAGAGATGATTCATATGCTGTGTCCTTTGTCTAACAGTCCCTTTAAATACTTAAATTTTTTAAAGAATGATTTAACAGATCTTCTTTTTCAAAATTGTGACAAATTAGTTCAACTGGATACATTAATCTTGCAGAAGAATAAATTTGAGAGCCTTTCCAAGGTAAGCTTCATGACCAGCCGTATGAAATTGCTGCAATATTTGGACATGAGCAACAACTTGCTGAGTCACGATGCAGCTGATGTGCAATGCCGGTGGGCTGAGTCTCTGACAGAGTTGGACCTGTCCTCAAATCAGTTGACGGATTCCGTGTTTGAGTGCTTGCCAGTCAACATCAAAAAACTCAACCTACAAAACAATCAGATCACCAGTGTCGCCAAAGGGATGGCTGAGCTGAAATCCTTGGAAGAGCTGAACCTGGCATCGAACAGGCTGGCTGACCTGCCGGGGTGCAGTGGCTTTATGTCCCTGGAGTTCCTGAACGTAGAGATGAATTTGATCCTCACCCCATCTGCCGACTTCTTCCAGAGCTGCCTAAGGGTCAAGGAGCTACAAGCCGGGCACAACCCGTTCAAGTGTTCCTGTGAACTGCAAGACTTTATCCGTCTGGAGAGGCAGTCTGGGGTGAAGCTGTCTGACTGGCCGGAGGCATACGTG

>1017 TLR1B

TGGAATGCTCTTACTGAAGTTTTTCAGACTGTATGGCACTCATCCATTGAATACTTCAATATTAACAGTCTAACACAATTGTCGGACATCAAAAGATATGACTTTGACTATTCAGGTACCTCTATGAAAGCATTGACAATAAAGAAAGTTTTAATCACAGATCTGTACTTCTCACAGGATGACCTATACAAAATGTTTGCAGACATGAATATTGCAGCCTTGACAATAGCTGAATCAGAGATGATTCATATGCTGTGTCCTTTGTCTAACAGTCCCTTTAAATACTTAAATTTTTTAAAGAATGATTTAACAGATCTTCTTTTTCAAAATTGTGACAAATTAGTTCAACTGGATACATTAATCTTGCAGAAGAATAAATTTGAGAGCCTTTCCAAGGTAAGCTTCATGACCAGCCGTATGAAATTGCTGCAGTATTTGGACATGAGCAACAACTTGCTGAGTCACGATGCAGCTGATGTGCAATGCCGGTGGGCTGAGTCTCTGACAGAGTTGGACCTGTCCTCAAATCAGTTGACGGATTCCGTGTTTGAGTGCTTGCCAGTCAACATCAAAAAACTCAACCTACAAAACAATCAGATCACCAGTGTCACCAAAGGGATGGCTGAGCTGAAATCCTTGGAAGAGCTGAACCTGGCATCGAACAGGCTGGCTGACCTGCCGGAGTGCAGTGGCTTTATGTCCCTGGAGTTCCTGAACGTAGAGATGAATTTGATCCTCACCCCATCTGCCGACTTCTTCCAGAGCTGCCTAAGGGTCAAGGAGCTACAAGCCGGGCACAACCCGTTCAAGTGTTCCTGTGAACTGCAAGACTTTATCCGTCTGGAGAGGCAGTCTGGGGTGAAGCTGTCTGACTGGCCGGAGGCATACGTG

>1210 TLR1B

TGGAATGCTCTTACTGAAGTTTTTCAGACTGTATGGCACTCATCCATTGAATACTTCAATATTAACAGTCTAACACAATTGTCGGACATCAAAAGATATGACTTTGACTATTCAGGTACCTCTATGAAAGCATTGACAATAAAGAAAGTTTTAATCACAGATCTGTACTTCTCACAGGATGACCTATACAAAATGTTTGCAGACATGAATATTGCAGCCTTGACAATAGCTGAATCAGAGATGATTCATATGCTGTGTCCTTTGTCTAACAGTCCCTTTAAATACTTAAATTTTTTAAAGAATGATTTAACAGATCTTCTTTTTCAAAATTGTGACAAATTAGTTCAACTGGATACATTAATCTTGCAGAAGAATAAATTTGAGAGCCTTTCCAAGGTAAGCTTCATGACCAGCCGTATGAAATTGCTGCAGTATTTGGACATGAGCAACAACTTGCTGAGTCACGATGCAGCTGATGTGCAATGCCGGTGGGCTGAGTCTCTGACAGAGTTGGACCTGTCCTCAAATCAGTTGACGGATTCCGTGTTTGAGTGCTTGCCAGTCAACATCAAAAAACTCAACCTACAAAACAATCAGATCACCAGTGTCACCAAAGGGATGGCTGAGCTGAAATCCTTGGAAGAGCTGAACCTGGCATCGAACAGGCTGGCTGACCTGCCGGAGTGCAGTGGCTTTATGTCCCTGGAGTTCCTGAACGTAGAGATGAATTTGATCCTCACCCCATCTGCCGACTTCTTCCAGAGCTGCCTAAGGGTCAAGGAGCTACAAGCCGGGCACAACCCGTTCAAGTGTTCCTGTGAACTGCAAGACTTTATCCGTCTGGAGAGGCAGTCTGGGGTGAAGCTGTCTGACTGGCCGGAGGCATACGTG

>1211 TLR1B

TGGAATGCTCTTACTGAAGTTTTTCAGACTGTATGGCACTCATCCATTGAATACTTCAATATTAACAGTCTAACACAATTGTCGGACATCAAAAGATATGACTTTGACTATTCAGGTACCTCTATGAAAGCATTGACAATAAAGAAAGTTTTAATCACAGATCTGTACTTCTCACAGGATGACCTATACAAAATGTTTGCAGACATGAATATTGCAGCCTTGACAATAGCTGAATCAGAGATGATTCATATGCTGTGTCCTTTGTCTAACAGTCCCTTTAAATACTTAAATTTTTTAAAGAATGATTTAACAGATCTTCTTTTTCAAAATTGTGACAAATTAGTTCAACTGGATACATTAATCTTGCAGAAGAATAAATTTGAGAGCCTTTCCAAGGTAAGCTTCATGACCAGCCGTATGAAATTGCTGCAGTATTTGGACATGAGCAACAACTTGCTGAGTCACGATGCAGCTGATGTGCAATGCCGGTGGGCTGAGTCTCTGACAGAGTTGGACCTGTCCTCAAATCAGTTGACGGATTCCGTGTTTGAGTGCTTGCCAGTCAACATCAAAAAACTCAACCTACAAAACAATCAGATCACCAGTGTCACCAAAGGGATGGCTGAGCTGAAATCCTTGGAAGAGCTGAACCTGGCATCGAACAGGCTGGCTGACCTGCCGGAGTGCAGTGGCTTTATGTCCCTGGAGTTCCTGAACGTAGAGATGAATTTGATCCTCACCCCATCTGCCGACTTCTTCCAGAGCTGCCTAAGGGTCAAGGAGCTACAAGCCGGGCACAACCCGTTCAAGTGTTCCTGTGAACTGCAAGACTTTATCCGTCTGGAGAGGCAGTCTGGGGTGAAGCTGTCTGACTGGCCGGAGGCATACGTG

>1214 TLR1B

TGGAATGCTCTTACTGAAGTTTTTCAGACTGTATGGCACTCATCCATTGAATACTTCAATATTAACAGTCTAACACAATTGTCGGACATCAAAAGATATGACTTTGACTATTCAGGTACCTCTATGAAAGCATTGACAATAAAGAAAGTTTTAATCACAGATCTGTACTTCTCACAGGATGACCTATACAAAATGTTTGCAGACATGAATATTGCAGCCTTGACAATAGCTGAATCAGAGATGATTCATATGCTGTGTCCTTTGTCTAACAGTCCCTTTAAATACTTAAATTTTTTAAAGAATGATTTAACAGATCTTCTTTTTCAAAATTGTGACAAATTAGTTCAACTGGATACATTAATCTTGCAGAAGAATAAATTTGAGAGCCTTTCCAAGGTAAGCTTCATGACCAGCCGTATGAAATTGCTGCAATATTTGGACATGAGCAACAACTTGCTGAGTCACGATGCAGCTGATGTGCAATGCCGGTGGGCTGAGTCTCTGACAGAGTTGGACCTGTCCTCAAATCAGTTGACGGATTCCGTGTTTGAGTGCTTGCCAGTCAACATCAAAAAACTCAACCTACAAAACAATCAGATCACCAGTGTCACCAAAGGGATGGCTGAGCTGAAATCCTTGGAAGAGCTGAACCTGGCATCGAACAGGCTGGCTGACCTGCCGGGGTGCAGTGGCTTTATGTCCCTGGAGTTCCTGAACGTAGAGATGAATTTGATCCTCACCCCATCTGCCGACTTCTTCCAGAGCTGCCTAAGGGTCAAGGAGCTACAAGCCGGGCACAACCCGTTCAAGTGTTCCTGTGAACTGCAAGACTTTATCCGTCTGGAGAGGCAGTCTGGGGTGAAGCTGTCTGACTGGCCGGAGGCATACGTG

>169 TLR1B

TGGAATGCTCTTACTGAAGTTTTTCAGACTGTATGGCACTCATCCATTGAATACTTCAATATTAACAGTCTAACACAATTGTCGGACATCAAAAGATATGACTTTGACTATTCAGGTACCTCTATGAAAGCATTGACAATAAAGAAAGTTTTAATCACAGATCTGTACTTCTCACAGGATGACCTATACAAAATGTTTGCAGACATGAATATTGCAGCCTTGACAATAGCTGAATCAGAGATGATTCATATGCTGTGTCCTTTGTCTAACAGTCCCTTTAAATACTTAAATTTTTTAAAGAATGATTTAACAGATCTTCTTTTTCAAAATTGTGACAAATTAGTTCAACTGGATACATTAATCTTGCAGAAGAATAAATTTGAGAGCCTTTCCAAGGTAAGCTTCATGACCAGCCGTATGAAATTGCTGCAATATTTGGACATGAGCAACAACTTGCTGAGTCACGATGCAGCTGATGTGCAATGCCGGTGGGCTGAGTCTCTGACAGAGTTGGACCTGTCCTCAAATCAGTTGACGGATTCCGTGTTTGAGTGCTTGCCAGTCAACATCAAAAAACTCAACCTACAAAACAATCAGATCACCAGTGTCACCAAAGGGATGGCTGAGCTGAAATCCTTGGAAGAGCTGAACCTGGCATCGAACAGGCTGGCTGACCTGCCGGAGTGCAGTGGCTTTATGTCCCTGGAGTTCCTGAACGTAGAGATGAATTTGATCCTCACCCCATCTGCCGACTTCTTCCAGAGCTGCCTAAGGGTCAAGGAGCTACAAGCCGGGCACAACCCGTTCAAGTGTTCCTGTGAACTGCAAGACTTTATCCGTCTGGAGAGGCAGTCTGGGGTGAAGCTGTCTGACTGGCCGGAGGCATACGTG

>1610 TLR1B

TGGAATGCTCTTACTGAAGTTTTTCAGACTGTATGGCACTCATCCATTGAATACTTCAATATTAACAGTCTAACACAATTGTCGGACATCAAAAGATATGACTTTGACTATTCAGGTACCTCTATGAAAGCATTGACAATAAAGAAAGTTTTAATCACAGATCTGTACTTCTCACAGGATGACCTATACAAAATGTTTGCAGACATGAATATTGCAGCCTTGACAATAGCTGAATCAGAGATGATTCATATGCTGTGTCCTTTGTCTAACAGTCCCTTTAAATACTTAAATTTTTTAAAGAATGATTTAACAGATCTTCTTTTTCAAAATTGTGACAAATTAGTTCAACTGGATACATTAATCTTGCAGAAGAATAAATTTGAGAGCCTTTCCAAGGTAAGCTTCATGACCAGCCGTATGAAATTGCTGCAGTATTTGGACATGAGCAACAACTTGCTGAGTCACGATGCAGCTGATGTGCAATGCCGGTGGGCTGAGTCTCTGACAGAGTTGGACCTGTCCTCAAATCAGTTGACGGATTCCGTGTTTGAGTGCTTGCCAGTCAACATCAAAAAACTCAACCTACAAAACAATCAGATCACCAGTGTCACCAAAGGGATGGCTGAGCTGAAATCCTTGGAAGAGCTGAACCTGGCATCGAACAGGCTGGCTGACCTGCCGGAGTGCAGTGGCTTTATGTCCCTGGAGTTCCTGAACGTAGAGATGAATTTGATCCTCACCCCATCTGCCGACTTCTTCCAGAGCTGCCTAAGGGTCAAGGAGCTACAAGCCGGGCACAACCCGTTCAAGTGTTCCTGTGAACTGCAAGACTTTATCCGTCTGGAGAGGCAGTCTGGGGTGAAGCTGTCTGACTGGCCGGAGGCATACGTG

>1616 TLR1B

TGGAATGCTCTTACTGAAGTTTTTCAGACTGTATGGCACTCATCCATTGAATACTTCAATATTAACAGTCTAACACAATTGTCGGACATCAAAAGATATGACTTTGACTATTCAGGTACCTCTATGAAAGCATTGACAATAAAGAAAGTTTTAATCACAGATCTGTACTTCTCACAGGATGACCTATACAAAATGTTTGCAGACATGAATATTGCAGCCTTGACAATAGCTGAATCAGAGATGATTCATATGCTGTGTCCTTTGTCTAACAGTCCCTTTAAATACTTAAATTTTTTAAAGAATGATTTAACAGATCTTCTTTTTCAAAATTGTGACAAATTAGTTCAACTGGATACATTAATCTTGCAGAAGAATAAATTTGAGAGCCTTTCCAAGGTAAGCTTCATGACCAGCCGTATGAAATTGCTGCAGTATTTGGACATGAGCAACAACTTGCTGAGTCACGATGCAGCTGATGTGCAATGCCGGTGGGCTGAGTCTCTGACAGAGTTGGACCTGTCCTCAAATCAGTTGACGGATTCCGTGTTTGAGTGCTTGCCAGTCAACATCAAAAAACTCAACCTACAAAACAATCAGATCACCAGTGTCACCAAAGGGATGGCTGAGCTGAAATCCTTGGAAGAGCTGAACCTGGCATCGAACAGGCTGGCTGACCTGCCGGAGTGCAGTGGCTTTATGTCCCTGGAGTTCCTGAACGTAGAGATGAATTTGATCCTCACCCCATCTGCCGACTTCTTCCAGAGCTGCCTAAGGGTCAAGGAGCTACAAGCCGGGCACAACCCGTTCAAGTGTTCCTGTGAACTGCAAGACTTTATCCGTCTGGAGAGGCAGTCTGGGGTGAAGCTGTCTGACTGGCCGGAGGCATACGTG

>175 TLR1B

TGGAATGCTCTTACTGAAGTTTTTCAGACTGTATGGCACTCATCCATTGAATACTTCAATATTAACAGTCTAACACAATTGTCGGACATCAAAAGATATGACTTTGACTATTCAGGTACCTCTATGAAAGCATTGACAATAAAGAAAGTTTTAATCACAGATCTGTACTTCTCACAGGATGACCTATACAAAATGTTTGCAGACATGAATATTGCAGCCTTGACAATAGCTGAATCAGAGATGATTCATATGCTGTGTCCTTTGTCTAACAGTCCCTTTAAATACTTAAATTTTTTAAAGAATGATTTAACAGATCTTCTTTTTCAAAATTGTGACAAATTAGTTCAACTGGATACATTAATCTTGCAGAAGAATAAATTTGAGAGCCTTTCCAAGGTAAGCTTCATGACCAGCCGTATGAAATTGCTGCAGTATTTGGACATGAGCAACAACTTGCTGAGTCACGATGCAGCTGATGTGCAATGCCGGTGGGCTGAGTCTCTGACAGAGTTGGACCTGTCCTCAAATCAGTTGACGGATTCCGTGTTTGAGTGCTTGCCAGTCAACATCAAAAAACTCAACCTACAAAACAATCAGATCACCAGTGTCACCAAAGGGATGGCTGAGCTGAAATCCTTGGAAGAGCTGAACCTGGCATCGAACAGGCTGGCTGACCTGCCGGAGTGCAGTGGCTTTATGTCCCTGGAGTTCCTGAACGTAGAGATGAATTTGATCCTCACCCCATCTGCCGACTTCTTCCAGAGCTGCCTAAGGGTCAAGGAGCTACAAGCCGGGCACAACCCGTTCAAGTGTTCCTGTGAACTGCAAGACTTTATCCGTCTGGAGAGGCAGTCTGGGGTGAAGCTGTCTGACTGGCCGGAGGCATACGTG

>176 TLR1B

TGGAATGCTCTTACTGAAGTTTTTCAGACTGTATGGCACTCATCCATTGAATACTTCAATATTAACAGTCTAACACAATTGTCGGACATCAAAAGATATGACTTTGACTATTCAGGTACCTCTATGAAAGCATTGACAATAAAGAAAGTTTTAATCACAGATCTGTACTTCTCACAGGATGACCTATACAAAATGTTTGCAGACATGAATATTGCAGCCTTGACAATAGCTGAATCAGAGATGATTCATATGCTGTGTCCTTTGTCTAACAGTCCCTTTAAATACTTAAATTTTTTAAAGAATGATTTAACAGATCTTCTTTTTCAAAATTGTGACAAATTAGTTCAACTGGATACATTAATCTTGCAGAAGAATAAATTTGAGAGCCTTTCCAAGGTAAGCTTCATGACCAGCCGTATGAAATTGCTGCAGTATTTGGACATGAGCAACAACTTGCTGAGTCACGATGCAGCTGATGTGCAATGCCGGTGGGCTGAGTCTCTGACAGAGTTGGACCTGTCCTCAAATCAGTTGACGGATTCCGTGTTTGAGTGCTTGCCAGTCAACATCAAAAAACTCAACCTACAAAACAATCAGATCACCAGTGTCACCAAAGGGATGGCTGAGCTGAAATCCTTGGAAGAGCTGAACCTGGCATCGAACAGGCTGGCTGACCTGCCGGAGTGCAGTGGCTTTATGTCCCTGGAGTTCCTGAACGTAGAGATGAATTTGATCCTCACCCCATCTGCCGACTTCTTCCAGAGCTGCCTAAGGGTCAAGGAGCTACAAGCCGGGCACAACCCGTTCAAGTGTTCCTGTGAACTGCAAGACTTTATCCGTCTGGAGAGGCAGTCTGGGGTGAAGCTGTCTGACTGGCCGGAGGCATACGTG

>1714 TLR1B

TGGAATGCTCTTACTGAAGTTTTTCAGACTGTATGGCACTCATCCATTGAATACTTCAATATTAACAGTCTAACACAATTGTCGGACATCAAAAGATATGACTTTGACTATTCAGGTACCTCTATGAAAGCATTGACAATAAAGAAAGTTTTAATCACAGATCTGTACTTCTCACAGGATGACCTATACAAAATGTTTGCAGACATGAATATTGCAGCCTTGACAATAGCTGAATCAGAGATGATTCATATGCTGTGTCCTTTGTCTAACAGTCCCTTTAAATACTTAAATTTTTTAAAGAATGATTTAACAGATCTTCTTTTTCAAAATTGTGACAAATTAGTTCAACTGGATACATTAATCTTGCAGAAGAATAAATTTGAGAGCCTTTCCAAGGTAAGCTTCATGACCAGCCGTATGAAATTGCTGCAGTATTTGGACATGAGCAACAACTTGCTGAGTCACGATGCAGCTGATGTGCAATGCCGGTGGGCTGAGTCTCTGACAGAGTTGGACCTGTCCTCAAATCAGTTGACGGATTCCGTGTTTGAGTGCTTGCCAGTCAACATCAAAAAACTCAACCTACAAAACAATCAGATCACCAGTGTCACCAAAGGGATGGCTGAGCTGAAATCCTTGGAAGAGCTGAACCTGGCATCGAACAGGCTGGCTGACCTGCCGGAGTGCAGTGGCTTTATGTCCCTGGAGTTCCTGAACGTAGAGATGAATTTGATCCTCACCCCATCTGCCGACTTCTTCCAGAGCTGCCTAAGGGTCAAGGAGCTACAAGCCGGGCACAACCCGTTCAAGTGTTCCTGTGAACTGCAAGACTTTATCCGTCTGGAGAGGCAGTCTGGGGTGAAGCTGTCTGACTGGCCGGAGGCATACGTG

>30583 TLR1B

TGGAATGCTCTTACTGAAGTTTTTCAGACTGTATGGCACTCATCCATTGAATACTTCAATATTAACAGTCTAACACAATTGTCGGACATCAAAAGATATGACTTTGACTATTCAGGTACCTCTATGAAAGCATTGACAATAAAGAAAGTTTTAATCACAGATCTGTACTTCTCACAGGATGACCTATACAAAATGTTTGCAGACATGAATATTGCAGCCTTGACAATAGCTGAATCAGAGATGATTCATATGCTGTGTCCTTTGTCTAACAGTCCCTTTAAATACTTAAATTTTTTAAAGAATGATTTAACAGATCTTCTTTTTCAAAATTGTGACAAATTAGTTCAACTGGATACATTAATCTTGCAGAAGAATAAATTTGAGAGCCTTTCCAAGGTAAGCTTCATGACCAGCCGTATGAAATTGCTGCAGTATTTGGACATGAGCAACAACTTGCTGAGTCACGATGCAGCTGATGTGCAATGCCGGTGGGCTGAGTCTCTGACAGAGTTGGACCTGTCCTCAAATCAGTTGACGGATTCCGTGTTTGAGTGCTTGCCAGTCAACATCAAAAAACTCAACCTACAAAACAATCAGATCACCAGTGTCACCAAAGGGATGGCTGAGCTGAAATCCTTGGAAGAGCTGAACCTGGCATCGAACAGGCTGGCTGACCTGCCGGAGTGCAGTGGCTTTATGTCCCTGGAGTTCCTGAACGTAGAGATGAATTTGATCCTCACCCCATCTGCCGACTTCTTCCAGAGCTGCCTAAGGGTCAAGGAGCTACAAGCCGGGCACAACCCGTTCAAGTGTTCCTGTGAACTGCAAGACTTTATCCGTCTGGAGAGGCAGTCTGGGGTGAAGCTGTCTGACTGGCCGGAGGCATACGTG

>30585 TLR1B

TGGAATGCTCTTACTGAAGTTTTTCAGACTGTATGGCACTCATCCATTGAATACTTCAATATTAACAGTCTAACACAATTGTCGGACATCAAAAGATATGACTTTGACTATTCAGGTACCTCTATGAAAGCATTGACAATAAAGAAAGTTTTAATCACAGATCTGTACTTCTCACAGGATGACCTATACAAAATGTTTGCAGACATGAATATTGCAGCCTTGACAATAGCTGAATCAGAGATGATTCATATGCTGTGTCCTTTGTCTAACAGTCCCTTTAAATACTTAAATTTTTTAAAGAATGATTTAACAGATCTTCTTTTTCAAAATTGTGACAAATTAGTTCAACTGGATACATTAATCTTGCAGAAGAATAAATTTGAGAGCCTTTCCAAGGTAAGCTTCATGACCAGCCGTATGAAATTGCTGCAGTATTTGGACATGAGCAACAACTTGCTGAGTCACGATGCAGCTGATGTGCAATGCCGGTGGGCTGAGTCTCTGACAGAGTTGGACCTGTCCTCAAATCAGTTGACGGATTCCGTGTTTGAGTGCTTGCCAGTCAACATCAAAAAACTCAACCTACAAAACAATCAGATCACCAGTGTCACCAAAGGGATGGCTGAGCTGAAATCCTTGGAAGAGCTGAACCTGGCATCGAACAGGCTGGCTGACCTGCCGGAGTGCAGTGGCTTTATGTCCCTGGAGTTCCTGAACGTAGAGATGAATTTGATCCTCACCCCATCTGCCGACTTCTTCCAGAGCTGCCTAAGGGTCAAGGAGCTACAAGCCGGGCACAACCCGTTCAAGTGTTCCTGTGAACTGCAAGACTTTATCCGTCTGGAGAGGCAGTCTGGGGTGAAGCTGTCTGACTGGCCGGAGGCATACGTG

>30586 TLR1B

TGGAATGCTCTTACTGAAGTTTTTCAGACTGTATGGCACTCATCCATTGAATACTTCAATATTAACAGTCTAACACAATTGTCGGACATCAAAAGATATGACTTTGACTATTCAGGTACCTCTATGAAAGCATTGACAATAAAGAAAGTTTTAATCACAGATCTGTACTTCTCACAGGATGACCTATACAAAATGTTTGCAGACATGAATATTGCAGCCTTGACAATAGCTGAATCAGAGATGATTCATATGCTGTGTCCTTTGTCTAACAGTCCCTTTAAATACTTAAATTTTTTAAAGAATGATTTAACAGATCTTCTTTTTCAAAATTGTGACAAATTAGTTCAACTGGATACATTAATCTTGCAGAAGAATAAATTTGAGAGCCTTTCCAAGGTAAGCTTCATGACCAGCCGTATGAAATTGCTGCAGTATTTGGACATGAGCAACAACTTGCTGAGTCACGATGCAGCTGATGTGCAATGCCGGTGGGCTGAGTCTCTGACAGAGTTGGACCTGTCCTCAAATCAGTTGACGGATTCCGTGTTTGAGTGCTTGCCAGTCAACATCAAAAAACTCAACCTACAAAACAATCAGATCACCAGTGTCACCAAAGGGATGGCTGAGCTGAAATCCTTGGAAGAGCTGAACCTGGCATCGAACAGGCTGGCTGACCTGCCGGAGTGCAGTGGCTTTATGTCCCTGGAGTTCCTGAACGTAGAGATGAATTTGATCCTCACCCCATCTGCCGACTTCTTCCAGAGCTGCCTAAGGGTCAAGGAGCTACAAGCCGGGCACAACCCGTTCAAGTGTTCCTGTGAACTGCAAGACTTTATCCGTCTGGAGAGGCAGTCTGGGGTGAAGCTGTCTGACTGGCCGGAGGCATACGTG

>30588 TLR1B

TGGAATGCTCTTACTGAAGTTTTTCAGACTGTATGGCACTCATCCATTGAATACTTCAATATTAACAGTCTAACACAATTGTCGGACATCAAAAGATATGACTTTGACTATTCAGGTACCTCTATGAAAGCATTGACAATAAAGAAAGTTTTAATCACAGATCTGTACTTCTCACAGGATGACCTATACAAAATGTTTGCAGACATGAATATTGCAGCCTTGACAATAGCTGAATCAGAGATGATTCATATGCTGTGTCCTTTGTCTAACAGTCCCTTTAAATACTTAAATTTTTTAAAGAATGATTTAACAGATCTTCTTTTTCAAAATTGTGACAAATTAGTTCAACTGGATACATTAATCTTGCAGAAGAATAAATTTGAGAGCCTTTCCAAGGTAAGCTTCATGACCAGCCGTATGAAATTGCTGCAGTATTTGGACATGAGCAACAACTTGCTGAGTCACGATGCAGCTGATGTGCAATGCCGGTGGGCTGAGTCTCTGACAGAGTTGGACCTGTCCTCAAATCAGTTGACGGATTCCGTGTTTGAGTGCTTGCCAGTCAACATCAAAAAACTCAACCTACAAAACAATCAGATCACCAGTGTCACCAAAGGGATGGCTGAGCTGAAATCCTTGGAAGAGCTGAACCTGGCATCGAACAGGCTGGCTGACCTGCCGGAGTGCAGTGGCTTTATGTCCCTGGAGTTCCTGAACGTAGAGATGAATTTGATCCTCACCCCATCTGCCGACTTCTTCCAGAGCTGCCTAAGGGTCAAGGAGCTACAAGCCGGGCACAACCCGTTCAAGTGTTCCTGTGAACTGCAAGACTTTATCCGTCTGGAGAGGCAGTCTGGGGTGAAGCTGTCTGACTGGCCGGAGGCATACGTG

>30589 TLR1B

TGGAATGCTCTTACTGAAGTTTTTCAGACTGTATGGCACTCATCCATTGAATACTTCAATATTAACAGTCTAACACAATTGTCGGACATCAAAAGATATGACTTTGACTATTCAGGTACCTCTATGAAAGCATTGACAATAAAGAAAGTTTTAATCACAGATCTGTACTTCTCACAGGATGACCTATACAAAATGTTTGCAGACATGAATATTGCAGCCTTGACAATAGCTGAATCAGAGATGATTCATATGCTGTGTCCTTTGTCTAACAGTCCCTTTAAATACTTAAATTTTTTAAAGAATGATTTAACAGATCTTCTTTTTCAAAATTGTGACAAATTAGTTCAACTGGATACATTAATCTTGCAGAAGAATAAATTTGAGAGCCTTTCCAAGGTAAGCTTCATGACCAGCCGTATGAAATTGCTGCAGTATTTGGACATGAGCAACAACTTGCTGAGTCACGATGCAGCTGATGTGCAATGCCGGTGGGCTGAGTCTCTGACAGAGTTGGACCTGTCCTCAAATCAGTTGACGGATTCCGTGTTTGAGTGCTTGCCAGTCAACATCAAAAAACTCAACCTACAAAACAATCAGATCACCAGTGTCACCAAAGGGATGGCTGAGCTGAAATCCTTGGAAGAGCTGAACCTGGCATCGAACAGGCTGGCTGACCTGCCGGAGTGCAGTGGCTTTATGTCCCTGGAGTTCCTGAACGTAGAGATGAATTTGATCCTCACCCCATCTGCCGACTTCTTCCAGAGCTGCCTAAGGGTCAAGGAGCTACAAGCCGGGCACAACCCGTTCAAGTGTTCCTGTGAACTGCAAGACTTTATCCGTCTGGAGAGGCAGTCTGGGGTGAAGCTGTCTGACTGGCCGGAGGCATACGTG

>30590 TLR1B

TGGAATGCTCTTACTGAAGTTTTTCAGACTGTATGGCACTCATCCATTGAATACTTCAATATTAACAGTCTAACACAATTGTCGGACATCAAAAGATATGACTTTGACTATTCAGGTACCTCTATGAAAGCATTGACAATAAAGAAAGTTTTAATCACAGATCTGTACTTCTCACAGGATGACCTATACAAAATGTTTGCAGACATGAATATTGCAGCCTTGACAATAGCTGAATCAGAGATGATTCATATGCTGTGTCCTTTGTCTAACAGTCCCTTTAAATACTTAAATTTTTTAAAGAATGATTTAACAGATCTTCTTTTTCAAAATTGTGACAAATTAGTTCAACTGGATACATTAATCTTGCAGAAGAATAAATTTGAGAGCCTTTCCAAGGTAAGCTTCATGACCAGCCGTATGAAATTGCTGCAGTATTTGGACATGAGCAACAACTTGCTGAGTCACGATGCAGCTGATGTGCAATGCCGGTGGGCTGAGTCTCTGACAGAGTTGGACCTGTCCTCAAATCAGTTGACGGATTCCGTGTTTGAGTGCTTGCCAGTCAACATCAAAAAACTCAACCTACAAAACAATCAGATCACCAGTGTCACCAAAGGGATGGCTGAGCTGAAATCCTTGGAAGAGCTGAACCTGGCATCGAACAGGCTGGCTGACCTGCCGGAGTGCAGTGGCTTTATGTCCCTGGAGTTCCTGAACGTAGAGATGAATTTGATCCTCACCCCATCTGCCGACTTCTTCCAGAGCTGCCTAAGGGTCAAGGAGCTACAAGCCGGGCACAACCCGTTCAAGTGTTCCTGTGAACTGCAAGACTTTATCCGTCTGGAGAGGCAGTCTGGGGTGAAGCTGTCTGACTGGCCGGAGGCATACGTG

>30591 TLR1B

TGGAATGCTCTTACTGAAGTTTTTCAGACTGTATGGCACTCATCCATTGAATACTTCAATATTAACAGTCTAACACAATTGTCGGACATCAAAAGATATGACTTTGACTATTCAGGTACCTCTATGAAAGCATTGACAATAAAGAAAGTTTTAATCACAGATCTGTACTTCTCACAGGATGACCTATACAAAATGTTTGCAGACATGAATATTGCAGCCTTGACAATAGCTGAATCAGAGATGATTCATATGCTGTGTCCTTTGTCTAACAGTCCCTTTAAATACTTAAATTTTTTAAAGAATGATTTAACAGATCTTCTTTTTCAAAATTGTGACAAATTAGTTCAACTGGATACATTAATCTTGCAGAAGAATAAATTTGAGAGCCTTTCCAAGGTAAGCTTCATGACCAGCCGTATGAAATTGCTGCAGTATTTGGACATGAGCAACAACTTGCTGAGTCACGATGCAGCTGATGTGCAATGCCGGTGGGCTGAGTCTCTGACAGAGTTGGACCTGTCCTCAAATCAGTTGACGGATTCCGTGTTTGAGTGCTTGCCAGTCAACATCAAAAAACTCAACCTACAAAACAATCAGATCACCAGTGTCACCAAAGGGATGGCTGAGCTGAAATCCTTGGAAGAGCTGAACCTGGCATCGAACAGGCTGGCTGACCTGCCGGAGTGCAGTGGCTTTATGTCCCTGGAGTTCCTGAACGTAGAGATGAATTTGATCCTCACCCCATCTGCCGACTTCTTCCAGAGCTGCCTAAGGGTCAAGGAGCTACAAGCCGGGCACAACCCGTTCAAGTGTTCCTGTGAACTGCAAGACTTTATCCGTCTGGAGAGGCAGTCTGGGGTGAAGCTGTCTGACTGGCCGGAGGCATACGTG

>30703 TLR1B

TGGGATGCTCTTACTGAAGTTTTTCAGACTGTATGGCACTCATCCATTGAATACTTCAATATTAACAGTCTAACACAATTGTCGGACATCAAAAGATATGACTTTGACTATTCAGGTACCTCTATGAAAGCATTGACAATAAAGAAAGTTTTAATCACAGATCTGTACTTCTCACAGGATGACCTATACAAAATGTTTGCAGACATGAATATTGCAGCCTTGACAATAGCTGAATCAGAGATGATTCATATGCTGTGTCCTTTGTCTAACAGTCCCTTTAAATACTTAAATTTTTTAAAGAATGATTTAACAGATCTTCTTTTTCAAAATTGTGACAAATTAGTTCAACTGGATACATTAATCTTGCAGAAGAATAAATTTGAGAGCCTTTCCAAGGTAAGCTTCATGACCAGCCGTATGAAATTGCTGCAGTATTTGGACATGAGCAACAACTTGCTGAGTCACGATGCAGCTGATGTGCAATGCCGGTGGGCTGAGTCTCTGACAGAGTTGGACCTGTCCTCAAATCAGTTGACGGATTCCGTGTTTGAGTGCTTGCCAGTCAACATCAAAAAACTCAACCTACAAAACAATCAGATCACCAGTGTCACCAAAGGGATGGCTGAGCTGAAATCCTTGGAAGAGCTGAACCTGGCATCGAACAGGCTGGCTGACCTGCCGGAGTGCAGTGGCTTTATGTCCCTGGAGTTCCTGAACGTAGAGATGAATTTGATCCTCACCCCATCTGCCGACTTCTTCCAGAGCTGCCTAAGGGTCAAGGAGCTACAAGCCGGGCACAACCCGTTCAAGTGTTCCTGTGAACTGCAAGACTTTATCCGTCTGGAGAGGCAGTCTGGGGTGAAGCTGTCTGACTGGCCGGAGGCATACGTG

>30704 TLR1B

TGGAATGCTCTTACTGAAGTTTTTCAGACTGTATGGCACTCATCCATTGAATACTTCAATATTAACAGTCTAACACAATTGTCGGACATCGAAAGATATGACTTTGACTATTCAGGTACCTCTATGAAAGCATTGACAATAAAGAAAGTTTTAATCACAGATCTGTACTTCTCACAGGATGACCTATACAAAATGTTTGCAGACATGAATATTGCAGCCTTGACAATAGCTGAATCAGAGATGATTCATATGCTGTGTCCTTTGTCTAACAGTCCCTTTAAATACTTAAATTTTTTAAAGAATGATTTAACAGATCTTCTTTTTCAAAATTGTGACAAATTAGTTCAACTGGATACATTAATCTTGCAGAAGAATAAATTTGAGAGCCTTTCCAAGGTAAGCTTCATGACCAGCCGTATGAAATTGCTGCAGTATTTGGACATGAGCAACAACTTGCTGAGTCACGATGCAGCTGATGTGCAATGCCGGTGGGCTGAGTCTCTGACAGAGTTGGACCTGTCCTCAAATCAGTTGACGGATTCCGTGTTTGAGTGCTTGCCAGTCAACATCAAAAAACTCAACCTACAAAACAATCAGATCACCAGTGTCACCAAAGGGATGGCTGAGCTGAAATCCTTGGAAGAGCTGAACCTGGCATCGAACAGGCTGGCTGACCTGCCGGAGTGCAGTGGCTTTATGTCCCTGGAGTTCCTGAACGTAGAGATGAATTTGATCCTCACCCCATCTGCCGACTTCTTCCAGAGCTGCCTAAGGGTCAAGGAGCTACAAGCCGGGCACAACCCGTTCAAGTGTTCCTGTGAACTGCAAGACTTTATCCGTCTGGAGAGGCAGTCTGGGGTGAAGCTGTCTGACTGGCCGGAGGCATACGTG

>30706 TLR1B

TGGGATGCTCTTACTGAAGTTTTTCAGACTGTATGGCACTCATCCATTGAATACTTCAATATTAACAGTCTAACACAATTGTCGGACATCAAAAGATATGACTTTGACTATTCAGGTACCTCTATGAAAGCATTGACAATAAAGAAAGTTTTAATCACAGATCTGTACTTCTCACAGGATGACCTATACAAAATGTTTGCAGACATGAATATTGCAGCCTTGACAATAGCTGAATCAGAGATGATTCATATGCTGTGTCCTTTGTCTAACAGTCCCTTTAAATACTTAAATTTTTTAAAGAATGATTTAACAGATCTTCTTTTTCAAAATTGTGACAAATTAGTTCAACTGGATACATTAATCTTGCAGAAGAATAAATTTGAGAGCCTTTCCAAGGTAAGCTTCATGACCAGCCGTATGAAATTGCTGCAGTATTTGGACATGAGCAACAACTTGCTGAGTCACGATGCAGCTGATGTGCAATGCCGGTGGGCTGAGTCTCTGACAGAGTTGGACCTGTCCTCAAATCAGTTGACGGATTCCGTGTTTGAGTGCTTGCCAGTCAACATCAAAAAACTCAACCTACAAAACAATCAGATCACCAGTGTCACCAAAGGGATGGCTGAGCTGAAATCCTTGGAAGAGCTGAACCTGGCATCGAACAGGCTGGCTGACCTGCCGGAGTGCAGTGGCTTTATGTCCCTGGAGTTCCTGAACGTAGAGATGAATTTGATCCTCACCCCATCTGCCGACTTCTTCCAGAGCTGCCTAAGGGTCAAGGAGCTACAAGCCGGGCACAACCCGTTCAAGTGTTCCTGTGAACTGCAAGACTTTATCCGTCTGGAGAGGCAGTCTGGGGTGAAGCTGTCTGACTGGCCGGAGGCATACGTG

>30708 TLR1B

TGGGATGCTCTTACTGAAGTTTTTCAGACTGTATGGCACTCATCCATTGAATACTTCAATATTAACAGTCTAACACAATTGTCGGACATCAAAAGATATGACTTTGACTATTCAGGTACCTCTATGAAAGCATTGACAATAAAGAAAGTTTTAATCACAGATCTGTACTTCTCACAGGATGACCTATACAAAATGTTTGCAGACATGAATATTGCAGCCTTGACAATAGCTGAATCAGAGATGATTCATATGCTGTGTCCTTTGTCTAACAGTCCCTTTAAATACTTAAATTTTTTAAAGAATGATTTAACAGATCTTCTTTTTCAAAATTGTGACAAATTAGTTCAACTGGATACATTAATCTTGCAGAAGAATAAATTTGAGAGCCTTTCCAAGGTAAGCTTCATGACCAGCCGTATGAAATTGCTGCAGTATTTGGACATGAGCAACAACTTGCTGAGTCACGATGCAGCTGATGTGCAATGCCGGTGGGCTGAGTCTCTGACAGAGTTGGACCTGTCCTCAAATCAGTTGACGGATTCCGTGTTTGAGTGCTTGCCAGTCAACATCAAAAAACTCAACCTACAAAACAATCAGATCACCAGTGTCACCAAAGGGATGGCTGAGCTGAAATCCTTGGAAGAGCTGAACCTGGCATCGAACAGGCTGGCTGACCTGCCGGAGTGCAGTGGCTTTATGTCCCTGGAGTTCCTGAACGTAGAGATGAATTTGATCCTCACCCCATCTGCCGACTTCTTCCAGAGCTGCCTAAGGGTCAAGGAGCTACAAGCCGGGCACAACCCGTTCAAGTGTTCCTGTGAACTGCAAGACTTTATCCGTCTGGAGAGGCAGTCTGGGGTGAAGCTGTCTGACTGGCCGGAGGCATACGTG

>30710 TLR1B

TGGAATGCTCTTACTGAAGTTTTTCAGACTGTATGGCACTCATCCATTGAATACTTCAATATTAACAGTCTAACACAATTGTCGGACATCAAAAGATATGACTTTGACTATTCAGGTACCTCTATGAAAGCATTGACAATAAAGAAAGTTTTAATCACAGATCTGTACTTCTCACAGGATGACCTATACAAAATGTTTGCAGACATGAATATTGCAGCCTTGACAATAGCTGAATCAGAGATGATTCATATGCTGTGTCCTTTGTCTAACAGTCCCTTTAAATACTTAAATTTTTTAAAGAATGATTTAACAGATCTTCTTTTTCAAAATTGTGACAAATTAGTTCAACTGGATACATTAATCTTGCAGAAGAATAAATTTGAGAGCCTTTCCAAGGTAAGCTTCATGACCAGCCGTATGAAATTGCTGCAGTATTTGGACATGAGCAACAACTTGCTGAGTCACGATGCAGCTGATGTGCAATGCCGGTGGGCTGAGTCTCTGACAGAGTTGGACCTGTCCTCAAATCAGTTGACGGATTCCGTGTTTGAGTGCTTGCCAGTCAACATCAAAAAACTCAACCTACAAAACAATCAGATCACCAGTGTCACCAAAGGGATGGCTGAGCTGAAATCCTTGGAAGAGCTGAACCTGGCATCGAACAGGCTGGCTGACCTGCCGGAGTGCAGTGGCTTTATGTCCCTGGAGTTCCTGAACGTAGAGATGAATTTGATCCTCACCCCATCTGCCGACTTCTTCCAGAGCTGCCTAAGGGTCAAGGAGCTACAAGCCGGGCACAACCCGTTCAAGTGTTCCTGTGAACTGCAAGACTTTATCCGTCTGGAGAGGCAGTCTGGGGTGAAGCTGTCTGACTGGCCGGAGGCATACGTG

>30711 TLR1B

TGGGATGCTCTTACTGAAGTTTTTCAGACTGTATGGCACTCATCCATTGAATACTTCAATATTAACAGTCTAACACAATTGTCGGACATCAAAAGATATGACTTTGACTATTCAGGTACCTCTATGAAAGCATTGACAATAAAGAAAGTTTTAATCACAGATCTGTACTTCTCACAGGATGACCTATACAAAATGTTTGCAGACATGAATATTGCAGCCTTGACAATAGCTGAATCAGAGATGATTCATATGCTGTGTCCTTTGTCTAACAGTCCCTTTAAATACTTAAATTTTTTAAAGAATGATTTAACAGATCTTCTTTTTCAAAATTGTGACAAATTAGTTCAACTGGATACATTAATCTTGCAGAAGAATAAATTTGAGAGCCTTTCCAAGGTAAGCTTCATGACCAGCCGTATGAAATTGCTGCAGTATTTGGACATGAGCAACAACTTGCTGAGTCACGATGCAGCTGATGtGCAATGCCGGTGGGCTGAGTCTCTGACAGAGTTGGACCTGTCCTCAAATCAGTTGACGGATTCCGTGTTTGAGTGCTTGCCAGTCAACATCAAAAAACTCAACCTACAAAACAATCAGATCACCAGTGTCACCAAAGGGATGGCTGAGCTGAAATCCTTGGAAGAGCTGAACCTGGCATCGAACAGGCTGGCTGACCTGCCGGAGTGCAGTGGCTTTATGTCCCTGGAGTTCCTGAACGTAGAGATGAATTTGATCCTCACCCCATCTGCCGACTTCTTCCAGAGCTGCCTAAGGGTCAAGGAGCTACAAGCCGGGCACAACCCGTTCAAGTGTTCCTGTGAACTGCAAGACTTTATCCGTCTGGAGAGGCAGTCTGGGGTGAAGCTGTCTGACTGGCCGGAGGCATACGTG

>46151 TLR1B

TGGAATGCTCTTACTGAAGTTTTTCAGACTGTATGGCACTCATCCATTGAATACTTCAATATTAACAGTCTAACACAATTGTCGGACATCAAAAGATATGACTTTGACTATTCAGGTACCTCTATGAAAGCATTGACAATAAAGAAAGTTTTAATCACAGATCTGTACTTCTCACAGGATGACCTATACAAAATGTTTGCAGACATGAATATTGCAGCCTTGACAATAGCTGAATCAGAGATGATTCATATGCTGTGTCCTTTGTCTAACAGTCCCTTTAAATACTTAAATTTTTTAAAGAATGATTTAACAGATCTTCTTTTTCAAAATTGTGACAAATTAGTTCAACTGGATACATTAATCTTGCAGAAGAATAAATTTGAGAGCCTTTCCAAGGTAAGCTTCATGACCAGCCGTATGAAATTGCTGCAATATTTGGACATGAGCAACAACTTGCTGAGTCACGATGCAGCTGATGtGCAATGCCGGTGGGCTGAGTCTCTGACAGAGTTGGACCTGTCCTCAAATCAGTTGACGGATTCCGTGTTTGAGTGCTTGCCAGTCAACATCAAAAAACTCAACCTACAAAACAATCAGATCACCAGTGTCACCAAAGGGATGGCTGAGCTGAAATCCTTGGAAGAGCTGAACCTGGCATCGAACAGGCTGGCTGACCTGCCGGAGTGCAGTGGCTTTATGTCCCTGGAGTTCCTGAACGTAGAGATGAATTTGATCCTCACCCCATCTGCCGACTTCTTCCAGAGCTGCCTAAGGGTCAAGGAGCTACAAGCCGGGCACAACCCGTTCAAGTGTTCCTGTGAACTGCAAGACTTTATCCGTCTGGAGAGGCAGTCTGGGGTGAAGCTGTCTGACTGGCCGGAGGCATACGTG

>46152 TLR1B

TGGAATGCTCTTACTGAAGTTTTTCAGACTGTATGGCACTCATCCATTGAATACTTCAATATTAACAGACTAACACAATTGTCGGACATCAAAAGATATGACTTTGACTATTCAGGTACCTCTATGAAAGCATTGACAATAAAGAAAGTTTTAATCACAGATCTGTACTTCTCACAGGATGACCTATACAAAATGTTTGCAGACATGAATATTGCAGCCTTGACAATAGCTGAATCAGAGATGATTCATATGCTGTGTCCTTTGTCTAACAGTCCCTTTAAATACTTAAATTTTTTAAAGAATGATTTAACAGATCTTCTTTTTCAAAATTGTGACAAATTAGTTCAACTGGATACATTAATCTTGCAGAAGAATAAATTTGAGAGCCTTTCCAAGGTAAGCTTCATGACCAGCCGTATGAAATTGCTGCAATATTTGGACATGAGCAACAACTTGCTGAGTCACGATGCAGCTGATGTGCAATGCCGGTGGGCTGAGTCTCTGACAGAGTTGGACCTGTCCTCAAATCAGTTGACGGATTCCGTGTTTGAGTGCTTGCCAGTCAACATCAAAAAACTCAACCTACAAAACAATCAGATCACCAGTGTCACCAAAGGGATGGCTGAGCTGAAATCCTTGGAAGAGCTGAACCTGGCATCGAACAGGCTGGCTGACCTGCCGGGGTGCAGTGGCTTTATGTCCCTGGAGTTCCTGAACGTAGAGATGAATTTGATCCTCACCCCATCTGCCGACTTCTTCCAGAGCTGCCTAAGGGTCAAGGAGCTACAAGCCGGGCACAACCCGTTCAAGTGTTCCTGTGAACTGCAAGACTTTATCCGTCTGGAGAGGCAGTCTGGGGTGAAGCTGTCTGACTGGCCGGAGGCATACGTG

>46153 TLR1B

TGGAATGCTCTTACTGAAGTTTTTCAGACTGTATGGCACTCATCCATTGAATACTTCAATATTAACAGACTAACACAATTGTCGGACATCAAAAGATATGACTTTGACTTTTCAGGTACCTCTATGAAAGCATTGACAATAAAGAAAGTTTTAATCACAGATCTGTACTTCTCACAGGATGACCTATACAAAATGTTTGCAGACATGAATATTGCAGCCTTGACAATAGCTGAATCAGAGATGATTCATATGCTGTGTCCTTTGTCTAACAGTCCCTTTAAATACTTAAATTTTTTAAAGAATGATTTAACAGATCTTCTTTTTCAAAATTGTGACAAATTAGTTCAACTGGATACATTAATCTTGCAGAAGAATAAATTTGAGAGCCTTTCCAAGGTAAGCTTCATGACCAGCCGTATGAAATTGCTGCAGTATTTGGACATGAGCAACAACTTGCTGAGTCACGATGCAGCTGATGTGCAATGCCGGTGGGCTGAGTCTCTGACAGAGTTGGACCTGTCCTCAAATCAGTTGACGGATTCCGTGTTTGAGTGCTTGCCAGTCAACATCAAAAAACTCAACCTACAAAACAATCAGATCACCAGTGTCACCAAAGGGATGGCTGAGCTGAAATCCTTGGAAGAGCTGAACCTGGCATCGAACAGGCTGGCTGACCTGCCGGAGTGCAGTGGCTTTATGTCCCTGGAGTTCCTGAACGTAGAGATGAATTTGATCCTCACCCCATCTGCCGACTTCTTCCAGAGCTGCCTAAGGGTCAAGGAGCTACAAGCCGGGCACAACCCGTTCAAGTGTTCCTGTGAACTGCAAGACTTTATCCGTCTGGAGAGGCAGTCTGGGGTGAAGCTGTCTGACTGGCCGGAGGCATACGTG

>46154 TLR1B

TGGGATGCTCTTACTGAAGTTTTTCAGACTGTATGGCACTCATCCATTGAATACTTCAATATTAACAGACTAACACAATTGTCGGACATCGAAAGATATGACTTTGACTATTCAGGTACCTCTATGAAAGCATTGACAATAAAGAAAGTTTTAATCACAGATCTGTACTTCTCACAGGATGACCTATACAAAATGTTTGCAGACATGAATATTGCAGCCTTGACAATAGCTGAATCAGAGATGATTCATATGCTGTGTCCTTTGTCTAACAGTCCCTTTAAATACTTAAATTTTTTAAAGAATGATTTAACAGATCTTCTTTTTCAAAATTGTGACAAATTAGTTCAACTGGATACATTAATCTTGCAGAAGAATAAATTTGAGAGCCTTTCCAAGGTAAGCTTCATGACCAGCCGTATGAAATTGCTGCAGTATTTGGACATGAGCAACAACTTGCTGAGTCACGATGCAGCTGATGTGCAATGCCGGTGGGCTGAGTCTCTGACAGAGTTGGACCTGTCCTCAAATCAGTTGACGGATTCCGTGTTTGAGTGCTTGCCAGTCAACATCAAAAAACTCAACCTACAAAACAATCAGATCACCAGTGTCACCAAAGGGATGGCTGAGCTGAAATCCTTGGAAGAGCTGAACCTGGCATCGAACAGGCTGGCTGACCTGCCGGAGTGCAGTGGCTTTATGTCCCTGGAGTTCCTGAACGTAGAGATGAATTTGATCCTCACCCCATCTGCCGACTTCTTCCAGAGCTGCCTAAGGGTCAAGGAGCTACAAGCCGGGCACAACCCGTTCAAGTGTTCCTGTGAACTGCAAGACTTTATCCGTCTGGAGAGGCAGTCTGGGGTGAAGCTGTCTGACTGGCCGGAGGCATACGTG

>46155 TLR1B

TGGAATGCTCTTACTGAAGTTTTTCAGACTGTATGGCACTCATCCATTGAATACTTCAATATTAACAGACTAACACAATTGTCGGACATCAAAAGATATGACTTTGACTATTCAGGTACCTCTATGAAAGCATTGACAATAAAGAAAGTTTTAATCACAGATCTGTACTTCTCACAGGATGACCTATACAAAATGTTTGCAGACATGAATATTGCAGCCTTGACAATAGCTGAATCAGAGATGATTCATATGCTGTGTCCTTTGTCTAACAGTCCCTTTAAATACTTAAATTTTTTAAAGAATGATTTAACAGATCTTCTTTTTCAAAATTGTGACAAATTAGTTCAACTGGATACATTAATCTTGCAGAAGAATAAATTTGAGAGCCTTTCCAAGGTAAGCTTCATGACCAGCCGTATGAAATTGCTGCAGTATTTGGACATGAGCAACAACTTGCTGAGTCACGATGCAGCTGATGTGCAATGCCGGTGGGCTGAGTCTCTGACAGAGTTGGACCTGTCCTCAAATCAGTTGACGGATTCCGTGTTTGAGTGCTTGCCAGTCAACATCAAAAAACTCAACCTACAAAACAATCAGATCACCAGTGTCACCAAAGGGATGGCTGAGCTGAAATCCTTGGAAGAGCTGAACCTGGCATCGAACAGGCTGGCTGACCTGCCGGAGTGCAGTGGCTTTATGTCCCTGGAGTTCCTGAACGTAGAGATGAATTTGATCCTCACCCCATCTGCCGACTTCTTCCAGAGCTGCCTAAGGGTCAAGGAGCTACAAGCCGGGCACAACCCGTTCAAGTGTTCCTGTGAACTGCAAGACTTTATCCGTCTGGAGAGGCAGTCTGGGGTGAAGCTGTCTGACTGGCCGGAGGCATACGTG

>46157 TLR1B

TGGGATGCTCTTACTGAAGTTTTTCAGACTGTATGGCACTCATCCATTGAATACTTCAATATTAACAGACTAACACAATTGTCGGACATCAAAAGATATGACTTTGACTATTCAGGTACCTCTATGAAAGCATTGACAATAAAGAAAGTTTTAATCACAGATCTGTACTTCTCACAGGATGACCTATACAAAATGTTTGCAGACATGAATATTGCAGCCTTGACAATAGCTGAATCAGAGATGATTCATATGCTGTGTCCTTTGTCTAACAGTCCCTTTAAATACTTAAATTTTTTAAAGAATGATTTAACAGATCTTCTTTTTCAAAATTGTGACAAATTAGTTCAACTGGATACATTAATCTTGCAGAAGAATAAATTTGAGAGCCTTTCCAAGGTAAGCTTCATGACCAGCCGTATGAAATTGCTGCAGTATTTGGACATGAGCAACAACTTGCTGAGTCACGATGCAGCTGATGTGCAATGCCGGTGGGCTGAGTCTCTGACAGAGTTGGACCTGTCCTCAAATCAGTTGACGGATTCCGTGTTTGAGTGCTTGCCAGTCAACATCAAAAAACTCAACCTACAAAACAATCAGATCACCAGTGTCACCAAAGGGATGGCTGAGCTGAAATCCTTGGAAGAGCTGAACCTGGCATCGAACAGGCTGGCTGACCTGCCGGAGTGCAGTGGCTTTATGTCCCTGGAGTTCCTGAACGTAGAGATGAATTTGATCCTCACCCCATCTGCCGACTTCTTCCAGAGCTGCCTAAGGGTCAAGGAGCTACAAGCCGGGCACAACCCGTTCAAGTGTTCCTGTGAACTGCAAGACTTTATCCGTCTGGAGAGGCAGTCTGGGGTGAAGCTGTCTGACTGGCCGGAGGCATACGTG

>46158 TLR1B

TGGAATGCTCTTACTGAAGTTTTTCAGACTGTATGGCACTCATCCATTGAATACTTCAATATTAACAGACTAACACAATTGTCGGACATCAAAAGATATGACTTTGACTATTCAGGTACCTCTATGAAAGCATTGACAATAAAGAAAGTTTTAATCACAGATCTGTACTTCTCACAGGATGACCTATACAAAATGTTTGCAGACATGAATATTGCAGCCTTGACAATAGCTGAATCAGAGATGATTCATATGCTGTGTCCTTTGTCTAACAGTCCCTTTAAATACTTAAATTTTTTAAAGAATGATTTAACAGATCTTCTTTTTCAAAATTGTGACAAATTAGTTCAACTGGATACATTAATCTTGCAGAAGAATAAATTTGAGAGCCTTTCCAAGGTAAGCTTCATGACCAGCCGTATGAAATTGCTGCAGTATTTGGACATGAGCAACAACTTGCTGAGTCACGATGCAGCTGATGTGCAATGCCGGTGGGCTGAGTCTCTGACAGAGTTGGACCTGTCCTCAAATCAGTTGACGGATTCCGTGTTTGAGTGCTTGCCAGTCAACATCAAAAAACTCAACCTACAAAACAATCAGATCACCAGTGTCACCAAAGGGATGGCTGAGCTGAAATCCTTGGAAGAGCTGAACCTGGCATCGAACAGGCTGGCTGACCTGCCGGAGTGCAGTGGCTTTATGTCCCTGGAGTTCCTGAACGTAGAGATGAATTTGATCCTCACCCCATCTGCCGACTTCTTCCAGAGCTGCCTAAGGGTCAAGGAGCTACAAGCCGGGCACAACCCGTTCAAGTGTTCCTGTGAACTGCAAGACTTTATCCGTCTGGAGAGGCAGTCTGGGGTGAAGCTGTCTGACTGGCCGGAGGCATACGTG

**TLR2**

>4156 TLR2

TGTAAATGGCCAAAAAACCTTAAATATTTAAACCTCTCCAGCACTCAAATTCCTAAACTAACAACTTGCATCCCTCCGACGCTGGAAGTTTTGGACGTCAGCGCTAACAACCTGATGGAGTTTGGACTGCAGCTCCCATTTCTCAAAGAGCTGTACCTTGCAAAAAACCAGCTGAAGACCTTGCCTGGTGCCGCACCCATTCCCAACTTAGTGGCCATGTCAGTCAGAAGAAACAAGCTCAACAGTTTCTCCAGGGAAGAGTTTGAGTCCTTCAAGAAACTGGAGCTGCTGGATGCCAGTGACAACAACTTCATCTGCTCCTGTGAATTCCTTTCCTTCATCCACCACCAGGCCGGGATAGCCCAGGTGCTGGTGGGGTGGCCAGACAAGTATGTCTGTGACTCTCCCCTGGCAGTGAGAGGGGTGCATGTTGGAGCTGTGCACCTCTCCCTGATGGAGTGCCACAAGTTCCTCGTGGTGTCGTTGATCTGCGCCCTGGTGTTCCTGGTCATCCTCATCCTCGTGGCCGTCGGCTACAAGTACCACGCGGTCTGGTACCTGAGA

>4161 TLR2

TGTAAATGGCCAAAAAACCTTAAATATTTAAACCTCTCCAGCACTCAAATTCCTAAACTAACAACTTGCATCCCTCCGACGCTGGAAGTTTTGGACGTCAGCGCTAACAACCTGATGGAGTTTGGACTGCAGCTCCCATTTCTCAAAGAGCTGTACCTTGCAAAAAACCAGCTGAAGACCTTGCCTGGTACCGCACCCATTCCCAACTTAGTGGCCATGTCAGTCAGAAGAAACAAGCTCAACAGTTTCTCCAGGGAAGAGTTTGAGTCCTTCAAGAAACTGGAGCTGCTGGATGCCAGTGACAAGAACTTCATCTGCTCCTGTGAATTCCTTTCCTTCATCCACCACCAGGCCGGGATAGCCCAGGTGCTGGTGGGGTGGCCAGACAAGTATGTCTGTGACTCTCCCCTGGCAGTGAGAGGGGTGCATGTTGGAGCTGTGCACCTCTCCCTGATGGAGTGCCACAAGTTCCTCGTGGTGTCGTTGATCTGCGCCCTGGTGTTCCTGGTCATCCTCATCCTCGTGGCCGTCGGCTACAAGTACCACGCGGTCTGGTACCTGAGA

>4177 TLR2

TGTAAATGGCCAAAAAACCTTAAATATTTAAACCTCTCCAGCACTCAAATTCCTAAACTAACAACTTGCATCCCTCCGACGCTGGAAGTTTTGGACGTCAGCGCTAACAACCTGATGGAGTTTGGACTGCAGCTCCCATTTCTCAAAGAGCTGTACCTTGCAAAAAACCAGCTGAAGACCTTGCCTGGTGCCGCACCCATTCCCAACTTAGTGGCCATGTCAGTCAGAAGAAACAAGCTCAACAGTTTCTCCAGGGAAGAGTTTGAGTCCTTCAAGAAACTGGAGCTGCTGGATGCCAGTGACAACAACTTCATCTGCTCCTGTGAATTCCTTTCCTTCATCCACCACCAGGCCGGGATAGCCCAGGTGCTGGTGGGGTGGCCAGACAAGTATGTCTGTGACTCTCCCCTGGCAGTGAGAGGGGTGCATGTTGGAGCTGTGCACCTCTCCCTGATGGAGTGCCACAAGTTCCTCGTGGTGTCGTTGATCTGCGCCCTGGTGTTCCTGGTCATCCTCATCCTCGTGGCCGTCGGCTACAAGTACCACGCGGTCTGGTACCTGAGA

>711 TLR2

TGTAAATGGCCAAAAAACCTTAAATATTTAAACCTCTCCAGCACTCAAATTCCTAAACTAACAACTTGCATCCCTCCGACGCTGGAAGTTTTGGACGTCAGCGCTAACAACCTGATGGAGTTTGGACTGCAGCTCCCATTTCTCAAAGAGCTGTACCTTGCAAAAAACCAGCTGAAGACCTTGCCTGGTGCCGCACCCATTCCCAACTTAGTGGCCATGTCAGTCAGAAGAAACAAGCTCAACAGTTTCTCCAGGGAAGAGTTTGAGTCCTTCAAGAAACTGGAGCTGCTGGATGCCAGTGACAACAACTTCATCTGCTCCTGTGAATTCCTTTCCTTCATCCACCACCAGGCCGGGATAGCCCAGGTGCTGGTGGGGTGGCCAGACAAGTATGTCTGTGACTCTCCCCTGGCAGTGAGAGGGGTGCATGTTGGAGCTGTGCACCTCTCCCTGATGGAGTGCCACAAGTTCCTCGTGGTGTCGTTGATCTGCGCCCTGGTGTTCCTGGTCATCCTCATCCTCGTGGCCGTCGGCTACAAGTACCACGCGGTCTGGTACCTGAGA

>713 TLR2

TGTAAATGGCCAAAAAACCTTAAATATTTAAACCTCTCCAGCACTCAAATTCCTAAACTAACAACTTGCATCCCTCCGACGCTGGAAGTTTTGGACGTCAGCGCTAACAACCTGATGGAGTTTGGACTGCAGCTCCCATTTCTCAAAGAGCTGTACCTTGCAAAAAACCAGCTGAAGACCTTGCCTGGTGCCGCACCCATTCCCAACTTAGTGGCCATGTCAGTCAGAAGAAACAAGCTCAACAGTTTCTCCAGGGAAGAGTTTGAGTCCTTCAAGAAACTGGAGCTGCTGGATGCCAGTGACAACAACTTCATCTGCTCCTGTGAATTCCTTTCCTTCATCCACCACCAGGCCGGGATAGCCCAGGTGCTGGTGGGGTGGCCAGACAAGTATGTCTGTGACTCTCCCCTGGCAGTGAGAGGGGTGCATGTTGGAGCTGTGCACCTCTCCCTGATGGAGTGCCACAAGTTCCTCGTGGTGTCGTTGATCTGCGCCCTGGTGTTCCTGGTCATCCTCATCCTCGTGGCCGTCGGCTACAAGTACCACGCGGTCTGGTACCTGAGA

>715 TLR2

TGTAAATGGCCAAAAAACCTTAAATATTTAAACCTCTCCAGCACTCAAATTCCTAAACTAACAACTTGCATCCCTCCGACGCTGGAAGTTTTGGACGTCAGCGCTAACAACCTGATGGAGTTTGGACTGCAGCTCCCATTTCTCAAAGAGCTGTACCTTGCAAAAAACCAGCTGAAGACCTTGCCTGGTGCCGCACCCATTCCCAACTTAGTGGCCATGTCAGTCAGAAGAAACAAGCTCAACAGTTTCTCCAGGGAAGAGTTTGAGTCCTTCAAGAAACTGGAGCTGCTGGATGCCAGTGACAACAACTTCATCTGCTCCTGTGAATTCCTTTCCTTCATCCACCACCAGGCCGGGATAGCCCAGGTGCTGGTGGGGTGGCCAGACAAGTATGTCTGTGACTCTCCCCTGGCAGTGAGAGGGGTGCATGTTGGAGCTGTGCACCTCTCCCTGATGGAGTGCCACAAGTTCCTCGTGGTGTCGTTGATCTGCGCCCTGGTGTTCCTGGTCATCCTCATCCTCGTGGCCGTCGGCTACAAGTACCACGCGGTCTGGTACCTGAGA

>822 TLR2

TGTAAATGGCCAAAAAACCTTAAATATTTAAACCTCTCCAGCACTCAAATTCCTAAACTAACAACTTGCATCCCTCCGACGCTGGAAGTTTTGGACGTCAGCGCTAACAACCTGATGGAGTTTGGACTGCAGCTCCCATTTCTCAAAGAGCTGTACCTTGCAAAAAACCAGCTGAAGACCTTGCCTGGTGCCGCACCCATTCCCAACTTAGTGGCCATGTCAGTCAGAAGAAACAAGCTCAACAGTTTCTCCAGGGAAGAGTTTGAGTCCTTCAAGAAACTGGAGCTGCTGGATGCCAGTGACAACAACTTCATCTGCTCCTGTGAATTCCTTTCCTTCATCCACCACCAGGCCGGGATAGCCCAGGTGCTGGTGGGGTGGCCAGACAAGTATGTCTGTGACTCTCCCCTGGCAGTGAGAGGGGTGCATGTTGGAGCTGTGCACCTCTCCCTGATGGAGTGCCACAAGTTCCTCGTGGTGTCGTTGATCTGCGCCCTGGTGTTCCTGGTCATCCTCATCCTCGTGGCCGTCGGCTACAAGTACCACGCGGTCTGGTACCTGAGA

>823 TLR2

TGTAAATGGCCAAAAAACCTTAAATATTTAAACCTCTCCAGCACTCAAATTCCTAAACTAACAACTTGCATCCCTCCGACGCTGGAAGTTTTGGACGTCAGCGCTAACAACCTGATGGAGTTTGGACTGCAGCTCCCATTTCTCAAAGAGCTGTACCTTGCAAAAAACCAGCTGAAGACCTTGCCTGGTGCCGCACCCATTCCCAACTTAGTGGCCATGTCAGTCAGAAGAAACAAGCTCAACAGTTTCTCCAGGGAAGAGTTTGAGTCCTTCAAGAAACTGGAGCTGCTGGATGCCAGTGACAACAACTTCATCTGCTCCTGTGAATTCCTTTCCTTCATCCACCACCAGGCCGGGATAGCCCAGGTGCTGGTGGGGTGGCCAGACAAGTATGTCTGTGACTCTCCCCTGGCAGTGAGAGGGGTGCATGTTGGAGCTGTGCACCTCTCCCTGATGGAGTGCCACAAGTTCCTCGTGGTGTCGTTGATCTGCGCCCTGGTGTTCCTGGTCATCCTCATCCTCGTGGCCGTCGGCTACAAGTACCACGCGGTCTGGTACCTGAGA

>826 TLR2

TGTAAATGGCCAAAAAACCTTAAATATTTAAACCTCTCCAGCACTCAAATTCCTAAACTAACAACTTGCATCCCTCCGACGCTGGAAGTTTTGGACGTCAGCGCTAACAACCTGATGGAGTTTGGACTGCAGCTCCCATTTCTCAAAGAGCTGTACCTTGCAAAAAACCAGCTGAAGACCTTGCCTGGTGCCGCACCCATTCCCAACTTAGTGGCCATGTCAGTCAGAAGAAACAAGCTCAACAGTTTCTCCAGGGAAGAGTTTGAGTCCTTCAAGAAACTGGAGCTGCTGGATGCCAGTGACAACAACTTCATCTGCTCCTGTGAATTCCTTTCCTTCATCCACCACCAGGCCGGGATAGCCCAGGTGCTGGTGGGGTGGCCAGACAAGTATGTCTGTGACTCTCCCCTGGCAGTGAGAGGGGTGCATGTTGGAGCTGTGCACCTCTCCCTGATGGAGTGCCACAAGTTCCTCGTGGTGTCGTTGATCTGCGCCCTGGTGTTCCTGGTCATCCTCATCCTCGTGGCCGTCGGCTACAAGTACCACGCGGTCTGGTACCTGAGA

>103 TLR2

TGTAAATGGCCAAAAAACCTTAAATATTTAAACCTCTCCAGCACTCAAATTCCTAAACTAACAACTTGCATCCCTCCGACGCTGGAAGTTTTGGACGTCAGCGCTAACAACCTGATGGAGTTTGGACTGCAGCTCCCATTTCTCAAAGAGCTGTACCTTGCAAAAAACCAGCTGAAGACCTTGCCTGGTGCCGCACCCATTCCCAACTTAGTGGCCATGTCAGTCAGAAGAAACAAGCTCAACAGTTTCTCCAGGGAAGAGTTTGAGTCCTTCAAGAAACTGGAGCTGCTGGATGCCAGTGACAACAACTTCATCTGCTCCTGTGAATTCCTTTCCTTCATCCACCACCAGGCCGGGATAGCCCAGGTGCTGGTGGGGTGGCCAGACAAGTATGTCTGTGACTCTCCCCTGGCAGTGAGAGGGGTGCATGTTGGAGCTGTGCACCTCTCCCTGATGGAGTGCCACAAGTTCCTCGTGGTGTCGTTGATCTGCGCCCTGGTGTTCCTGGTCATCCTCATCCTCGTGGCCGTCGGCTACAAGTACCACGCGGTCTGGTACCTGAGA

>1011 TLR2

TGTAAATGGCCAAAAAACCTTAAATATTTAAACCTCTCCAGCACTCAAATTCCTAAACTAACAACTTGCATCCCTCCGACGCTGGAAGTTTTGGACGTCAGCGCTAACAACCTGATGGAGTTTGGACTGCAGCTCCCATTTCTCAAAGAGCTGTACCTTGCAAAAAACCAGCTGAAGACCTTGCCTGGTGCCGCACCCATTCCCAACTTAGTGGCCATGTCAGTCAGAAGAAACAAGCTCAACAGTTTCTCCAGGGAAGAGTTTGAGTCCTTCAAGAAACTGGAGGTGCTGGATGCCAGTGACAACAACTTCATCTGCTCCTGTGAATTCCTTTCCTTCATCCACCACCAGGCCGGGATAGCCCAGGTGCTGGTGGGGTGGCCAGACAAGTATGTCTGTGACTCTCCCCTGGCAGTGAGAGGGGTGCATGTTGGAGCTGTGCACCTCTCCCTGATGGAGTGCCACAAGTTCCTCGTGGTGTCGTTGATCTGCGCCCTGGTGTTCCTGGTCATCCTCATCCTCGTGGCCGTCGGCTACAAGTACCACGCGGTCTGGTACCTGAGA

>1017 TLR2

TGTAAATGGCCAAAAAACCTTAAATATTTAAACCTCTCCAGCACTCAAATTCCTAAACTAACAACTTGCATCCCTCCGACGCTGGAAGTTTTGGACGTCAGCGCTAACAACCTGATGGAGTTTGGACTGCAGCTCCCATTTCTCAAAGAGCTGTACCTTGCAAAAAACCAGCTGAAGACCTTGCCTGGTGCCGCACCCATTCCCAACTTAGTGGCCATGTCAGTCAGAAGAAACAAGCTCAACAGTTTCTCCAGGGAAGAGTTTGAGTCCTTCAAGAAACTGGAGCTGCTGGATGCCAGTGACAACAACTTCATCTGCTCCTGTGAATTCCTTTCCTTCATCCACCACCAGGCCGGGATAGCCCAGGTGCTGGTGGGGTGGCCAGACAAGTATGTCTGTGACTCTCCCCTGGCAGTGAGAGGGGTGCATGTTGGAGCTGTGCACCTCTCCCTGATGGAGTGCCACAAGTTCCTCGTGGTGTCGTTGATCTGCGCCCTGGTGTTCCTGGTCATCCTCATCCTCGTGGCCGTCGGCTACAAGTACCACGCGGTCTGGTACCTGAGA

>1210 TLR2

TGTAAATGGCCAAAAAACCTTAAATATTTAAACCTCTCCAGCACTCAAATTCCTAAACTAACAACTTGCATCCCTCCGACGCTGGAAGTTTTGGACGTCAGCGCTAACAACCTGATGGAGTTTGGACTGCAGCTCCCATTTCTCAAAGAGCTGTACCTTGCAAAAAACCAGCTGAAGACCTTGCCTGGTGCCGCACCCATTCCCAACTTAGTGGCCATGTCAGTCAGAAGAAACAAGCTCAACAGTTTCTCCAGGGAAGAGTTTGAGTCCTTCAAGAAACTGGAGCTGCTGGATGCCAGTGACAACAACTTCATCTGCTCCTGTGAATTCCTTTCCTTCATCCACCACCAGGCCGGGATAGCCCAGGTGCTGGTGGGGTGGCCAGACAAGTATGTCTGTGACTCTCCCCTGGCAGTGAGAGGGGTGCATGTTGGAGCTGTGCACCTCTCCCTGATGGAGTGCCACAAGTTCCTCGTGGTGTCGTTGATCTGCGCCCTGGTGTTCCTGGTCATCCTCATCCTCGTGGCCGTCGGCTACAAGTACCACGCGGTCTGGTACCTGAGA

>1211 TLR2

TGTAAATGGCCAAAAAACCTTAAATATTTAAACCTCTCCAGCACTCAAATTCCTAAACTAACAACTTGCATCCCTCCGACGCTGGAAGTTTTGGACGTCAGCGCTAACAACCTGATGGAGTTTGGACTGCAGCTCCCATTTCTCAAAGAGCTGTACCTTGCAAAAAACCAGCTGAAGACCTTGCCTGGTGCCGCACCCATTCCCAACTTAGTGGCCATGTCAGTCAGAAGAAACAAGCTCAACAGTTTCTCCAGGGAAGAGTTTGAGTCCTTCAAGAAACTGGAGCTGCTGGATGCCAGTGACAACAACTTCATCTGCTCCTGTGAATTCCTTTCCTTCATCCACCACCAGGCCGGGATAGCCCAGGTGCTGGTGGGGTGGCCAGACAAGTATGTCTGTGACTCTCCCCTGGCAGTGAGAGGGGTGCATGTTGGAGCTGTGCACCTCTCCCTGATGGAGTGCCACAAGTTCCTCGTGGTGTCGTTGATCTGCGCCCTGGTGTTCCTGGTCATCCTCATCCTCGTGGCCGTCGGCTACAAGTACCACGCGGTCTGGTACCTGAGA

>1214 TLR2

TGTAAATGGCCAAAAAACCTTAAATATTTAAACCTCTCCAGCACTCAAATTCCTAAACTAACAACTTGCATCCCTCCGACGCTGGAAGTTTTGGACGTCAGCGCTAACAACCTGATGGAGTTTGGACTGCAGCTCCCATTTCTCAAAGAGCTGTACCTTGCAAAAAACCAGCTGAAGACCTTGCCTGGTGCCGCACCCATTCCCAACTTAGTGGCCATGTCAGTCAGAAGAAACAAGCTCAACAGTTTCTCCAGGGAAGAGTTTGAGTCCTTCAAGAAACTGGAGCTGCTGGATGCCAGTGACAACAACTTCATCTGCTCCTGTGAATTCCTTTCCTTCATCCACCACCAGGCCGGGATAGCCCAGGTGCTGGTGGGGTGGCCAGACAAGTATGTCTGTGACTCTCCCCTGGCAGTGAGAGGGGTGCATGTTGGAGCTGTGCACCTCTCCCTGATGGAGTGCCACAAGTTCCTCGTGGTGTCGTTGATCTGCGCCCTGGTGTTCCTGGTCATCCTCATCCTCGTGGCCGTCGGCTACAAGTACCACGCGGTCTGGTACCTGAGA

>169 TLR2

TGTAAATGGCCAAAAAACCTTAAATATTTAAACCTCTCCAGCACTCAAATTCCTAAACTAACAACTTGCATCCCTCCGACGCTGGAAGTTTTGGACGTCAGCGCTAACAACCTGATGGAGTTTGGACTGCAGCTCCCATTTCTCAAAGAGCTGTACCTTGCAAAAAACCAGCTGAAGACCTTGCCTGGTGCCGCACCCATTCCCAACTTAGTGGCCATGTCAGTCAGAAGAAACAAGCTCAACAGTTTCTCCAGGGAAGAGTTTGAGTCCTTCAAGAAACGGGAGCTGCTGGATGCCAGTGACAACAACTTCATCTGCTCCTGTGAATTCCTTTCCTTCATCCACCACCAGGCCGGGATAGCCCAGGTGCTGGTGGGGTGGCCAGACAAGTATGTCTGTGACTCTCCCCTGGCAGTGAGAGGGGTGCATGTTGGAGCTGTGCACCTCTCCCTGATGGAGTGCCACAAGTTCCTCGTGGTGTCGTTGATCTGCGCCCTGGTGTTCCTGGTCATCCTCATCCTCGTGGCCGTCGGCTACAAATACCACGCGGTCTGGTACCTGAGA

>1610 TLR2

TGTAAATGGCCAAAAAACCTTAAATATTTAAACCTCTCCAGCACTCAAATTCCTAAACTAACAACTTGCATCCCTCCGACGCTGGAAGTTTTGGACGTCAGCGCTAACAACCTGATGGAGTTTGGACTGCAGCTCCCATTTCTCAAAGAGCTGTACCTTGCAAAAAACCAGCTGAAGACCTTGCCTGGTGCCGCACCCATTCCCAACTTAGTGGCCATGTCAGTCAGAAGAAACAAGCTCAACAGTTTCTCCAGGGAAGAGTTTGAGTCCTTCAAGAAACGGGAGCTGCTGGATGCCAGTGACAACAACTTCATCTGCTCCTGTGAATTCCTTTCCTTCATCCACCACCAGGCCGGGATAGCCCAGGTGCTGGTGGGGTGGCCAGACAAGTATGTCTGTGACTCTCCCCTGGCAGTGAGAGGGGTGCATGTTGGAGCTGTGCACCTCTCCCTGATGGAGTGCCACAAGTTCCTCGTGGTGTCGTTGATCTGCGCCCTGGTGTTCCTGGTCATCCTCATCCTCGTGGCCGTCGGCTACAAATACCACGCGGTCTGGTACCTGAGA

>1616 TLR2

TGTAAATGGCCAAAAAACCTTAAATATTTAAACCTCTCCAGCACTCAAATTCCTAAACTAACAACTTGCATCCCTCCGACGCTGGAAGTTTTGGACGTCAGCGCTAACAACCTGATGGAGTTTGGACTGCAGCTCCCATTTCTCAAAGAGCTGTACCTTGCAAAAAACCAGCTGAAGACCTTGCCTGGTGCCGCACCCATTCCCAACTTAGTGGCCATGTCAGTCAGAAGAAACAAGCTCAACAGTTTCTCCAGGGAAGAGTTTGAGTCCTTCAAGAAACGGGAGCTGCTGGATGCCAGTGACAACAACTTCATCTGCTCCTGTGAATTCCTTTCCTTCATCCACCACCAGGCCGGGATAGCCCAGGTGCTGGTGGGGTGGCCAGACAAGTATGTCTGTGACTCTCCCCTGGCAGTGAGAGGGGTGCATGTTGGAGCTGTGCACCTCTCCCTGATGGAGTGCCACAAGTTCCTCGTGGTGTCGTTGATCTGCGCCCTGGTGTTCCTGGTCATCCTCATCCTCGTGGCCGTCGGCTACAAATACCACGCGGTCTGGTACCTGAGA

>175 TLR2

TGTAAATGGCCAAAAAACCTTAAATATTTAAACCTCTCCAGCACTCAAATTCCTAAACTAACAACTTGCATCCCTCCGACGCTGGAAGTTTTGGACGTCAGCGCTAACAACCTGATGGAGTTTGGACTGCAGCTCCCATTTCTCAAAGAGCTGTACCTTGCAAAAAACCAGCTGAAGACCTTGCCTGGTGCCGCACCCATTCCCAACTTAGTGGCCATGTCAGTCAGAAGAAACAAGCTCAACAGTTTCTCCAGGGAAGAGTTTGAGTCCTTCAAGAAACTGGAGCTGCTGGATGCCAGTGACAACAACTTCATCTGCTCCTGTGAATTCCTTTCCTTCATCCACCACCAGGCCGGGATAGCCCAGGTGCTGGTGGGGTGGCCAGACAAGTATGTCTGTGACTCTCCCCTGGCAGTGAGAGGGGTGCATGTTGGAGCTGTGCACCTCTCCCTGATGGAGTGCCACAAGTTCCTCGTGGTGTCGTTGATCTGCGCCCTGGTGTTCCTGGTCATCCTCATCCTCGTGGCCGTCGGCTACAAGTACCACGCGGTCTGGTACCTGAGA

>176 TLR2

TGTAAATGGCCAAAAAACCTTAAATATTTAAACCTCTCCAGCACTCAAATTCCTAAACTAACAACTTGCATCCCTCCGACGCTGGAAGTTTTGGACGTCAGCGCTAACAACCTGATGGAGTTTGGACTGCAGCTCCCATTTCTCAAAGAGCTGTACCTTGCAAAAAACCAGCTGAAGACCTTGCCTGGTGCCGCACCCATTCCCAACTTAGTGGCCATGTCAGTCAGAAGAAACAAGCTCAACAGTTTCTCCAGGGAAGAGTTTGAGTCCTTCAAGAAACTGGAGCTGCTGGATGCCAGTGACAACAACTTCATCTGCTCCTGTGAATTCCTTTCCTTCATCCACCACCAGGCCGGGATAGCCCAGGTGCTGGTGGGGTGGCCAGACAAGTATGTCTGTGACTCTCCCCTGGCAGTGAGAGGGGTGCATGTTGGAGCTGTGCACCTCTCCCTGATGGAGTGCCACAAGTTCCTCGTGGTGTCGTTGATCTGCGCCCTGGTGTTCCTGGTCATCCTCATCCTCGTGGCCGTCGGCTACAAGTACCACGCGGTCTGGTACCTGAGA

>1714 TLR2

TGTAAATGGCCAAAAAACCTTAAATATTTAAACCTCTCCAGCACTCAAATTCCTAAACTAACAACTTGCATCCCTCCGACGCTGGAAGTTTTGGACGTCAGCGCTAACAACCTGATGGAGTTTGGACTGCAGCTCCCATTTCTCAAAGAGCTGTACCTTGCAAAAAACCAGCTGAAGACCTTGCCTGGTGCCGCACCCATTCCCAACTTAGTGGCCATGTCAGTCAGAAGAAACAAGCTCAACAGTTTCTCCAGGGAAGAGTTTGAGTCCTTCAAGAAACTGGAGCTGCTGGATGCCAGTGACAACAACTTCATCTGCTCCTGTGAATTCCTTTCCTTCATCCACCACCAGGCCGGGATAGCCCAGGTGCTGGTGGGGTGGCCAGACAAGTATGTCTGTGACTCTCCCCTGGCAGTGAGAGGGGTGCATGTTGGAGCTGTGCACCTCTCCCTGATGGAGTGCCACAAGTTCCTCGTGGTGTCGTTGATCTGCGCCCTGGTGTTCCTGGTCATCCTCATCCTCGTGGCCGTCGGCTACAAGTACCACGCGGTCTGGTACCTGAGA

>30583 TLR2

TGTAAATGGCCAAAAAACCTTAAATATTTAAACCTCTCCAGCACTCAAATTCCTAAACTAACAACTTGCATCCCTCCGACGCTGGAAGTTTTGGACGTCAGCGCTAACAACCTGATGGAGTTTGGACTGCAGCTCCCATTTCTCAAAGAGCTGTACCTTGCAAAAAACCAGCTGAAGACCTTGCCTGGTGCCGCACCCATTCCCAACTTAGTGGCCATGTCAGTCAGAAGAAACAAGCTCAACAGTTTCTCCAGGGAAGAGTTTGAGTCCTTCAAGAAACTGGAGCTGCTGGATGCCAGTGACAACAACTTCATCTGCTCCTGTGAATTCCTTTCCTTCATCCACCACCAGGCCGGGATAGCCCAGGTGCTGGTGGGGTGGCCAGACAAGTATGTCTGTGACTCTCCCCTGGCAGTGAGAGGGGTGCATGTTGGAGCTGTGCACCTCTCCCTGATGGAGTGCCACAAGTTCCTCGTGGTGTCGTTGATCTGCGCCCTGGTGTTCCTGGTCATCCTCATCCTCGTGGCCGTCGGCTACAAGTACCACGCGGTCTGGTACCTGAGA

>30585 TLR2

TGTAAATGGCCAAAAAACCTTAAATATTTAAACCTCTCCAGCACTCAAATTCCTAAACTAACAACTTGCATCCCTCCGACGCTGGAAGTTTTGGACGTCAGCGCTAACAACCTGATGGAGTTTGGACTGCAGCTCCCATTTCTCAAAGAGCTGTACCTTGCAAAAAACCATCTGAAGACCTTGCCTGGTGCCGCACCCATTCCCAACTTAGTGGCCATGTCAGTCAGAAGAAACAAGCTCAACAGTTTCTCCAGGGAAGAGTTTGAGTCCTTCAAGAAACTGGAGCTGCTGGATGCCAGTGACAACAACTTCATCTGCTCCTGTGAATTCCTTTCCTTCATCCACCACCAGGCCGGGATAGCCCAGGTGCTGGTGGGGTGGCCAGACAAGTATGTCTGTGACTCTCCCCTGGCAGTGAGAGGGGTGCATGTTGGAGCTGTGCACCTCTCCCTGATGGAGTGCCACAAGTTCCTCGTGGTGTCGTTGATCTGCGCCCTGGTGTTCCTGGTCATCCTCATCCTCGTGGCCGTCGGCTACAAGTACCACGCGGTCTGGTACCTGAGA

>30586 TLR2

TGTAAATGGCCAAAAAACCTTAAATATTTAAACCTCTCCAGCACTCAAATTCCTAAACTAACAACTTGCATCCCTCCGACGCTGGAAGTTTTGGACGTCAGCGCTAACAACCTGATGGAGTTTGGACTGCAGCTCCCATTTCTCAAAGAGCTGTACCTTGCAAAAAACCAGCTGAAGACCTTGCCTGGTGCCGCACCCATTCCCAACTTAGTGGCCATGTCAGTCAGAAGAAACAAGCTCAACAGTTTCTCCAGGGAAGAGTTTGAGTCCTTCAAGAAACTGGAGCTGCTGGATGCCAGTGACAACAACTTCATCTGCTCCTGTGAATTCCTTTCCTTCATCCACCACCAGGCCGGGATAGCCCAGGTGCTGGTGGGGTGGCCAGACAAGTATGTCTGTGACTCTCCCCTGGCAGTGAGAGGGGTGCATGTTGGAGCTGTGCACCTCTCCCTGATGGAGTGCCACAAGTTCCTCGTGGTGTCGTTGATCTGCGCCCTGGTGTTCCTGGTCATCCTCATCCTCGTGGCCGTCGGCTACAAGTACCACGCGGTCTGGTACCTGAGA

>30588 TLR2

TGTAAATGGCCAAAAAACCTTAAATATTTAAACCTCTCCAGCACTCAAATTCCTAAACTAACAACTTGCATCCCTCCGACGCTGGAAGTTTTGGACGTCAGCGCTAACAACCTGATGGAGTTTGGACTGCAGCTCCCATTTCTCAAAGAGCTGTACCTTGCAAAAAACCAGCTGAAGACCTTGCCTGGTGCCGCACCCATTCCCAACTTAGTGGCCATGTCAGTCAGAAGAAACAAGCTCAACAGTTTCTCCAGGGAAGAGTTTGAGTCCTTCAAGAAACTGGAGCTGCTGGATGCCAGTGACAACAACTTCATCTGCTCCTGTGAATTCCTTTCCTTCATCCACCACCAGGCCGGGATAGCCCAGGTGCTGGTGGGGTGGCCAGACAAGTATGTCTGTGACTCTCCCCTGGCAGTGAGAGGGGTGCATGTTGGAGCTGTGCACCTCTCCCTGATGGAGTGCCACAAGTTCCTCGTGGTGTCGTTGATCTGCGCCCTGGTGTTCCTGGTCATCCTCATCCTCGTGGCCGTCGGCTACAAGTACCACGCGGTCTGGTACCTGAGA

>30589 TLR2

TGTAAATGGCCAAAAAACCTTAAATATTTAAACCTCTCCAGCACTCAAATTCCTAAACTAACAACTTGCATCCCTCCGACGCTGGAAGTTTTGGACGTCAGCGCTAACAACCTGATGGAGTTTGGACTGCAGCTCCCATTTCTCAAAGAGCTGTACCTTGCAAAAAACCAGCTGAAGACCTTGCCTGGTGCCGCACCCATTCCCAACTTAGTGGCCATGTCAGTCAGAAGAAACAAGCTCAACAGTTTCTCCAGGGAAGAGTTTGAGTCCTTCAAGAAACTGGAGCTGCTGGATGCCAGTGACAACAACTTCATCTGCTCCTGTGAATTCCTTTCCTTCATCCACCACCAGGCCGGGATAGCCCAGGTGCTGGTGGGGTGGCCAGACAAGTATGTCTGTGACTCTCCCCTGGCAGTGAGAGGGGTGCATGTTGGAGCTGTGCACCTCTCCCTGATGGAGTGCCACAAGTTCCTCGTGGTGTCGTTGATCTGCGCCCTGGTGTTCCTGGTCATCCTCATCCTCGTGGCCGTCGGCTACAAGTACCACGCGGTCTGGTACCTGAGA

>30590 TLR2

TGTAAATGGCCAAAAAACCTTAAATATTTAAACCTCTCCAGCACTCAAATTCCTAAACTAACAACTTGCATCCCTCCGACGCTGGAAGTTTTGGACGTCAGCGCTAACAACCTGATGGAGTTTGGACTGCAGCTCCCATTTCTCAAAGAGCTGTACCTTGCAAAAAACCAGCTGAAGACCTTGCCTGGTGCCGCACCCATTCCCAACTTAGTGGCCATGTCAGTCAGAAGAAACAAGCTCAACAGTTTCTCCAGGGAAGAGTTTGAGTCCTTCAAGAAACTGGAGCTGCTGGATGCCAGTGACAACAACTTCATCTGCTCCTGTGAATTCCTTTCCTTCATCCACCACCAGGCCGGGATAGCCCAGGTGCTGGTGGGGTGGCCAGACAAGTATGTCTGTGACTCTCCCCTGGCAGTGAGAGGGGTGCATGTTGGAGCTGTGCACCTCTCCCTGATGGAGTGCCACAAGTTCCTCGTGGTGTCGTTGATCTGCGCCCTGGTGTTCCTGGTCATCCTCATCCTCGTGGCCGTCGGCTACAAGTACCACGCGGTCTGGTACCTGAGA

>30591 TLR2

TGTAAATGGCCAAAAAACCTTAAATATTTAAACCTCTCCAGCACTCAAATTCCTAAACTAACAACTTGCATCCCTCCGACGCTGGAAGTTTTGGACGTCAGCGCTAACAACCTGATGGAGTTTGGACTGCAGCTCCCATTTCTCAAAGAGCTGTACCTTGCAAAAAACCAGCTGAAGACCTTGCCTGGTGCCGCACCCATTCCCAACTTAGTGGCCATGTCAGTCAGAAGAAACAAGCTCAACAGTTTCTCCAGGGAAGAGTTTGAGTCCTTCAAGAAACTGGAGCTGCCGGATGCCAGTGACAACAACTTCATCTGCTCCTGTGAATTCCTTTCCTTCATCCACCACCAGGCCGGGATAGCCCAGGTGCTGGTGGGGTGGCCAGACAAGTATGTCTGTGACTCTCCCCTGGCAGTGAGAGGGGTGCATGTTGGAGCTGTGCACCTCTCCCTGATGGAGTGCCACAAGTTCCTCGTGGTGTCGTTGATCTGCGCCCTGGTGTTCCTGGTCATCCTCATCCTCGTGGCCGTCGGCTACAAGTACCACGCGGTCTGGTACCTGAGA

>30703 TLR2

TGTAAATGGCCAAAAAACCTTAAATATTTAAACCTCTCCAGCACTCAAATTCCTAAACTAACAACTTGCATCCCTCCGACGCTGGAAGTTTTGGACGTCAGCGCTAACAACCTGATGGAGTTTGGACTGCAGCTCCCATTTCTCAAAGAGCTGTACCTTGCAAAAAACCATCTGAAGACCTTGCCTGGTGCCGCACCCATTCCCAACTTAGTGGCCATGTCAGTCAGAAGAAACAAGCTCAACAGTTTCTCCAGGGAAGAGTTTGAGTCCTTCAAGAAACTGGAGCTGCTGGATGCCAGTGACAACAACTTCATCTGCTCCTGTGAATTCCTTTCCTTCATCCACCACCAGGCCGGGATAGCCCAGGTGCTGGTGGGGTGGCCAGACAAGTATGTCTGTGACTCTCCCCTGGCAGTGAGAGGGGTGCATGTTGGAGCTGTGCACCTCTCCCTGATGGAGTGCCACAAGTTCCTCGTGGTGTCGTTGATCTGCGCCCTGGTGTTCCTGGTCATCCTCATCCTCGTGGCCGTCGGCTACATGTACCACGCGGTCTGGTACCTGAGA

>30704 TLR2

TGTAAATGGCCAAAAAACCTTAAATATTTAAACCTCTCCAGCACTCAAATTCCTAAACTAACAACTTGCATCCCTCCGACGCTGGAAGTTTTGGACGTCAGCGCTAACAACCTGATGGAGTTTGGACTGCAGCTCCCATTTCTCAAAGAGCTGTACCTTGCAAAAAACCAGCTGAAGACCTTGCCTGGTGCCGCACCCATTCCCAACTTAGTGGCCATGTCAGTCAGAAGAAACAAGCTCAACAGTTTCTCCAGGGAAGAGTTTGAGTCCTTCAAGAAACTGGAGCTGCTGGATGCCAGTGACAACAACTTCATCTGCTCCTGTGAATTCCTTTCCTTCATCCACCACCAGGCCGGGATAGCCCAGGTGCTGGTGGGGTGGCCAGACAAGTATGTCTGTGACTCTCCCCTGGCAGTGAGAGGGGTGCATGTTGGAGCTGTGCACCTCTCCCTGATGGAGTGCCACAAGTTCCTCGTGGTGTCGTTGATCTGCGCCCTGGTGTTCCTGGTCATCCTCATCCTCGTGGCCGTCGGCTACAAGTACCACGCGGTCTGGTACCTGAGA

>30706 TLR2

TGTAAATGGCCAAAAAACCTTAAATATTTAAACCTCTCCAGCACTCAAATTCCTAAACTAACAACTTGCATCCCTCCGACGCTGGAAGTTTTGGACGTCAGCGCTAACAACCTGATGGAGTTTGGACTGCAGCTCCCATTTCTCAAAGAGCTGTACCTTGCAAAAAACCAGCTGAAGACCTTGCCTGGTGCCGCACCCATTCCCAACTTAGTGGCCATGTCAGTCAGAAGAAACAAGCTCAACAGTTTCTCCAGGGAAGAGTTTGAGTCCTTCAAGAAACTGGAGCTGCTGGATGCCAGTGACAACAACTTCATCTGCTCCTGTGAATTCCTTTCCTTCATCCACCACCAGGCCGGGATAGCCCAGGTGCTGGTGGGGTGGCCAGACAAGTATGTCTGTGACTCTCCCCTGGCAGTGAGAGGGGTGCATGTTGGAGCTGTGCACCTCTCCCTGATGGAGTGCCACAAGTTCCTCGTGGTGTCGTTGATCTGCGCCCTGGTGTTCCTGGTCATCCTCATCCTCGTGGCCGTCGGCTACAAGTACCACGCGGTCTGGTACCTGAGA

>30708 TLR2

TGTAAATGGCCAAAAAACCTTAAATATTTAAACCTCTCCAGCACTCAAATTCCTAAACTAACAACTTGCATCCCTCCGACGCTGGAAGTTTTGGACGTCAGCGCTAACAACCTGATGGAGTTTGGACTGCAGCTCCCATTTCTCAAAGAGCTGTACCTTGCAAAAAACCAGCTGAAGACCTTGCCTGGTGCCGCACCCATTCCCAACTTAGTGGCCATGTCAGTCAGAAGAAACAAGCTCAACAGTTTCTCCAGGGAAGAGTTTGAGTCCTTCAAGAAACTGGAGCTGCTGGATGCCAGTGACAACAACTTCATCTGCTCCTGTGAATTCCTTTCCTTCATCCACCACCAGGCCGGGATAGCCCAGGTGCTGGTGGGGTGGCCAGACAAGTATGTCTGTGACTCTCCCCTGGCAGTGAGAGGGGTGCATGTTGGAGCTGTGCACCTCTCCCTGATGGAGTGCCACAAGTTCCTCGTGGTGTCGTTGATCTGCGCCCTGGTGTTCCTGGTCATCCTCATCCTCGTGGCCGTCGGCTACAAGTACCACGCGGTCTGGTACCTGAGA

>30710 TLR2

TGTAAATGGCCAAAAAACCTTAAATATTTAAACCTCTCCAGCACTCAAATTCCTAAACTAACAACTTGCATCCCTCCGACGCTGGAAGTTTTGGACGTCAGCGCTAACAACCTGATGGAGTTTGGACTGCAGCTCCCATTTCTCAAAGAGCTGTACCTTGCAAAAAACCAGCTGAAGACCTTGCCTGGTGCCGCACCCATTCCCAACTTAGTGGCCATGTCAGTCAGAAGAAACAAGCTCAACAGTTTCTCCAGGGAAGAGTTTGAGTCCTTCAAGAAACTGGAGCTGCTGGATGCCAGTGACAACAACTTCATCTGCTCCTGTGAATTCCTTTCCTTCATCCACCACCAGGCCGGGATAGCCCAGGTGCTGGTGGGGTGGCCAGACAAGTATGTCTGTGACTCTCCCCTGGCAGTGAGAGGGGTGCATGTTGGAGCTGTGCACCTCTCCCTGATGGAGTGCCACAAGTTCCTCGTGGTGTCGTTGATCTGCGCCCTGGTGTTCCTGGTCATCCTCATCCTCGTGGCCGTCGGCTACAAGTACCACGCGGTCTGGTACCTGAGA

>30711 TLR2

TGTAAATGGCCAAAAAACCTTAAATATTTAAACCTCTCCAGCACTCAAATTCCTAAACTAACAACTTGCATCCCTCCGACGCTGGAAGTTTTGGACGTCAGCGCTAACAACCTGATGGAGTTTGGACTGCAGCTCCCATTTCTCAAAGAGCTGTACCTTGCAAAAAACCAGCTGAAGACCTTGCCTGGTGCCGCACCCATTCCCAACTTAGTGGCCATGTCAGTCAGAAGAAACAAGCTCAACAGTTTCTCCAGGGAAGAGTTTGAGTCCTTCAAGAAACTGGAGCTGCTGGATGCCAGTGACAACAACTTCATCTGCTCCTGTGAATTCCTTTCCTTCATCCACCACCAGGCCGGGATAGCCCAGGTGCTGGTGGGGTGGCCAGACAAGTATGTCTGTGACTCTCCCCTGGCAGTGAGAGGGGTGCATGTTGGAGCTGTGCACCTCTCCCTGATGGAGTGCCACAAGTTCCTCGTGGTGTCGTTGATCTGCGCCCTGGTGTTCCTGGTCATCCTCATCCTCGTGGCCGTCGGCTACAAGTACCACGCGGTCTGGTACCTGAGA

>46151 TLR2

TGTAAATGGCCAAAAAACCTTAAATATTTAAACCTCTCCAGCACTCAAATTCCTAAACTAACAACTTGCATCCCTCCGACGCTGGAAGTTTTGGACGTCAGCGCTAACAACCTGATGGAGTTTGGACTGCAGCTCCCATTTCTCAAAGAGCTGTACCTTGCAAAAAACCAGCTGAAGACCTTGCCTGGTGCCGCACCCATTCCCAACTTAGTGGCCATGTCAGTCAGAAGAAACAAGCTCAACAGTTTCTCCAGGGAAGAGTTTGAGTCCTTCAAGAAACTGGAGCTGCTGGATGCCAGTGACAACAACTTCATCTGCTCCTGTGAATTCCTTTCCTTCATCCACCACCAGGCCGGGATAGCCCAGGTGCTGGTGGGGTGGCCAGACAAGTATGTCTGTGACTCTCCCCTGGCAGTGAGAGGGGTGCATGTTGGAGCTGTGCACCTCTCCCTGATGGAGTGCCACAAGTTCCTCGTGGTGTCGTTGATCTGCGCCCTGGTGTTCCTGGTCATCCTCATCCTCGTGGCCGTCGGCTACAAGTACCACGCGGTCTGGTACCTGAGA

>46152 TLR2

TGTAAATGGCCAAAAAACCTTAAATATTTAAACCTCTCCAGCACTCAAATTCCTAAACTAACAACTTGCATCCCTCCGACGCTGGAAGTTTTGGACGTCAGCGCTAACAACCTGATGGAGTTTGGACTGCAGCTCCCATTTCTCAAAGAGCTGTACCTTGCAAAAAACCAGCTGAAGACCTTGCCTGGTGCCGCACCCATTCCCAACTTAGTGGCCATGTCAGTCAGAAGAAACAAGCTCAACAGTTTCTCCAGGGAAGAGTTTGAGTCCTTCAAGAAACTGGAGCTGCTGGATGCCAGTGACAACAACTTCATCTGCTCCTGTGAATTCCTTTCCTTCATCCACCACCAGGCCGGGATAGCCCAGGTGCTGGTGGGGTGGCCAGACAAGTATGTCTGTGACTCTCCCCTGGCAGTGAGAGGGGTGCATGTTGGAGCTGTGCACCTCTCCCTGATGGAGTGCCACAAGTTCCTCGTGGTGTCGTTGATCTGCGCCCTGGTGTTCCTGGTCATCCTCATCCTCGTGGCCGTCGGCTACTAGTACCACGCGGTCTGGTACCTGAGA

>46153 TLR2

TGTAAATGGCCAAAAAACCTTAAATATTTAAACCTCTCCAGCACTCAAATTCCTAAACTAACAACTTGCATCCCTCCGACGCTGGAAGTTTTGGACGTCAGCGCTAACAACCTGATGGAGTTTGGACTGCAGCTCCCATTTCTCAAAGAGCTGTACCTTGCAAAAAACCAGCTGAAGACCTTGCCTGGTGCCGCACCCATTCCCAACTTAGTGGCCATGTCAGTCAGAAGAAACAAGCTCAACAGTTTCTCCAGGGAAGAGTTTGAGTCCTTCAAGAAACTGGAGCTGCTGGATGCCAGTGACAACAACTTCATCTGCTCCTGTGAATTCCTTTCCTTCATCCACCACCAGGCCGGGATAGCCCAGGTGCTGGTGGGGTGGCCAGACAAGTATGTCTGTGACTCTCCCCTGGCAGTGAGAGGGGTGCATGTTGGAGCTGTGCACCTCTCCCTGATGGAGTGCCACAAGTTCCTCGTGGTGTCGTTGATCTGCGCCCTGGTGTTCCTGGTCATCCTCATCCTCGTGGCCGTCGGCTACTAGTACCACGCGGTCTGGTACCTGAGA

>46154 TLR2

TGTAAATGGCCAAAAAACCTTAAATATTTAAACCTCTCCAGCACTCAAATTCCTAAACTAACAACTTGCATCCCTCCGACGCTGGAAGTTTTGGACGTCAGCGCTAACAACCTGATGGAGTTTGGACTGCAGCTCCCATTTCTCAAAGAGCTGTACCTTGCAAAAAACCAGCTGAAGACCTTGCCTGGTGCCGCACCCATTCCCAACTTAGTGGCCATGTCAGTCAGAAGAAACAAGCTCAACAGTTTCTCCAGGGAAGAGTTTGAGTCCTTCAAGAAACTGGAGCTGCTGGATGCCAGTGACAACAACTTCATCTGCTCCTGTGAATTCCTTTCCTTCATCCACCACCAGGCCGGGATAGCCCAGGTGCTGGTGGGGTGGCCAGACAAGTATGTCTGTGACTCTCCCCTGGCAGTGAGAGGGGTGCATGTTGGAGCTGTGCACCTCTCCCTGATGGAGTGCCACAAGTTCCTCGTGGTGTCGTTGATCTGCGCCCTGGTGTTCCTGGTCATCCTCATCCTCGTGGCCGTCGGCTACAAGTACCACGCGGTCTGGTACCTGAGA

>46155 TLR2

TGTAAATGGCCAAAAAACCTTAAATATTTAAACCTCTCCAGCACTCAAATTCCTAAACTAACAACTTGCATCCCTCCGACGCTGGAAGTTTTGGACGTCAGCGCTAACAACCTGATGGAGTTTGGACTGCAGCTCCCATTTCTCAAAGAGCTGTACCTTGCAAAAAACCAGCTGAAGACCTTGCCTGGTGCCGCACCCATTCCCAACTTAGTGGCCATGTCAGTCAGAAGAAACAAGCTCAACAGTTTCTCCAGGGAAGAGTTTGAGTCCTTCAAGAAACTGGAGCTGCTGGATGCCAGTGACAACAACTTCATCTGCTCCTGTGAATTCCTTTCCTTCATCCACCACCAGGCCGGGATAGCCCAGGTGCTGGTGGGGTGGCCAGACAAGTATGTCTGTGACTCTCCCCTGGCAGTGAGAGGGGTGCATGTTGGAGCTGTGCACCTCTCCCTGATGGAGTGCCACAAGTTCCTCGTGGTGTCGTTGATCTGCGCCCTGGTGTTCCTGGTCATCCTCATCCTCGTGGCCGTCGGCTACAAGTACCACGCGGTCTGGTACCTGAGA

>46157 TLR2

TGTAAATGGCCAAAAAACCTTAAATATTTAAACCTCTCCAGCACTCAAATTCCTAAACTAACAACTTGCATCCCTCCGACGCTGGAAGTTTTGGACGTCAGCGCTAACAACCTGATGGAGTTTGGACTGCAGCTCCCATTTCTCAAAGAGCTGTACCTTGCAAAAAACCAGCTGAAGACCTTGCCTGGTGCCGCACCCATTCCCAACTTAGTGGCCATGTCAGTCAGAAGAAACAAGCTCAACAGTTTCTCCAGGGAAGAGTTTGAGTCCTTCAAGAAACTGGAGCTGCTGGATGCCAGTGACAACAACTTCATCTGCTCCTGTGAATTCCTTTCCTTCATCCACCACCAGGCCGGGATAGCCCAGGTGCTGGTGGGGTGGCCAGACAAGTATGTCTGTGACTCTCCCCTGGCAGTGAGAGGGGTGCATGTTGGAGCTGTGCACCTCTCCCTGATGGAGTGCCACAAGTTCCTCGTGGTGTCGTTGATCTGCGCCCTGGTGTTCCTGGTCATCCTCATCCTCGTGGCCGTCGGCTACAAGTACCACGCGGTCTGGTACCTGAGA

>46158 TLR2

TGTAAATGGCCAAAAAACCTTAAATATTTAAACCTCTCCAGCACTCAAATTCCTAAACTAACAACTTGCATCCCTCCGACGCTGGAAGTTTTGGACGTCAGCGCTAACAACCTGATGGAGTTTGGACTGCAGCTCCCATTTCTCAAAGAGCTGTACCTTGCAAAAAACCAGCTGAAGACCTTGCCTGGTGCCGCACCCATTCCCAACTTAGTGGCCATGTCAGTCAGAAGAAACAAGCTCAACAGTTTCTCCAGGGAAGAGTTTGAGTCCTTCAAGAAACTGGAGCTGCTGGATGCCAGTGACAACAACTTCATCTGCTCCTGTGAATTCCTTTCCTTCATCCACCACCAGGCCGGGATAGCCCAGGTGCTGGTGGGGTGGCCAGACAAGTATGTCTGTGACTCTCCCCTGGCAGTGAGAGGGGTGCATGTTGGAGCTGTGCACCTCTCCCTGATGGAGTGCCACAAGTTCCTCGTGGTGTCGTTGATCTGCGCCCTGGTGTTCCTGGTCATCCTCATCCTCGTGGCCGTCGGCTACAAGTACCACGCGGTCTGGTACCTGAGA

**TLR5**

>4156 TLR5

AATCCTTTCAGAAATATTACGTTTAACTCACTAGACCTTAGTGACAATGGCTGGAGCACAGAGGAAGTCCAATATTTCTGTACAGCCATTAAAGGGACTCAAATCGGTTCTTTAATACTTAGCTCTCATACAATGGGTTCAGGATTTGGCTTTAATAACTTGAAAAATCCAGATAATGATACATTTGCAGGACTAGCAAGCAGTGATCTTCATTTGCTTGACATTTCAAATGGTTACATTTTCTCTCTCAATTCTTTAATCTTTCAAAGCCTTGGTAATCTGGAATTGCTGAACCTTTCCAAAAACAAGATAAATCAAATCCAAAGGCAAGCATTTTTTGGCTTGGGAAACCTAAAAACTCTCAATCTCTCAAGTAATCTTTTAGGTGAGTTGTACGATTATACTTTTGAAGGTCTACATAGCGTAATGTATATTGATTTACAGCAAAATCATATCGGGATGATTGGTGAAAAATCATTCAGTAACTTAGTAAGCCTGAAAATAATTGATCTCCGAGACAACGCCATTAAAAAACTCCCTTCCTTTCCACATCTGACCTCTGCCTTTTTAGGTGACAATAAGCTAATGTCTGTGGTTGGTACAGCAATAGCAGCAACACTCCTTGAATTAGAAAGAAATTGGTTGGCAAACCTGGGTGACCTGTATATTCTTTTCCAAGTTCCAGATGTGCAGTATATCTTCTTAAAACAGAATCGCTTCTCTTACTGTGTGAAACGTGATACTGTTACAGAAAACAACCAGTTAATCTATATGGATCTAGGTGAAAATATGTTACAGCTTGTGTGGGAGAGAGATTTATGTTTGGATGTGTTCAGGGCACTGTCCAAACTTCAGGTTCTGCATCTGAATAACAACTACCTTAGTGCTCTTCCACGGGAGATTTTTAGAGGTCTAACATCTCTGAAAAGACTTAATCTAGCTTCCAACCTATTGTCTCATCTTTCTCCTGCGCTTTTTCCACAGAGCCTAACAATCCTAAACTTATCTGGAAACCAGCTTTTTTCCCCTGAGCCTGAAGTCTTTATGACTTTGAGTATTCTGGATATAACACATAATAAGT

>4161 TLR5

AATCCTTTCAGAAATATTACGTTTAACTCACTAGACCTTAGTGACAATGGCTGGAGCACAGAGGAAGTCCAATATTTCTGTACAGCCATTAAAGGGACTCAAATCGGTTCTTTAATACTTAGCTCTCATACAATGGGTTCAGGATTTGGCTTTAATAACTTGAAAAATCCAGATAATGATACATTTGCAGGACTAGCAAGCAGTGATCTTCATTTGCTTGACATTTCAAATGGTTACATTTTCTCTCTCAATTCTTTAATCTTTCAAAGCCTTGGTAATCTGGAATTGCTGAACCTTTCCAAAAACAAGATAAATCAAATCCAAAGGCAAGCATTTTTTGGCTTGGGAAACCTAAAAACTCTCAATCTCTCAAGTAATCTTTTAGGTGAGTTGTACGATTATACTTTTGAAGGTCTACATAGCGTAATGTATATTGATTTACAGCAAAATCATATCGGGATGATTGGTGAAAAATCATTCAGTAACTTAGTAAGCCTGAAAATAATTGATCTCCGAGACAACGCCATTAAAAAACTCCCTTCCTTTCCACATCTGACCTCTGCCTTTTTAGGTGACAATAAGCTAATGTCTGTGGTTGGTACAGCAATAGCAGCAACACTCCTTGAATTAGAAAGAAATTGGTTGGCAAACCTGGGTGACCTGTATATTCTTTTCCAAGTTCCAGATGTGCAGTATATCTTCTTAAAACAGAATCGCTTCTCTTACTGTGTGAAACGTGATACTGTTACAGAAAACAACCAGTTAATCTATATGGATCTAGGTGAAAATATGTTACAGCTTGTGTGGGAGAGAGATTTATGTTTGGATGTGTTCAGGGCACTGTCCAAACTTCAGGTTCTGCATCTGAATAACAACTACCTTAGTGCTCTTCCACGGGAGATTTTTAGAGGTCTAACATCTCTGAAAAGACTTAATCTAGCTTCCAACCTATTGTCTCATCTTTCTCCTGCGCTTTTTCCACAGAGCCTAACAATCCTAAACTTATCTGGAAACCAGCTTTTTTCCCCTGAGCCTGAAGTCTTTATGACTTTGAGTATTCTGGATATAACACATAATAAGT

>4177 TLR5

AATCCTTTCAGAAATATTACGTTTAACTCACTAGACCTTAGTGACAATGGCTGGAGCACAGAGGAAGTCCAATATTTCTGTACAGCCATTAAAGGGACTCAAATCGGTTCTTTAATACTTAGCTCTCATACAATGGGTTCAGGATTTGGCTTTAATAACTTGAAAAATCCAGATAATGATACATTTGCAGGACTAGCAAGCAGTGATCTTCATTTGCTTGACATTTCAAATGGTTACATTTTCTCTCTCAATTCTTTAATCTTTCAAAGCCTTGGTAATCTGGAATTGCTGAACCTTTCCAAAAACAAGATAAATCAAATCCAAAGGCAAGCATTTTTTGGCTTGGGAAACCTAAAAACTCTCAATCTCTCAAGTAATCTTTTAGGTGAGTTGTACGATTATACTTTTGAAGGTCTACATAGCGTAATGTATATTGATTTACAGCAAAATCATATCGGGATGATTGGTGAAAAATCATTCAGTAACTTAGTAAGCCTGAAAATAATTGATCTCCGAGACAACGCCATTAAAAAACTCCCTTCCTTTCCACATCTGACCTCTGCCTTTTTAGGTGACAATAAGCTAATGTCTGTGGTTGGTACAGCAATAGCAGCAACACTCCTTGAATTAGAAAGAAATTGGTTGGCAAACCTGGGTGACCTGTATATTCTTTTCCAAGTTCCAGATGTGCAGTATATCTTCTTAAAACAGAATCGCTTCTCTTACTGTGTGAAACGTGATACTGTTACAGAAAACAACCAGTTAATCTATATGGATCTAGGTGAAAATATGTTACAGCTTGTGTGGGAGAGAGATTTATGTTTGGATGTGTTCAGGGCACTGTCCAAACTTCAGGTTCTGCATCTGAATAACAACTACCTTAGTGCTCTTCCACGGGAGATTTTTAGAGGTCTAACATCTCTGAAAAGACTTAATCTAGCTTCCAACCTATTGTCTCATCTTTCTCCTGCGCTTTTTCCACAGAGCCTAACAATCCTAAACTTATCTGGAAACCAGCTTTTTTCCCCTGAGCCTGAAGTCTTTATGACTTTGAGTATTCTGGATATAACACATAATAAGT

>711 TLR5

AATCCTTTCAGAAATATTACGTTTAACTCACTAGACCTTAGTGACAATGGCTGGAGCACAGAGGAAGTCCAATATTTCTGTACAGCCATTAAAGGGACTCAAATCGGTTCTTTAATACTTAGCTCTCATACAATGGGTTCAGGATTTGGCTTTAATAACTTGAAAAATCCAGATAATGATACATTTGCAGGACTAGCAAGCAGTGATCTTCATTTGCTTGACATTTCAAATGGTTACATTTTCTCTCTCAATTCTTTAATCTTTCAAAGCCTTGGTAATCTGGAATTGCTGAACCTTTCCAAAAACAAGATAAATCAAATCCAAAGGCAAGCATTTTTTGGCTTGGGAAACCTAAAAACTCTCAATCTCTCAAGTAATCTTTTAGGTGAGTTGTACGATTATACTTTTGAAGGTCTACATAGCGTAATGTATATTGATTTACAGCAAAATCATATCGGGATGATTGGTGAAAAATCATTCAGTAACTTAGTAAGCCTGAAAATAATTGATCTCCGAGACAACGCCATTAAAAAACTCCCTTCCTTTCCACATCTGACCTCTGCCTTTTTAGGTGACAATAAGCTAATGTCTGTGGTTGGTACAGCAATAGCAGCAACACTCCTTGAATTAGAAAGAAATTGGTTGGCAAACCTGGGTGACCTGTATATTCTTTTCCAAGTTCCAGATGTGCAGTATATCTTCTTAAAACAGAATCGCTTCTCTTACTGTGTGAAACGTGATACTGTTACAGAAAACAACCAGTTAATCTATATGGATCTAGGTGAAAATATGTTACAGCTTGTGTGGGAGAGAGATTTATGTTTGGATGTGTTCAGGGCACTGTCCAAACTTCAGGTTCTGCATCTGAATAACAACTACCTTAGTGCTCTTCCACGGGAGATTTTTAGAGGTCTAACATCTCTGAAAAGACTTAATCTAGCTTCCAACCTATTGTCTCATCTTTCTCCTGCGCTTTTTCCACAGAGCCTAACAATCCTAAACTTATCTGGAAACCAGCTTTTTTCCCCTGAGCCTGAAGTCTTTATGACTTTGAGTATTCTGGATATAACACATAATAAGT

>713 TLR5

AATCCTTTCAGAAATATTACGTTTAACTCACTAGACCTTAGTGACAATGGCTGGAGCACAGAGGAAGTCCAATATTTCTGTACAGCCATTAAAGGGACTCAAATCGGTTCTTTAATACTTAGCTCTCATACAATGGGTTCAGGATTTGGCTTTAATAACTTGAAAAATCCAGATAATGATACATTTGCAGGACTAGCAAGCAGTGATCTTCATTTGCTTGACATTTCAAATGGTTACATTTTCTCTCTCAATTCTTTAATCTTTCAAAGCCTTGGTAATCTGGAATTGCTGAACCTTTCCAAAAACAAGATAAATCAAATCCAAAGGCAAGCATTTTTTGGCTTGGGAAACCTAAAAACTCTCAATCTCTCAAGTAATCTTTTAGGTGAGTTGTACGATTATACTTTTGAAGGTCTACATAGCGTAATGTATATTGATTTACAGCAAAATCATATCGGGATGATTGGTGAAAAATCATTCAGTAACTTAGTAAGCCTGAAAATAATTGATCTCCGAGACAACGCCATTAAAAAACTCCCTTCCTTTCCACATCTGACCTCTGCCTTTTTAGGTGACAATAAGCTAATGTCTGTGGTTGGTACAGCAATAGCAGCAACACTCCTTGAATTAGAAAGAAATTGGTTGGCAAACCTGGGTGACCTGTATATTCTTTTCCAAGTTCCAGATGTGCAGTATATCTTCTTAAAACAGAATCGCTTCTCTTACTGTGTGAAACGTGATACTGTTACAGAAAACAACCAGTTAATCTATATGGATCTAGGTGAAAATATGTTACAGCTTGTGTGGGAGAGAGATTTATGTTTGGATGTGTTCAGGGCACTGTCCAAACTTCAGGTTCTGCATCTGAATAACAACTACCTTAGTGCTCTTCCACGGGAGATTTTTAGAGGTCTAACATCTCTGAAAAGACTTAATCTAGCTTCCAACCTATTGTCTCATCTTTCTCCTGCGCTTTTTCCACAGAGCCTAACAATCCTAAACTTATCTGGAAACCAGCTTTTTTCCCCTGAGCCTGAAGTCTTTATGACTTTGAGTATTCTGGATATAACACATAATAAGT

>715 TLR5

AATCCTTTCAGAAATATTACGTTTAACTCACTAGACCTTAGTGACAATGGCTGGAGCACAGAGGAAGTCCAATATTTCTGTACAGCCATTAAAGGGACTCAAATCGGTTCTTTAATACTTAGCTCTCATACAATGGGTTCAGGATTTGGCTTTAATAACTTGAAAAATCCAGATAATGATACATTTGCAGGACTAGCAAGCAGTGATCTTCATTTGCTTGACATTTCAAATGGTTACATTTTCTCTCTCAATTCTTTAATCTTTCAAAGCCTTGGTAATCTGGAATTGCTGAACCTTTCCAAAAACAAGATAAATCAAATCCAAAGGCAAGCATTTTTTGGCTTGGGAAACCTAAAAACTCTCAATCTCTCAAGTAATCTTTTAGGTGAGTTGTACGATTATACTTTTGAAGGTCTACATAGCGTAATGTATATTGATTTACAGCAAAATCATATCGGGATGATTGGTGAAAAATCATTCAGTAACTTAGTAAGCCTGAAAATAATTGATCTCCGAGACAACGCCATTAAAAAACTCCCTTCCTTTCCACATCTGACCTCTGCCTTTTTAGGTGACAATAAGCTAATGTCTGTGGTTGGTACAGCAATAGCAGCAACACTCCTTGAATTAGAAAGAAATTGGTTGGCAAACCTGGGTGACCTGTATATTCTTTTCCAAGTTCCAGATGTGCAGTATATCTTCTTAAAACAGAATCGCTTCTCTTACTGTGTGAAACGTGATACTGTTACAGAAAACAACCAGTTAATCTATATGGATCTAGGTGAAAATATGTTACAGCTTGTGTGGGAGAGAGATTTATGTTTGGATGTGTTCAGGGCACTGTCCAAACTTCAGGTTCTGCATCTGAATAACAACTACCTTAGTGCTCTTCCACGGGAGATTTTTAGAGGTCTAACATCTCTGAAAAGACTTAATCTAGCTTCCAACCTATTGTCTCATCTTTCTCCTGCGCTTTTTCCACAGAGCCTAACAATCCTAAACTTATCTGGAAACCAGCTTTTTTCCCCTGAGCCTGAAGTCTTTATGACTTTGAGTATTCTGGATATAACACATAATAAGT

>822 TLR5

AATCCTTTCAGAAATATTACGTTTAACTCACTAGACCTTAGTGACAATGGCTGGAGCACAGAGGAAGTCCAATATTTCTGTACAGCCATTAAAGGGACTCAAATCGGTTCTTTAATACTTAGCTCTCATACAATGGGTTCAGGATTTGGCTTTAATAACTTGAAAAATCCAGATAATGATACATTTGCAGGACTAGCAAGCAGTGATCTTCATTTGCTTGACATTTCAAATGGTTACATTTTCTCTCTCAATTCTTTAATCTTTCAAAGCCTTGGTAATCTGGAATTGCTGAACCTTTCCAAAAACAAGATAAATCAAATCCAAAGGCAAGCATTTTTTGGCTTGGGAAACCTAAAAACTCTCAATCTCTCAAGTAATCTTTTAGGTGAGTTGTACGATTATACTTTTGAAGGTCTACATAGCGTAATGTATATTGATTTACAGCAAAATCATATCGGGATGATTGGTGAAAAATCATTCAGTAACTTAGTAAGCCTGAAAATAATTGATCTCCGAGACAACGCCATTAAAAAACTCCCTTCCTTTCCACATCTGACCTCTGCCTTTTTAGGTGACAATAAGCTAATGTCTGTGGTTGGTACAGCAATAGCAGCAACACTCCTTGAATTAGAAAGAAATTGGTTGGCAAACCTGGGTGACCTGTATATTCTTTTCCAAGTTCCAGATGTGCAGTATATCTTCTTAAAACAGAATCGCTTCTCTTACTGTGTGAAACGTGATACTGTTACAGAAAACAACCAGTTAATCTATATGGATCTAGGTGAAAATATGTTACAGCTTGTGTGGGAGAGAGATTTATGTTTGGATGTGTTCAGGGCACTGTCCAAACTTCAGGTTCTGCATCTGAATAACAACTACCTTAGTGCTCTTCCACGGGAGATTTTTAGAGGTCTAACATCTCTGAAAAGACTTAATCTAGCTTCCAACCTATTGTCTCATCTTTCTCCTGCGCTTTTTCCACAGAGCCTAACAATCCTAAACTTATCTGGAAACCAGCTTTTTTCCCCTGAGCCTGAAGTCTTTATGACTTTGAGTATTCTGGATATAACACATAATAAGT

>823 TLR5

AATCCTTTCAGAAATATTACGTTTAACTCACTAGACCTTAGTGACAATGGCTGGAGCACAGAGGAAGTCCAATATTTCTGTACAGCCATTAAAGGGACTCAAATCGGTTCTTTAATACTTAGCTCTCATACAATGGGTTCAGGATTTGGCTTTAATAACTTGAAAAATCCAGATAATGATACATTTGCAGGACTAGCAAGCAGTGATCTTCATTTGCTTGACATTTCAAATGGTTACATTTTCTCTCTCAATTCTTTAATCTTTCAAAGCCTTGGTAATCTGGAATTGCTGAACCTTTCCAAAAACAAGATAAATCAAATCCAAAGGCAAGCATTTTTTGGCTTGGGAAACCTAAAAACTCTCAATCTCTCAAGTAATCTTTTAGGTGAGTTGTACGATTATACTTTTGAAGGTCTACATAGCGTAATGTATATTGATTTACAGCAAAATCATATCGGGATGATTGGTGAAAAATCATTCAGTAACTTAGTAAGCCTGAAAATAATTGATCTCCGAGACAACGCCATTAAAAAACTCCCTTCCTTTCCACATCTGACCTCTGCCTTTTTAGGTGACAATAAGCTAATGTCTGTGGTTGGTACAGCAATAGCAGCAACACTCCTTGAATTAGAAAGAAATTGGTTGGCAAACCTGGGTGACCTGTATATTCTTTTCCAAGTTCCAGATGTGCAGTATATCTTCTTAAAACAGAATCGCTTCTCTTACTGTGTGAAACGTGATACTGTTACAGAAAACAACCAGTTAATCTATATGGATCTAGGTGAAAATATGTTACAGCTTGTGTGGGAGAGAGATTTATGTTTGGATGTGTTCAGGGCACTGTCCAAACTTCAGGTTCTGCATCTGAATAACAACTACCTTAGTGCTCTTCCACGGGAGATTTTTAGAGGTCTAACATCTCTGAAAAGACTTAATCTAGCTTCCAACCTATTGTCTCATCTTTCTCCTGCGCTTTTTCCACAGAGCCTAACAATCCTAAACTTATCTGGAAACCAGCTTTTTTCCCCTGAGCCTGAAGTCTTTATGACTTTGAGTATTCTGGATATAACACATAATAAGT

>826 TLR5

AATCCTTTCAGAAATATTACGTTTAACTCACTAGACCTTAGTGACAATGGCTGGAGCACAGAGGAAGTCCAATATTTCTGTACAGCCATTAAAGGGACTCAAATCGGTTCTTTAATACTTAGCTCTCATACAATGGGTTCAGGATTTGGCTTTAATAACTTGAAAAATCCAGATAATGATACATTTGCAGGACTAGCAAGCAGTGATCTTCATTTGCTTGACATTTCAAATGGTTACATTTTCTCTCTCAATTCTTTAATCTTTCAAAGCCTTGGTAATCTGGAATTGCTGAACCTTTCCAAAAACAAGATAAATCAAATCCAAAGGCAAGCATTTTTTGGCTTGGGAAACCTAAAAACTCTCAATCTCTCAAGTAATCTTTTAGGTGAGTTGTACGATTATACTTTTGAAGGTCTACATAGCGTAATGTATATTGATTTACAGCAAAATCATATCGGGATGATTGGTGAAAAATCATTCAGTAACTTAGTAAGCCTGAAAATAATTGATCTCCGAGACAACGCCATTAAAAAACTCCCTTCCTTTCCACATCTGACCTCTGCCTTTTTAGGTGACAATAAGCTAATGTCTGTGGTTGGTACAGCAATAGCAGCAACACTCCTTGAATTAGAAAGAAATTGGTTGGCAAACCTGGGTGACCTGTATATTCTTTTCCAAGTTCCAGATGTGCAGTATATCTTCTTAAAACAGAATCGCTTCTCTTACTGTGTGAAACGTGATACTGTTACAGAAAACAACCAGTTAATCTATATGGATCTAGGTGAAAATATGTTACAGCTTGTGTGGGAGAGAGATTTATGTTTGGATGTGTTCAGGGCACTGTCCAAACTTCAGGTTCTGCATCTGAATAACAACTACCTTAGTGCTCTTCCACGGGAGATTTTTAGAGGTCTAACATCTCTGAAAAGACTTAATCTAGCTTCCAACCTATTGTCTCATCTTTCTCCTGCGCTTTTTCCACAGAGCCTAACAATCCTAAACTTATCTGGAAACCAGCTTTTTTCCCCTGAGCCTGAAGTCTTTATGACTTTGAGTATTCTGGATATAACACATAATAAGT

>103 TLR5

AATCCTTTCAGAAATATTACGTTTAACTCACTAGACCTTAGTGACAATGGCTGGAGCACAGAGGAAGTCCAATATTTCTGTACAGCCATTAAAGGGACTCAAATCGGTTCTTTAATACTTAGCTCTCATACAATGGGTTCAGGATTTGGCTTTAATAACTTGAAAAATCCAGATAATGATACATTTGCAGGACTAGCAAGCAGTGATCTTCATTTGCTTGACATTTCAAATGGTTACATTTTCTCTCTCAATTCTTTAATCTTTCAAAGCCTTGGTAATCTGGAATTGCTGAACCTTTCCAAAAACAAGATAAATCAAATCCAAAGGCAAGCATTTTTTGGCTTGGGAAACCTAAAAACTCTCAATCTCTCAAGTAATCTTTTAGGTGAGTTGTACGATTATACTTTTGAAGGTCTACATAGCGTAATGTATATTGATTTACAGCAAAATCATATCGGGATGATTGGTGAAAAATCATTCAGTAACTTAGTAAGCCTGAAAATAATTGATCTCCGAGACAACGCCATTAAAAAACTCCCTTCCTTTCCACATCTGACCTCTGCCTTTTTAGGTGACAATAAGCTAATGTCTGTGGTTGGTACAGCAATAGCAGCAACACTCCTTGAATTAGAAAGAAATTGGTTGGCAAACCTGGGTGACCTGTATATTCTTTTCCAAGTTCCAGATGTGCAGTATATCTTCTTAAAACAGAATCGCTTCTCTTACTGTGTGAAACGTGATACTGTTACAGAAAACAACCAGTTAATCTATATGGATCTAGGTGAAAATATGTTACAGCTTGTGTGGGAGAGAGATTTATGTTTGGATGTGTTCAGGGCACTGTCCAAACTTCAGGTTCCGCATCTGAATAACAACTACCTTAGTGCTCTTCCACGGGAGATTTTTAGAGGTCTAACATCTCTGAAAAGACTTAATCTAGCTTCCAACCTATTGTCTCATCTTTCTCCTGCGCTTTTTCCACAGAGCCTAACAATCCTAAACTTATCTGGAAACCAGCTTTTTTCCCCTGAGCCTGAAGTCTTTATGACTTTGAGTATTCTGGATATAACACATAATAAGT

>1011 TLR5

AATCCTTTCAGAAATATTACGTTTAACTCACTAGACCTTAGTGACAATGGCTGGAGCACAGAGGAAGTCCAATATTTCTGTACAGCCATTAAAGGGACTCAAATCGGTTCTTTAATACTTAGCTCTCATACAATGGGTTCAGGATTTGGCTTTAATAACTTGAAAAATCCAGATAATGATACATTTGCAGGACTAGCAAGCAGTGATCTTCATTTGCTTGACATTTCAAATGGTTACATTTTCTCTCTCAATTCTTTAATCTTTCAAAGCCTTGGTAATCTGGAATTGCTGAACCTTTCCAAAAACAAGATAAATCAAATCCAAAGGCAAGCATTTTTTGGCTTGGGAAACCTAAAAACTCTCAATCTCTCAAGTAATCTTTTAGGTGAGTTGTACGATTATACTTTTGAAGGTCTACATAGCGTAATGTATATTGATTTACAGCAAAATCATATCGGGATGATTGGTGAAAAATCATTCAGTAACTTAGTAAGCCTGAAAATAATTGATCTCCGAGACAACGCCATTAAAAAACTCCCTTCCTTTCCACATCTGACCTCTGCCTTTTTAGGTGACAATAAGCTAATGTCTGTGGTTGGTACAGCAATAGCAGCAACACTCCTTGAATTAGAAAGAAATTGGTTGGCAAACCTGGGTGACCTGTATATTCTTTTCCAAGTTCCAGATGTGCAGTATATCTTCTTAAAACAGAATCGCTTCTCTTACTGTGTGAAACGTGATACTGTTACAGAAAACAACCAGTTAATCTATATGGATCTAGGTGAAAATATGTTACAGCTTGTGTGGGAGAGAGATTTATGTTTGGATGTGTTCAGGGCACTGTCCAAACTTCAGGTTCTGCATCTGAATAACAACTACCTTAGTGCTCTTCCACGGGAGATTTTTAGAGGTCTAACATCTCTGAAAAGACTTAATCTAGCTTCCAACCTATTGTCTCATCTTTCTCCTGCGCTTTTTCCACAGAGCCTAACAATCCTAAACTTATCTGGAAACCAGCTTTTTTCCCCTGAGCCTGAAGTCTTTATGACTTTGAGTATTCTGGATATAACACATAATAAGT

>1017 TLR5

AATCCTTTCAGAAATATTACGTTTAACTCACTAGACCTTAGTGACAATGGCTGGAGCACAGAGGAAGTCCAATATTTCTGTACAGCCATTAAAGGGACTCAAATCGGTTCTTTAATACTTAGCTCTCATACAATGGGTTCAGGATTTGGCTTTAATAACTTGAAAAATCCAGATAATGATACATTTGCAGGACTAGCAAGCAGTGATCTTCATTTGCTTGACATTTCAAATGGTTACATTTTCTCTCTCAATTCTTTAATCTTTCAAAGCCTTGGTAATCTGGAATTGCTGAACCTTTCCAAAAACAAGATAAATCAAATCCAAAGGCAAGCATTTTTTGGCTTGGGAAACCTAAAAACTCTCAATCTCTCAAGTAATCTTTTAGGTGAGTTGTACGATTATACTTTTGAAGGTCTACATAGCGTAATGTATATTGATTTACAGCAAAATCATATCGGGATGATTGGTGAAAAATCATTCAGTAACTTAGTAAGCCTGAAAATAATTGATCTCCGAGACAACGCCATTAAAAAACTCCCTTCCTTTCCACATCTGACCTCTGCCTTTTTAGGTGACAATAAGCTAATGTCTGTGGTTGGTACAGCAATAGCAGCAACACTCCTTGAATTAGAAAGAAATTGGTTGGCAAACCTGGGTGACCTGTATATTCTTTTCCAAGTTCCAGATGTGCAGTATATCTTCTTAAAACAGAATCGCTTCTCTTACTGTGTGAAACGTGATACTGTTACAGAAAACAACCAGTTAATCTATATGGATCTAGGTGAAAATATGTTACAGCTTGTGTGGGAGAGAGATTTATGTTTGGATGTGTTCAGGGCACTGTCCAAACTTCAGGTTCTGCATCTGAATAACAACTACCTTAGTGCTCTTCCACGGGAGATTTTTAGAGGTCTAACATCTCTGAAAAGACTTAATCTAGCTTCCAACCTATTGTCTCATCTTTCTCCTGCGCTTTTTCCACAGAGCCTAACAATCCTAAACTTATCTGGAAACCAGCTTTTTTCCCCTGAGCCTGAAGTCTTTATGACTTTGAGTATTCTGGATATAACACATAATAAGT

>1210 TLR5

AATCCTTTCAGAAATATTACGTTTAACTCACTAGACCTTAGTGACAATGGCTGGAGCACAGAGGAAGTCCAATATTTCTGTACAGCCATTAAAGGGACTCAAATCGGTTCTTTAATACTTAGCTCTCATACAATGGGTTCAGGATTTGGCTTTAATAACTTGAAAAATCCAGATAATGATACATTTGCAGGACTAGCAAGCAGTGATCTTCATTTGCTTGACATTTCAAATGGTTACATTTTCTCTCTCAATTCTTTAATCTTTCAAAGCCTTGGTAATCTGGAATTGCTGAACCTTTCCAAAAACAAGATAAATCAAATCCAAAGGCAAGCATTTTTTGGCTTGGGAAACCTAAAAACTCTCAATCTCTCAAGTAATCTTTTAGGTGAGTTGTACGATTATACTTTTGAAGGTCTACATAGCGTAATGTATATTGATTTACAGCAAAATCATATCGGGATGATTGGTGAAAAATCATTCAGTAACTTAGTAAGCCTGAAAATAATTGATCTCCGAGACAACGCCATTAAAAAACTCCCTTCCTTTCCACATCTGACCTCTGCCTTTTTAGGTGACAATAAGCTAATGTCTGTGGTTGGTACAGCAATAGCAGCAACACTCCTTGAATTAGAAAGAAATTGGTTGGCAAACCTGGGTGACCTGTATATTCTTTTCCAAGTTCCAGATGTGCAGTATATCTTCTTAAAACAGAATCGCTTCTCTTACTGTGTGAAACGTGATACTGTTACAGAAAACAACCAGTTAATCTATATGGATCTAGGTGAAAATATGTTACAGCTTGTGTGGGAGAGAGATTTATGTTTGGATGTGTTCAGGGCACTGTCCAAACTTCAGGTTCTGCATCTGAATAACAACTACCTTAGTGCTCTTCCACGGGAGATTTTTAGAGGTCTAACATCTCTGAAAAGACTTAATCTAGCTTCCAACCTATTGTCTCATCTTTCTCCTGCGCTTTTTCCACAGAGCCTAACAATCCTAAACTTATCTGGAAACCAGCTTTTTTCCCCTGAGCCTGAAGTCTTTATGACTTTGAGTATTCTGGATATAACACATAATAAGT

>1211 TLR5

AATCCTTTCAGAAATATTACGTTTAACTCACTAGACCTTAGTGACAATGGCTGGAGCACAGAGGAAGTCCAATATTTCTGTACAGCCATTAAAGGGACTCAAATCGGTTCTTTAATACTTAGCTCTCATACAATGGGTTCAGGATTTGGCTTTAATAACTTGAAAAATCCAGATAATGATACATTTGCAGGACTAGCAAGCAGTGATCTTCATTTGCTTGACATTTCAAATGGTTACATTTTCTCTCTCAATTCTTTAATCTTTCAAAGCCTTGGTAATCTGGAATTGCTGAACCTTTCCAAAAACAAGATAAATCAAATCCAAAGGCAAGCATTTTTTGGCTTGGGAAACCTAAAAACTCTCAATCTCTCAAGTAATCTTTTAGGTGAGTTGTACGATTATACTTTTGAAGGTCTACATAGCGTAATGTATATTGATTTACAGCAAAATCATATCGGGATGATTGGTGAAAAATCATTCAGTAACTTAGTAAGCCTGAAAATAATTGATCTCCGAGACAACGCCATTAAAAAACTCCCTTCCTTTCCACATCTGACCTCTGCCTTTTTAGGTGACAATAAGCTAATGTCTGTGGTTGGTACAGCAATAGCAGCAACACTCCTTGAATTAGAAAGAAATTGGTTGGCAAACCTGGGTGACCTGTATATTCTTTTCCAAGTTCCAGATGTGCAGTATATCTTCTTAAAACAGAATCGCTTCTCTTACTGTGTGAAACGTGATACTGTTACAGAAAACAACCAGTTAATCTATATGGATCTAGGTGAAAATATGTTACAGCTTGTGTGGGAGAGAGATTTATGTTTGGATGTGTTCAGGGCACTGTCCAAACTTCAGGTTCCGCATCTGAATAACAACTACCTTAGTGCTCTTCCACGGGAGATTTTTAGAGGTCTAACATCTCTGAAAAGACTTAATCTAGCTTCCAACCTATTGTCTCATCTTTCTCCTGCGCTTTTTCCACAGAGCCTAACAATCCTAAACTTATCTGGAAACCAGCTTTTTTCCCCTGAGCCTGAAGTCTTTATGACTTTGAGTATTCTGGATATAACACATAATAAGT

>1214 TLR5

AATCCTTTCAGAAATATTACGTTTAACTCACTAGACCTTAGTGACAATGGCTGGAGCACAGAGGAAGTCCAATATTTCTGTACAGCCATTAAAGGGACTCAAATCGGTTCTTTAATACTTAGCTCTCATACAATGGGTTCAGGATTTGGCTTTAATAACTTGAAAAATCCAGATAATGATACATTTGCAGGACTAGCAAGCAGTGATCTTCATTTGCTTGACATTTCAAATGGTTACATTTTCTCTCTCAATTCTTTAATCTTTCAAAGCCTTGGTAATCTGGAATTGCTGAACCTTTCCAAAAACAAGATAAATCAAATCCAAAGGCAAGCATTTTTTGGCTTGGGAAACCTAAAAACTCTCAATCTCTCAAGTAATCTTTTAGGTGAGTTGTACGATTATACTTTTGAAGGTCTACATAGCGTAATGTATATTGATTTACAGCAAAATCATATCGGGATGATTGGTGAAAAATCATTCAGTAACTTAGTAAGCCTGAAAATAATTGATCTCCGAGACAACGCCATTAAAAAACTCCCTTCCTTTCCACATCTGACCTCTGCCTTTTTAGGTGACAATAAGCTAATGTCTGTGGTTGGTACAGCAATAGCAGCAACACTCCTTGAATTAGAAAGAAATTGGTTGGCAAACCTGGGTGACCTGTATATTCTTTTCCAAGTTCCAGATGTGCAGTATATCTTCTTAAAACAGAATCGCTTCTCTTACTGTGTGAAACGTGATACTGTTACAGAAAACAACCAGTTAATCTATATGGATCTAGGTGAAAATATGTTACAGCTTGTGTGGGAGAGAGATTTATGTTTGGATGTGTTCAGGGCACTGTCCAAACTTCAGGTTCTGCATCTGAATAACAACTACCTTAGTGCTCTTCCACGGGAGATTTTTAGAGGTCTAACATCTCTGAAAAGACTTAATCTAGCTTCCAACCTATTGTCTCATCTTTCTCCTGCGCTTTTTCCACAGAGCCTAACAATCCTAAACTTATCTGGAAACCAGCTTTTTTCCCCTGAGCCTGAAGTCTTTATGACTTTGAGTATTCTGGATATAACACATAATAAGT

>169 TLR5

AATCCTTTCAGAAATATTACGTTTAACTCACTAGACCTTAGTGACAATGGCTGGAGCACAGAGGAAGTCCAATATTTCTGTACAGCCATTAAAGGGACTCAAATCGGTTCTTTAATACTTAGCTCTCATACAATGGGTTCAGGATTTGGCTTTAATAACTTGAAAAATCCAGATAATGATACATTTGCAGGACTAGCAAGCAGTGATCTTCATTTGCTTGACATTTCAAATGGTTACATTTTCTCTCTCAATTCTTTAATCTTTCAAAGCCTTGGTAATCTGGAATTGCTGAACCTTTCCAAAAACAAGATAAATCAAATCCAAAGGCAAGCATTTTTTGGCTTGGGAAACCTAAAAACTCTCAATCTCTCAAGTAATCTTTTAGGTGAGTTGTACGATTATACTTTTGAAGGTCTACATAGCGTAATGTATATTGATTTACAGCAAAATCATATCGGGATGATTGGTGAAAAATCATTCAGTAACTTAGTAAGCCTGAAAATAATTGATCTCCGAGACAACGCCATTAAAAAACTCCCTTCCTTTCCACATCTGACCTCTGCCTTTTTAGGTGACAATAAGCTAATGTCTGTGGTTGGTACAGCAATAGCAGCAACACTCCTTGAATTAGAAAGAAATTGGTTGGCAAACCTGGGTGACCTGTATATTCTTTTCCAAGTTCCAGATGTGCAGTATATCTTCTTAAAACAGAATCGCTTCTCTTACTGTGTGAAACGTGATACTGTTACAGAAAACAACCAGTTAATCTATATGGATCTAGGTGAAAATATGTTACAGCTTGTGTGGGAGAGAGATTTATGTTTGGATGTGTTCAGGGCACTGTCCAAACTTCAGGTTCTGCATCTGAATAACAACTACCTTAGTGCTCTTCCACGGGAGATTTTTAGAGGTCTAACATCTCTGAAAAGACTTAATCTAGCTTCCAACCTATTGTCTCATCTTTCTCCTGCGCTTTTTCCACAGAGCCTAACAATCCTAAACTTATCTGGAAACCAGCTTTTTTCCCCTGAGCCTGAAGTCTTTATGACTTTGAGTATTCTGGATATAACACATAATAAGT

>1610 TLR5

AATCCTTTCAGAAATATTACGTTTAACTCACTAGACCTTAGTGACAATGGCTGGAGCACAGAGGAAGTCCAATATTTCTGTACAGCCATTAAAGGGACTCAAATCGGTTCTTTAATACTTAGCTCTCATACAATGGGTTCAGGATTTGGCTTTAATAACTTGAAAAATCCAGATAATGATACATTTGCAGGACTAGCAAGCAGTGATCTTCATTTGCTTGACATTTCAAATGGTTACATTTTCTCTCTCAATTCTTTAATCTTTCAAAGCCTTGGTAATCTGGAATTGCTGAACCTTTCCAAAAACAAGATAAATCAAATCCAAAGGCAAGCATTTTTTGGCTTGGGAAACCTAAAAACTCTCAATCTCTCAAGTAATCTTTTAGGTGAGTTGTACGATTATACTTTTGAAGGTCTACATAGCGTAATGTATATTGATTTACAGCAAAATCATATCGGGATGATTGGTGAAAAATCATTCAGTAACTTAGTAAGCCTGAAAATAATTGATCTCCGAGACAACGCCATTAAAAAACTCCCTTCCTTTCCACATCTGACCTCTGCCTTTTTAGGTGACAATAAGCTAATGTCTGTGGTTGGTACAGCAATAGCAGCAACACTCCTTGAATTAGAAAGAAATTGGTTGGCAAACCTGGGTGACCTGTATATTCTTTTCCAAGTTCCAGATGTGCAGTATATCTTCTTAAAACAGAATCGCTTCTCTTACTGTGTGAAACGTGATACTGTTACAGAAAACAACCAGTTAATCTATATGGATCTAGGTGAAAATATGTTACAGCTTGTGTGGGAGAGAGATTTATGTTTGGATGTGTTCAGGGCACTGTCCAAACTTCAGGTTCTGCATCTGAATAACAACTACCTTAGTGCTCTTCCACGGGAGATTTTTAGAGGTCTAACATCTCTGAAAAGACTTAATCTAGCTTCCAACCTATTGTCTCATCTTTCTCCTGCGCTTTTTCCACAGAGCCTAACAATCCTAAACTTATCTGGAAACCAGCTTTTTTCCCCTGAGCCTGAAGTCTTTATGACTTTGAGTATTCTGGATATAACACATAATAAGT

>1616 TLR5

AATCCTTTCAGAAATATTACGTTTAACTCACTAGACCTTAGTGACAATGGCTGGAGCACAGAGGAAGTCCAATATTTCTGTACAGCCATTAAAGGGACTCAAATCGGTTCTTTAATACTTAGCTCTCATACAATGGGTTCAGGATTTGGCTTTAATAACTTGAAAAATCCAGATAATGATACATTTGCAGGACTAGCAAGCAGTGATCTTCATTTGCTTGACATTTCAAATGGTTACATTTTCTCTCTCAATTCTTTAATCTTTCAAAGCCTTGGTAATCTGGAATTGCTGAACCTTTCCAAAAACAAGATAAATCAAATCCAAAGGCAAGCATTTTTTGGCTTGGGAAACCTAAAAACTCTCAATCTCTCAAGTAATCTTTTAGGTGAGTTGTACGATTATACTTTTGAAGGTCTACATAGCGTAATGTATATTGATTTACAGCAAAATCATATCGGGATGATTGGTGAAAAATCATTCAGTAACTTAGTAAGCCTGAAAATAATTGATCTCCGAGACAACGCCATTAAAAAACTCCCTTCCTTTCCACATCTGACCTCTGCCTTTTTAGGTGACAATAAGCTAATGTCTGTGGTTGGTACAGCAATAGCAGCAACACTCCTTGAATTAGAAAGAAATTGGTTGGCAAACCTGGGTGACCTGTATATTCTTTTCCAAGTTCCAGATGTGCAGTATATCTTCTTAAAACAGAATCGCTTCTCTTACTGTGTGAAACGTGATACTGTTACAGAAAACAACCAGTTAATCTATATGGATCTAGGTGAAAATATGTTACAGCTTGTGTGGGAGAGAGATTTATGTTTGGATGTGTTCAGGGCACTGTCCAAACTTCAGGTTCTGCATCTGAATAACAACTACCTTAGTGCTCTTCCACGGGAGATTTTTAGAGGTCTAACATCTCTGAAAAGACTTAATCTAGCTTCCAACCTATTGTCTCATCTTTCTCCTGCGCTTTTTCCACAGAGCCTAACAATCCTAAACTTATCTGGAAACCAGCTTTTTTCCCCTGAGCCTGAAGTCTTTATGACTTTGAGTATTCTGGATATAACACATAATAAGT

>175 TLR5

AATCCTTTCAGAAATATTACGTTTAACTCACTAGACCTTAGTGACAATGGCTGGAGCACAGAGGAAGTCCAATATTTCTGTACAGCCATTAAAGGGACTCAAATCGGTTCTTTAATACTTAGCTCTCATACAATGGGTTCAGGATTTGGCTTTAATAACTTGAAAAATCCAGATAATGATACATTTGCAGGACTAGCAAGCAGTGATCTTCATTTGCTTGACATTTCAAATGGTTACATTTTCTCTCTCAATTCTTTAATCTTTCAAAGCCTTGGTAATCTGGAATTGCTGAACCTTTCCAAAAACAAGATAAATCAAATCCAAAGGCAAGCATTTTTTGGCTTGGGAAACCTAAAAACTCTCAATCTCTCAAGTAATCTTTTAGGTGAGTTGTACGATTATACTTTTGAAGGTCTACATAGCGTAATGTATATTGATTTACAGCAAAATCATATCGGGATGATTGGTGAAAAATCATTCAGTAACTTAGTAAGCCTGAAAATAATTGATCTCCGAGACAACGCCATTAAAAAACTCCCTTCCTTTCCACATCTGACCTCTGCCTTTTTAGGTGACAATAAGCTAATGTCTGTGGTTGGTACAGCAATAGCAGCAACACTCCTTGAATTAGAAAGAAATTGGTTGGCAAACCTGGGTGACCTGTATATTCTTTTCCAAGTTCCAGATGTGCAGTATATCTTCTTAAAACAGAATCGCTTCTCTTACTGTGTGAAACGTGATACTGTTACAGAAAACAACCAGTTAATCTATATGGATCTAGGTGAAAATATGTTACAGCTTGTGTGGGAGAGAGATTTATGTTTGGATGTGTTCAGGGCACTGTCCAAACTTCAGGTTCTGCATCTGAATAACAACTACCTTAGTGCTCTTCCACGGGAGATTTTTAGAGGTCTAACATCTCTGAAAAGACTTAATCTAGCTTCCAACCTATTGTCTCATCTTTCTCCTGCGCTTTTTCCACAGAGCCTAACAATCCTAAACTTATCTGGAAACCAGCTTTTTTCCCCTGAGCCTGAAGTCTTTATGACTTTGAGTATTCTGGATATAACACATAATAAGT

>176 TLR5

AATCCTTTCAGAAATATTACGTTTAACTCACTAGACCTTAGTGACAATGGCTGGAGCACAGAGGAAGTCCAATATTTCTGTACAGCCATTAAAGGGACTCAAATCGGTTCTTTAATACTTAGCTCTCATACAATGGGTTCAGGATTTGGCTTTAATAACTTGAAAAATCCAGATAATGATACATTTGCAGGACTAGCAAGCAGTGATCTTCATTTGCTTGACATTTCAAATGGTTACATTTTCTCTCTCAATTCTTTAATCTTTCAAAGCCTTGGTAATCTGGAATTGCTGAACCTTTCCAAAAACAAGATAAATCAAATCCAAAGGCAAGCATTTTTTGGCTTGGGAAACCTAAAAACTCTCAATCTCTCAAGTAATCTTTTAGGTGAGTTGTACGATTATACTTTTGAAGGTCTACATAGCGTAATGTATATTGATTTACAGCAAAATCATATCGGGATGATTGGTGAAAAATCATTCAGTAACTTAGTAAGCCTGAAAATAATTGATCTCCGAGACAACGCCATTAAAAAACTCCCTTCCTTTCCACATCTGACCTCTGCCTTTTTAGGTGACAATAAGCTAATGTCTGTGGTTGGTACAGCAATAGCAGCAACACTCCTTGAATTAGAAAGAAATTGGTTGGCAAACCTGGGTGACCTGTATATTCTTTTCCAAGTTCCAGATGTGCAGTATATCTTCTTAAAACAGAATCGCTTCTCTTACTGTGTGAAACGTGATACTGTTACAGAAAACAACCAGTTAATCTATATGGATCTAGGTGAAAATATGTTACAGCTTGTGTGGGAGAGAGATTTATGTTTGGATGTGTTCAGGGCACTGTCCAAACTTCAGGTTCTGCATCTGAATAACAACTACCTTAGTGCTCTTCCACGGGAGATTTTTAGAGGTCTAACATCTCTGAAAAGACTTAATCTAGCTTCCAACCTATTGTCTCATCTTTCTCCTGCGCTTTTTCCACAGAGCCTAACAATCCTAAACTTATCTGGAAACCAGCTTTTTTCCCCTGAGCCTGAAGTCTTTATGACTTTGAGTATTCTGGATATAACACATAATAAGT

>1714 TLR5

AATCCTTTCAGAAATATTACGTTTAACTCACTAGACCTTAGTGACAATGGCTGGAGCACAGAGGAAGTCCAATATTTCTGTACAGCCATTAAAGGGACTCAAATCGGTTCTTTAATACTTAGCTCTCATACAATGGGTTCAGGATTTGGCTTTAATAACTTGAAAAATCCAGATAATGATACATTTGCAGGACTAGCAAGCAGTGATCTTCATTTGCTTGACATTTCAAATGGTTACATTTTCTCTCTCAATTCTTTAATCTTTCAAAGCCTTGGTAATCTGGAATTGCTGAACCTTTCCAAAAACAAGATAAATCAAATCCAAAGGCAAGCATTTTTTGGCTTGGGAAACCTAAAAACTCTCAATCTCTCAAGTAATCTTTTAGGTGAGTTGTACGATTATACTTTTGAAGGTCTACATAGCGTAATGTATATTGATTTACAGCAAAATCATATCGGGATGATTGGTGAAAAATCATTCAGTAACTTAGTAAGCCTGAAAATAATTGATCTCCGAGACAACGCCATTAAAAAACTCCCTTCCTTTCCACATCTGACCTCTGCCTTTTTAGGTGACAATAAGCTAATGTCTGTGGTTGGTACAGCAATAGCAGCAACACTCCTTGAATTAGAAAGAAATTGGTTGGCAAACCTGGGTGACCTGTATATTCTTTTCCAAGTTCCAGATGTGCAGTATATCTTCTTAAAACAGAATCGCTTCTCTTACTGTGTGAAACGTGATACTGTTACAGAAAACAACCAGTTAATCTATATGGATCTAGGTGAAAATATGTTACAGCTTGTGTGGGAGAGAGATTTATGTTTGGATGTGTTCAGGGCACTGTCCAAACTTCAGGTTCTGCATCTGAATAACAACTACCTTAGTGCTCTTCCACGGGAGATTTTTAGAGGTCTAACATCTCTGAAAAGACTTAATCTAGCTTCCAACCTATTGTCTCATCTTTCTCCTGCGCTTTTTCCACAGAGCCTAACAATCCTAAACTTATCTGGAAACCAGCTTTTTTCCCCTGAGCCTGAAGTCTTTATGACTTTGAGTATTCTGGATATAACACATAATAAGT

>30583 TLR5

AATCCTTTCAGAAATATTACGTTTAACTCACTAGACCTTAGTGACAATGGCTGGAGCACAGAGGAAGTCCAATATTTCTGTACAGCCATTAAAGGGACTCAAATCGGTTCTTTAATACTTAGCTCTCATACAATGGGTTCAGGATTTGGCTTTAATAACTTGAAAAATCCAGATAATGATACATTTGCAGGACTAGCAAGCAGTGATCTTCATTTGCTTGACATTTCAAATGGTTACATTTTCTCTCTCAATTCTTTAATCTTTCAAAGCCTTGGTAATCTGGAATTGCTGAACCTTTCCAAAAACAAGATAAATCAAATCCAAAGGCAAGCATTTTTTGGCTTGGGAAACCTAAAAACTCTCAATCTCTCAAGTAATCTTTTAGGTGAGTTGTACGATTATACTTTTGAAGGTCTACATAGCGTAATGTATATTGATTTACAGCAAAATCATATCGGGATGATTGGTGAAAAATCATTCAGTAACTTAGTAAGCCTGAAAATAATTGATCTCCGAGACAACGCCATTAAAAAACTCCCTTCCTTTCCACATCTGACCTCTGCCTTTTTAGGTGACAATAAGCTAATGTCTGTGGTTGGTACAGCAATAGCAGCAACACTCCTTGAATTAGAAAGAAATTGGTTGGCAAACCTGGGTGACCTGTATATTCTTTTCCAAGTTCCAGATGTGCAGTATATCTTCTTAAAACAGAATCGCTTCTCTTACTGTGTGAAACGTGATACTGTTACAGAAAACAACCAGTTAATCTATATGGATCTAGGTGAAAATATGTTACAGCTTGTGTGGGAGAGAGATTTATGTTTGGATGTGTTCAGGGCACTGTCCAAACTTCAGGTTCTGCATCTGAATAACAACTACCTTAGTGCTCTTCCACGGGAGATTTTTAGAGGTCTAACATCTCTGAAAAGACTTAATCTAGCTTCCAACCTATTGTCTCATCTTTCTCCTGCGCTTTTTCCACAGAGCCTAACAATCCTAAACTTATCTGGAAACCAGCTTTTTTCCCCTGAGCCTGAAGTCTTTATGACTTTGAGTATTCTGGATATAACACATAATAAGT

>30585 TLR5

AATCCTTTCAGAAATATTACGTTTAACTCACTAGACCTTAGTGACAATGGCTGGAGCACAGAGGAAGTCCAATATTTCTGTACAGCCATTAAAGGGACTCAAATCGGTTCTTTAATACTTAGCTCTCATACAATGGGTTCAGGATTTGGCTTTAATAACTTGAAAAATCCAGATAATGATACATTTGCAGGACTAGCAAGCAGTGATCTTCATTTGCTTGACATTTCAAATGGTTACATTTTCTCTCTCAATTCTTTAATCTTTCAAAGCCTTGGTAATCTGGAATTGCTGAACCTTTCCAAAAACAAGATAAATCAAATCCAAAGGCAAGCATTTTTTGGCTTGGGAAACCTAAAAACTCTCAATCTCTCAAGTAATCTTTTAGGTGAGTTGTACGATTATACTTTTGAAGGTCTACATAGCGTAATGTATATTGATTTACAGCAAAATCATATCGGGATGATTGGTGAAAAATCATTCAGTAACTTAGTAAGCCTGAAAATAATTGATCTCCGAGACAACGCCATTAAAAAACTCCCTTCCTTTCCACATCTGACCTCTGCCTTTTTAGGTGACAATAAGCTAATGTCTGTGGTTGGTACAGCAATAGCAGCAACACTCCTTGAATTAGAAAGAAATTGGTTGGCAAACCTGGGTGACCTGTATATTCTTTTCCAAGTTCCAGATGTGCAGTATATCTTCTTAAAACAGAATCGCTTCTCTTACTGTGTGAAACGTGATACTGTTACAGAAAACAACCAGTTAATCTATATGGATCTAGGTGAAAATATGTTACAGCTTGTGTGGGAGAGAGATTTATGTTTGGATGTGTTCAGGGCACTGTCCAAACTTCAGGTTCTGCATCTGAATAACAACTACCTTAGTGCTCTTCCACGGGAGATTTTTAGAGGTCTAACATCTCTGAAAAGACTTAATCTAGCTTCCAACCTATTGTCTCATCTTTCTCCTGCGCTTTTTCCACAGAGCCTAACAATCCTAAACTTATCTGGAAACCAGCTTTTTTCCCCTGAGCCTGAAGTCTTTATGACTTTGAGTATTCTGGATATAACACATAATAAGT

>30586 TLR5

AATCCTTTCAGAAATATTACGTTTAACTCACTAGACCTTAGTGACAATGGCTGGAGCACAGAGGAAGTCCAATATTTCTGTACAGCCATTAAAGGGACTCAAATCGGTTCTTTAATACTTAGCTCTCATACAATGGGTTCAGGATTTGGCTTTAATAACTTGAAAAATCCAGATAATGATACATTTGCAGGACTAGCAAGCAGTGATCTTCATTTGCTTGACATTTCAAATGGTTACATTTTCTCTCTCAATTCTTTAATCTTTCAAAGCCTTGGTAATCTGGAATTGCTGAACCTTTCCAAAAACAAGATAAATCAAATCCAAAGGCAAGCATTTTTTGGCTTGGGAAACCTAAAAACTCTCAATCTCTCAAGTAATCTTTTAGGTGAGTTGTACGATTATACTTTTGAAGGTCTACATAGCGTAATGTATATTGATTTACAGCAAAATCATATCGGGATGATTGGTGAAAAATCATTCAGTAACTTAGTAAGCCTGAAAATAATTGATCTCCGAGACAACGCCATTAAAAAACTCCCTTCCTTTCCACATCTGACCTCTGCCTTTTTAGGTGACAATAAGCTAATGTCTGTGGTTGGTACAGCAATAGCAGCAACACTCCTTGAATTAGAAAGAAATTGGTTGGCAAACCTGGGTGACCTGTATATTCTTTTCCAAGTTCCAGATGTGCAGTATATCTTCTTAAAACAGAATCGCTTCTCTTACTGTGTGAAACGTGATACTGTTACAGAAAACAACCAGTTAATCTATATGGATCTAGGTGAAAATATGTTACAGCTTGTGTGGGAGAGAGATTTATGTTTGGATGTGTTCAGGGCACTGTCCAAACTTCAGGTTCTGCATCTGAATAACAACTACCTTAGTGCTCTTCCACGGGAGATTTTTAGAGGTCTAACATCTCTGAAAAGACTTAATCTAGCTTCCAACCTATTGTCTCATCTTTCTCCTGCGCTTTTTCCACAGAGCCTAACAATCCTAAACTTATCTGGAAACCAGCTTTTTTCCCCTGAGCCTGAAGTCTTTATGACTTTGAGTATTCTGGATATAACACATAATAAGT

>30588 TLR5

AATCCTTTCAGAAATATTACGTTTAACTCACTAGACCTTAGTGACAATGGCTGGAGCACAGAGGAAGTCCAATATTTCTGTACAGCCATTAAAGGGACTCAAATCGGTTCTTTAATACTTAGCTCTCATACAATGGGTTCAGGATTTGGCTTTAATAACTTGAAAAATCCAGATAATGATACATTTGCAGGACTAGCAAGCAGTGATCTTCATTTGCTTGACATTTCAAATGGTTACATTTTCTCTCTCAATTCTTTAATCTTTCAAAGCCTTGGTAATCTGGAATTGCTGAACCTTTCCAAAAACAAGATAAATCAAATCCAAAGGCAAGCATTTTTTGGCTTGGGAAACCTAAAAACTCTCAATCTCTCAAGTAATCTTTTAGGTGAGTTGTACGATTATACTTTTGAAGGTCTACATAGCGTAATGTATATTGATTTACAGCAAAATCATATCGGGATGATTGGTGAAAAATCATTCAGTAACTTAGTAAGCCTGAAAATAATTGATCTCCGAGACAACGCCATTAAAAAACTCCCTTCCTTTCCACATCTGACCTCTGCCTTTTTAGGTGACAATAAGCTAATGTCTGTGGTTGGTACAGCAATAGCAGCAACACTCCTTGAATTAGAAAGAAATTGGTTGGCAAACCTGGGTGACCTGTATATTCTTTTCCAAGTTCCAGATGTGCAGTATATCTTCTTAAAACAGAATCGCTTCTCTTACTGTGTGAAACGTGATACTGTTACAGAAAACAACCAGTTAATCTATATGGATCTAGGTGAAAATATGTTACAGCTTGTGTGGGAGAGAGATTTATGTTTGGATGTGTTCAGGGCACTGTCCAAACTTCAGGTTCTGCATCTGAATAACAACTACCTTAGTGCTCTTCCACGGGAGATTTTTAGAGGTCTAACATCTCTGAAAAGACTTAATCTAGCTTCCAACCTATTGTCTCATCTTTCTCCTGCGCTTTTTCCACAGAGCCTAACAATCCTAAACTTATCTGGAAACCAGCTTTTTTCCCCTGAGCCTGAAGTCTTTATGACTTTGAGTATTCTGGATATAACACATAATAAGT

>30589 TLR5

AATCCTTTCAGAAATATTACGTTTAACTCACTAGACCTTAGTGACAATGGCTGGAGCACAGAGGAAGTCCAATATTTCTGTACAGCCATTAAAGGGACTCAAATCGGTTCTTTAATACTTAGCTCTCATACAATGGGTTCAGGATTTGGCTTTAATAACTTGAAAAATCCAGATAATGATACATTTGCAGGACTAGCAAGCAGTGATCTTCATTTGCTTGACATTTCAAATGGTTACATTTTCTCTCTCAATTCTTTAATCTTTCAAAGCCTTGGTAATCTGGAATTGCTGAACCTTTCCAAAAACAAGATAAATCAAATCCAAAGGCAAGCATTTTTTGGCTTGGGAAACCTAAAAACTCTCAATCTCTCAAGTAATCTTTTAGGTGAGTTGTACGATTATACTTTTGAAGGTCTACATAGCGTAATGTATATTGATTTACAGCAAAATCATATCGGGATGATTGGTGAAAAATCATTCAGTAACTTAGTAAGCCTGAAAATAATTGATCTCCGAGACAACGCCATTAAAAAACTCCCTTCCTTTCCACATCTGACCTCTGCCTTTTTAGGTGACAATAAGCTAATGTCTGTGGTTGGTACAGCAATAGCAGCAACACTCCTTGAATTAGAAAGAAATTGGTTGGCAAACCTGGGTGACCTGTATATTCTTTTCCAAGTTCCAGATGTGCAGTATATCTTCTTAAAACAGAATCGCTTCTCTTACTGTGTGAAACGTGATACTGTTACAGAAAACAACCAGTTAATCTATATGGATCTAGGTGAAAATATGTTACAGCTTGTGTGGGAGAGAGATTTATGTTTGGATGTGTTCAGGGCACTGTCCAAACTTCAGGTTCTGCATCTGAATAACAACTACCTTAGTGCTCTTCCACGGGAGATTTTTAGAGGTCTAACATCTCTGAAAAGACTTAATCTAGCTTCCAACCTATTGTCTCATCTTTCTCCTGCGCTTTTTCCACAGAGCCTAACAATCCTAAACTTATCTGGAAACCAGCTTTTTTCCCCTGAGCCTGAAGTCTTTATGACTTTGAGTATTCTGGATATAACACATAATAAGT

>30590 TLR5

AATCCTTTCAGAAATATTACGTTTAACTCACTAGACCTTAGTGACAATGGCTGGAGCACAGAGGAAGTCCAATATTTCTGTACAGCCATTAAAGGGACTCAAATCGGTTCTTTAATACTTAGCTCTCATACAATGGGTTCAGGATTTGGCTTTAATAACTTGAAAAATCCAGATAATGATACATTTGCAGGACTAGCAAGCAGTGATCTTCATTTGCTTGACATTTCAAATGGTTACATTTTCTCTCTCAATTCTTTAATCTTTCAAAGCCTTGGTAATCTGGAATTGCTGAACCTTTCCAAAAACAAGATAAATCAAATCCAAAGGCAAGCATTTTTTGGCTTGGGAAACCTAAAAACTCTCAATCTCTCAAGTAATCTTTTAGGTGAGTTGTACGATTATACTTTTGAAGGTCTACATAGCGTAATGTATATTGATTTACAGCAAAATCATATCGGGATGATTGGTGAAAAATCATTCAGTAACTTAGTAAGCCTGAAAATAATTGATCTCCGAGACAACGCCATTAAAAAACTCCCTTCCTTTCCACATCTGACCTCTGCCTTTTTAGGTGACAATAAGCTAATGTCTGTGGTTGGTACAGCAATAGCAGCAACACTCCTTGAATTAGAAAGAAATTGGTTGGCAAACCTGGGTGACCTGTATATTCTTTTCCAAGTTCCAGATGTGCAGTATATCTTCTTAAAACAGAATCGCTTCTCTTACTGTGTGAAACGTGATACTGTTACAGAAAACAACCAGTTAATCTATATGGATCTAGGTGAAAATATGTTACAGCTTGTGTGGGAGAGAGATTTATGTTTGGATGTGTTCAGGGCACTGTCCAAACTTCAGGTTCTGCATCTGAATAACAACTACCTTAGTGCTCTTCCACGGGAGATTTTTAGAGGTCTAACATCTCTGAAAAGACTTAATCTAGCTTCCAACCTATTGTCTCATCTTTCTCCTGCGCTTTTTCCACAGAGCCTAACAATCCTAAACTTATCTGGAAACCAGCTTTTTTCCCCTGAGCCTGAAGTCTTTATGACTTTGAGTATTCTGGATATAACACATAATAAGT

>30591 TLR5

AATCCTTTCAGAAATATTACGTTTAACTCACTAGACCTTAGTGACAATGGCTGGAGCACAGAGGAAGTCCAATATTTCTGTACAGCCATTAAAGGGACTCAAATCGGTTCTTTAATACTTAGCTCTCATACAATGGGTTCAGGATTTGGCTTTAATAACTTGAAAAATCCAGATAATGATACATTTGCAGGACTAGCAAGCAGTGATCTTCATTTGCTTGACATTTCAAATGGTTACATTTTCTCTCTCAATTCTTTAATCTTTCAAAGCCTTGGTAATCTGGAATTGCTGAACCTTTCCAAAAACAAGATAAATCAAATCCAAAGGCAAGCATTTTTTGGCTTGGGAAACCTAAAAACTCTCAATCTCTCAAGTAATCTTTTAGGTGAGTTGTACGATTATACTTTTGAAGGTCTACATAGCGTAATGTATATTGATTTACAGCAAAATCATATCGGGATGATTGGTGAAAAATCATTCAGTAACTTAGTAAGCCTGAAAATAATTGATCTCCGAGACAACGCCATTAAAAAACTCCCTTCCTTTCCACATCTGACCTCTGCCTTTTTAGGTGACAATAAGCTAATGTCTGTGGTTGGTACAGCAATAGCAGCAACACTCCTTGAATTAGAAAGAAATTGGTTGGCAAACCTGGGTGACCTGTATATTCTTTTCCAAGTTCCAGATGTGCAGTATATCTTCTTAAAACAGAATCGCTTCTCTTACTGTGTGAAACGTGATACTGTTACAGAAAACAACCAGTTAATCTATATGGATCTAGGTGAAAATATGTTACAGCTTGTGTGGGAGAGAGATTTATGTTTGGATGTGTTCAGGGCACTGTCCAAACTTCAGGTTCTGCATCTGAATAACAACTACCTTAGTGCTCTTCCACGGGAGATTTTTAGAGGTCTAACATCTCTGAAAAGACTTAATCTAGCTTCCAACCTATTGTCTCATCTTTCTCCTGCGCTTTTTCCACAGAGCCTAACAATCCTAAACTTATCTGGAAACCAGCTTTTTTCCCCTGAGCCTGAAGTCTTTATGACTTTGAGTATTCTGGATATAACACATAATAAGT

>30703 TLR5

AATCCTTTCAGAAATATTACGTTTAACTCACTAGACCTTAGTGACAATGGCTGGAGCACAGAGGAAGTCCAATATTTCTGTACAGCCATTAAAGGGACTCAAATCGGTTCTTTAATACTTAGCTCTCATACAATGGGTTCAGGATTTGGCTTTAATAACTTGAAAAATCCAGATAATGATACATTTGCAGGACTAGCAAGCAGTGATCTTCATTTGCTTGACATTTCAAATGGTTACATTTTCTCTCTCAATTCTTTAATCTTTCAAAGCCTTGGTAATCTGGAATTGCTGAACCTTTCCAAAAACAAGATAAATCAAATCCAAAGGCAAGCATTTTTTGGCTTGGGAAACCTAAAAACTCTCAATCTCTCAAGTAATCTTTTAGGTGAGTTGTACGATTATACTTTTGAAGGTCTACATAGCGTAATGTATATTGATTTACAGCAAAATCATATCGGGATGATTGGTGAAAAATCATTCAGTAACTTAGTAAGCCTGAAAATAATTGATCTCCGAGACAACGCCATTAAAAAACTCCCTTCCTTTCCACATCTGACCTCTGCCTTTTTAGGTGACAATAAGCTAATGTCTGTGGTTGGTACAGCAATAGCAGCAACACTCCTTGAATTAGAAAGAAATTGGTTGGCAAACCTGGGTGACCTGTATATTCTTTTCCAAGTTCCAGATGTGCAGTATATCTTCTTAAAACAGAATCGCTTCTCTTACTGTGTGAAACGTGATACTGTTACAGAAAACAACCAGTTAATCTATATGGATCTAGGTGAAAATATGTTACAGCTTGTGTGGGAGAGAGATTTATGTTTGGATGTGTTCAGGGCACTGTCCAAACTTCAGGTTCTGCATCTGAATAACAACTACCTTAGTGCTCTTCCACGGGAGATTTTTAGAGGTCTAACATCTCTGAAAAGACTTAATCTAGCTTCCAACCTATTGTCTCATCTTTCTCCTGCGCTTTTTCCACAGAGCCTAACAATCCTAAACTTATCTGGAAACCAGCTTTTTTCCCCTGAGCCTGAAGTCTTTATGACTTTGAGTATTCTGGATATAACACATAATAAGT

>30704 TLR5

AATCCTTTCAGAAATATTACGTTTAACTCACTAGACCTTAGTGACAATGGCTGGAGCACAGAGGAAGTCCAATATTTCTGTACAGCCATTAAAGGGACTCAAATCGGTTCTTTAATACTTAGCTCTCATACAATGGGTTCAGGATTTGGCTTTAATAACTTGAAAAATCCAGATAATGATACATTTGCAGGACTAGCAAGCAGTGATCTTCATTTGCTTGACATTTCAAATGGTTACATTTTCTCTCTCAATTCTTTAATCTTTCAAAGCCTTGGTAATCTGGAATTGCTGAACCTTTCCAAAAACAAGATAAATCAAATCCAAAGGCAAGCATTTTTTGGCTTGGGAAACCTAAAAACTCTCAATCTCTCAAGTAATCTTTTAGGTGAGTTGTACGATTATACTTTTGAAGGTCTACATAGCGTAATGTATATTGATTTACAGCAAAATCATATCGGGATGATTGGTGAAAAATCATTCAGTAACTTAGTAAGCCTGAAAATAATTGATCTCCGAGACAACGCCATTAAAAAACTCCCTTCCTTTCCACATCTGACCTCTGCCTTTTTAGGTGACAATAAGCTAATGTCTGTGGTTGGTACAGCAATAGCAGCAACACTCCTTGAATTAGAAAGAAATTGGTTGGCAAACCTGGGTGACCTGTATATTCTTTTCCAAGTTCCAGATGTGCAGTATATCTTCTTAAAACAGAATCGCTTCTCTTACTGTGTGAAACGTGATACTGTTACAGAAAACAACCAGTTAATCTATATGGATCTAGGTGAAAATATGTTACAGCTTGTGTGGGAGAGAGATTTATGTTTGGATGTGTTCAGGGCACTGTCCAAACTTCAGGTTCTGCATCTGAATAACAACTACCTTAGTGCTCTTCCACGGGAGATTTTTAGAGGTCTAACATCTCTGAAAAGACTTAATCTAGCTTCCAACCTATTGTCTCATCTTTCTCCTGCGCTTTTTCCACAGAGCCTAACAATCCTAAACTTATCTGGAAACCAGCTTTTTTCCCCTGAGCCTGAAGTCTTTATGACTTTGAGTATTCTGGATATAACACATAATAAGT

>30706 TLR5

AATCCTTTCAGAAATATTACGTTTAACTCACTAGACCTTAGTGACAATGGCTGGAGCACAGAGGAAGTCCAATATTTCTGTACAGCCATTAAAGGGACTCAAATCGGTTCTTTAATACTTAGCTCTCATACAATGGGTTCAGGATTTGGCTTTAATAACTTGAAAAATCCAGATAATGATACATTTGCAGGACTAGCAAGCAGTGATCTTCATTTGCTTGACATTTCAAATGGTTACATTTTCTCTCTCAATTCTTTAATCTTTCAAAGCCTTGGTAATCTGGAATTGCTGAACCTTTCCAAAAACAAGATAAATCAAATCCAAAGGCAAGCATTTTTTGGCTTGGGAAACCTAAAAACTCTCAATCTCTCAAGTAATCTTTTAGGTGAGTTGTACGATTATACTTTTGAAGGTCTACATAGCGTAATGTATATTGATTTACAGCAAAATCATATCGGGATGATTGGTGAAAAATCATTCAGTAACTTAGTAAGCCTGAAAATAATTGATCTCCGAGACAACGCCATTAAAAAACTCCCTTCCTTTCCACATCTGACCTCTGCCTTTTTAGGTGACAATAAGCTAATGTCTGTGGTTGGTACAGCAATAGCAGCAACACTCCTTGAATTAGAAAGAAATTGGTTGGCAAACCTGGGTGACCTGTATATTCTTTTCCAAGTTCCAGATGTGCAGTATATCTTCTTAAAACAGAATCGCTTCTCTTACTGTGTGAAACGTGATACTGTTACAGAAAACAACCAGTTAATCTATATGGATCTAGGTGAAAATATGTTACAGCTTGTGTGGGAGAGAGATTTATGTTTGGATGTGTTCAGGGCACTGTCCAAACTTCAGGTTCTGCATCTGAATAACAACTACCTTAGTGCTCTTCCACGGGAGATTTTTAGAGGTCTAACATCTCTGAAAAGACTTAATCTAGCTTCCAACCTATTGTCTCATCTTTCTCCTGCGCTTTTTCCACAGAGCCTAACAATCCTAAACTTATCTGGAAACCAGCTTTTTTCCCCTGAGCCTGAAGTCTTTATGACTTTGAGTATTCTGGATATAACACATAATAAGT

>30708 TLR5

AATCCTTTCAGAAATATTACGTTTAACTCACTAGACCTTAGTGACAATGGCTGGAGCACAGAGGAAGTCCAATATTTCTGTACAGCCATTAAAGGGACTCAAATCGGTTCTTTAATACTTAGCTCTCATACAATGGGTTCAGGATTTGGCTTTAATAACTTGAAAAATCCAGATAATGATACATTTGCAGGACTAGCAAGCAGTGATCTTCATTTGCTTGACATTTCAAATGGTTACATTTTCTCTCTCAATTCTTTAATCTTTCAAAGCCTTGGTAATCTGGAATTGCTGAACCTTTCCAAAAACAAGATAAATCAAATCCAAAGGCAAGCATTTTTTGGCTTGGGAAACCTAAAAACTCTCAATCTCTCAAGTAATCTTTTAGGTGAGTTGTACGATTATACTTTTGAAGGTCTACATAGCGTAATGTATATTGATTTACAGCAAAATCATATCGGGATGATTGGTGAAAAATCATTCAGTAACTTAGTAAGCCTGAAAATAATTGATCTCCGAGACAACGCCATTAAAAAACTCCCTTCCTTTCCACATCTGACCTCTGCCTTTTTAGGTGACAATAAGCTAATGTCTGTGGTTGGTACAGCAATAGCAGCAACACTCCTTGAATTAGAAAGAAATTGGTTGGCAAACCTGGGTGACCTGTATATTCTTTTCCAAGTTCCAGATGTGCAGTATATCTTCTTAAAACAGAATCGCTTCTCTTACTGTGTGAAACGTGATACTGTTACAGAAAACAACCAGTTAATCTATATGGATCTAGGTGAAAATATGTTACAGCTTGTGTGGGAGAGAGATTTATGTTTGGATGTGTTCAGGGCACTGTCCAAACTTCAGGTTCTGCATCTGAATAACAACTACCTTAGTGCTCTTCCACGGGAGATTTTTAGAGGTCTAACATCTCTGAAAAGACTTAATCTAGCTTCCAACCTATTGTCTCATCTTTCTCCTGCGCTTTTTCCACAGAGCCTAACAATCCTAAACTTATCTGGAAACCAGCTTTTTTCCCCTGAGCCTGAAGTCTTTATGACTTTGAGTATTCTGGATATAACACATAATAAGT

>30710 TLR5

AATCCTTTCAGAAATATTACGTTTAACTCACTAGACCTTAGTGACAATGGCTGGAGCACAGAGGAAGTCCAATATTTCTGTACAGCCATTAAAGGGACTCAAATCGGTTCTTTAATACTTAGCTCTCATACAATGGGTTCAGGATTTGGCTTTAATAACTTGAAAAATCCAGATAATGATACATTTGCAGGACTAGCAAGCAGTGATCTTCATTTGCTTGACATTTCAAATGGTTACATTTTCTCTCTCAATTCTTTAATCTTTCAAAGCCTTGGTAATCTGGAATTGCTGAACCTTTCCAAAAACAAGATAAATCAAATCCAAAGGCAAGCATTTTTTGGCTTGGGAAACCTAAAAACTCTCAATCTCTCAAGTAATCTTTTAGGTGAGTTGTACGATTATACTTTTGAAGGTCTACATAGCGTAATGTATATTGATTTACAGCAAAATCATATCGGGATGATTGGTGAAAAATCATTCAGTAACTTAGTAAGCCTGAAAATAATTGATCTCCGAGACAACGCCATTAAAAAACTCCCTTCCTTTCCACATCTGACCTCTGCCTTTTTAGGTGACAATAAGCTAATGTCTGTGGTTGGTACAGCAATAGCAGCAACACTCCTTGAATTAGAAAGAAATTGGTTGGCAAACCTGGGTGACCTGTATATTCTTTTCCAAGTTCCAGATGTGCAGTATATCTTCTTAAAACAGAATCGCTTCTCTTACTGTGTGAAACGTGATACTGTTACAGAAAACAACCAGTTAATCTATATGGATCTAGGTGAAAATATGTTACAGCTTGTGTGGGAGAGAGATTTATGTTTGGATGTGTTCAGGGCACTGTCCAAACTTCAGGTTCTGCATCTGAATAACAACTACCTTAGTGCTCTTCCACGGGAGATTTTTAGAGGTCTAACATCTCTGAAAAGACTTAATCTAGCTTCCAACCTATTGTCTCATCTTTCTCCTGCGCTTTTTCCACAGAGCCTAACAATCCTAAACTTATCTGGAAACCAGCTTTTTTCCCCTGAGCCTGAAGTCTTTATGACTTTGAGTATTCTGGATATAACACATAATAAGT

>30711 TLR5

AATCCTTTCAGAAATATTACGTTTAACTCACTAGACCTTAGTGACAATGGCTGGAGCACAGAGGAAGTCCAATATTTCTGTACAGCCATTAAAGGGACTCAAATCGGTTCTTTAATACTTAGCTCTCATACAATGGGTTCAGGATTTGGCTTTAATAACTTGAAAAATCCAGATAATGATACATTTGCAGGACTAGCAAGCAGTGATCTTCATTTGCTTGACATTTCAAATGGTTACATTTTCTCTCTCAATTCTTTAATCTTTCAAAGCCTTGGTAATCTGGAATTGCTGAACCTTTCCAAAAACAAGATAAATCAAATCCAAAGGCAAGCATTTTTTGGCTTGGGAAACCTAAAAACTCTCAATCTCTCAAGTAATCTTTTAGGTGAGTTGTACGATTATACTTTTGAAGGTCTACATAGCGTAATGTATATTGATTTACAGCAAAATCATATCGGGATGATTGGTGAAAAATCATTCAGTAACTTAGTAAGCCTGAAAATAATTGATCTCCGAGACAACGCCATTAAAAAACTCCCTTCCTTTCCACATCTGACCTCTGCCTTTTTAGGTGACAATAAGCTAATGTCTGTGGTTGGTACAGCAATAGCAGCAACACTCCTTGAATTAGAAAGAAATTGGTTGGCAAACCTGGGTGACCTGTATATTCTTTTCCAAGTTCCAGATGTGCAGTATATCTTCTTAAAACAGAATCGCTTCTCTTACTGTGTGAAACGTGATACTGTTACAGAAAACAACCAGTTAATCTATATGGATCTAGGTGAAAATATGTTACAGCTTGTGTGGGAGAGAGATTTATGTTTGGATGTGTTCAGGGCACTGTCCAAACTTCAGGTTCTGCATCTGAATAACAACTACCTTAGTGCTCTTCCACGGGAGATTTTTAGAGGTCTAACATCTCTGAAAAGACTTAATCTAGCTTCCAACCTATTGTCTCATCTTTCTCCTGCGCTTTTTCCACAGAGCCTAACAATCCTAAACTTATCTGGAAACCAGCTTTTTTCCCCTGAGCCTGAAGTCTTTATGACTTTGAGTATTCTGGATATAACACATAATAAGT

>46151 TLR5

AATCCTTTCAGAAATATTACGTTTAACTCACTAGACCTTAGTGACAATGGCTGGAGCACAGAGGAAGTCCAATATTTCTGTACAGCCATTAAAGGGACTCAAATCGGTTCTTTAATACTTAGCTCTCATACAATGGGTTCAGGATTTGGCTTTAATAACTTGAAAAATCCAGATAATGATACATTTGCAGGACTAGCAAGCAGTGATCTTCATTTGCTTGACATTTCAAATGGTTACATTTTCTCTCTCAATTCTTTAATCTTTCAAAGCCTTGGTAATCTGGAATTGCTGAACCTTTCCAAAAACAAGATAAATCAAATCCAAAGGCAAGCATTTTTTGGCTTGGGAAACCTAAAAACTCTCAATCTCTCAAGTAATCTTTTAGGTGAGTTGTACGATTATACTTTTGAAGGTCTACATAGCGTAATGTATATTGATTTACAGCAAAATCATATCGGGATGATTGGTGAAAAATCATTCAGTAACTTAGTAAGCCTGAAAATAATTGATCTCCGAGACAACGCCATTAAAAAACTCCCTTCCTTTCCACATCTGACCTCTGCCTTTTTAGGTGACAATAAGCTAATGTCTGTGGTTGGTACAGCAATAGCAGCAACACTCCTTGAATTAGAAAGAAATTGGTTGGCAAACCTGGGTGACCTGTATATTCTTTTCCAAGTTCCAGATGTGCAGTATATCTTCTTAAAACAGAATCGCTTCTCTTACTGTGTGAAACGTGATACTGTTACAGAAAACAACCAGTTAATCTATATGGATCTAGGTGAAAATATGTTACAGCTTGTGTGGGAGAGAGATTTATGTTTGGATGTGTTCAGGGCACTGTCCAAACTTCAGGTTCTGCATCTGAATAACAACTACCTTAGTGCTCTTCCACGGGAGATTTTTAGAGGTCTAACATCTCTGAAAAGACTTAATCTAGCTTCCAACCTATTGTCTCATCTTTCTCCTGCGCTTTTTCCACAGAGCCTAACAATCCTAAACTTATCTGGAAACCAGCTTTTTTCCCCTGAGCCTGAAGTCTTTATGACTTTGAGTATTCTGGATATAACACATAATAAGT

>46152 TLR5

AATCCTTTCAGAAATATTACGTTTAACTCACTAGACCTTAGTGACAATGGCTGGAGCACAGAGGAAGTCCAATATTTCTGTACAGCCATTAAAGGGACTCAAATCGGTTCTTTAATACTTAGCTCTCATACAATGGGTTCAGGATTTGGCTTTAATAACTTGAAAAATCCAGATAATGATACATTTGCAGGACTAGCAAGCAGTGATCTTCATTTGCTTGACATTTCAAATGGTTACATTTTCTCTCTCAATTCTTTAATCTTTCAAAGCCTTGGTAATCTGGAATTGCTGAACCTTTCCAAAAACAAGATAAATCAAATCCAAAGGCAAGCATTTTTTGGCTTGGGAAACCTAAAAACTCTCAATCTCTCAAGTAATCTTTTAGGTGAGTTGTACGATTATACTTTTGAAGGTCTACATAGCGTAATGTATATTGATTTACAGCAAAATCATATCGGGATGATTGGTGAAAAATCATTCAGTAACTTAGTAAGCCTGAAAATAATTGATCTCCGAGACAACGCCATTAAAAAACTCCCTTCCTTTCCACATCTGACCTCTGCCTTTTTAGGTGACAATAAGCTAATGTCTGTGGTTGGTACAGCAATAGCAGCAACACTCCTTGAATTAGAAAGAAATTGGTTGGCAAACCTGGGTGACCTGTATATTCTTTTCCAAGTTCCAGATGTGCAGTATATCTTCTTAAAACAGAATCGCTTCTCTTACTGTGTGAAACGTGATACTGTTACAGAAAACAACCAGTTAATCTATATGGATCTAGGTGAAAATATGTTACAGCTTGTGTGGGAGAGAGATTTATGTTTGGATGTGTTCAGGGCACTGTCCAAACTTCAGGTTCTGCATCTGAATAACAACTACCTTAGTGCTCTTCCACGGGAGATTTTTAGAGGTCTAACATCTCTGAAAAGACTTAATCTAGCTTCCAACCTATTGTCTCATCTTTCTCCTGCGCTTTTTCCACAGAGCCTAACAATCCTAAACTTATCTGGAAACCAGCTTTTTTCCCCTGAGCCTGAAGTCTTTATGACTTTGAGTATTCTGGATATAACACATAATAAGT

>46153 TLR5

AATCCTTTCAGAAATATTACGTTTAACTCACTAGACCTTAGTGACAATGGCTGGAGCACAGAGGAAGTCCAATATTTCTGTACAGCCATTAAAGGGACTCAAATCGGTTCTTTAATACTTAGCTCTCATACAATGGGTTCAGGATTTGGCTTTAATAACTTGAAAAATCCAGATAATGATACATTTGCAGGACTAGCAAGCAGTGATCTTCATTTGCTTGACATTTCAAATGGTTACATTTTCTCTCTCAATTCTTTAATCTTTCAAAGCCTTGGTAATCTGGAATTGCTGAACCTTTCCAAAAACAAGATAAATCAAATCCAAAGGCAAGCATTTTTTGGCTTGGGAAACCTAAAAACTCTCAATCTCTCAAGTAATCTTTTAGGTGAGTTGTACGATTATACTTTTGAAGGTCTACATAGCGTAATGTATATTGATTTACAGCAAAATCATATCGGGATGATTGGTGAAAAATCATTCAGTAACTTAGTAAGCCTGAAAATAATTGATCTCCGAGACAACGCCATTAAAAAACTCCCTTCCTTTCCACATCTGACCTCTGCCTTTTTAGGTGACAATAAGCTAATGTCTGTGGTTGGTACAGCAATAGCAGCAACACTCCTTGAATTAGAAAGAAATTGGTTGGCAAACCTGGGTGACCTGTATATTCTTTTCCAAGTTCCAGATGTGCAGTATATCTTCTTAAAACAGAATCGCTTCTCTTACTGTGTGAAACGTGATACTGTTACAGAAAACAACCAGTTAATCTATATGGATCTAGGTGAAAATATGTTACAGCTTGTGTGGGAGAGAGATTTATGTTTGGATGTGTTCAGGGCACTGTCCAAACTTCAGGTTCTGCATCTGAATAACAACTACCTTAGTGCTCTTCCACGGGAGATTTTTAGAGGTCTAACATCTCTGAAAAGACTTAATCTAGCTTCCAACCTATTGTCTCATCTTTCTCCTGCGCTTTTTCCACAGAGCCTAACAATCCTAAACTTATCTGGAAACCAGCTTTTTTCCCCTGAGCCTGAAGTCTTTATGACTTTGAGTATTCTGGATATAACACATAATAAGT

>46154 TLR5

AATCCTTTCAGAAATATTACGTTTAACTCACTAGACCTTAGTGACAATGGCTGGAGCACAGAGGAAGTCCAATATTTCTGTACAGCCATTAAAGGGACTCAAATCGGTTCTTTAATACTTAGCTCTCATACAATGGGTTCAGGATTTGGCTTTAATAACTTGAAAAATCCAGATAATGATACATTTGCAGGACTAGCAAGCAGTGATCTTCATTTGCTTGACATTTCAAATGGTTACATTTTCTCTCTCAATTCTTTAATCTTTCAACGCCTTGGTAATCTGGAATTGCTGAACCTTTCCAAAAACAAGATAAATCAAATCCAAAGGCAAGCATTTTTTGGCTTGGGAAACCTAAAAACTCTCAATCTCTCAAGTAATCTTTTAGGTGAGTTGTACGATTATACTTTTGAAGGTCTACATAGCGTAATGTATATTGATTTACAGCAAAATCATATCGGGATGATTGGTGAAAAATCATTCAGTAACTTAGTAAGCCTGAAAATAATTGATCTCCGAGACAACGCCATTAAAAAACTCCCTTCCTTTCCACATCTGACCTCTGCCTTTTTAGGTGACAATAAGCTAATGTCTGTGGTTGGTACAGCAATAGCAGCAACACTCCTTGAATTAGAAAGAAATTGGTTGGCAAACCTGGGTGACCTGTATATTCTTTTCCAAGTTCCAGATGTGCAGTATATCTTCTTAAAACAGAATCGCTTCTCTTACTGTGTGAAACGTGATACTGTTACAGAAAACAACCAGTTAATCTATATGGATCTAGGTGAAAATATGTTACAGCTTGTGTGGGAGAGAGATTTATGTTTGGATGTGTTCAGGGCACTGTCCAAACTTCAGGTTCTGCATCTGAATAACAACTACCTTAGTGCTCTTCCACGGGAGATTTTTAGAGGTCTAACATCTCTGAAAAGACTTAATCTAGCTTCCAACCTATTGTCTCATCTTTCTCCTGCGCTTTTTCCACAGAGCCTAACAATCCTAAACTTATCTGGAAACCAGCTTTTTTCCCCTGAGCCTGAAGTCTTTATGACTTTGAGTATTCTGGATATAACACATAATAAGT

>46155 TLR5

AATCCTTTCAGAAATATTACGTTTAACTCACTAGACCTTAGTGACAATGGCTGGAGCACAGAGGAAGTCCAATATTTCTGTACAGCCATTAAAGGGACTCAAATCGGTTCTTTAATACTTAGCTCTCATACAATGGGTTCAGGATTTGGCTTTAATAACTTGAAAAATCCAGATAATGATACATTTGCAGGACTAGCAAGCAGTGATCTTCATTTGCTTGACATTTCAAATGGTTACATTTTCTCTCTCAATTCTTTAATCTTTCAACGCCTTGGTAATCTGGAATTGCTGAACCTTTCCAAAAACAAGATAAATCAAATCCAAAGGCAAGCATTTTTTGGCTTGGGAAACCTAAAAACTCTCAATCTCTCAAGTAATCTTTTAGGTGAGTTGTACGATTATACTTTTGAAGGTCTACATAGCGTAATGTATATTGATTTACAGCAAAATCATATCGGGATGATTGGTGAAAAATCATTCAGTAACTTAGTAAGCCTGAAAATAATTGATCTCCGAGACAACGCCATTAAAAAACTCCCTTCCTTTCCACATCTGACCTCTGCCTTTTTAGGTGACAATAAGCTAATGTCTGTGGTTGGTACAGCAATAGCAGCAACACTCCTTGAATTAGAAAGAAATTGGTTGGCAAACCTGGGTGACCTGTATATTCTTTTCCAAGTTCCAGATGTGCAGTATATCTTCTTAAAACAGAATCGCTTCTCTTACTGTGTGAAACGTGATACTGTTACAGAAAACAACCAGTTAATCTATATGGATCTAGGTGAAAATATGTTACAGCTTGTGTGGGAGAGAGATTTATGTTTGGATGTGTTCAGGGCACTGTCCAAACTTCAGGTTCTGCATCTGAATAACAACTACCTTAGTGCTCTTCCACGGGAGATTTTTAGAGGTCTAACATCTCTGAAAAGACTTAATCTAGCTTCCAACCTATTGTCTCATCTTTCTCCTGCGCTTTTTCCACAGAGCCTAACAATCCTAAACTTATCTGGAAACCAGCTTTTTTCCCCTGAGCCTGAAGTCTTTATGACTTTGAGTATTCTGGATATAACACATAATAAGT

>46157 TLR5

AATCCTTTCAGAAATATTACGTTTAACTCACTAGACCTTAGTGACAATGGCTGGAGCACAGAGGAAGTCCAATATTTCTGTACAGCCATTAAAGGGACTCAAATCGGTTCTTTAATACTTAGCTCTCATACAATGGGTTCAGGATTTGGCTTTAATAACTTGAAAAATCCAGATAATGATACATTTGCAGGACTAGCAAGCAGTGATCTTCATTTGCTTGACATTTCAAATGGTTACATTTTCTCTCTCAATTCTTTAATCTTTCAAAGCCTTGGTAATCTGGAATTGCTGAACCTTTCCAAAAACAAGATAAATCAAATCCAAAGGCAAGCATTTTTTGGCTTGGGAAACCTAAAAACTCTCAATCTCTCAAGTAATCTTTTAGGTGAGTTGTACGATTATACTTTTGAAGGTCTACATAGCGTAATGTATATTGATTTACAGCAAAATCATATCGGGATGATTGGTGAAAAATCATTCAGTAACTTAGTAAGCCTGAAAATAATTGATCTCCGAGACAACGCCATTAAAAAACTCCCTTCCTTTCCACATCTGACCTCTGCCTTTTTAGGTGACAATAAGCTAATGTCTGTGGTTGGTACAGCAATAGCAGCAACACTCCTTGAATTAGAAAGAAATTGGTTGGCAAACCTGGGTGACCTGTATATTCTTTTCCAAGTTCCAGATGTGCAGTATATCTTCTTAAAACAGAATCGCTTCTCTTACTGTGTGAAACGTGATACTGTTACAGAAAACAACCAGTTAATCTATATGGATCTAGGTGAAAATATGTTACAGCTTGTGTGGGAGAGAGATTTATGTTTGGATGTGTTCAGGGCACTGTCCAAACTTCAGGTTCTGCATCTGAATAACAACTACCTTAGTGCTCTTCCACGGGAGATTTTTAGAGGTCTAACATCTCTGAAAAGACTTAATCTAGCTTCCAACCTATTGTCTCATCTTTCTCCTGCGCTTTTTCCACAGAGCCTAACAATCCTAAACTTATCTGGAAACCAGCTTTTTTCCCCTGAGCCTGAAGTCTTTATGACTTTGAGTATTCTGGATATAACACATAATAAGT

>46158 TLR5

AATCCTTTCAGAAATATTACGTTTAACTCACTAGACCTTAGTGACAATGGCTGGAGCACAGAGGAAGTCCAATATTTCTGTACAGCCATTAAAGGGACTCAAATCGGTTCTTTAATACTTAGCTCTCATACAATGGGTTCAGGATTTGGCTTTAATAACTTGAAAAATCCAGATAATGATACATTTGCAGGACTAGCAAGCAGTGATCTTCATTTGCTTGACATTTCAAATGGTTACATTTTCTCTCTCAATTCTTTAATCTTTCAAAGCCTTGGTAATCTGGAATTGCTGAACCTTTCCAAAAACAAGATAAATCAAATCCAAAGGCAAGCATTTTTTGGCTTGGGAAACCTAAAAACTCTCAATCTCTCAAGTAATCTTTTAGGTGAGTTGTACGATTATACTTTTGAAGGTCTACATAGCGTAATGTATATTGATTTACAGCAAAATCATATCGGGATGATTGGTGAAAAATCATTCAGTAACTTAGTAAGCCTGAAAATAATTGATCTCCGAGACAACGCCATTAAAAAACTCCCTTCCTTTCCACATCTGACCTCTGCCTTTTTAGGTGACAATAAGCTAATGTCTGTGGTTGGTACAGCAATAGCAGCAACACTCCTTGAATTAGAAAGAAATTGGTTGGCAAACCTGGGTGACCTGTATATTCTTTTCCAAGTTCCAGATGTGCAGTATATCTTCTTAAAACAGAATCGCTTCTCTTACTGTGTGAAACGTGATACTGTTACAGAAAACAACCAGTTAATCTATATGGATCTAGGTGAAAATATGTTACAGCTTGTGTGGGAGAGAGATTTATGTTTGGATGTGTTCAGGGCACTGTCCAAACTTCAGGTTCTGCATCTGAATAACAACTACCTTAGTGCTCTTCCACGGGAGATTTTTAGAGGTCTAACATCTCTGAAAAGACTTAATCTAGCTTCCAACCTATTGTCTCATCTTTCTCCTGCGCTTTTTCCACAGAGCCTAACAATCCTAAACTTATCTGGAAACCAGCTTTTTTCCCCTGAGCCTGAAGTCTTTATGACTTTGAGTATTCTGGATATAACACATAATAAGT

**TLR7**

>4156 TLR7

CCACCCAATTTGTCATCTACTTTAAAGGAATTGTATATTTACAATAACATGATTCAAGTGATTCAAGAACAAGATTTAAGTGCCCTTCACAACCTAGAAATTCTTGATCTGAGTGGTAATTGCCCACGTTGCTATAATGCCCCATATCCTTGTATTCCCTGCCCCAAGAGCACAATTGAGATACATTCAAAGGCTTTTTATTCCCTGAAAAATTTAAGAATTTTGCGACTTCACAGTAACTCTCTTCAGAGCATACCCAGCATCTGGTTTAAAAACATCAGAAATCTCAAAGAGCTTGACCTCTCCCAAAATTTCCTCATAAAGGAGATTGGAGATGCTCAGTTTTTGAAGTTTATCCCTGGCCTTGTAGAGCTTGATTTGTCCTTTAATTTTGAACTGCAGATGTATTCTCCATTCTTGAATCTGTCTAAGACATTTTCCTCCCTCTCTAACCTGGAAACCCTGAGGCTCAGGGGTTATGTCTTTAAAGAACTGAGAGAAGGAAATCTAGATCCATTGCTCAGTCTTAGAAATCTAACAGTGTTGGATCTCGGGCTAATTTTATTAAAGTTGCTGACCTGAAAGTGTTCAAAGAATTCCCAGCTCTTAAATTCATAGACCTCTCAGTGAATAAAATTTCTCCTTCTTCAAGTGAAAGCAACTTTTATGGATTTTGCTCTAATCCTAGGATTTCAGTAGAGCAATACAACAGGCAAGTATTACAAGAGATGCATTATTTCAGGTATGATGAGTATGGGCGAAGTTGCAGGTCCAAAGACAAAGAGGCTGCTTCCTACCAATCTTCAGTTAACGAAGAGTGCCTGAAATACGGAGAAACTCTGGATTTAAGCAGAAACAACATATTTTTTATTAACCCCTCGGACTTCCGTGGTCTTAGTTTCCTCAGATGCCTCAACTTGTCAGGTAATGCAATAAGTCAAACTTTAAATGGAAGTGAATTCTACTATTTGTCTGGATTGAAATATCTGGATTTTTCTAACAACAGGATCGACTTGCTATACTCAACTGCTTTCAAAGAGCT

>4161 TLR7

CCACCCAATTTGTCATCTACTTTAAAGGAATTGTATATTTACAATAACATGATTCAAGTGATTCAAGAACAAGATTTAAGTGCCCTTCACAACCTAGAAATTCTTGATCTGAGTGGTAATTGCCCACGTTGCTATAATGCCCCATATCCTTGTATTCCCTGCCCCAAGAGCACAATTGAGATACATTCAAAGGCTTTTTATTCCCTGAAAAATTTAAGAATTTTGCGACTTCACAGTAACTCTCTTCAGAGCATACCCAGCATCTGGTTTAAAAACATCAGAAATCTCAAAGAGCTTGACCTCTCCCAAAATTTCCTCATAAAGGAGATTGGAGATGCTCAGTTTTTGAAGTTTATCCCTGGCCTTGTAGAGCTTGATTTGTCCTTTAATTTTGAACTGCAGATGTATTCTCCATTCTTGAATCTGTCTAAGACATTTTCCTCCCTCTCTAACCTGGAAACCCTGAGGCTCAGGGGTTATGTCTTTAAAGAACTGAGAGAAGGAAATCTAGATCCATTGCTCAGTCTTAGAAATCTAACAGTGTTGGATCTCGGGCTAATTTTATTAAAGTTGCTGACCTGAAAGTGTTCAAAGAATTCCCAGCTCTTAAATTCATAGACCTCTCAGTGAATAAAATTTCTCCTTCTTCAAGTGAAAGCAACTTTTATGGATTTTGCTCTAATCCTAGGATTTCAGTAGAGCAATACAACAGGCAAGTATTACAAGAGATGCATTATTTCAGGTATGATGAGTATGGGCGAAGTTGCAGGTCCAAAGACAAAGAGGCTGCTTCCTACCAATCTTCAGTTAACGAAGAGTGCCTGAAATACGGAGAAACTCTGGATTTAAGCAGAAACAACATATTTTTTATTAACCCCTCGGACTTCCGTGGTCTTAGTTTCCTCAGATGCCTCAACTTGTCAGGTAATGCAATAAGTCAAACTTTAAATGGAAGTGAATTCTACTATTTGTCTGGATTGAAATATCTGGATTTTTCTAACAACAGGATCGACTTGCTATACTCAACTGCTTTCAAAGAGCT

>4177 TLR7

CCACCCAATTTGTCATCTACTTTAAAGGAATTGTATATTTACAATAACATGATTCAAGTGATTCAAGAACAAGATTTAAGTGCCCTTCACAACCTAGAAATTCTTGATCTGAGTGGTAATTGCCCACGTTGCTATAATGCCCCATATCCTTGTATTCCCTGCCCCAAGAGCACAATTGAGATACATTCAAAGGCTTTTTATTCCCTGAAAAATTTAAGAATTTTGCGACTTCACAGTAACTCTCTTCAGAGCATACCCAGCATCTGGTTTAAAAACATCAGAAATCTCAAAGAGCTTGACCTCTCCCAAAATTTCCTCATAAAGGAGATTGGAGATGCTCAGTTTTTGAAGTTTATCCCTGGCCTTGTAGAGCTTGATTTGTCCTTTAATTTTGAACTGCAGATGTATTCTCCATTCTTGAATCTGTCTAAGACATTTTCCTCCCTCTCTAACCTGGAAACCCTGAGGCTCAGGGGTTATGTCTTTAAAGAACTGAGAGAAGGAAATCTAGATCCATTGCTCAGTCTTAGAAATCTAACAGTGTTGGATCTCGGGCTAATTTTATTAAAGTTGCTGACCTGAAAGTGTTCAAAGAATTCCCAGCTCTTAAATTCATAGACCTCTCAGTGAATAAAATTTCTCCTTCTTCAAGTGAAAGCAACTTTTATGGATTTTGCTCTAATCCTAGGATTTCAGTAGAGCAATACAACAGGCAAGTATTACAAGAGATGCATTATTTCAGGTATGATGAGTATGGGCGAAGTTGCAGGTCCAAAGACAAAGAGGCTGCTTCCTACCAATCTTCAGTTAACGAAGAGTGCCTGAAATACGGAGAAACTCTGGATTTAAGCAGAAACAACATATTTTTTATTAACCCCTCGGACTTCCGTGGTCTTAGTTTCCTCAGATGCCTCAACTTGTCAGGTAATGCAATAAGTCAAACTTTAAATGGAAGTGAATTCTACTATTTGTCTGGATTGAAATATCTGGATTTTTCTAACAACAGGATCGACTTGCTATACTCAACTGCTTTCAAAGAGCT

>4178 TLR7

CCACCCGATTTGTCATCTACTTTAAAGGAATTGGATATTTACAATAACATGATTCAAGTGATTCAAGAACAAGATTTAAGTGCCCTTCACAACCTAGAAATTCTTGATCTGAGTGGTAATTGCCCACGTTGCTATAATGCCCCATATCCTTGTATTCCCTGCCCCAAGAGCACAATTGAGATACATTCAAAGGCTTTTTATTCCCTGAAAAATTTAAGAATTTTGCGACTTCACAGTAACTCTCTTCAGAGCATACCCAGCATCTGGTTTAAAAACATCAGAAATCTCAAAGAGCTTGACCTCTCCCAAAATTTCCTCATAAAGGAGATTGGAGATGCTCAGTTTTTGAAGTTTATCCCTGGCCTTGTAGAGCTTGATTTGTCCTTTAATTTTGAACTGCAGATGTATTCTCCATTCTTGAATCTGTCTAAGACATTTTCCTCCCTCTCTAACCTGGAAACCCTGAGGCTCAGGGGTTATGTCTTTAAAGAACTGAGAGAAGGAAATCTAGATCCATTGCTCAGTCTTAGAAATCTAACAGTGTTGGATCTCGGGCTAATTTTATTAAAGTTGCTGACCTGAAAGTGTTCAAAGAATTCCCAGCTCTTAAATTCATAGACCTCTCAGTGAATAAAATTTCTCCTTCTTCAAGTGAAAGCAACTTTTATGGATTTTGCTCTAATCCTAGGATTTCAGTAGAGCAATACAACAGGCAAGTATTACAAGAGATGCATTATTTCAGGTATGATGAGTATGGGCGAAGTTGCAGGTCCAAAGACAAAGAGGCTGCTTCCTACCAATCTTCAGTTAACGAAGAGTGCCTGAAATACGGAGAAACTCTGGATTTAAGCAGAAACAACATATTTTTTATTAACCCCTCGGACTTCCGTGGTCTTAGTTTCCTCAGATGCCTCAACTTGTCAGGTAATGCAATAAGTCAAACTTTAAATGGAAGTGAATTCTACTATTTGTCTGGATTGAAATATCTGGATTTTTCTAACAACAGGATCGACTTGCTATACTCAACTGCTTTCAAAGAGCT

>4181 TLR7

CCACCCAATTTGTCATCTACTTTAAAGGAATTGTATATTTACAATAACATGATTCAAGTGATTCAAGAACAAGATTTAAGTGCCCTTCACAACCTAGAAATTCTTGATCTGAGTGGTAATTGCCCACGTTGCTATAATGCCCCATATCCTTGTATTCCCTGCCCCAAGAGCACAATTGAGATACATTCAAAGGCTTTTTATTCCCTGAAAAATTTAAGAATTTTGCGACTTCACAGTAACTCTCTTCAGAGCATACCCAGCATCTGGTTTAAAAACATCAGAAATCTCAAAGAGCTTGACCTCTCCCAAAATTTCCTCATAAAGGAGATTGGAGATGCTCAGTTTTTGAAGTTTATCCCTGGCCTTGTAGAGCTTGATTTGTCCTTTAATTTTGAACTGCAGATGTATTCTCCATTCTTGAATCTGTCTAAGACATTTTCCTCCCTCTCTAACCTGGAAACCCTGAGGCTCAGGGGTTATGTCTTTAAAGAACTGAGAGAAGGAAATCTAGATCCATTGCTCAGTCTTAGAAATCTAACAGTGTTGGATCTCGGGCTAATTTTATTAAAGTTGCTGACCTGAAAGTGTTCAAAGAATTCCCAGCTCTTAAATTCATAGACCTCTCAGTGAATAAAATTTCTCCTTCTTCAAGTGAAAGCAACTTTTATGGATTTTGCTCTAATCCTAGGATTTCAGTAGAGCAATACAACAGGCAAGTATTACAAGAGATGCATTATTTCAGGTATGATGAGTATGGGCGAAGTTGCAGGTCCAAAGACAAAGAGGCTGCTTCCTACCAATCTTCAGTTAACGAAGAGTGCCTGAAATACGGAGAAACTCTGGATTTAAGCAGAAACAACATATTTTTTATTAACCCCTCGGACTTCCGTGGTCTTAGTTTCCTCAGATGCCTCAACTTGTCAGGTAATGCAATAAGTCAAACTTTAAATGGAAGTGAATTCTACTATTTGTCTGGATTGAAATATCTGGATTTTTCTAACAACAGGATCGACTTGCTATACTCAACTGCTTTCAAAGAGCT

>4189 TLR7

CCACCCAATTTGTCATCTACTTTAAAGGAATTGTATATTTACAATAACATGATTCAAGTGATTCAAGAACAAGATTTAAGTGCCCTTCACAACCTAGAAATTCTTGATCTGAGTGGTAATTGCCCACGTTGCTATAATGCCCCATATCCTTGTATTCCCTGCCCCAAGAGCACAATTGAGATACATTCAAAGGCTTTTTATTCCCTGAAAAATTTAAGAATTTTGCGACTTCACAGTAACTCTCTTCAGAGCATACCCAGCATCTGGTTTAAAAACATCAGAAATCTCAAAGAGCTTGACCTCTCCCAAAATTTCCTCATAAAGGAGATTGGAGATGCTCAGTTTTTGAAGTTTATCCCTGGCCTTGTAGAGCTTGATTTGTCCTTTAATTTTGAACTGCAGATGTATTCTCCATTCTTGAATCTGTCTAAGACATTTTCCTCCCTCTCTAACCTGGAAACCCTGAGGCTCAGGGGTTATGTCTTTAAAGAACTGAGAGAAGGAAATCTAGATCCATTGCTCAGTCTTAGAAATCTAACAGTGTTGGATCTCGGGCTAATTTTATTAAAGTTGCTGACCTGAAAGTGTTCAAAGAATTCCCAGCTCTTAAATTCATAGACCTCTCAGTGAATAAAATTTCTCCTTCTTCAAGTGAAAGCAACTTTTATGGATTTTGCTCTAATCCTAGGATTTCAGTAGAGCAATACAACAGGCAAGTATTACAAGAGATGCATTATTTCAGGTATGATGAGTATGGGCGAAGTTGCAGGTCCAAAGACAAAGAGGCTGCTTCCTACCAATCTTCAGTTAACGAAGAGTGCCTGAAATACGGAGAAACTCTGGATTTAAGCAGAAACAACATATTTTTTATTAACCCCTCGGACTTCCGTGGTCTTAGTTTCCTCAGATGCCTCAACTTGTCAGGTAATGCAATAAGTCAAACTTTAAATGGAAGTGAATTCTACTATTTGTCTGGATTGAAATATCTGGATTTTTCTAACAACAGGATCGACTTGCTATACTCAACTGCTTTCAAAGAGCT

>711 TLR7

CCACCCAATTTGTCATCTACTTTAAAGGAATTGTATATTTACAATAACATGATTCAAGTGATTCAAGAACAAGATTTAAGTGCCCTTCACAACCTAGAAATTCTTGATCTGAGTGGTAATTGCCCACGTTGCTATAATGCCCCATATCCTTGTATTCCCTGCCCCAAGAGCACAATTGAGATACATTCAAAGGCTTTTTATTCCCTGAAAAATTTAAGAATTTTGCGACTTCACAGTAACTCTCTTCAGAGCATACCCAGCATCTGGTTTAAAAACATCAGAAATCTCAAAGAGCTTGACCTCTCCCAAAATTTCCTCATAAAGGAGATTGGAGATGCTCAGTTTTTGAAGTTTATCCCTGGCCTTGTAGAGCTTGATTTGTCCTTTAATTTTGAACTGCAGATGTATTCTCCATTCTTGAATCTGTCTAAGACATTTTCCTCCCTCTCTAACCTGGAAACCCTGAGGCTCAGGGGTTATGTCTTTAAAGAACTGAGAGAAGGAAATCTAGATCCATTGCTCAGTCTTAGAAATCTAACAGTGTTGGATCTCGGGCTAATTTTATTAAAGTTGCTGACCTGAAAGTGTTCAAAGAATTCCCAGCTCTTAAATTCATAGACCTCTCAGTGAATAAAATTTCTCCTTCTTCAAGTGAAAGCAACTTTTATGGATTTTGCTCTAATCCTAGGATTTCAGTAGAGCAATACAACAGGCAAGTATTACAAGAGATGCATTATTTCAGGTATGATGAGTATGGGCGAAGTTGCAGGTCCAAAGACAAAGAGGCTGCTTCCTACCAATCTTCAGTTAACGAAGAGTGCCTGAAATACGGAGAAACTCTGGATTTAAGCAGAAACAACATATTTTTTATTAACCCCTCGGACTTCCGTGGTCTTAGTTTCCTCAGATGCCTCAACTTGTCAGGTAATGCAATAAGTCAAACTTTAAATGGAAGTGAATTCTACTATTTGTCTGGATTGAAATATCTGGATTTTTCTAACAACAGGATCGACTTGCTATACTCAACTGCTTTCAAAGAGCT

>713 TLR7

CCACCCGATTTGTCATCTACTTTAAAGGAAGTGGATATTTACAATAACATGATTCAAGTGATTCAGGAACAAGATTTAAGTGCCCTTCACAACCTAGAAATTCTTGATCTGAGTGGTAATTGCCCACGTTGCTATAATGCCCCATATCCTTGTATTCCCTGCCCCAAGAGCACAATTGAGATACATTCAAAGGCTTTTTATTCCCTGAAAAATTTAAGAATTTTGCGACTTCACAGTAACTCTCTTCAGAGCATACCCAGCATCTGGTTTAAAAACATCAGAAATCTCAAAGAGCTTGACCTCTCCCAAAATTTCCTCATAAAGGAGATTGGAGATGCTCAGTTTTTGAAGTTTATCCCTGGCCTTGTAGAGCTTGATTTGTCCTTTAATTTTGAACTGCAGATGTATTCTCCATTCTTGAATCTGTCTAAGACATTTTCCTCCCTCTCTAACCTGGAAACCCTGAGGCTCAGGGGTTATGTCTTTAAAGAACTGAGAGAAGGAAATCTAGATCCATTGCTCAGTCTTAGAAATCTAACAGTGTTGGATCTCGGGCTAATTTTATTAAAGTTGCTGACCTGAAAGTGTTCAAAGAATTCCCAGCTCTTAAATTCATAGACCTCTCAGTGAATAAAATTTCTCCTTCTTCAAGTGAAAGCAACTTTTATGGATTTTGCTCTAATCCTAGGATTTCAGTAGAGCAATACAACAGGCAAGTATTACAAGAGATGCATTATTTCAGGTATGATGAGTATGGGCGAAGTTGCAGGTCCAAAGACAAAGAGGCTGCTTCCTACCAATCTTCAGTTAACGAAGAGTGCCTGAAATACGGAGAAACTCTGGATTTAAGCAGAAACAACATATTTTTTATTAACCCCTCGGACTTCCGTGGTCTTAGTTTCCTCAGATGCCTCAACTTGTCAGGTAATGCAATAAGTCAAACTTTAAATGGAAGTGAATTCTACTATTTGTCTGGATTGAAATATCTGGATTTTTCTAACAACAGGATCGACTTGCTATACTCAACTGCTTTCAAAGAGCT

>715 TLR7

CCACCCGATTTGTCATCTACTTTAAAGGAATTGTATATTTACAATAACATGATTCAAGTGATTCAAGAACAAGATTTAAGTGCCCTTCACAACCTAGAAATTCTTGATCTGAGTGGTAATTGCCCACGTTGCTATAATGCCCCATATCCTTGTATTCCCTGCCCCAAGAGCACAATTGAGATACATTCAAAGGCTTTTTATTCCCTGAAAAATTTAAGAATTTTGCGACTTCACAGTAACTCTCTTCAGAGCATACCCAGCATCTGGTTTAAAAACATCAGAAATCTCAAAGAGCTTGACCTCTCCCAAAATTTCCTCATAAAGGAGATTGGAGATGCTCAGTTTTTGAAGTTTATCCCTGGCCTTGTAGAGCTTGATTTGTCCTTTAATTTTGAACTGCAGATGTATTCTCCATTCTTGAATCTGTCTAAGACATTTTCCTCCCTCTCTAACCTGGAAACCCTGAGGCTCAGGGGTTATGTCTTTAAAGAACTGAGAGAAGGAAATCTAGATCCATTGCTCAGTCTTAGAAATCTAACAGTGTTGGATCTCGGGCTAATTTTATTAAAGTTGCTGACCTGAAAGTGTTCAAAGAATTCCCAGCTCTTAAATTCATAGACCTCTCAGTGAATAAAATTTCTCCTTCTTCAAGTGAAAGCAACTTTTATGGATTTTGCTCTAATCCTAGGATTTCAGTAGAGCAATACAACAGGCAAGTATTACAAGAGATGCATTATTTCAGGTATGATGAGTATGGGCGAAGTTGCAGGTCCAAAGACAAAGAGGCTGCTTCCTACCAATCTTCAGTTAACGAAGAGTGCCTGAAATACGGAGAAACTCTGGATTTAAGCAGAAACAACATATTTTTTATTAACCCCTCGGACTTCCGTGGTCTTAGTTTCCTCAGATGCCTCAACTTGTCAGGTAATGCAATAAGTCAAACTTTAAATGGAAGTGAATTCTACTATTTGTCTGGATTGAAATATCTGGATTTTTCTAACAACAGGATCGACTTGCTATACTCAACTGCTTTCAAAGAGCT

>822 TLR7

CCGCCCGATTTGTCATCTACGTTAAAGGAAGTGGATATTTACAATAACATGATTCAGCTGATTCAGGAACGGGATTTAAGTGCCCTTCACAACCTAGAAATTCTTGATCTGAGTGGTAATTGCCCACGTTGCTATAATGCCCCATATCCTTGTATTCCCTGCCCCAAGAGCACAATTGAGATACATTCAAAGGCTTTTTATTCCCTGAAAAATTTAAGAATTTTGCGACTTCACAGTAACTCTCTTCAGAGCATACCCAGCATCTGGTTTAAAAACATCAGAAATCTCAAAGAGCTTGACCTCTCCCAAAATTTCCTCATAAAGGAGATTGGAGATGCTCAGTTTTTGAAGTTTATCCCTGGCCTTGTAGAGCTTGATTTGTCCTTTAATTTTGAACTGCAGATGTATTCTCCATTCTTGAATCTGTCTAAGACATTTTCCTCCCTCTCTAACCTGGAAACCCTGAGGCTCAGGGGTTATGTCTTTAAAGAACTGAGAGAAGGAAATCTAGATCCATTGCTCAGTCTTAGAAATCTAACAGTGTTGGATCTCGGGCTAATTTTATTAAAGTTGCTGACCTGAAAGTGTTCAAAGAATTCCCAGCTCTTAAATTCATAGACCTCTCAGTGAATAAAATTTCTCCTTCTTCAAGTGAAAGCAACTTTTATGGATTTTGCTCTAATCCTAGGATTTCAGTAGAGCAATACAACAGGCAAGTATTACAAGAGATGCATTATTTCAGGTATGATGAGTATGGGCGAAGTTGCAGGTCCAAAGACAAAGAGGCTGCTTCCTACCAATCTTCAGTTAACGAAGAGTGCCTGAAATACGGAGAAACTCTGGATTTAAGCAGAAACAACATATTTTTTATTAACCCCTCGGACTTCCGTGGTCTTAGTTTCCTCAGATGCCTCAACTTGTCAGGTAATGCAATAAGTCAAACTTTAAATGGAAGTGAATTCTACTATTTGTCTGGATTGAAATATCTGGATTTTTCTAACAACAGGATCGACTTGCTATACTCAACTGCTTTCAAAGAGCT

>823 TLR7

CCACCCGATTTGTCATCTACTTTAAAGGAATTGTATATTTACAATAACATGATTCAAGTGATTCAAGAACAAGATTTAAGTGCCCTTCACAACCTAGAAATTCTTGATCTGAGTGGTAATTGCCCACGTTGCTATAATGCCCCATATCCTTGTATTCCCTGCCCCAAGAGCACAATTGAGATACATTCAAAGGCTTTTTATTCCCTGAAAAATTTAAGAATTTTGCGACTTCACAGTAACTCTCTTCAGAGCATACCCAGCATCTGGTTTAAAAACATCAGAAATCTCAAAGAGCTTGACCTCTCCCAAAATTTCCTCATAAAGGAGATTGGAGATGCTCAGTTTTTGAAGTTTATCCCTGGCCTTGTAGAGCTTGATTTGTCCTTTAATTTTGAACTGCAGATGTATTCTCCATTCTTGAATCTGTCTAAGACATTTTCCTCCCTCTCTAACCTGGAAACCCTGAGGCTCAGGGGTTATGTCTTTAAAGAACTGAGAGAAGGAAATCTAGATCCATTGCTCAGTCTTAGAAATCTAACAGTGTTGGATCTCGGGCTAATTTTATTAAAGTTGCTGACCTGAAAGTGTTCAAAGAATTCCCAGCTCTTAAATTCATAGACCTCTCAGTGAATAAAATTTCTCCTTCTTCAAGTGAAAGCAACTTTTATGGATTTTGCTCTAATCCTAGGATTTCAGTAGAGCAATACAACAGGCAAGTATTACAAGAGATGCATTATTTCAGGTATGATGAGTATGGGCGAAGTTGCAGGTCCAAAGACAAAGAGGCTGCTTCCTACCAATCTTCAGTTAACGAAGAGTGCCTGAAATACGGAGAAACTCTGGATTTAAGCAGAAACAACATATTTTTTATTAACCCCTCGGACTTCCGTGGTCTTAGTTTCCTCAGATGCCTCAACTTGTCAGGTAATGCAATAAGTCAAACTTTAAATGGAAGTGAATTCTACTATTTGTCTGGATTGAAATATCTGGATTTTTCTAACAACAGGATCGACTTGCTATACTCAACTGCTTTCAAAGAGCT

>824 TLR7

CCACCCAATTTGTCATCTACTTTAAAGGAATTGTATATTTACAATAACATGATTCAAGTGATTCAAGAACAAGATTTAAGTGCCCTTCACAACCTAGAAATTCTTGATCTGAGTGGTAATTGCCCACGTTGCTATAATGCCCCATATCCTTGTATTCCCTGCCCCAAGAGCACAATTGAGATACATTCAAAGGCTTTTTATTCCCTGAAAAATTTAAGAATTTTGCGACTTCACAGTAACTCTCTTCAGAGCATACCCAGCATCTGGTTTAAAAACATCAGAAATCTCAAAGAGCTTGACCTCTCCCAAAATTTCCTCATAAAGGAGATTGGAGATGCTCAGTTTTTGAAGTTTATCCCTGGCCTTGTAGAGCTTGATTTGTCCTTTAATTTTGAACTGCAGATGTATTCTCCATTCTTGAATCTGTCTAAGACATTTTCCTCCCTCTCTAACCTGGAAACCCTGAGGCTCAGGGGTTATGTCTTTAAAGAACTGAGAGAAGGAAATCTAGATCCATTGCTCAGTCTTAGAAATCTAACAGTGTTGGATCTCGGGCTAATTTTATTAAAGTTGCTGACCTGAAAGTGTTCAAAGAATTCCCAGCTCTTAAATTCATAGACCTCTCAGTGAATAAAATTTCTCCTTCTTCAAGTGAAAGCAACTTTTATGGATTTTGCTCTAATCCTAGGATTTCAGTAGAGCAATACAACAGGCAAGTATTACAAGAGATGCATTATTTCAGGTATGATGAGTATGGGCGAAGTTGCAGGTCCAAAGACAAAGAGGCTGCTTCCTACCAATCTTCAGTTAACGAAGAGTGCCTGAAATACGGAGAAACTCTGGATTTAAGCAGAAACAACATATTTTTTATTAACCCCTCGGACTTCCGTGGTCTTAGTTTCCTCAGATGCCTCAACTTGTCAGGTAATGCAATAAGTCAAACTTTAAATGGAAGTGAATTCTACTATTTGTCTGGATTGAAATATCTGGATTTTTCTAACAACAGGATCGACTTGCTATACTCAACTGCTTTCAAAGAGCT

>826 TLR7

CCACCCAATTTGTCATCTACTTTAAAGGAATTGTATATTTACAATAACATGATTCAAGTGATTCAAGAACAAGATTTAAGTGCCCTTCACAACCTAGAAATTCTTGATCTGAGTGGTAATTGCCCACGTTGCTATAATGCCCCATATCCTTGTATTCCCTGCCCCAAGAGCACAATTGAGATACATTCAAAGGCTTTTTATTCCCTGAAAAATTTAAGAATTTTGCGACTTCACAGTAACTCTCTTCAGAGCATACCCAGCATCTGGTTTAAAAACATCAGAAATCTCAAAGAGCTTGACCTCTCCCAAAATTTCCTCATAAAGGAGATTGGAGATGCTCAGTTTTTGAAGTTTATCCCTGGCCTTGTAGAGCTTGATTTGTCCTTTAATTTTGAACTGCAGATGTATTCTCCATTCTTGAATCTGTCTAAGACATTTTCCTCCCTCTCTAACCTGGAAACCCTGAGGCTCAGGGGTTATGTCTTTAAAGAACTGAGAGAAGGAAATCTAGATCCATTGCTCAGTCTTAGAAATCTAACAGTGTTGGATCTCGGGCTAATTTTATTAAAGTTGCTGACCTGAAAGTGTTCAAAGAATTCCCAGCTCTTAAATTCATAGACCTCTCAGTGAATAAAATTTCTCCTTCTTCAAGTGAAAGCAACTTTTATGGATTTTGCTCTAATCCTAGGATTTCAGTAGAGCAATACAACAGGCAAGTATTACAAGAGATGCATTATTTCAGGTATGATGAGTATGGGCGAAGTTGCAGGTCCAAAGACAAAGAGGCTGCTTCCTACCAATCTTCAGTTAACGAAGAGTGCCTGAAATACGGAGAAACTCTGGATTTAAGCAGAAACAACATATTTTTTATTAACCCCTCGGACTTCCGTGGTCTTAGTTTCCTCAGATGCCTCAACTTGTCAGGTAATGCAATAAGTCAAACTTTAAATGGAAGTGAATTCTACTATTTGTCTGGATTGAAATATCTGGATTTTTCTAACAACAGGATCGACTTGCTATACTCAACTGCTTTCAAAGAGCT

>838 TLR7

CCACCCGATTTGTGATCTACTTTAAAGGAATTGGATATTTACAATAACATGATTCAAGTGATTCAAGAACAAGATTTAAGTGCCCTTCACAACCTAGAAATTCTTGATCTGAGTGGTAATTGCCCACGTTGCTATAATGCCCCATATCCTTGTATTCCCTGCCCCAAGAGCACAATTGAGATACATTCAAAGGCTTTTTATTCCCTGAAAAATTTAAGAATTTTGCGACTTCACAGTAACTCTCTTCAGAGCATACCCAGCATCTGGTTTAAAAACATCAGAAATCTCAAAGAGCTTGACCTCTCCCAAAATTTCCTCATAAAGGAGATTGGAGATGCTCAGTTTTTGAAGTTTATCCCTGGCCTTGTAGAGCTTGATTTGTCCTTTAATTTTGAACTGCAGATGTATTCTCCATTCTTGAATCTGTCTAAGACATTTTCCTCCCTCTCTAACCTGGAAACCCTGAGGCTCAGGGGTTATGTCTTTAAAGAACTGAGAGAAGGAAATCTAGATCCATTGCTCAGTCTTAGAAATCTAACAGTGTTGGATCTCGGGCTAATTTTATTAAAGTTGCTGACCTGAAAGTGTTCAAAGAATTCCCAGCTCTTAAATTCATAGACCTCTCAGTGAATAAAATTTCTCCTTCTTCAAGTGAAAGCAACTTTTATGGATTTTGCTCTAATCCTAGGATTTCAGTAGAGCAATACAACAGGCAAGTATTACAAGAGATGCATTATTTCAGGTATGATGAGTATGGGCGAAGTTGCAGGTCCAAAGACAAAGAGGCTGCTTCCTACCAATCTTCAGTTAACGAAGAGTGCCTGAAATACGGAGAAACTCTGGATTTAAGCAGAAACAACATATTTTTTATTAACCCCTCGGACTTCCGTGGTCTTAGTTTCCTCAGATGCCTCAACTTGTCAGGTAATGCAATAAGTCAAACTTTAAATGGAAGTGAATTCTACTATTTGTCTGGATTGAAATATCTGGATTTTTCTAACAACAGGATCGACTTGCTATACTCAACTGCTTTCAAAGAGCT

>103 TLR7

CCACCCGATTTGTCATCTACTTTAAAGGAATTGGATATTTACAATAACATGATTCAAGTGATTCAAGAACAAGATTTAAGTGCCCTTCACAACCTAGAAATTCTTGATCTGAGTGGTAATTGCCCACGTTGCTATAATGCCCCATATCCTTGTATTCCCTGCCCCAAGAGCACAATTGAGATACATTCAAAGGCTTTTTATTCCCTGAAAAATTTAAGAATTTTGCGACTTCACAGTAACTCTCTTCAGAGCATACCCAGCATCTGGTTTAAAAACATCAGAAATCTCAAAGAGCTTGACCTCTCCCAAAATTTCCTCATAAAGGAGATTGGAGATGCTCAGTTTTTGAAGTTTATCCCTGGCCTTGTAGAGCTTGATTTGTCCTTTAATTTTGAACTGCAGATGTATTCTCCATTCTTGAATCTGTCTAAGACATTTTCCTCCCTCTCTAACCTGGAAACCCTGAGGCTCAGGGGTTATGTCTTTAAAGAACTGAGAGAAGGAAATCTAGATCCATTGCTCAGTCTTAGAAATCTAACAGTGTTGGATCTCGGGCTAATTTTATTAAAGTTGCTGACCTGAAAGTGTTCAAAGAATTCCCAGCTCTTAAATTCATAGACCTCTCAGTGAATAAAATTTCTCCTTCTTCAAGTGAAAGCAACTTTTATGGATTTTGCTCTAATCCTAGGATTTCAGTAGAGCAATACAACAGGCAAGTATTACAAGAGATGCATTATTTCAGGTATGATGAGTATGGGCGAAGTTGCAGGTCCAAAGACAAAGAGGCTGCTTCCTACCAATCTTCAGTTAACGAAGAGTGCCTGAAATACGGAGAAACTCTGGATTTAAGCAGAAACAACATATTTTTTATTAACCCCTCGGACTTCCGTGGTCTTAGTTTCCTCAGATGCCTCAACTTGTCAGGTAATGCAATAAGTCAAACTTTAAATGGAAGTGAATTCTACTATTTGTCTGGATTGAAATATCTGGATTTTTCTAACAACAGGATCGACTTGCTATACTCAACTGCTTTCAAAGAGCT

>1011 TLR7

CCACCCGATTTGTCATCTACTTTAAAGGAATTGTATATTTACAATAACATGATTCAAGTGATTCAAGAACAAGATTTAAGTGCCCTTCACAACCTAGAAATTCTTGATCTGAGTGGTAATTGCCCACGTTGCTATAATGCCCCATATCCTTGTATTCCCTGCCCCAAGAGCACAATTGAGATACATTCAAAGGCTTTTTATTCCCTGAAAAATTTAAGAATTTTGCGACTTCACAGTAACTCTCTTCAGAGCATACCCAGCATCTGGTTTAAAAACATCAGAAATCTCAAAGAGCTTGACCTCTCCCAAAATTTCCTCATAAAGGAGATTGGAGATGCTCAGTTTTTGAAGTTTATCCCTGGCCTTGTAGAGCTTGATTTGTCCTTTAATTTTGAACTGCAGATGTATTCTCCATTCTTGAATCTGTCTAAGACATTTTCCTCCCTCTCTAACCTGGAAACCCTGAGGCTCAGGGGTTATGTCTTTAAAGAACTGAGAGAAGGAAATCTAGATCCATTGCTCAGTCTTAGAAATCTAACAGTGTTGGATCTCGGGCTAATTTTATTAAAGTTGCTGACCTGAAAGTGTTCAAAGAATTCCCAGCTCTTAAATTCATAGACCTCTCAGTGAATAAAATTTCTCCTTCTTCAAGTGAAAGCAACTTTTATGGATTTTGCTCTAATCCTAGGATTTCAGTAGAGCAATACAACAGGCAAGTATTACAAGAGATGCATTATTTCAGGTATGATGAGTATGGGCGAAGTTGCAGGTCCAAAGACAAAGAGGCTGCTTCCTACCAATCTTCAGTTAACGAAGAGTGCCTGAAATACGGAGAAACTCTGGATTTAAGCAGAAACAACATATTTTTTATTAACCCCTCGGACTTCCGTGGTCTTAGTTTCCTCAGATGCCTCAACTTGTCAGGTAATGCAATAAGTCAAACTTTAAATGGAAGTGAATTCTACTATTTGTCTGGATTGAAATATCTGGATTTTTCTAACAACAGGATCGACTTGCTATACTCAACTGCTTTCAAAGAGCT

>1017 TLR7

CCACCCGATTTGTCATCTACTTTAAAGGAATTGTATATTTACAATAACATGATTCAAGTGATTCAAGAACAAGATTTAAGTGCCCTTCACAACCTAGAAATTCTTGATCTGAGTGGTAATTGCCCACGTTGCTATAATGCCCCATATCCTTGTATTCCCTGCCCCAAGAGCACAATTGAGATACATTCAAAGGCTTTTTATTCCCTGAAAAATTTAAGAATTTTGCGACTTCACAGTAACTCTCTTCAGAGCATACCCAGCATCTGGTTTAAAAACATCAGAAATCTCAAAGAGCTTGACCTCTCCCAAAATTTCCTCATAAAGGAGATTGGAGATGCTCAGTTTTTGAAGTTTATCCCTGGCCTTGTAGAGCTTGATTTGTCCTTTAATTTTGAACTGCAGATGTATTCTCCATTCTTGAATCTGTCTAAGACATTTTCCTCCCTCTCTAACCTGGAAACCCTGAGGCTCAGGGGTTATGTCTTTAAAGAACTGAGAGAAGGAAATCTAGATCCATTGCTCAGTCTTAGAAATCTAACAGTGTTGGATCTCGGGCTAATTTTATTAAAGTTGCTGACCTGAAAGTGTTCAAAGAATTCCCAGCTCTTAAATTCATAGACCTCTCAGTGAATAAAATTTCTCCTTCTTCAAGTGAAAGCAACTTTTATGGATTTTGCTCTAATCCTAGGATTTCAGTAGAGCAATACAACAGGCAAGTATTACAAGAGATGCATTATTTCAGGTATGATGAGTATGGGCGAAGTTGCAGGTCCAAAGACAAAGAGGCTGCTTCCTACCAATCTTCAGTTAACGAAGAGTGCCTGAAATACGGAGAAACTCTGGATTTAAGCAGAAACAACATATTTTTTATTAACCCCTCGGACTTCCGTGGTCTTAGTTTCCTCAGATGCCTCAACTTGTCAGGTAATGCAATAAGTCAAACTTTAAATGGAAGTGAATTCTACTATTTGTCTGGATTGAAATATCTGGATTTTTCTAACAACAGGATCGACTTGCTATACTCAACTGCTTTCAAAGAGCT

>1020 TLR7

CCACCCGATTTGTCATCTACTTTAAAGGAATTGTATATTTACAATAACATGATTCAAGTGATTCAAGAACAAGATTTAAGTGCCCTTCACAACCTAGAAATTCTTGATCTGAGTGGTAATTGCCCACGTTGCTATAATGCCCCATATCCTTGTATTCCCTGCCCCAAGAGCACAATTGAGATACATTCAAAGGCTTTTTATTCCCTGAAAAATTTAAGAATTTTGCGACTTCACAGTAACTCTCTTCAGAGCATACCCAGCATCTGGTTTAAAAACATCAGAAATCTCAAAGAGCTTGACCTCTCCCAAAATTTCCTCATAAAGGAGATTGGAGATGCTCAGTTTTTGAAGTTTATCCCTGGCCTTGTAGAGCTTGATTTGTCCTTTAATTTTGAACTGCAGATGTATTCTCCATTCTTGAATCTGTCTAAGACATTTTCCTCCCTCTCTAACCTGGAAACCCTGAGGCTCAGGGGTTATGTCTTTAAAGAACTGAGAGAAGGAAATCTAGATCCATTGCTCAGTCTTAGAAATCTAACAGTGTTGGATCTCGGGCTAATTTTATTAAAGTTGCTGACCTGAAAGTGTTCAAAGAATTCCCAGCTCTTAAATTCATAGACCTCTCAGTGAATAAAATTTCTCCTTCTTCAAGTGAAAGCAACTTTTATGGATTTTGCTCTAATCCTAGGATTTCAGTAGAGCAATACAACAGGCAAGTATTACAAGAGATGCATTATTTCAGGTATGATGAGTATGGGCGAAGTTGCAGGTCCAAAGACAAAGAGGCTGCTTCCTACCAATCTTCAGTTAACGAAGAGTGCCTGAAATACGGAGAAACTCTGGATTTAAGCAGAAACAACATATTTTTTATTAACCCCTCGGACTTCCGTGGTCTTAGTTTCCTCAGATGCCTCAACTTGTCAGGTAATGCAATAAGTCAAACTTTAAATGGAAGTGAATTCTACTATTTGTCTGGATTGAAATATCTGGATTTTTCTAACAACAGGATCGACTTGCTATACTCAACTGCTTTCAAAGAGCT

>1210 TLR7

CCACCCGATTTGTCATCTACTTTAAAGGAATTGGATATTTACAATAACATGATTCAAGTGATTCAAGAACAAGATTTAAGTGCCCTTCACAACCTAGAAATTCTTGATCTGAGTGGTAATTGCCCACGTTGCTATAATGCCCCATATCCTTGTATTCCCTGCCCCAAGAGCACAATTGAGATACATTCAAAGGCTTTTTATTCCCTGAAAAATTTAAGAATTTTGCGACTTCACAGTAACTCTCTTCAGAGCATACCCAGCATCTGGTTTAAAAACATCAGAAATCTCAAAGAGCTTGACCTCTCCCAAAATTTCCTCATAAAGGAGATTGGAGATGCTCAGTTTTTGAAGTTTATCCCTGGCCTTGTAGAGCTTGATTTGTCCTTTAATTTTGAACTGCAGATGTATTCTCCATTCTTGAATCTGTCTAAGACATTTTCCTCCCTCTCTAACCTGGAAACCCTGAGGCTCAGGGGTTATGTCTTTAAAGAACTGAGAGAAGGAAATCTAGATCCATTGCTCAGTCTTAGAAATCTAACAGTGTTGGATCTCGGGCTAATTTTATTAAAGTTGCTGACCTGAAAGTGTTCAAAGAATTCCCAGCTCTTAAATTCATAGACCTCTCAGTGAATAAAATTTCTCCTTCTTCAAGTGAAAGCAACTTTTATGGATTTTGCTCTAATCCTAGGATTTCAGTAGAGCAATACAACAGGCAAGTATTACAAGAGATGCATTATTTCAGGTATGATGAGTATGGGCGAAGTTGCAGGTCCAAAGACAAAGAGGCTGCTTCCTACCAATCTTCAGTTAACGAAGAGTGCCTGAAATACGGAGAAACTCTGGATTTAAGCAGAAACAACATATTTTTTATTAACCCCTCGGACTTCCGTGGTCTTAGTTTCCTCAGATGCCTCAACTTGTCAGGTAATGCAATAAGTCAAACTTTAAATGGAAGTGAATTCTACTATTTGTCTGGATTGAAATATCTGGATTTTTCTAACAACAGGATCGACTTGCTATACTCAACTGCTTTCAAAGAGCT

>1211 TLR7

CCACCCGATTTGTCATCTACTTTAAAGGAATTGTATATTTACAATAACATGATTCAAGTGATTCAAGAACAAGATTTAAGTGCCCTTCACAACCTAGAAATTCTTGATCTGAGTGGTAATTGCCCACGTTGCTATAATGCCCCATATCCTTGTATTCCCTGCCCCAAGAGCACAATTGAGATACATTCAAAGGCTTTTTATTCCCTGAAAAATTTAAGAATTTTGCGACTTCACAGTAACTCTCTTCAGAGCATACCCAGCATCTGGTTTAAAAACATCAGAAATCTCAAAGAGCTTGACCTCTCCCAAAATTTCCTCATAAAGGAGATTGGAGATGCTCAGTTTTTGAAGTTTATCCCTGGCCTTGTAGAGCTTGATTTGTCCTTTAATTTTGAACTGCAGATGTATTCTCCATTCTTGAATCTGTCTAAGACATTTTCCTCCCTCTCTAACCTGGAAACCCTGAGGCTCAGGGGTTATGTCTTTAAAGAACTGAGAGAAGGAAATCTAGATCCATTGCTCAGTCTTAGAAATCTAACAGTGTTGGATCTCGGGCTAATTTTATTAAAGTTGCTGACCTGAAAGTGTTCAAAGAATTCCCAGCTCTTAAATTCATAGACCTCTCAGTGAATAAAATTTCTCCTTCTTCAAGTGAAAGCAACTTTTATGGATTTTGCTCTAATCCTAGGATTTCAGTAGAGCAATACAACAGGCAAGTATTACAAGAGATGCATTATTTCAGGTATGATGAGTATGGGCGAAGTTGCAGGTCCAAAGACAAAGAGGCTGCTTCCTACCAATCTTCAGTTAACGAAGAGTGCCTGAAATACGGAGAAACTCTGGATTTAAGCAGAAACAACATATTTTTTATTAACCCCTCGGACTTCCGTGGTCTTAGTTTCCTCAGATGCCTCAACTTGTCAGGTAATGCAATAAGTCAAACTTTAAATGGAAGTGAATTCTACTATTTGTCTGGATTGAAATATCTGGATTTTTCTAACAACAGGATCGACTTGCTATACTCAACTGCTTTCAAAGAGCT

>1214 TLR7

CCACCCAATTTGTCATCTACTTTAAAGGAATTGTATATTTACAATAACATGATTCAAGTGATTCAAGAACAAGATTTAAGTGCCCTTCACAACCTAGAAATTCTTGATCTGAGTGGTAATTGCCCACGTTGCTATAATGCCCCATATCCTTGTATTCCCTGCCCCAAGAGCACAATTGAGATACATTCAAAGGCTTTTTATTCCCTGAAAAATTTAAGAATTTTGCGACTTCACAGTAACTCTCTTCAGAGCATACCCAGCATCTGGTTTAAAAACATCAGAAATCTCAAAGAGCTTGACCTCTCCCAAAATTTCCTCATAAAGGAGATTGGAGATGCTCAGTTTTTGAAGTTTATCCCTGGCCTTGTAGAGCTTGATTTGTCCTTTAATTTTGAACTGCAGATGTATTCTCCATTCTTGAATCTGTCTAAGACATTTTCCTCCCTCTCTAACCTGGAAACCCTGAGGCTCAGGGGTTATGTCTTTAAAGAACTGAGAGAAGGAAATCTAGATCCATTGCTCAGTCTTAGAAATCTAACAGTGTTGGATCTCGGGCTAATTTTATTAAAGTTGCTGACCTGAAAGTGTTCAAAGAATTCCCAGCTCTTAAATTCATAGACCTCTCAGTGAATAAAATTTCTCCTTCTTCAAGTGAAAGCAACTTTTATGGATTTTGCTCTAATCCTAGGATTTCAGTAGAGCAATACAACAGGCAAGTATTACAAGAGATGCATTATTTCAGGTATGATGAGTATGGGCGAAGTTGCAGGTCCAAAGACAAAGAGGCTGCTTCCTACCAATCTTCAGTTAACGAAGAGTGCCTGAAATACGGAGAAACTCTGGATTTAAGCAGAAACAACATATTTTTTATTAACCCCTCGGACTTCCGTGGTCTTAGTTTCCTCAGATGCCTCAACTTGTCAGGTAATGCAATAAGTCAAACTTTAAATGGAAGTGAATTCTACTATTTGTCTGGATTGAAATATCTGGATTTTTCTAACAACAGGATCGACTTGCTATACTCAACTGCTTTCAAAGAGCT

>1215 TLR7

CCACCCAATTTGTCATCTACTTTAAAGGAATTGTATATTTACAATAACATGATTCAAGTGATTCAAGAACAAGATTTAAGTGCCCTTCACAACCTAGAAATTCTTGATCTGAGTGGTAATTGCCCACGTTGCTATAATGCCCCATATCCTTGTATTCCCTGCCCCAAGAGCACAATTGAGATACATTCAAAGGCTTTTTATTCCCTGAAAAATTTAAGAATTTTGCGACTTCACAGTAACTCTCTTCAGAGCATACCCAGCATCTGGTTTAAAAACATCAGAAATCTCAAAGAGCTTGACCTCTCCCAAAATTTCCTCATAAAGGAGATTGGAGATGCTCAGTTTTTGAAGTTTATCCCTGGCCTTGTAGAGCTTGATTTGTCCTTTAATTTTGAACTGCAGATGTATTCTCCATTCTTGAATCTGTCTAAGACATTTTCCTCCCTCTCTAACCTGGAAACCCTGAGGCTCAGGGGTTATGTCTTTAAAGAACTGAGAGAAGGAAATCTAGATCCATTGCTCAGTCTTAGAAATCTAACAGTGTTGGATCTCGGGCTAATTTTATTAAAGTTGCTGACCTGAAAGTGTTCAAAGAATTCCCAGCTCTTAAATTCATAGACCTCTCAGTGAATAAAATTTCTCCTTCTTCAAGTGAAAGCAACTTTTATGGATTTTGCTCTAATCCTAGGATTTCAGTAGAGCAATACAACAGGCAAGTATTACAAGAGATGCATTATTTCAGGTATGATGAGTATGGGCGAAGTTGCAGGTCCAAAGACAAAGAGGCTGCTTCCTACCAATCTTCAGTTAACGAAGAGTGCCTGAAATACGGAGAAACTCTGGATTTAAGCAGAAACAACATATTTTTTATTAACCCCTCGGACTTCCGTGGTCTTAGTTTCCTCAGATGCCTCAACTTGTCAGGTAATGCAATAAGTCAAACTTTAAATGGAAGTGAATTCTACTATTTGTCTGGATTGAAATATCTGGATTTTTCTAACAACAGGATCGACTTGCTATACTCAACTGCTTTCAAAGAGCT

>1218 TLR7

CCACCCAATTTGTCATCTACTTTAAAGGAATTGTATATTTACAATAACATGATTCAAGTGATTCAAGAACAAGATTTAAGTGCCCTTCACAACCTAGAAATTCTTGATCTGAGTGGTAATTGCCCACGTTGCTATAATGCCCCATATCCTTGTATTCCCTGCCCCAAGAGCACAATTGAGATACATTCAAAGGCTTTTTATTCCCTGAAAAATTTAAGAATTTTGCGACTTCACAGTAACTCTCTTCAGAGCATACCCAGCATCTGGTTTAAAAACATCAGAAATCTCAAAGAGCTTGACCTCTCCCAAAATTTCCTCATAAAGGAGATTGGAGATGCTCAGTTTTTGAAGTTTATCCCTGGCCTTGTAGAGCTTGATTTGTCCTTTAATTTTGAACTGCAGATGTATTCTCCATTCTTGAATCTGTCTAAGACATTTTCCTCCCTCTCTAACCTGGAAACCCTGAGGCTCAGGGGTTATGTCTTTAAAGAACTGAGAGAAGGAAATCTAGATCCATTGCTCAGTCTTAGAAATCTAACAGTGTTGGATCTCGGGCTAATTTTATTAAAGTTGCTGACCTGAAAGTGTTCAAAGAATTCCCAGCTCTTAAATTCATAGACCTCTCAGTGAATAAAATTTCTCCTTCTTCAAGTGAAAGCAACTTTTATGGATTTTGCTCTAATCCTAGGATTTCAGTAGAGCAATACAACAGGCAAGTATTACAAGAGATGCATTATTTCAGGTATGATGAGTATGGGCGAAGTTGCAGGTCCAAAGACAAAGAGGCTGCTTCCTACCAATCTTCAGTTAACGAAGAGTGCCTGAAATACGGAGAAACTCTGGATTTAAGCAGAAACAACATATTTTTTATTAACCCCTCGGACTTCCGTGGTCTTAGTTTCCTCAGATGCCTCAACTTGTCAGGTAATGCAATAAGTCAAACTTTAAATGGAAGTGAATTCTACTATTTGTCTGGATTGAAATATCTGGATTTTTCTAACAACAGGATCGACTTGCTATACTCAACTGCTTTCAAAGAGCT

>169 TLR7

CCACCCAATTTGTCATCTACTTTAAAGGAATTGTATATTTACAATAACATGATTCAAGTGATTCAAGAACAAGATTTAAGTGCCCTTCACAACCTAGAAATTCTTGATCTGAGTGGTAATTGCCCACGTTGCTATAATGCCCCATATCCTTGTATTCCCTGCCCCAAGAGCACAATTGAGATACATTCAAAGGCTTTTTATTCCCTGAAAAATTTAAGAATTTTGCGACTTCACAGTAACTCTCTTCAGAGCATACCCAGCATCTGGTTTAAAAACATCAGAAATCTCAAAGAGCTTGACCTCTCCCAAAATTTCCTCATAAAGGAGATTGGAGATGCTCAGTTTTTGAAGTTTATCCCTGGCCTTGTAGAGCTTGATTTGTCCTTTAATTTTGAACTGCAGATGTATTCTCCATTCTTGAATCTGTCTAAGACATTTTCCTCCCTCTCTAACCTGGAAACCCTGAGGCTCAGGGGTTATGTCTTTAAAGAACTGAGAGAAGGAAATCTAGATCCATTGCTCAGTCTTAGAAATCTAACAGTGTTGGATCTCGGGCTAATTTTATTAAAGTTGCTGACCTGAAAGTGTTCAAAGAATTCCCAGCTCTTAAATTCATAGACCTCTCAGTGAATAAAATTTCTCCTTCTTCAAGTGAAAGCAACTTTTATGGATTTTGCTCTAATCCTAGGATTTCAGTAGAGCAATACAACAGGCAAGTATTACAAGAGATGCATTATTTCAGGTATGATGAGTATGGGCGAAGTTGCAGGTCCAAAGACAAAGAGGCTGCTTCCTACCAATCTTCAGTTAACGAAGAGTGCCTGAAATACGGAGAAACTCTGGATTTAAGCAGAAACAACATATTTTTTATTAACCCCTCGGACTTCCGTGGTCTTAGTTTCCTCAGATGCCTCAACTTGTCAGGTAATGCAATAAGTCAAACTTTAAATGGAAGTGAATTCTACTATTTGTCTGGATTGAAATATCTGGATTTTTCTAACAACAGGATCGACTTGCTATACTCAACTGCTTTCAAAGAGCT

>1610 TLR7

CCACCCAATTTGTCATCTACTTTAAAGGAATTGTATATTTACAATAACATGATTCAAGTGATTCAAGAACAAGATTTAAGTGCCCTTCACAACCTAGAAATTCTTGATCTGAGTGGTAATTGCCCACGTTGCTATAATGCCCCATATCCTTGTATTCCCTGCCCCAAGAGCACAATTGAGATACATTCAAAGGCTTTTTATTCCCTGAAAAATTTAAGAATTTTGCGACTTCACAGTAACTCTCTTCAGAGCATACCCAGCATCTGGTTTAAAAACATCAGAAATCTCAAAGAGCTTGACCTCTCCCAAAATTTCCTCATAAAGGAGATTGGAGATGCTCAGTTTTTGAAGTTTATCCCTGGCCTTGTAGAGCTTGATTTGTCCTTTAATTTTGAACTGCAGATGTATTCTCCATTCTTGAATCTGTCTAAGACATTTTCCTCCCTCTCTAACCTGGAAACCCTGAGGCTCAGGGGTTATGTCTTTAAAGAACTGAGAGAAGGAAATCTAGATCCATTGCTCAGTCTTAGAAATCTAACAGTGTTGGATCTCGGGCTAATTTTATTAAAGTTGCTGACCTGAAAGTGTTCAAAGAATTCCCAGCTCTTAAATTCATAGACCTCTCAGTGAATAAAATTTCTCCTTCTTCAAGTGAAAGCAACTTTTATGGATTTTGCTCTAATCCTAGGATTTCAGTAGAGCAATACAACAGGCAAGTATTACAAGAGATGCATTATTTCAGGTATGATGAGTATGGGCGAAGTTGCAGGTCCAAAGACAAAGAGGCTGCTTCCTACCAATCTTCAGTTAACGAAGAGTGCCTGAAATACGGAGAAACTCTGGATTTAAGCAGAAACAACATATTTTTTATTAACCCCTCGGACTTCCGTGGTCTTAGTTTCCTCAGATGCCTCAACTTGTCAGGTAATGCAATAAGTCAAACTTTAAATGGAAGTGAATTCTACTATTTGTCTGGATTGAAATATCTGGATTTTTCTAACAACAGGATCGACTTGCTATACTCAACTGCTTTCAAAGAGCT

>1616 TLR7

CCACCCAATTTGTCATCTACTTTAAAGGAATTGTATATTTACAATAACATGATTCAAGTGATTCAAGAACAAGATTTAAGTGCCCTTCACAACCTAGAAATTCTTGATCTGAGTGGTAATTGCCCACGTTGCTATAATGCCCCATATCCTTGTATTCCCTGCCCCAAGAGCACAATTGAGATACATTCAAAGGCTTTTTATTCCCTGAAAAATTTAAGAATTTTGCGACTTCACAGTAACTCTCTTCAGAGCATACCCAGCATCTGGTTTAAAAACATCAGAAATCTCAAAGAGCTTGACCTCTCCCAAAATTTCCTCATAAAGGAGATTGGAGATGCTCAGTTTTTGAAGTTTATCCCTGGCCTTGTAGAGCTTGATTTGTCCTTTAATTTTGAACTGCAGATGTATTCTCCATTCTTGAATCTGTCTAAGACATTTTCCTCCCTCTCTAACCTGGAAACCCTGAGGCTCAGGGGTTATGTCTTTAAAGAACTGAGAGAAGGAAATCTAGATCCATTGCTCAGTCTTAGAAATCTAACAGTGTTGGATCTCGGGCTAATTTTATTAAAGTTGCTGACCTGAAAGTGTTCAAAGAATTCCCAGCTCTTAAATTCATAGACCTCTCAGTGAATAAAATTTCTCCTTCTTCAAGTGAAAGCAACTTTTATGGATTTTGCTCTAATCCTAGGATTTCAGTAGAGCAATACAACAGGCAAGTATTACAAGAGATGCATTATTTCAGGTATGATGAGTATGGGCGAAGTTGCAGGTCCAAAGACAAAGAGGCTGCTTCCTACCAATCTTCAGTTAACGAAGAGTGCCTGAAATACGGAGAAACTCTGGATTTAAGCAGAAACAACATATTTTTTATTAACCCCTCGGACTTCCGTGGTCTTAGTTTCCTCAGATGCCTCAACTTGTCAGGTAATGCAATAAGTCAAACTTTAAATGGAAGTGAATTCTACTATTTGTCTGGATTGAAATATCTGGATTTTTCTAACAACAGGATCGACTTGCTATACTCAACTGCTTTCAAAGAGCT

>1618 TLR7

CCACCCAATTTGTCATCTACTTTAAAGGAATTGTATATTTACAATAACATGATTCAAGTGATTCAAGAACAAGATTTAAGTGCCCTTCACAACCTAGAAATTCTTGATCTGAGTGGTAATTGCCCACGTTGCTATAATGCCCCATATCCTTGTATTCCCTGCCCCAAGAGCACAATTGAGATACATTCAAAGGCTTTTTATTCCCTGAAAAATTTAAGAATTTTGCGACTTCACAGTAACTCTCTTCAGAGCATACCCAGCATCTGGTTTAAAAACATCAGAAATCTCAAAGAGCTTGACCTCTCCCAAAATTTCCTCATAAAGGAGATTGGAGATGCTCAGTTTTTGAAGTTTATCCCTGGCCTTGTAGAGCTTGATTTGTCCTTTAATTTTGAACTGCAGATGTATTCTCCATTCTTGAATCTGTCTAAGACATTTTCCTCCCTCTCTAACCTGGAAACCCTGAGGCTCAGGGGTTATGTCTTTAAAGAACTGAGAGAAGGAAATCTAGATCCATTGCTCAGTCTTAGAAATCTAACAGTGTTGGATCTCGGGCTAATTTTATTAAAGTTGCTGACCTGAAAGTGTTCAAAGAATTCCCAGCTCTTAAATTCATAGACCTCTCAGTGAATAAAATTTCTCCTTCTTCAAGTGAAAGCAACTTTTATGGATTTTGCTCTAATCCTAGGATTTCAGTAGAGCAATACAACAGGCAAGTATTACAAGAGATGCATTATTTCAGGTATGATGAGTATGGGCGAAGTTGCAGGTCCAAAGACAAAGAGGCTGCTTCCTACCAATCTTCAGTTAACGAAGAGTGCCTGAAATACGGAGAAACTCTGGATTTAAGCAGAAACAACATATTTTTTATTAACCCCTCGGACTTCCGTGGTCTTAGTTTCCTCAGATGCCTCAACTTGTCAGGTAATGCAATAAGTCAAACTTTAAATGGAAGTGAATTCTACTATTTGTCTGGATTGAAATATCTGGATTTTTCTAACAACAGGATCGACTTGCTATACTCAACTGCTTTCAAAGAGCT

>175 TLR7

CCGCCCGATTTGTCATCTACTTTAAAGGAATTGGATATTTACAATAACATGATTCAAGTGATTCAAGAACAAGATTTAAGTGCCCTTCACAACCTAGAAATTCTTGATCTGAGTGGTAATTGCCCACGTTGCTATAATGCCCCATATCCTTGTATTCCCTGCCCCAAGAGCACAATTGAGATACATTCAAAGGCTTTTTATTCCCTGAAAAATTTAAGAATTTTGCGACTTCACAGTAACTCTCTTCAGAGCATACCCAGCATCTGGTTTAAAAACATCAGAAATCTCAAAGAGCTTGACCTCTCCCAAAATTTCCTCATAAAGGAGATTGGAGATGCTCAGTTTTTGAAGTTTATCCCTGGCCTTGTAGAGCTTGATTTGTCCTTTAATTTTGAACTGCAGATGTATTCTCCATTCTTGAATCTGTCTAAGACATTTTCCTCCCTCTCTAACCTGGAAACCCTGAGGCTCAGGGGTTATGTCTTTAAAGAACTGAGAGAAGGAAATCTAGATCCATTGCTCAGTCTTAGAAATCTAACAGTGTTGGATCTCGGGCTAATTTTATTAAAGTTGCTGACCTGAAAGTGTTCAAAGAATTCCCAGCTCTTAAATTCATAGACCTCTCAGTGAATAAAATTTCTCCTTCTTCAAGTGAAAGCAACTTTTATGGATTTTGCTCTAATCCTAGGATTTCAGTAGAGCAATACAACAGGCAAGTATTACAAGAGATGCATTATTTCAGGTATGATGAGTATGGGCGAAGTTGCAGGTCCAAAGACAAAGAGGCTGCTTCCTACCAATCTTCAGTTAACGAAGAGTGCCTGAAATACGGAGAAACTCTGGATTTAAGCAGAAACAACATATTTTTTATTAACCCCTCGGACTTCCGTGGTCTTAGTTTCCTCAGATGCCTCAACTTGTCAGGTAATGCAATAAGTCAAACTTTAAATGGAAGTGAATTCTACTATTTGTCTGGATTGAAATATCTGGATTTTTCTAACAACAGGATCGACTTGCTATACTCAACTGCTTTCAAAGAGCT

>176 TLR7

CCGCCCGATTTGTCATCTACTTTAAAGGAATTGGATATTTACAATAACATGATTCAAGTGATTCAAGAACAAGATTTAAGTGCCCTTCACAACCTAGAAATTCTTGATCTGAGTGGTAATTGCCCACGTTGCTATAATGCCCCATATCCTTGTATTCCCTGCCCCAAGAGCACAATTGAGATACATTCAAAGGCTTTTTATTCCCTGAAAAATTTAAGAATTTTGCGACTTCACAGTAACTCTCTTCAGAGCATACCCAGCATCTGGTTTAAAAACATCAGAAATCTCAAAGAGCTTGACCTCTCCCAAAATTTCCTCATAAAGGAGATTGGAGATGCTCAGTTTTTGAAGTTTATCCCTGGCCTTGTAGAGCTTGATTTGTCCTTTAATTTTGAACTGCAGATGTATTCTCCATTCTTGAATCTGTCTAAGACATTTTCCTCCCTCTCTAACCTGGAAACCCTGAGGCTCAGGGGTTATGTCTTTAAAGAACTGAGAGAAGGAAATCTAGATCCATTGCTCAGTCTTAGAAATCTAACAGTGTTGGATCTCGGGCTAATTTTATTAAAGTTGCTGACCTGAAAGTGTTCAAAGAATTCCCAGCTCTTAAATTCATAGACCTCTCAGTGAATAAAATTTCTCCTTCTTCAAGTGAAAGCAACTTTTATGGATTTTGCTCTAATCCTAGGATTTCAGTAGAGCAATACAACAGGCAAGTATTACAAGAGATGCATTATTTCAGGTATGATGAGTATGGGCGAAGTTGCAGGTCCAAAGACAAAGAGGCTGCTTCCTACCAATCTTCAGTTAACGAAGAGTGCCTGAAATACGGAGAAACTCTGGATTTAAGCAGAAACAACATATTTTTTATTAACCCCTCGGACTTCCGTGGTCTTAGTTTCCTCAGATGCCTCAACTTGTCAGGTAATGCAATAAGTCAAACTTTAAATGGAAGTGAATTCTACTATTTGTCTGGATTGAAATATCTGGATTTTTCTAACAACAGGATCGACTTGCTATACTCAACTGCTTTCAAAGAGCT

>1714 TLR7

CCGCCCGATTTGTCATCTACTTTAAAGGAATTGGATATTTACAATAACATGATTCAAGTGATTCAAGAACAAGATTTAAGTGCCCTTCACAACCTAGAAATTCTTGATCTGAGTGGTAATTGCCCACGTTGCTATAATGCCCCATATCCTTGTATTCCCTGCCCCAAGAGCACAATTGAGATACATTCAAAGGCTTTTTATTCCCTGAAAAATTTAAGAATTTTGCGACTTCACAGTAACTCTCTTCAGAGCATACCCAGCATCTGGTTTAAAAACATCAGAAATCTCAAAGAGCTTGACCTCTCCCAAAATTTCCTCATAAAGGAGATTGGAGATGCTCAGTTTTTGAAGTTTATCCCTGGCCTTGTAGAGCTTGATTTGTCCTTTAATTTTGAACTGCAGATGTATTCTCCATTCTTGAATCTGTCTAAGACATTTTCCTCCCTCTCTAACCTGGAAACCCTGAGGCTCAGGGGTTATGTCTTTAAAGAACTGAGAGAAGGAAATCTAGATCCATTGCTCAGTCTTAGAAATCTAACAGTGTTGGATCTCGGGCTAATTTTATTAAAGTTGCTGACCTGAAAGTGTTCAAAGAATTCCCAGCTCTTAAATTCATAGACCTCTCAGTGAATAAAATTTCTCCTTCTTCAAGTGAAAGCAACTTTTATGGATTTTGCTCTAATCCTAGGATTTCAGTAGAGCAATACAACAGGCAAGTATTACAAGAGATGCATTATTTCAGGTATGATGAGTATGGGCGAAGTTGCAGGTCCAAAGACAAAGAGGCTGCTTCCTACCAATCTTCAGTTAACGAAGAGTGCCTGAAATACGGAGAAACTCTGGATTTAAGCAGAAACAACATATTTTTTATTAACCCCTCGGACTTCCGTGGTCTTAGTTTCCTCAGATGCCTCAACTTGTCAGGTAATGCAATAAGTCAAACTTTAAATGGAAGTGAATTCTACTATTTGTCTGGATTGAAATATCTGGATTTTTCTAACAACAGGATCGACTTGCTATACTCAACTGCTTTCAAAGAGCT

>1716 TLR7

CCACCCGATTTGTCATCTACTTTAAAGGAATTGGATATTTACAATAACATGATTCAAGTGATTCAAGAACAAGATTTAAGTGCCCTTCACAACCTAGAAATTCTTGATCTGAGTGGTAATTGCCCACGTTGCTATAATGCCCCATATCCTTGTATTCCCTGCCCCAAGAGCACAATTGAGATACATTCAAAGGCTTTTTATTCCCTGAAAAATTTAAGAATTTTGCGACTTCACAGTAACTCTCTTCAGAGCATACCCAGCATCTGGTTTAAAAACATCAGAAATCTCAAAGAGCTTGACCTCTCCCAAAATTTCCTCATAAAGGAGATTGGAGATGCTCAGTTTTTGAAGTTTATCCCTGGCCTTGTAGAGCTTGATTTGTCCTTTAATTTTGAACTGCAGATGTATTCTCCATTCTTGAATCTGTCTAAGACATTTTCCTCCCTCTCTAACCTGGAAACCCTGAGGCTCAGGGGTTATGTCTTTAAAGAACTGAGAGAAGGAAATCTAGATCCATTGCTCAGTCTTAGAAATCTAACAGTGTTGGATCTCGGGCTAATTTTATTAAAGTTGCTGACCTGAAAGTGTTCAAAGAATTCCCAGCTCTTAAATTCATAGACCTCTCAGTGAATAAAATTTCTCCTTCTTCAAGTGAAAGCAACTTTTATGGATTTTGCTCTAATCCTAGGATTTCAGTAGAGCAATACAACAGGCAAGTATTACAAGAGATGCATTATTTCAGGTATGATGAGTATGGGCGAAGTTGCAGGTCCAAAGACAAAGAGGCTGCTTCCTACCAATCTTCAGTTAACGAAGAGTGCCTGAAATACGGAGAAACTCTGGATTTAAGCAGAAACAACATATTTTTTATTAACCCCTCGGACTTCCGTGGTCTTAGTTTCCTCAGATGCCTCAACTTGTCAGGTAATGCAATAAGTCAAACTTTAAATGGAAGTGAATTCTACTATTTGTCTGGATTGAAATATCTGGATTTTTCTAACAACAGGATCGACTTGCTATACTCAACTGCTTTCAAAGAGCT

>30583 TLR7

CCACCCGATTTGTCATCTACGTTAAAGGAATTGTATATTTACAATAACATGATTCAAGTGATTCAAGAACAAGATTTAAGTGCCCTTCACAACCTAGAAATTCTTGATCTGAGTGGTAATTGCCCACGTTGCTATAATGCCCCATATCCTTGTATTCCCTGCCCCAAGAGCACAATTGAGATACATTCAAAGGCTTTTTATTCCCTGAAAAATTTAAGAATTTTGCGACTTCACAGTAACTCTCTTCAGAGCATACCCAGCATCTGGTTTAAAAACATCAGAAATCTCAAAGAGCTTGACCTCTCCCAAAATTTCCTCATAAAGGAGATTGGAGATGCTCAGTTTTTGAAGTTTATCCCTGGCCTTGTAGAGCTTGATTTGTCCTTTAATTTTGAACTGCAGATGTATTCTCCATTCTTGAATCTGTCTAAGACATTTTCCTCCCTCTCTAACCTGGAAACCCTGAGGCTCAGGGGTTATGTCTTTAAAGAACTGAGAGAAGGAAATCTAGATCCATTGCTCAGTCTTAGAAATCTAACAGTGTTGGATCTCGGGCTAATTTTATTAAAGTTGCTGACCTGAAAGTGTTCAAAGAATTCCCAGCTCTTAAATTCATAGACCTCTCAGTGAATAAAATTTCTCCTTCTTCAAGTGAAAGCAACTTTTATGGATTTTGCTCTAATCCTAGGATTTCAGTAGAGCAATACAACAGGCAAGTATTACAAGAGATGCATTATTTCAGGTATGATGAGTATGGGCGAAGTTGCAGGTCCAAAGACAAAGAGGCTGCTTCCTACCAATCTTCAGTTAACGAAGAGTGCCTGAAATACGGAGAAACTCTGGATTTAAGCAGAAACAACATATTTTTTATTAACCCCTCGGACTTCCGTGGTCTTAGTTTCCTCAGATGCCTCAACTTGTCAGGTAATGCAATAAGTCAAACTTTAAATGGAAGTGAATTCTACTATTTGTCTGGATTGAAATATCTGGATTTTTCTAACAACAGGATCGACTTGCTATACTCAACTGCTTTCAAAGAGCT

>30585 TLR7

CCACCCAATTTGTCATCTACTTTAAAGGAATTGTATATTTACAATAACATGATTCAAGTGATTCAAGAACAAGATTTAAGTGCCCTTCACAACCTAGAAATTCTTGATCTGAGTGGTAATTGCCCACGTTGCTATAATGCCCCATATCCTTGTATTCCCTGCCCCAAGAGCACAATTGAGATACATTCAAAGGCTTTTTATTCCCTGAAAAATTTAAGAATTTTGCGACTTCACAGTAACTCTCTTCAGAGCATACCCAGCATCTGGTTTAAAAACATCAGAAATCTCAAAGAGCTTGACCTCTCCCAAAATTTCCTCATAAAGGAGATTGGAGATGCTCAGTTTTTGAAGTTTATCCCTGGCCTTGTAGAGCTTGATTTGTCCTTTAATTTTGAACTGCAGATGTATTCTCCATTCTTGAATCTGTCTAAGACATTTTCCTCCCTCTCTAACCTGGAAACCCTGAGGCTCAGGGGTTATGTCTTTAAAGAACTGAGAGAAGGAAATCTAGATCCATTGCTCAGTCTTAGAAATCTAACAGTGTTGGATCTCGGGCTAATTTTATTAAAGTTGCTGACCTGAAAGTGTTCAAAGAATTCCCAGCTCTTAAATTCATAGACCTCTCAGTGAATAAAATTTCTCCTTCTTCAAGTGAAAGCAACTTTTATGGATTTTGCTCTAATCCTAGGATTTCAGTAGAGCAATACAACAGGCAAGTATTACAAGAGATGCATTATTTCAGGTATGATGAGTATGGGCGAAGTTGCAGGTCCAAAGACAAAGAGGCTGCTTCCTACCAATCTTCAGTTAACGAAGAGTGCCTGAAATACGGAGAAACTCTGGATTTAAGCAGAAACAACATATTTTTTATTAACCCCTCGGACTTCCGTGGTCTTAGTTTCCTCAGATGCCTCAACTTGTCAGGTAATGCAATAAGTCAAACTTTAAATGGAAGTGAATTCTACTATTTGTCTGGATTGAAATATCTGGATTTTTCTAACAACAGGATCGACTTGCTATACTCAACTGCTTTCAAAGAGCT

>30586 TLR7

CCACCCAATTTGTCATCTACTTTAAAGGAATTGTATATTTACAATAACATGATTCAAGTGATTCAAGAACAAGATTTAAGTGCCCTTCACAACCTAGAAATTCTTGATCTGAGTGGTAATTGCCCACGTTGCTATAATGCCCCATATCCTTGTATTCCCTGCCCCAAGAGCACAATTGAGATACATTCAAAGGCTTTTTATTCCCTGAAAAATTTAAGAATTTTGCGACTTCACAGTAACTCTCTTCAGAGCATACCCAGCATCTGGTTTAAAAACATCAGAAATCTCAAAGAGCTTGACCTCTCCCAAAATTTCCTCATAAAGGAGATTGGAGATGCTCAGTTTTTGAAGTTTATCCCTGGCCTTGTAGAGCTTGATTTGTCCTTTAATTTTGAACTGCAGATGTATTCTCCATTCTTGAATCTGTCTAAGACATTTTCCTCCCTCTCTAACCTGGAAACCCTGAGGCTCAGGGGTTATGTCTTTAAAGAACTGAGAGAAGGAAATCTAGATCCATTGCTCAGTCTTAGAAATCTAACAGTGTTGGATCTCGGGCTAATTTTATTAAAGTTGCTGACCTGAAAGTGTTCAAAGAATTCCCAGCTCTTAAATTCATAGACCTCTCAGTGAATAAAATTTCTCCTTCTTCAAGTGAAAGCAACTTTTATGGATTTTGCTCTAATCCTAGGATTTCAGTAGAGCAATACAACAGGCAAGTATTACAAGAGATGCATTATTTCAGGTATGATGAGTATGGGCGAAGTTGCAGGTCCAAAGACAAAGAGGCTGCTTCCTACCAATCTTCAGTTAACGAAGAGTGCCTGAAATACGGAGAAACTCTGGATTTAAGCAGAAACAACATATTTTTTATTAACCCCTCGGACTTCCGTGGTCTTAGTTTCCTCAGATGCCTCAACTTGTCAGGTAATGCAATAAGTCAAACTTTAAATGGAAGTGAATTCTACTATTTGTCTGGATTGAAATATCTGGATTTTTCTAACAACAGGATCGACTTGCTATACTCAACTGCTTTCAAAGAGCT

>30588 TLR7

CCACCCGATTTGTCATCTACTTTAAAGGAAGTGTATATTTACAATAACATGATTCAAGTGATTCAGGAACAAGATTTAAGTGCCCTTCACAACCTAGAAATTCTTGATCTGAGTGGTAATTGCCCACGTTGCTATAATGCCCCATATCCTTGTATTCCCTGCCCCAAGAGCACAATTGAGATACATTCAAAGGCTTTTTATTCCCTGAAAAATTTAAGAATTTTGCGACTTCACAGTAACTCTCTTCAGAGCATACCCAGCATCTGGTTTAAAAACATCAGAAATCTCAAAGAGCTTGACCTCTCCCAAAATTTCCTCATAAAGGAGATTGGAGATGCTCAGTTTTTGAAGTTTATCCCTGGCCTTGTAGAGCTTGATTTGTCCTTTAATTTTGAACTGCAGATGTATTCTCCATTCTTGAATCTGTCTAAGACATTTTCCTCCCTCTCTAACCTGGAAACCCTGAGGCTCAGGGGTTATGTCTTTAAAGAACTGAGAGAAGGAAATCTAGATCCATTGCTCAGTCTTAGAAATCTAACAGTGTTGGATCTCGGGCTAATTTTATTAAAGTTGCTGACCTGAAAGTGTTCAAAGAATTCCCAGCTCTTAAATTCATAGACCTCTCAGTGAATAAAATTTCTCCTTCTTCAAGTGAAAGCAACTTTTATGGATTTTGCTCTAATCCTAGGATTTCAGTAGAGCAATACAACAGGCAAGTATTACAAGAGATGCATTATTTCAGGTATGATGAGTATGGGCGAAGTTGCAGGTCCAAAGACAAAGAGGCTGCTTCCTACCAATCTTCAGTTAACGAAGAGTGCCTGAAATACGGAGAAACTCTGGATTTAAGCAGAAACAACATATTTTTTATTAACCCCTCGGACTTCCGTGGTCTTAGTTTCCTCAGATGCCTCAACTTGTCAGGTAATGCAATAAGTCAAACTTTAAATGGAAGTGAATTCTACTATTTGTCTGGATTGAAATATCTGGATTTTTCTAACAACAGGATCGACTTGCTATACTCAACTGCTTTCAAAGAGCT

>30589 TLR7

CCACCCAATTTGTCATCTACTTTAAAGGAATTGTATATTTACAATAACATGATTCAAGTGATTCAAGAACAAGATTTAAGTGCCCTTCACAACCTAGAAATTCTTGATCTGAGTGGTAATTGCCCACGTTGCTATAATGCCCCATATCCTTGTATTCCCTGCCCCAAGAGCACAATTGAGATACATTCAAAGGCTTTTTATTCCCTGAAAAATTTAAGAATTTTGCGACTTCACAGTAACTCTCTTCAGAGCATACCCAGCATCTGGTTTAAAAACATCAGAAATCTCAAAGAGCTTGACCTCTCCCAAAATTTCCTCATAAAGGAGATTGGAGATGCTCAGTTTTTGAAGTTTATCCCTGGCCTTGTAGAGCTTGATTTGTCCTTTAATTTTGAACTGCAGATGTATTCTCCATTCTTGAATCTGTCTAAGACATTTTCCTCCCTCTCTAACCTGGAAACCCTGAGGCTCAGGGGTTATGTCTTTAAAGAACTGAGAGAAGGAAATCTAGATCCATTGCTCAGTCTTAGAAATCTAACAGTGTTGGATCTCGGGCTAATTTTATTAAAGTTGCTGACCTGAAAGTGTTCAAAGAATTCCCAGCTCTTAAATTCATAGACCTCTCAGTGAATAAAATTTCTCCTTCTTCAAGTGAAAGCAACTTTTATGGATTTTGCTCTAATCCTAGGATTTCAGTAGAGCAATACAACAGGCAAGTATTACAAGAGATGCATTATTTCAGGTATGATGAGTATGGGCGAAGTTGCAGGTCCAAAGACAAAGAGGCTGCTTCCTACCAATCTTCAGTTAACGAAGAGTGCCTGAAATACGGAGAAACTCTGGATTTAAGCAGAAACAACATATTTTTTATTAACCCCTCGGACTTCCGTGGTCTTAGTTTCCTCAGATGCCTCAACTTGTCAGGTAATGCAATAAGTCAAACTTTAAATGGAAGTGAATTCTACTATTTGTCTGGATTGAAATATCTGGATTTTTCTAACAACAGGATCGACTTGCTATACTCAACTGCTTTCAAAGAGCT

>30590 TLR7

CCACCCGATTTGTCATCTACGTTAAAGGAATTGGATATTTACAATAACATGATTCAAGTGATTCAGGAACAAGATTTAAGTGCCCTTCACAACCTAGAAATTCTTGATCTGAGTGGTAATTGCCCACGTTGCTATAATGCCCCATATCCTTGTATTCCCTGCCCCAAGAGCACAATTGAGATACATTCAAAGGCTTTTTATTCCCTGAAAAATTTAAGAATTTTGCGACTTCACAGTAACTCTCTTCAGAGCATACCCAGCATCTGGTTTAAAAACATCAGAAATCTCAAAGAGCTTGACCTCTCCCAAAATTTCCTCATAAAGGAGATTGGAGATGCTCAGTTTTTGAAGTTTATCCCTGGCCTTGTAGAGCTTGATTTGTCCTTTAATTTTGAACTGCAGATGTATTCTCCATTCTTGAATCTGTCTAAGACATTTTCCTCCCTCTCTAACCTGGAAACCCTGAGGCTCAGGGGTTATGTCTTTAAAGAACTGAGAGAAGGAAATCTAGATCCATTGCTCAGTCTTAGAAATCTAACAGTGTTGGATCTCGGGCTAATTTTATTAAAGTTGCTGACCTGAAAGTGTTCAAAGAATTCCCAGCTCTTAAATTCATAGACCTCTCAGTGAATAAAATTTCTCCTTCTTCAAGTGAAAGCAACTTTTATGGATTTTGCTCTAATCCTAGGATTTCAGTAGAGCAATACAACAGGCAAGTATTACAAGAGATGCATTATTTCAGGTATGATGAGTATGGGCGAAGTTGCAGGTCCAAAGACAAAGAGGCTGCTTCCTACCAATCTTCAGTTAACGAAGAGTGCCTGAAATACGGAGAAACTCTGGATTTAAGCAGAAACAACATATTTTTTATTAACCCCTCGGACTTCCGTGGTCTTAGTTTCCTCAGATGCCTCAACTTGTCAGGTAATGCAATAAGTCAAACTTTAAATGGAAGTGAATTCTACTATTTGTCTGGATTGAAATATCTGGATTTTTCTAACAACAGGATCGACTTGCTATACTCAACTGCTTTCAAAGAGCT

>30591 TLR7

CCACCCAATTTGTCATCTACTTTAAAGGAATTGTATATTTACAATAACATGATTCAAGTGATTCAAGAACAAGATTTAAGTGCCCTTCACAACCTAGAAATTCTTGATCTGAGTGGTAATTGCCCACGTTGCTATAATGCCCCATATCCTTGTATTCCCTGCCCCAAGAGCACAATTGAGATACATTCAAAGGCTTTTTATTCCCTGAAAAATTTAAGAATTTTGCGACTTCACAGTAACTCTCTTCAGAGCATACCCAGCATCTGGTTTAAAAACATCAGAAATCTCAAAGAGCTTGACCTCTCCCAAAATTTCCTCATAAAGGAGATTGGAGATGCTCAGTTTTTGAAGTTTATCCCTGGCCTTGTAGAGCTTGATTTGTCCTTTAATTTTGAACTGCAGATGTATTCTCCATTCTTGAATCTGTCTAAGACATTTTCCTCCCTCTCTAACCTGGAAACCCTGAGGCTCAGGGGTTATGTCTTTAAAGAACTGAGAGAAGGAAATCTAGATCCATTGCTCAGTCTTAGAAATCTAACAGTGTTGGATCTCGGGCTAATTTTATTAAAGTTGCTGACCTGAAAGTGTTCAAAGAATTCCCAGCTCTTAAATTCATAGACCTCTCAGTGAATAAAATTTCTCCTTCTTCAAGTGAAAGCAACTTTTATGGATTTTGCTCTAATCCTAGGATTTCAGTAGAGCAATACAACAGGCAAGTATTACAAGAGATGCATTATTTCAGGTATGATGAGTATGGGCGAAGTTGCAGGTCCAAAGACAAAGAGGCTGCTTCCTACCAATCTTCAGTTAACGAAGAGTGCCTGAAATACGGAGAAACTCTGGATTTAAGCAGAAACAACATATTTTTTATTAACCCCTCGGACTTCCGTGGTCTTAGTTTCCTCAGATGCCTCAACTTGTCAGGTAATGCAATAAGTCAAACTTTAAATGGAAGTGAATTCTACTATTTGTCTGGATTGAAATATCTGGATTTTTCTAACAACAGGATCGACTTGCTATACTCAACTGCTTTCAAAGAGCT

>30701 TLR7

CCACCCAATTTGTCATCTACTTTAAAGGAATTGTATATTTACAATAACATGATTCAAGTGATTCAAGAACAAGATTTAAGTGCCCTTCACAACCTAGAAATTCTTGATCTGAGTGGTAATTGCCCACGTTGCTATAATGCCCCATATCCTTGTATTCCCTGCCCCAAGAGCACAATTGAGATACATTCAAAGGCTTTTTATTCCCTGAAAAATTTAAGAATTTTGCGACTTCACAGTAACTCTCTTCAGAGCATACCCAGCATCTGGTTTAAAAACATCAGAAATCTCAAAGAGCTTGACCTCTCCCAAAATTTCCTCATAAAGGAGATTGGAGATGCTCAGTTTTTGAAGTTTATCCCTGGCCTTGTAGAGCTTGATTTGTCCTTTAATTTTGAACTGCAGATGTATTCTCCATTCTTGAATCTGTCTAAGACATTTTCCTCCCTCTCTAACCTGGAAACCCTGAGGCTCAGGGGTTATGTCTTTAAAGAACTGAGAGAAGGAAATCTAGATCCATTGCTCAGTCTTAGAAATCTAACAGTGTTGGATCTCGGGCTAATTTTATTAAAGTTGCTGACCTGAAAGTGTTCAAAGAATTCCCAGCTCTTAAATTCATAGACCTCTCAGTGAATAAAATTTCTCCTTCTTCAAGTGAAAGCAACTTTTATGGATTTTGCTCTAATCCTAGGATTTCAGTAGAGCAATACAACAGGCAAGTATTACAAGAGATGCATTATTTCAGGTATGATGAGTATGGGCGAAGTTGCAGGTCCAAAGACAAAGAGGCTGCTTCCTACCAATCTTCAGTTAACGAAGAGTGCCTGAAATACGGAGAAACTCTGGATTTAAGCAGAAACAACATATTTTTTATTAACCCCTCGGACTTCCGTGGTCTTAGTTTCCTCAGATGCCTCAACTTGTCAGGTAATGCAATAAGTCAAACTTTAAATGGAAGTGAATTCTACTATTTGTCTGGATTGAAATATCTGGATTTTTCTAACAACAGGATCGACTTGCTATACTCAACTGCTTTCAAAGAGCT

>30703 TLR7

CCACCCAATTTGTCATCTACTTTAAAGGAATTGTATATTTACAATAACATGATTCAAGTGATTCAAGAACAAGATTTAAGTGCCCTTCACAACCTAGAAATTCTTGATCTGAGTGGTAATTGCCCACGTTGCTATAATGCCCCATATCCTTGTATTCCCTGCCCCAAGAGCACAATTGAGATACATTCAAAGGCTTTTTATTCCCTGAAAAATTTAAGAATTTTGCGACTTCACAGTAACTCTCTTCAGAGCATACCCAGCATCTGGTTTAAAAACATCAGAAATCTCAAAGAGCTTGACCTCTCCCAAAATTTCCTCATAAAGGAGATTGGAGATGCTCAGTTTTTGAAGTTTATCCCTGGCCTTGTAGAGCTTGATTTGTCCTTTAATTTTGAACTGCAGATGTATTCTCCATTCTTGAATCTGTCTAAGACATTTTCCTCCCTCTCTAACCTGGAAACCCTGAGGCTCAGGGGTTATGTCTTTAAAGAACTGAGAGAAGGAAATCTAGATCCATTGCTCAGTCTTAGAAATCTAACAGTGTTGGATCTCGGGCTAATTTTATTAAAGTTGCTGACCTGAAAGTGTTCAAAGAATTCCCAGCTCTTAAATTCATAGACCTCTCAGTGAATAAAATTTCTCCTTCTTCAAGTGAAAGCAACTTTTATGGATTTTGCTCTAATCCTAGGATTTCAGTAGAGCAATACAACAGGCAAGTATTACAAGAGATGCATTATTTCAGGTATGATGAGTATGGGCGAAGTTGCAGGTCCAAAGACAAAGAGGCTGCTTCCTACCAATCTTCAGTTAACGAAGAGTGCCTGAAATACGGAGAAACTCTGGATTTAAGCAGAAACAACATATTTTTTATTAACCCCTCGGACTTCCGTGGTCTTAGTTTCCTCAGATGCCTCAACTTGTCAGGTAATGCAATAAGTCAAACTTTAAATGGAAGTGAATTCTACTATTTGTCTGGATTGAAATATCTGGATTTTTCTAACAACAGGATCGACTTGCTATACTCAACTGCTTTCAAAGAGCT

>30704 TLR7

CCACCCAATTTGTCATCTACTTTAAAGGAATTGTATATTTACAATAACATGATTCAAGTGATTCAAGAACAAGATTTAAGTGCCCTTCACAACCTAGAAATTCTTGATCTGAGTGGTAATTGCCCACGTTGCTATAATGCCCCATATCCTTGTATTCCCTGCCCCAAGAGCACAATTGAGATACATTCAAAGGCTTTTTATTCCCTGAAAAATTTAAGAATTTTGCGACTTCACAGTAACTCTCTTCAGAGCATACCCAGCATCTGGTTTAAAAACATCAGAAATCTCAAAGAGCTTGACCTCTCCCAAAATTTCCTCATAAAGGAGATTGGAGATGCTCAGTTTTTGAAGTTTATCCCTGGCCTTGTAGAGCTTGATTTGTCCTTTAATTTTGAACTGCAGATGTATTCTCCATTCTTGAATCTGTCTAAGACATTTTCCTCCCTCTCTAACCTGGAAACCCTGAGGCTCAGGGGTTATGTCTTTAAAGAACTGAGAGAAGGAAATCTAGATCCATTGCTCAGTCTTAGAAATCTAACAGTGTTGGATCTCGGGCTAATTTTATTAAAGTTGCTGACCTGAAAGTGTTCAAAGAATTCCCAGCTCTTAAATTCATAGACCTCTCAGTGAATAAAATTTCTCCTTCTTCAAGTGAAAGCAACTTTTATGGATTTTGCTCTAATCCTAGGATTTCAGTAGAGCAATACAACAGGCAAGTATTACAAGAGATGCATTATTTCAGGTATGATGAGTATGGGCGAAGTTGCAGGTCCAAAGACAAAGAGGCTGCTTCCTACCAATCTTCAGTTAACGAAGAGTGCCTGAAATACGGAGAAACTCTGGATTTAAGCAGAAACAACATATTTTTTATTAACCCCTCGGACTTCCGTGGTCTTAGTTTCCTCAGATGCCTCAACTTGTCAGGTAATGCAATAAGTCAAACTTTAAATGGAAGTGAATTCTACTATTTGTCTGGATTGAAATATCTGGATTTTTCTAACAACAGGATCGACTTGCTATACTCAACTGCTTTCAAAGAGCT

>30706 TLR7

CCACCCAATTTGTCATCTACTTTAAAGGAATTGTATATTTACAATAACATGATTCAAGTGATTCAAGAACAAGATTTAAGTGCCCTTCACAACCTAGAAATTCTTGATCTGAGTGGTAATTGCCCACGTTGCTATAATGCCCCATATCCTTGTATTCCCTGCCCCAAGAGCACAATTGAGATACATTCAAAGGCTTTTTATTCCCTGAAAAATTTAAGAATTTTGCGACTTCACAGTAACTCTCTTCAGAGCATACCCAGCATCTGGTTTAAAAACATCAGAAATCTCAAAGAGCTTGACCTCTCCCAAAATTTCCTCATAAAGGAGATTGGAGATGCTCAGTTTTTGAAGTTTATCCCTGGCCTTGTAGAGCTTGATTTGTCCTTTAATTTTGAACTGCAGATGTATTCTCCATTCTTGAATCTGTCTAAGACATTTTCCTCCCTCTCTAACCTGGAAACCCTGAGGCTCAGGGGTTATGTCTTTAAAGAACTGAGAGAAGGAAATCTAGATCCATTGCTCAGTCTTAGAAATCTAACAGTGTTGGATCTCGGGCTAATTTTATTAAAGTTGCTGACCTGAAAGTGTTCAAAGAATTCCCAGCTCTTAAATTCATAGACCTCTCAGTGAATAAAATTTCTCCTTCTTCAAGTGAAAGCAACTTTTATGGATTTTGCTCTAATCCTAGGATTTCAGTAGAGCAATACAACAGGCAAGTATTACAAGAGATGCATTATTTCAGGTATGATGAGTATGGGCGAAGTTGCAGGTCCAAAGACAAAGAGGCTGCTTCCTACCAATCTTCAGTTAACGAAGAGTGCCTGAAATACGGAGAAACTCTGGATTTAAGCAGAAACAACATATTTTTTATTAACCCCTCGGACTTCCGTGGTCTTAGTTTCCTCAGATGCCTCAACTTGTCAGGTAATGCAATAAGTCAAACTTTAAATGGAAGTGAATTCTACTATTTGTCTGGATTGAAATATCTGGATTTTTCTAACAACAGGATCGACTTGCTATACTCAACTGCTTTCAAAGAGCT

>30708 TLR7

CCACCCAATTTGTCATCTACTTTAAAGGAATTGTATATTTACAATAACATGATTCAAGTGATTCAAGAACAAGATTTAAGTGCCCTTCACAACCTAGAAATTCTTGATCTGAGTGGTAATTGCCCACGTTGCTATAATGCCCCATATCCTTGTATTCCCTGCCCCAAGAGCACAATTGAGATACATTCAAAGGCTTTTTATTCCCTGAAAAATTTAAGAATTTTGCGACTTCACAGTAACTCTCTTCAGAGCATACCCAGCATCTGGTTTAAAAACATCAGAAATCTCAAAGAGCTTGACCTCTCCCAAAATTTCCTCATAAAGGAGATTGGAGATGCTCAGTTTTTGAAGTTTATCCCTGGCCTTGTAGAGCTTGATTTGTCCTTTAATTTTGAACTGCAGATGTATTCTCCATTCTTGAATCTGTCTAAGACATTTTCCTCCCTCTCTAACCTGGAAACCCTGAGGCTCAGGGGTTATGTCTTTAAAGAACTGAGAGAAGGAAATCTAGATCCATTGCTCAGTCTTAGAAATCTAACAGTGTTGGATCTCGGGCTAATTTTATTAAAGTTGCTGACCTGAAAGTGTTCAAAGAATTCCCAGCTCTTAAATTCATAGACCTCTCAGTGAATAAAATTTCTCCTTCTTCAAGTGAAAGCAACTTTTATGGATTTTGCTCTAATCCTAGGATTTCAGTAGAGCAATACAACAGGCAAGTATTACAAGAGATGCATTATTTCAGGTATGATGAGTATGGGCGAAGTTGCAGGTCCAAAGACAAAGAGGCTGCTTCCTACCAATCTTCAGTTAACGAAGAGTGCCTGAAATACGGAGAAACTCTGGATTTAAGCAGAAACAACATATTTTTTATTAACCCCTCGGACTTCCGTGGTCTTAGTTTCCTCAGATGCCTCAACTTGTCAGGTAATGCAATAAGTCAAACTTTAAATGGAAGTGAATTCTACTATTTGTCTGGATTGAAATATCTGGATTTTTCTAACAACAGGATCGACTTGCTATACTCAACTGCTTTCAAAGAGCT

>30710 TLR7

CCACCCGATTTGTCATCTACTTTAAAGGAATTGTATATTTACAATAACATGATTCAAGTGATTCAAGAACAAGATTTAAGTGCCCTTCACAACCTAGAAATTCTTGATCTGAGTGGTAATTGCCCACGTTGCTATAATGCCCCATATCCTTGTATTCCCTGCCCCAAGAGCACAATTGAGATACATTCAAAGGCTTTTTATTCCCTGAAAAATTTAAGAATTTTGCGACTTCACAGTAACTCTCTTCAGAGCATACCCAGCATCTGGTTTAAAAACATCAGAAATCTCAAAGAGCTTGACCTCTCCCAAAATTTCCTCATAAAGGAGATTGGAGATGCTCAGTTTTTGAAGTTTATCCCTGGCCTTGTAGAGCTTGATTTGTCCTTTAATTTTGAACTGCAGATGTATTCTCCATTCTTGAATCTGTCTAAGACATTTTCCTCCCTCTCTAACCTGGAAACCCTGAGGCTCAGGGGTTATGTCTTTAAAGAACTGAGAGAAGGAAATCTAGATCCATTGCTCAGTCTTAGAAATCTAACAGTGTTGGATCTCGGGCTAATTTTATTAAAGTTGCTGACCTGAAAGTGTTCAAAGAATTCCCAGCTCTTAAATTCATAGACCTCTCAGTGAATAAAATTTCTCCTTCTTCAAGTGAAAGCAACTTTTATGGATTTTGCTCTAATCCTAGGATTTCAGTAGAGCAATACAACAGGCAAGTATTACAAGAGATGCATTATTTCAGGTATGATGAGTATGGGCGAAGTTGCAGGTCCAAAGACAAAGAGGCTGCTTCCTACCAATCTTCAGTTAACGAAGAGTGCCTGAAATACGGAGAAACTCTGGATTTAAGCAGAAACAACATATTTTTTATTAACCCCTCGGACTTCCGTGGTCTTAGTTTCCTCAGATGCCTCAACTTGTCAGGTAATGCAATAAGTCAAACTTTAAATGGAAGTGAATTCTACTATTTGTCTGGATTGAAATATCTGGATTTTTCTAACAACAGGATCGACTTGCTATACTCAACTGCTTTCAAAGAGCT

>30711 TLR7

CCACCCAATTTGTCATCTACTTTAAAGGAATTGTATATTTACAATAACATGATTCAAGTGATTCAAGAACAAGATTTAAGTGCCCTTCACAACCTAGAAATTCTTGATCTGAGTGGTAATTGCCCACGTTGCTATAATGCCCCATATCCTTGTATTCCCTGCCCCAAGAGCACAATTGAGATACATTCAAAGGCTTTTTATTCCCTGAAAAATTTAAGAATTTTGCGACTTCACAGTAACTCTCTTCAGAGCATACCCAGCATCTGGTTTAAAAACATCAGAAATCTCAAAGAGCTTGACCTCTCCCAAAATTTCCTCATAAAGGAGATTGGAGATGCTCAGTTTTTGAAGTTTATCCCTGGCCTTGTAGAGCTTGATTTGTCCTTTAATTTTGAACTGCAGATGTATTCTCCATTCTTGAATCTGTCTAAGACATTTTCCTCCCTCTCTAACCTGGAAACCCTGAGGCTCAGGGGTTATGTCTTTAAAGAACTGAGAGAAGGAAATCTAGATCCATTGCTCAGTCTTAGAAATCTAACAGTGTTGGATCTCGGGCTAATTTTATTAAAGTTGCTGACCTGAAAGTGTTCAAAGAATTCCCAGCTCTTAAATTCATAGACCTCTCAGTGAATAAAATTTCTCCTTCTTCAAGTGAAAGCAACTTTTATGGATTTTGCTCTAATCCTAGGATTTCAGTAGAGCAATACAACAGGCAAGTATTACAAGAGATGCATTATTTCAGGTATGATGAGTATGGGCGAAGTTGCAGGTCCAAAGACAAAGAGGCTGCTTCCTACCAATCTTCAGTTAACGAAGAGTGCCTGAAATACGGAGAAACTCTGGATTTAAGCAGAAACAACATATTTTTTATTAACCCCTCGGACTTCCGTGGTCTTAGTTTCCTCAGATGCCTCAACTTGTCAGGTAATGCAATAAGTCAAACTTTAAATGGAAGTGAATTCTACTATTTGTCTGGATTGAAATATCTGGATTTTTCTAACAACAGGATCGACTTGCTATACTCAACTGCTTTCAAAGAGCT

>46151 TLR7

CCACCCAATTTGTCATCTACTTTAAAGGAATTGTATATTTACAATAACATGATTCAAGTGATTCAAGAACAAGATTTAAGTGCCCTTCACAACCTAGAAATTCTTGATCTGAGTGGTAATTGCCCACGTTGCTATAATGCCCCATATCCTTGTATTCCCTGCCCCAAGAGCACAATTGAGATACATTCAAAGGCTTTTTATTCCCTGAAAAATTTAAGAATTTTGCGACTTCACAGTAACTCTCTTCAGAGCATACCCAGCATCTGGTTTAAAAACATCAGAAATCTCAAAGAGCTTGACCTCTCCCAAAATTTCCTCATAAAGGAGATTGGAGATGCTCAGTTTTTGAAGTTTATCCCTGGCCTTGTAGAGCTTGATTTGTCCTTTAATTTTGAACTGCAGATGTATTCTCCATTCTTGAATCTGTCTAAGACATTTTCCTCCCTCTCTAACCTGGAAACCCTGAGGCTCAGGGGTTATGTCTTTAAAGAACTGAGAGAAGGAAATCTAGATCCATTGCTCAGTCTTAGAAATCTAACAGTGTTGGATCTCGGGCTAATTTTATTAAAGTTGCTGACCTGAAAGTGTTCAAAGAATTCCCAGCTCTTAAATTCATAGACCTCTCAGTGAATAAAATTTCTCCTTCTTCAAGTGAAAGCAACTTTTATGGATTTTGCTCTAATCCTAGGATTTCAGTAGAGCAATACAACAGGCAAGTATTACAAGAGATGCATTATTTCAGGTATGATGAGTATGGGCGAAGTTGCAGGTCCAAAGACAAAGAGGCTGCTTCCTACCAATCTTCAGTTAACGAAGAGTGCCTGAAATACGGAGAAACTCTGGATTTAAGCAGAAACAACATATTTTTTATTAACCCCTCGGACTTCCGTGGTCTTAGTTTCCTCAGATGCCTCAACTTGTCAGGTAATGCAATAAGTCAAACTTTAAATGGAAGTGAATTCTACTATTTGTCTGGATTGAAATATCTGGATTTTTCTAACAACAGGATCGACTTGCTATACTCAACTGCTTTCAAAGAGCT

>46152 TLR7

CCACCCAATTTGTCATCTACTTTAAAGGAATTGTATATTTACAATAACATGATTCAAGTGATTCAAGAACAAGATTTAAGTGCCCTTCACAACCTAGAAATTCTTGATCTGAGTGGTAATTGCCCACGTTGCTATAATGCCCCATATCCTTGTATTCCCTGCCCCAAGAGCACAATTGAGATACATTCAAAGGCTTTTTATTCCCTGAAAAATTTAAGAATTTTGCGACTTCACAGTAACTCTCTTCAGAGCATACCCAGCATCTGGTTTAAAAACATCAGAAATCTCAAAGAGCTTGACCTCTCCCAAAATTTCCTCATAAAGGAGATTGGAGATGCTCAGTTTTTGAAGTTTATCCCTGGCCTTGTAGAGCTTGATTTGTCCTTTAATTTTGAACTGCAGATGTATTCTCCATTCTTGAATCTGTCTAAGACATTTTCCTCCCTCTCTAACCTGGAAACCCTGAGGCTCAGGGGTTATGTCTTTAAAGAACTGAGAGAAGGAAATCTAGATCCATTGCTCAGTCTTAGAAATCTAACAGTGTTGGATCTCGGGCTAATTTTATTAAAGTTGCTGACCTGAAAGTGTTCAAAGAATTCCCAGCTCTTAAATTCATAGACCTCTCAGTGAATAAAATTTCTCCTTCTTCAAGTGAAAGCAACTTTTATGGATTTTGCTCTAATCCTAGGATTTCAGTAGAGCAATACAACAGGCAAGTATTACAAGAGATGCATTATTTCAGGTATGATGAGTATGGGCGAAGTTGCAGGTCCAAAGACAAAGAGGCTGCTTCCTACCAATCTTCAGTTAACGAAGAGTGCCTGAAATACGGAGAAACTCTGGATTTAAGCAGAAACAACATATTTTTTATTAACCCCTCGGACTTCCGTGGTCTTAGTTTCCTCAGATGCCTCAACTTGTCAGGTAATGCAATAAGTCAAACTTTAAATGGAAGTGAATTCTACTATTTGTCTGGATTGAAATATCTGGATTTTTCTAACAACAGGATCGACTTGCTATACTCAACTGCTTTCAAAGAGCT

>46153 TLR7

CCACCCAATTTGTCATCTACTTTAAAGGAATTGTATATTTACAATAACATGATTCAAGTGATTCAAGAACAAGATTTAAGTGCCCTTCACAACCTAGAAATTCTTGATCTGAGTGGTAATTGCCCACGTTGCTATAATGCCCCATATCCTTGTATTCCCTGCCCCAAGAGCACAATTGAGATACATTCAAAGGCTTTTTATTCCCTGAAAAATTTAAGAATTTTGCGACTTCACAGTAACTCTCTTCAGAGCATACCCAGCATCTGGTTTAAAAACATCAGAAATCTCAAAGAGCTTGACCTCTCCCAAAATTTCCTCATAAAGGAGATTGGAGATGCTCAGTTTTTGAAGTTTATCCCTGGCCTTGTAGAGCTTGATTTGTCCTTTAATTTTGAACTGCAGATGTATTCTCCATTCTTGAATCTGTCTAAGACATTTTCCTCCCTCTCTAACCTGGAAACCCTGAGGCTCAGGGGTTATGTCTTTAAAGAACTGAGAGAAGGAAATCTAGATCCATTGCTCAGTCTTAGAAATCTAACAGTGTTGGATCTCGGGCTAATTTTATTAAAGTTGCTGACCTGAAAGTGTTCAAAGAATTCCCAGCTCTTAAATTCATAGACCTCTCAGTGAATAAAATTTCTCCTTCTTCAAGTGAAAGCAACTTTTATGGATTTTGCTCTAATCCTAGGATTTCAGTAGAGCAATACAACAGGCAAGTATTACAAGAGATGCATTATTTCAGGTATGATGAGTATGGGCGAAGTTGCAGGTCCAAAGACAAAGAGGCTGCTTCCTACCAATCTTCAGTTAACGAAGAGTGCCTGAAATACGGAGAAACTCTGGATTTAAGCAGAAACAACATATTTTTTATTAACCCCTCGGACTTCCGTGGTCTTAGTTTCCTCAGATGCCTCAACTTGTCAGGTAATGCAATAAGTCAAACTTTAAATGGAAGTGAATTCTACTATTTGTCTGGATTGAAATATCTGGATTTTTCTAACAACAGGATCGACTTGCTATACTCAACTGCTTTCAAAGAGCT

>46154 TLR7

CCGCCCAATTTGTCATCTACTTTAAAGGAATTGTATATTTACAATAACATGATTCAAGTGATTCAAGAACAAGATTTAAGTGCCCTTCACAACCTAGAAATTCTTGATCTGAGTGGTAATTGCCCACGTTGCTATAATGCCCCATATCCTTGTATTCCCTGCCCCAAGAGCACAATTGAGATACATTCAAAGGCTTTTTATTCCCTGAAAAATTTAAGAATTTTGCGACTTCACAGTAACTCTCTTCAGAGCATACCCAGCATCTGGTTTAAAAACATCAGAAATCTCAAAGAGCTTGACCTCTCCCAAAATTTCCTCATAAAGGAGATTGGAGATGCTCAGTTTTTGAAGTTTATCCCTGGCCTTGTAGAGCTTGATTTGTCCTTTAATTTTGAACTGCAGATGTATTCTCCATTCTTGAATCTGTCTAAGACATTTTCCTCCCTCTCTAACCTGGAAACCCTGAGGCTCAGGGGTTATGTCTTTAAAGAACTGAGAGAAGGAAATCTAGATCCATTGCTCAGTCTTAGAAATCTAACAGTGTTGGATCTCGGGCTAATTTTATTAAAGTTGCTGACCTGAAAGTGTTCAAAGAATTCCCAGCTCTTAAATTCATAGACCTCTCAGTGAATAAAATTTCTCCTTCTTCAAGTGAAAGCAACTTTTATGGATTTTGCTCTAATCCTAGGATTTCAGTAGAGCAATACAACAGGCAAGTATTACAAGAGATGCATTATTTCAGGTATGATGAGTATGGGCGAAGTTGCAGGTCCAAAGACAAAGAGGCTGCTTCCTACCAATCTTCAGTTAACGAAGAGTGCCTGAAATACGGAGAAACTCTGGATTTAAGCAGAAACAACATATTTTTTATTAACCCCTCGGACTTCCGTGGTCTTAGTTTCCTCAGATGCCTCAACTTGTCAGGTAATGCAATAAGTCAAACTTTAAATGGAAGTGAATTCTACTATTTGTCTGGATTGAAATATCTGGATTTTTCTAACAACAGGATCGACTTGCTATACTCAACTGCTTTCAAAGAGCT

>46155 TLR7

CCACCCAATTTGTCATCTACTTTAAAGGAATTGTATATTTACAATAACATGATTCAAGTGATTCAAGAACAAGATTTAAGTGCCCTTCACAACCTAGAAATTCTTGATCTGAGTGGTAATTGCCCACGTTGCTATAATGCCCCATATCCTTGTATTCCCTGCCCCAAGAGCACAATTGAGATACATTCAAAGGCTTTTTATTCCCTGAAAAATTTAAGAATTTTGCGACTTCACAGTAACTCTCTTCAGAGCATACCCAGCATCTGGTTTAAAAACATCAGAAATCTCAAAGAGCTTGACCTCTCCCAAAATTTCCTCATAAAGGAGATTGGAGATGCTCAGTTTTTGAAGTTTATCCCTGGCCTTGTAGAGCTTGATTTGTCCTTTAATTTTGAACTGCAGATGTATTCTCCATTCTTGAATCTGTCTAAGACATTTTCCTCCCTCTCTAACCTGGAAACCCTGAGGCTCAGGGGTTATGTCTTTAAAGAACTGAGAGAAGGAAATCTAGATCCATTGCTCAGTCTTAGAAATCTAACAGTGTTGGATCTCGGGCTAATTTTATTAAAGTTGCTGACCTGAAAGTGTTCAAAGAATTCCCAGCTCTTAAATTCATAGACCTCTCAGTGAATAAAATTTCTCCTTCTTCAAGTGAAAGCAACTTTTATGGATTTTGCTCTAATCCTAGGATTTCAGTAGAGCAATACAACAGGCAAGTATTACAAGAGATGCATTATTTCAGGTATGATGAGTATGGGCGAAGTTGCAGGTCCAAAGACAAAGAGGCTGCTTCCTACCAATCTTCAGTTAACGAAGAGTGCCTGAAATACGGAGAAACTCTGGATTTAAGCAGAAACAACATATTTTTTATTAACCCCTCGGACTTCCGTGGTCTTAGTTTCCTCAGATGCCTCAACTTGTCAGGTAATGCAATAAGTCAAACTTTAAATGGAAGTGAATTCTACTATTTGTCTGGATTGAAATATCTGGATTTTTCTAACAACAGGATCGACTTGCTATACTCAACTGCTTTCAAAGAGCT

>46157 TLR7

CCACCCAATTTGTCATCTACTTTAAAGGAATTGTATATTTACAATAACATGATTCAAGTGATTCAAGAACAAGATTTAAGTGCCCTTCACAACCTAGAAATTCTTGATCTGAGTGGTAATTGCCCACGTTGCTATAATGCCCCATATCCTTGTATTCCCTGCCCCAAGAGCACAATTGAGATACATTCAAAGGCTTTTTATTCCCTGAAAAATTTAAGAATTTTGCGACTTCACAGTAACTCTCTTCAGAGCATACCCAGCATCTGGTTTAAAAACATCAGAAATCTCAAAGAGCTTGACCTCTCCCAAAATTTCCTCATAAAGGAGATTGGAGATGCTCAGTTTTTGAAGTTTATCCCTGGCCTTGTAGAGCTTGATTTGTCCTTTAATTTTGAACTGCAGATGTATTCTCCATTCTTGAATCTGTCTAAGACATTTTCCTCCCTCTCTAACCTGGAAACCCTGAGGCTCAGGGGTTATGTCTTTAAAGAACTGAGAGAAGGAAATCTAGATCCATTGCTCAGTCTTAGAAATCTAACAGTGTTGGATCTCGGGCTAATTTTATTAAAGTTGCTGACCTGAAAGTGTTCAAAGAATTCCCAGCTCTTAAATTCATAGACCTCTCAGTGAATAAAATTTCTCCTTCTTCAAGTGAAAGCAACTTTTATGGATTTTGCTCTAATCCTAGGATTTCAGTAGAGCAATACAACAGGCAAGTATTACAAGAGATGCATTATTTCAGGTATGATGAGTATGGGCGAAGTTGCAGGTCCAAAGACAAAGAGGCTGCTTCCTACCAATCTTCAGTTAACGAAGAGTGCCTGAAATACGGAGAAACTCTGGATTTAAGCAGAAACAACATATTTTTTATTAACCCCTCGGACTTCCGTGGTCTTAGTTTCCTCAGATGCCTCAACTTGTCAGGTAATGCAATAAGTCAAACTTTAAATGGAAGTGAATTCTACTATTTGTCTGGATTGAAATATCTGGATTTTTCTAACAACAGGATCGACTTGCTATACTCAACTGCTTTCAAAGAGCT

>46158 TLR7

CCACCCAATTTGTCATCTACTTTAAAGGAATTGTATATTTACAATAACATGATTCAAGTGATTCAAGAACAAGATTTAAGTGCCCTTCACAACCTAGAAATTCTTGATCTGAGTGGTAATTGCCCACGTTGCTATAATGCCCCATATCCTTGTATTCCCTGCCCCAAGAGCACAATTGAGATACATTCAAAGGCTTTTTATTCCCTGAAAAATTTAAGAATTTTGCGACTTCACAGTAACTCTCTTCAGAGCATACCCAGCATCTGGTTTAAAAACATCAGAAATCTCAAAGAGCTTGACCTCTCCCAAAATTTCCTCATAAAGGAGATTGGAGATGCTCAGTTTTTGAAGTTTATCCCTGGCCTTGTAGAGCTTGATTTGTCCTTTAATTTTGAACTGCAGATGTATTCTCCATTCTTGAATCTGTCTAAGACATTTTCCTCCCTCTCTAACCTGGAAACCCTGAGGCTCAGGGGTTATGTCTTTAAAGAACTGAGAGAAGGAAATCTAGATCCATTGCTCAGTCTTAGAAATCTAACAGTGTTGGATCTCGGGCTAATTTTATTAAAGTTGCTGACCTGAAAGTGTTCAAAGAATTCCCAGCTCTTAAATTCATAGACCTCTCAGTGAATAAAATTTCTCCTTCTTCAAGTGAAAGCAACTTTTATGGATTTTGCTCTAATCCTAGGATTTCAGTAGAGCAATACAACAGGCAAGTATTACAAGAGATGCATTATTTCAGGTATGATGAGTATGGGCGAAGTTGCAGGTCCAAAGACAAAGAGGCTGCTTCCTACCAATCTTCAGTTAACGAAGAGTGCCTGAAATACGGAGAAACTCTGGATTTAAGCAGAAACAACATATTTTTTATTAACCCCTCGGACTTCCGTGGTCTTAGTTTCCTCAGATGCCTCAACTTGTCAGGTAATGCAATAAGTCAAACTTTAAATGGAAGTGAATTCTACTATTTGTCTGGATTGAAATATCTGGATTTTTCTAACAACAGGATCGACTTGCTATACTCAACTGCTTTCAAAGAGCT
